# Proceedings of the 16^th^ Annual Conference on the Science of Dissemination and Implementation in Health

**DOI:** 10.1186/s13012-024-01370-y

**Published:** 2024-06-27

## I1 Introduction

### Gila Neta^1^, David A. Chambers^1^, Lisa Simpson^2^

#### ^1^National Cancer Institute, Rockville, MD, USA; ^2^AcademyHealth, Washington, DC, USA

*Implementation Science 2024*, **19(2):** I1

The National Institutes of Health and AcademyHealth, in collaboration with our co-sponsors, the Agency for Healthcare Research and Quality (AHRQ), the Patient Centered Outcomes Research Institute (PCORI), the Robert Wood Johnson Foundation (RWJF), and the US Department of Veterans Affairs (VA), hosted the 16^th^ Annual Conference on the Science of Dissemination and Implementation in Health in Arlington, VA on December 10-13, 2023.

The theme of this year’s conference was Raising Expectations for D&I Science: Challenges and Opportunities, which concentrated on capitalizing on methodological progress while also focusing attention on areas needing growth. Plenary sessions focused on key methodological aspects of the field, including a critical review of theories, models, and frameworks, a lively debate around the tensions between context-specific and generalizable knowledge to support dissemination and implementation efforts, and an exploration of study designs to harness implementation practice knowledge beyond the randomized controlled trial.

Conference participants included over 1400 researchers, practitioners, and other partners from around the world, representing 27 countries, eight of which are low- and middle-income countries (LMICs) in sub-Saharan Africa, Latin America, South and Southeast Asia, Eastern Europe, and the Middle East. We were pleased to welcome 121 trainees, five patient scholarship recipients, and 20 participants from LMICs, whose diverse perspectives added greatly to conference discourse. We again offered travel scholarships to 20 US-based participants, as well as travel scholarships to an additional 9 participants based in LMICs to enhance the diversity, equity, inclusion, and accessibility of the D&I Science community. For the first time, we were also able to provide scholarships to representatives from five community-based organizations.

The conference opened with an energizing keynote address from Mary Dixon Woods entitled “Doing Better at Doing Better: The Role of Co-design in Producing the Evidence for Dissemination and Implementation.” Dr. Dixon Woods walked us through three basic components of a health learning system (i.e., characterizing the problems, developing solutions, and evaluating those solutions), illustrating the importance of thinking through implementation and ensuring that researchers work together with practitioners at each of those steps.

The conference also included concurrent podium and poster sessions, discussion forums, and multiple networking events. The call for abstracts generated 1088 abstract submissions, including individual paper presentations, posters, and panel presentations spread across nine thematic tracks: Behavioral Health, Clinical Care Settings (separated into two tracks: Patient-Level Interventions and System-Level Interventions), Global Dissemination and Implementation Science, Promoting Health Equity and Eliminating Disparities, Health Policy Dissemination and Implementation Science, Prevention and Public Health, and Models, Measures and Methods, and Building the Future of D & I Science: Training, Infrastructure, and Emerging Research Areas. This supplement is organized by those tracks and includes 154 abstracts from the concurrent paper and panel sessions, which represents a variety of dissemination and implementation research funded by our conference sponsors as well as other agencies, organizations, and systems. The additional 616 abstracts from the poster sessions are not included here but can be viewed at https://academyhealth.confex.com/academyhealth/2023di/meetingapp.cgi/ModulePosterSessions/0.

For the second year in a row, we offered pre-conference workshops, which have grown in popularity. Five workshops were held in 2023 on topics as diverse as rapid qualitative methods and implementation costs and budget impact analysis. A special additional workshop on global implementation science had over 130 people attending.

The conference also featured a pre-recorded orientation to D&I with live Q&A for those new to the field, a social musical gathering based on attendee song requests, a new “first timers” session, yoga, poster walks, meet the editors, ancillary meetings, and daily morning coffee chats with D&I experts facilitating open discussions about key priorities for the field. The networking sessions were hugely popular and well attended, providing attendees with the opportunity to connect with the leaders in the field.

We look forward to welcoming attendees at the 17^th^ Annual D&I Science conference this December 8-11 in Arlington, VA at the Crystal Gateway Marriott.

## Behavioral Health

### S1 Implementation barriers and promising strategies to support the integration of psychiatric collaborative care in rural primary care settings

#### Elizabeth Austin^1^, Elsa Briggs^1^, Angel Chueng^2^, Erin LePoire^2^, Brittany Blanchard^3^, Amy Bauer^2^, Morhaf Al Achkar^3^, Diane Powers^2^

##### ^1^Department of Health Systems and Population Health, University of Washington, Seattle, WA, USA; ^2^University of Washington, Seattle, WA, USA; ^3^University of Washington, Seattle, USA

###### **Correspondence:** Elizabeth Austin (austie@uw.edu)

*Implementation Science 2024*, **19(2):** S1

**Background**: The Psychiatric Collaborative Care Model (CoCM) is a highly evidence-based model of behavioral health care delivery in primary care settings. Rural primary care (RPC) clinics may face unique barriers to implementation of CoCM that are important for implementation teams to understand. As part of a multisite implementation of CoCM in RPC clinics, we explored primary care staff and practice facilitator (PF) perspectives on barriers and promising implementation strategies for CoCM implementation.

**Methods:** This study followed a sequential explanatory mixed methods design. Clinics (n=23) were each assigned a trained PF who they met with monthly for 15 months to support CoCM implementation. Quantitative data included structured surveys completed by PFs at the conclusion of each monthly meeting with individual clinics (n=459 surveys) that asked PFs to report the frequency and strength of 16 pre-determined influencing factors for implementation. Survey data were analyzed descriptively and informed qualitative data collection. Qualitative data included key informant interviews with a purposive sample of 9 clinic staff and 2 PFs to expand on implementation experiences and recommended strategies. Qualitative analysts double-coded all transcripts using Rapid Assessment Process and a combination of inductive coding to identify themes and deductive coding to map implementations strategies to ERIC strategies.

**Findings:** All clinics (n=23) were located in low population density areas (average RUCA classification: 7). At baseline, 91% of clinics reported readiness for CoCM implementation. The strongest and most frequently-reported barriers reporting during implementation were: 1) the impact of the COVID-19 pandemic, 2) limited availability of site staff to participate in implementation activities, and 3) hiring of new staff to fill CoCM roles. Qualitative data further characterized the ways these barriers uniquely influenced RPC settings, including themes related to the influence of remote location, increased billing complexities, and maintaining a community focus. To mitigate these challenges, implementation teams emphasized the strategies of promoting adaptability, revising professional roles, identifying and preparing champions, and better organizing implementation meetings, offering several examples of strategies used in practice.

**Implications for D&I Research:** RPC settings face unique challenges to CoCM implementation, however several promising implementation strategies – when tailored to RPC contexts - may help.

**Primary Funding Source:** Insurer evaluation contract

### S2 Examining disparities in behavioral health treatment engagement in the implementation of a collaborative care for depression program

#### Allison Carroll^1^, Emily Fu^1^, Andrew Carlo^1^, Lisa Rosenthal^1^, Jeffrey Rado^1^, Jennifer Brockmeyer^1^, Sarah Philbin^1^, Inger Burnett-Zeigler^1^, Neil Jordan^1^, C. Hendricks Brown^1^, Jd Smith^2^

##### ^1^Northwestern University Feinberg School of Medicine, Chicago, IL, USA; Spencer Fox Eccles School of Medicine at the University of Utah, Salt Lake City, UT, USA

###### **Correspondence:** Allison Carroll (allison.carroll@northwestern.edu)

*Implementation Science 2024*, **19(2):**S2

**Background:**

Equitable access to behavioral health (BH) services is paramount to achieving equitable outcomes. Inequities exist at multiple points in the BH treatment pipeline, from identification of those in need of intervention, to enrollment, to treatment outcomes. This study examined disparities along the BH treatment pipeline in the context of a randomized implementation trial of collaborative care for depression.

**Methods:**

The Collaborative Behavioral Health Program (CBHP) was a randomized roll-out type 2 effectiveness-implementation study of collaborative care for depression in 11 primary care clinics within a single academic health system. We extracted data from the electronic health record for the first year of each clinic’s CBHP implementation (9/1/2018 to 1/31/2022). Demographic predictors were gender (male/female), race (minority/nonminority/unknown), ethnicity (Latinx/not Latinx/unknown), and age (years). BH treatment steps (1=occurred, 0=did not occur) were: (1) screened for depression (has patient health questionnaire [PHQ]), of all primary care patients, (2) referred to CBHP, of those eligible (PHQ-9 ≥10), (3) completed CBHP assessment, of those referred, (4) engaged in CBHP (attended ≥1 CBHP session), of those assessed, and (5) completed CBHP, of those engaged. We used individual logistic regression models to assess demographic predictors at each step.

**Findings:**

Of 89,008 patients seen by primary care clinicians during the first 12 months of CBHP implementation, 33.4% had ≥1 documented PHQ-2 score. Those screened were less likely to be of a minority race (B=-0.10, SE=0.03) or ethnicity (B=-0.11, SE=0.04). Of 967 patients with elevated PHQ-9 scores, 25.1% were referred to CBHP; those referred were less likely to be of a minority race (B=-0.35, SE=0.25). Further examination revealed disparities for specific racial/ethnic groups. Disparities were not identified among the 433 patients (41.8% of referred) who completed CBHP assessment, 347 patients (80.1% of assessed) who engaged in CBHP, and 76 patients (21.9% of engaged) who completed CBHP.

**Implications for D&I Research:**

Significant attrition of patients was observed at each step of the BH treatment pipeline. Racial and ethnic disparities emerged in screening and referral of primary care patients to collaborative care. Implementation strategies are needed to increase overall and equitable reach in BH screening and referral for the most vulnerable patients.

**Primary Funding Source:** Woman's Board of Northwestern Memorial Hospital

### S3 A theory-informed multi-level strategy for sustaining collaborative care models for depression in primary care settings (Transform DepCare): Impact on fidelity, clinical and reach outcomes across New York State

#### Nathalie Moise^1^, Amy Jones^2^, Danielle Gadbois^3^, Virna Little^4^, Elaine Fleck^1^, Andrea Duran^1^, Lisa Saldana^5^, Lisa Dixon^6^, Tom Smith^7^, Jay Carruthers^8^, Joseph Schwartz^9^

##### ^1^Columbia University Irving Medical Center, New York, NY, USA; ^2^New York State, New York State, Albany, NY, USA; ^3^New York State Office of Mental Health, Delmar, NY, USA; ^4^Institute for Family Health, New York, NY, USA; ^5^Chestnut Health Systems, Eugene, OR, USA; ^6^Box 100, New York State Psychiatric Institute, New York, NY, USA; ^7^New York State Office of Mental Health, New York, USA; ^8^New York State, Albany, NY, USA; ^9^Columbia University Irving Medical Center, New York, USA

###### **Correspondence:** Nathalie Moise (nm2562@cumc.columbia.edu)

*Implementation Science 2024*, **19(2):**S3

**Background:** In 2015, New York State (NYS) Office of Mental Health (OMH) launched the Collaborative Care Medicaid Program (CCMP), using technical assistance (TA) and Medicaid reimbursement to support CC implementation. To address barriers to sustainability, we piloted a multi-level strategy for sustainability.

**Methods:** From June 2021 to December 2022, we conducted a cluster randomized controlled trial of a multi-level sustainability strategy in 3 low-fidelity CCMP clinics. Informed by the Dynamic Sustainability Framework and Behavioral Change Wheel, our strategy (DepCare) involved (1) quarterly TA with care managers/administrators to promote quality improvement (QI)/problem-solving/learning health systems (2) primary care physician (PCP) education and (3) a digital patient psychoeducation/activation tool in a subset of CC-eligible patients (n=52). We employed descriptive statistics to compare pre-post (2021 vs. 2022) outcomes [mean rate/month] between pilot (n=25,829 patients) and state-wide (n=1,453,055 patients) CCMP clinics implementing CC in racially/ethnically diverse settings serving Medicaid patients. Outcomes included practice-level fidelity metrics (depression screening rates, monthly contact/engagement rates, psychiatry consultation rates for patients not improving); effectiveness (50% improvement in Patient Health Questionnaire-9); and reach (# of CC-enrolled patients/FTE care manager). Informed by Consolidated Framework for Implementation Research (CFIR) 2.0, we thematically analyzed TA meeting minutes to elucidate sustainability determinants.

**Findings:** Overall ≥1 champion/clinic attended every TA meeting (n=9, 21 attendees), prompting QI initiatives (e.g., medical assistant screening education, relapse prevention, automated registry, data integration). Preliminary analyses suggest greater pre-to-post improvements in the pilot vs. state-wide clinics for screening (72.4% to 82.6% vs. 64.0% to 66.5%), improvement (26.6% to 40.7% vs. 46.8% to 41.4%) and monthly contact/engagement (34.7% to 64.9% vs. 61.0% to 54.0%) but not psychiatry consultation (30.1% to 10.4% vs. 39.4% to 43.0%) rates or reach (43 to 36 patients/FTE/month vs. 51 to 56 patients/FTE/month). Key CFIR 2.0 constructs included psychiatrist turnover/resources, CC ineligible/psychosocially complex patients (need), low PCP motivation, billing capability/financing, remote communications, and compatibility.

**Implications for D&I Research:** Findings advance sustainability research, demonstrating the added benefit of a local TA strategy that promotes tenets of sustainability—training/adaptation/QI/learning systems. Our strategy did not impact long-term reach, though our separately reported findings on the doubling effect of a patient activation tool on CC enrollment suggests need for patient-level interventions.

**Primary Funding Source:** Agency for Healthcare Research and Quality

### S4 Trauma–focused cognitive behavioral therapy outcomes of a community based learning collaborative (CBLC): A comparison to standard learning collaboratives (LC)

#### Hannah Espeleta, Marin Kautz, Shelby Wade, Rochelle Hanson

##### Medical University of South Carolina, Charleston, SC, USA

###### **Correspondence:** Hannah Espeleta (espeleta@musc.edu)

*Implementation Science 2024*, **19(2):**S4

**Background**: Community-Based Learning Collaboratives (CBLCs) are promising training/implementation packages developed to provide multidisciplinary training and support for the sustained adoption of EBTs, such as Trauma-Focused Cognitive Behavioral Therapy (TF-CBT), among community agencies. CBLCs improve community-, organization-, and clinician-level factors related to EBT implementation and result in positive clinical outcomes for the children and families. CBLC’s involve multidisciplinary professionals across several youth-serving agencies and target an improved community response to childhood trauma; thus, they require more time/cost investment than the standard Learning Collaborative (LC) training model. Research is needed to compare CBLC and LC outcomes to inform decision making related to efficient and cost-effective training/implementation models.

**Methods**: The present study involved 437 community therapists participating in a TF-CBT LC (n=190) or CBLC (n=247). Analyses compared treatment outcomes for 1,460 youth (809, CBLC; 651, LC), ages 8-17, receiving TF-CBT from participating therapists. Youth and caregivers completed pre- and post-treatment measures of post-traumatic stress (PTS; Child PTSD Symptom Scale) and depression (Short Mood and Feelings Questionnaire–Short Version).

**Findings**: Mixed effect linear regressions with youth nested within therapists indicated that, at pre-treatment, no significant differences were observed across training models for child gender, age, or PTS. Youth in the CBLC were more likely to be White (*p*=.001) and to have higher depressive symptoms prior to treatment (*p*<.001). Youth from both CBLC-trained or LC-trained therapists demonstrated significant pre- to post-treatment decreases in PTS (*R*^2^_Marginal_=0.014, *p*=.008) and depressive symptoms (*R*^2^_Marginal_=0.032, *p* < .001). The interaction of clinical outcomes by training model was not significant for PTS (*p*=.08), suggesting outcomes from the CBLC and LC were equivalent. However, the statistically significant interaction for youth depressive symptoms (*p*=.03) suggested that the CBLC-trained therapists had better outcomes for youth depression than the LC model.

**Implications for D&I Research:** Results indicate that the LC and CBLC for TF-CBT have similar effects on child PTS; however, the multidisciplinary training model (CBLC) may support accurate identification of trauma symptoms that extend beyond PTS, which can improve appropriate treatment referrals and enhance outcomes for trauma exposed youth. Future directions and clinical implications will be further discussed.

**Primary Funding Source:** The Duke Endowment

### S5 Effectiveness and sustainment of the youth readiness intervention: An integrated behavior-change intervention for war-affected youth in Sierra Leone

#### Theresa Betancourt^1^, Nathan Hansen^2^, Adeyinka Akinsulure-Smith^3^, Jordan Farrar^4^, Matias Placencio-Castro^1^, Jordan Freeman^5^, Laura Bond^1^, Alethea Desrosiers^6^, Robert Brennan^4^

##### ^1^Boston College School of Social Work, Chestnut Hill, MA, USA; ^2^University of Georgia, Athens, GA, USA; ^3^The City College of New York, New York, NY, USA; ^4^Boston College, Chestnut Hill, MA, USA; ^5^Boston College School of Social Work, Research Program on Children and Adversity, Chestnut Hill, MA, USA; ^6^Brown University, Providence, RI, USA

###### **Correspondence:** Theresa Betancourt (theresa.betancourt@bc.edu)

*Implementation Science 2024*, **19(2):**S5

**Background:** The Youth Readiness Intervention (YRI) is a behavior change intervention for war-affected youth that has been tested and scaled in both education and livelihoods settings within Sierra Leone. In Sierra Leone, youth are at risk for poor psychosocial outcomes, which is exacerbated by contextual factors such as youth unemployment and underemployment. Mental health treatment remains scarce in Sierra Leone, and strategies to test and sustain evidence-based interventions across novel delivery platforms are critical.

**Methods:** In 2018/2019, the YRI was tested as a Hybrid Type-II Implementation-Effectiveness trial that used a cluster-randomized design to evaluate the YRI’s integration into a youth entrepreneurship program (ENTR). In total, 1,151 youth were enrolled in the study across all three study arms (ENTR only, ENTR and the YRI, and a control group). Primary effectiveness outcomes were emotion regulation, psychological distress (anxiety/depression) and interpersonal functioning, and primary implementation outcomes were dissemination and implementation indicators, and fidelity.

**Findings:** YRI+ENTR youth showed overall improvements in depression (β=-0.081, 95%CI: -0.124 – -0.038, d=-0.154), and anxiety (β=-0.043, 95%CI: -0.091 – 0.005, d=0.082) symptoms compared to control youth. Community leaders indicated that YRI+ENTR youth demonstrated improvements in overall performance in the ENTR compared to control youth (β=-0.114, 95%CI: 0.004 – 0.232, d=0.374). Qualitative data from the implementing agencies in the Collaborative Team, YRI facilitators and YRI participants revealed intimate partner relationships, coping strategies, externalizing and internalizing symptoms, all of which enabled youth to be more successful in their professional lives as well.

**Implications for D&I Research:** Now, four years later, a follow-up study (N = 590 youth) is ongoing to understand the long-term mental health and economic outcomes of the YRI and the ENTR. Findings from the Hybrid Type II and follow-up study will inform strategies for sustaining evidence-based interventions, using Collaborative implementation approaches that generate buy-in, and integration of behavior-change interventions into novel delivery platforms.

**Primary Funding Source:** National Institutes of Health

### S6 Therapist-reported barriers to delivery of trauma-focused cognitive behavioral therapy: Impact on youth treatment response and drop-out

#### Marin Kautz, Hannah Espeleta, Shelby Wade, Rochelle Hanson

##### Medical University of South Carolina, Charleston, SC, USA

###### **Correspondence:** Marin Kautz (marin.kautz01@gmail.com)

*Implementation Science 2024*, **19(2):**S6

**Background:** Trauma-focused cognitive behavioral therapy (TF-CBT) is an efficacious mental health treatment for youth with trauma sequalae. Despite extensive research, questions remain about factors that interfere with quality delivery of TF-CBT. For example, no studies to date have explored the impact of therapist-reported barriers on the delivery of TF-CBT on youth treatment response and reasons for treatment drop-out. Identifying the barriers that have the greatest impact on treatment delivery and drop out can inform strategies to integrate into training and implementation initiatives.

**Methods:** To address these issues, this study included 1,298 families receiving TF-CBT (n=681 completed treatment, n=617 did not complete) from 420 therapists enrolled in learning collaboratives between 2014-2021. Youth and their caregivers completed measures of post-traumatic stress (PTS) and depression, pre- and post-treatment. Therapists completed weekly surveys about each of their enrolled TF-CBT cases. Questions included reasons for discontinuing TF-CBT treatment and barriers for TF-CBT delivery (10,423 observations). The most frequent provider-level barriers included: “needing to discuss new events” and “difficulty engaging caregivers,” which occurred in 12.8% and 10.6% of reports, respectively. The frequency of barriers across all sessions was summed to create a total score for each youth. Multilevel modeling analyses estimated trajectories of barrier frequency for each youth, controlling for youth demographics.

**Findings:** Linear regressions indicated that greater total number of therapist-reported barriers and increasing frequency of barriers across the treatment period were associated with worse treatment outcomes for PTS, per caregiver-report. Greater frequency of the “poor attendance” barrier was associated with worse treatment response as measured by youth-report of PTS. The total number of barriers was not associated with the risk of drop-out, but as the rates of barriers increased across treatment, the risk for dropping out due to clinical instability increased.

**Implications for D&I Research:** Findings have implications for training initiatives to help therapists address difficulties with youth and caregiver engagement and poor attendance, which may improve treatment outcomes. Results suggest that TF-CBT augmentation with evidence-based crisis management and engagement strategies should be explored further to possibly increase retention, especially following early signs of clinical instability (e.g., suicidality, substance-use, or home environment instability). Further clinical and research implications will be discussed.

**Primary Funding Source:** Duke Endowment

### S7 Moving outside of research: Using determinants to improve the dissemination and implementation of sync in juvenile-justice settings

#### Nyssa Snow-Hill^1^, Amanda Barry^1^, Ugochinyere Onyeukwu^1^, Morgan Cohen^2^, Evelyn Rios^2^, Geri Donenberg^2^

##### ^1^DePaul University, Chicago, IL, USA; ^2^University of Illinois at Chicago, Chicago, IL, USA

###### **Correspondence:** Nyssa Snow-Hill (nsnow@depaul.edu)

*Implementation Science 2024*, **19(2):**S7

**Background:** Justice-involved youth experience disproportionate rates of sexually transmitted infections partly due to the increased likelihood of engaging in risky sexual behaviors. Supporting Youth to Navigate Choices (SYNC) is a sex education and health promotion program designed for justice-involved youth. It has demonstrated significant effects on sexual behavior, substance use, aggression, and recidivism. To ensure that the research on SYNC produces real-world improvements in the lives of justice-involved youth, the next step is to identify how to effectively disseminate and implement the program outside of the research context. Understanding the barriers and facilitators that have hindered or helped research-related implementation of SYNC can inform implementation and dissemination strategies to improve future adoption, implementation, and sustainment of the program in new juvenile justice settings.

**Methods:** SYNC facilitators and other stakeholders (n=18) who were involved in the SYNC efficacy and effectiveness studies participated in hour-long structured interviews guided by the Consolidated Framework for Implementation Research (CFIR). Each interview was coded using consensus coding according to CFIR domains and subdomains, and data were examined to identify patterns among those domains.

**Findings:** Participants noted the most determinants within the Inner Setting domain. Overall, they believed that there was a strong need for SYNC and indicated significant support for the intervention from staff and supervisors. Participants viewed the training, curriculum, and manual as strengths of the program, but had concerns regarding the length of the program and the quantity of materials involved. There was agreement that SYNC provides necessary skills and information to youth, but some facilitators felt uncomfortable discussing sexual health with youth. While participants enjoyed the training, there were concerns about the sustainability of the in-person training since it does not accommodate staff turnover, programming, and ongoing refreshers. The presentation will discuss how these determinants can inform implementation and dissemination strategies for juvenile justice settings.

**Implications for D&I Research:** To prevent the halt of evidence-based programs at the end of grant-funded research, community partners from those grants can assist in identifying what intervention components or implementation strategies need adapted to better translate evidence-based programs in complex settings, like juvenile-justice settings.

**Primary Funding Source:** National Institutes of Health

### S8 Studying team- and leadership-level implementation strategies for a pediatric chronic care model: Early practical and methodologic challenges

#### David Kolko^1^, Elizabeth A. McGuier^1^, Amy Kilbourne^2^, Eileen Thompson^3^, Renee Turchi^4^, Shawna Smith^5^, Ian Bennett^6^, Byron Powell^7^, Kimberly Hoagwood^8^

##### ^1^University of Pittsburgh School of Medicine, Pittsburgh, PA, USA; ^2^University of Michigan Medical School, Ann Arbor, MI, USA; ^3^Pennsylvania Chapter of the American Academy of Pediatrics, Medical Home Program, Media, PA, USA; ^4^Drexel University, Philadelphia, PA, USA; ^5^University of Michigan School of Public Health, Ann Arbor, MI, USA; ^6^University of Washington, Seattle, WA, USA; ^7^Washington University in St. Louis, St. Louis, MO, USA ^8^The Child Study Center at NYU Langone Medical Center, New York, NY, New York, NY, USA

###### **Correspondence:** David Kolko (kolkodj@upmc.edu)

*Implementation Science 2024*, **19(2):**S8

**Background:** Chronic and collaborative care models (CCMs) can improve the quality of behavioral healthcare, but their adoption in pediatric primary care is challenging. We are conducting a hybrid type 3 effectiveness-implementation trial of Doctor-Office Collaborative Care (DOCC), a CCM for disruptive behavior disorders and ADHD in pediatric primary care. The trial evaluates implementation strategies targeting the care team (TEAM) and leadership levels (LEAD). This presentation describes early challenges related to practice engagement and readiness, and how they were addressed to balance relevance and rigor.

**Methods:** The trial uses a 2x2 factorial design to evaluate the main and interactive effects of TEAM and LEAD. TEAM facilitation targets provider clinical competencies and team functioning/integration. LEAD facilitation targets implementation climate and implementation leadership. The study began in 2021 and is recruiting practices/providers and caregivers. Initial qualitative data are based on bi-weekly meetings with practice network leaders, monthly meetings with investigators, and tracking log impressions from the implementation facilitators.

**Findings:** Readiness challenges in this CCM implementation trial included the many “touches” needed to support the necessary research and clinical operations and the administrative and legal/research burdens created by using telehealth for all functions. There were also burdens associated with the CCM (e.g., registry use, CCM principles) and difficulty finding adequate candidates to fit CCM roles. Some practices may place value in the intervention but not as much in following CCM principles, and local champions are difficult to find. Modifications to improve practice recruitment and engagement included flexible timelines for enrollment, randomization by practice (vs. by wave), expanded study role definitions, and changes to the nature and timing of pre-implementation tasks for each site. Other strategies used to advance study aims included the use of single site IRB to minimize administrative and research burdens, building relationships early with leaders/champions, a greater focus on the integration of CCM principles from the onset, conducting two focused readiness checks, and clarifying practices’ commitment to the parameters of Facilitation before trial launch.

**Implications for D&I Research:** CCM implementation trials often present challenges. We will discuss how to balance practicality and rigor in the face of a rapidly changing clinical and financial context and its associated demands.

**Primary Funding Source:** National Institutes of Health

### S9 Assessment strategies for readiness to implement collaborative care for common mental disorders

#### Ian Bennett

##### University of Washington, Seattle, WA, USA

###### **Correspondence:** Ian Bennett (ibennett@uw.edu)

*Implementation Science 2024*, **19(2):**S9

**Background:** The Collaborative Care Model (CoCM) for common mental disorders is a complex intervention requiring the use of a psychiatric consultant and specialized care manager who provide support to primary care providers. The implementation of this model has proven to be difficult in part because the practice change required is novel and requires interdisciplinary work between behavioral and primary care teams. The assessment of readiness for implementation has been suggested to be critical to the tailoring of implementation support for complex interventions. We wished to assess the ability of a systematic checklist of readiness by implementation teams with external implementation facilitators to reduce variation in early implementation stages.

**Methods:** Twenty clinics participating in the Maternal Infant Dyad Implementation (MInD-I; NCT02976025) Type-III Implementation-Effectiveness trial (2017-2022) were included. Sites were identified from the OCHIN national network of community health centers as well as primary care settings providing prenatal care to at least 50 patients annually. External implementation facilitators utilized a checklist of key elements of implementation readiness and tailored their support based on the results. A CoCM specific version of the Stages of Implementation Completion (CoCM-SIC) was used to assess implementation outcomes across 8 stages with measures of proportion of tasks completed and duration of each stage.

**Findings:** Among the 20 sites that participated in this study, all had high proportions of task completion (>80%) in the pre-implementation stages (1-5). However, wide variation in proportion of tasks completed was found in later stages of the CoCM-SIC.

**Implications for D&I Research:** While a systematic checklist approach to assessment of readiness was associated with high rates of task completion in early stages of completion, there was a wide range in final implementation success later in the process. Additional work is needed to assess whether distinct strategies to address variation in readiness might improve implementation success of the CoCM intervention.

**Primary Funding Source:** National Institutes of Health

### S10 Learning how to adapt: Lessons learned from a clustered non-responder sequential multiple assignment randomized trial implementing a collaborative care model in primary care and community mental health clinics

#### Shawna Smith^1^, Amy Kilbourne^2^

##### ^1^University of Michigan School of Public Health, Ann Arbor, MI, USA; ^2^University of Michigan Medical School, Ann Arbor, MI, USA

###### **Correspondence:** Shawna Smith (shawnana@med.umich.edu)

*Implementation Science 2024*, **19(2):**S10

**Background:** Collaborative care models (CCMs) improve physical and mental health outcomes for people with mood disorders, but CCM adoption has been slow, especially in lower-resourced community-based practices. Adaptive implementation efforts, and particularly those that adapt implementation support to accommodate low implementation readiness or address early implementation failure) may help to inform scale-up and equitable reach. We discuss lessons learned from a trial comparing ways to tailor implementation support for community-based sites that failed to initially adopt a CCM.

**Methods:** Providers initially received support using a low-intensity implementation strategy, Replicating Effective Programs (REP). After 6 months, slower-responder clinics were randomized to add either External Facilitation (REP+EF) or External+Internal Facilitation (REP+EF/IF). Comparative effectiveness and moderators of REP+EF vs. REP+EF/IF were examined for both implementation (CCM delivery) and effectiveness (mental health quality of life, depression) outcomes. Qualitative process data on EF and EF/IF engagement and fidelity was also examined.

**Findings:** N=43 slower-responder clinics were randomized to receive REP+EF (N=21) or REP+EF/IF (N=22). Overall, EF/IF practices saw more CCM delivery than EF practices (Δ_EF/IF-EF_ = 4.4 patients, 95% CI = 1.87-6.87). Moderation analyses, however, revealed that only site with pre-randomization CCM delivery significantly benefitted from EF/IF (Δ_EF/IF-EF_ = 9.2 patients, 95% CI: 5.72, 12.63). Only 27 non-responder sites (63%) identified patients at baseline that were suitable for CCM; patients at EF clinics showed significantly larger improvement in functioning than patients at EF/IF clinics (Δ_EF/IF-EF_ =8.38; 95%CI=3.59, 13.18). Process data indicated challenges in implementing IF with fidelity at many clinics, and notably those with low implementation readiness.

**Implications for D&I Research:** Key lessons learned included: (1) for clinics struggling with implementation, higher-intensity implementation support did not outperform lower-intensity; (2) indicators of implementation readiness (e.g., patient identification) and early success (e.g., pre-randomization delivery) were useful in tailoring later implementation support; and (3) more intensive strategies requiring substantial site buy-in and oversight may be less effective when implementation readiness is lower. While readiness for implementation has traditionally been considered an important precursor to implementation efforts, differential levels of readiness may also signal how best to align implementation support—for example, by ensuring that implementation support is flexible or non-burdensome-while--also ensuring equitable implementation efforts.

**Primary Funding Source:** National Institutes of Health

### S11 Effectiveness, acceptability, and feasibility of co-delivery of two mass drug administrations (Soil-transmitted helminths and Onchocerciasis) along with integrations of behavioral change intervention: Implementation research in oromia, Ethiopia

#### Sudhakar Morankar, Zewdie Birhanu

##### Aba Jifar Palace Road, Jimma University, Jimma, Oromia, Ethiopia

###### **Correspondence:** Sudhakar Morankar (morankarsn@yahoo.com)

*Implementation Science 2024*, **19(2):**S11

**Background:** Increased global attention focused on health campaign effectiveness in terms of meeting Neglected Tropical Diseases elimination goals through the integration of services for effective and efficient co-delivery. This study assessed effectiveness and acceptability of co-delivery of Mass Drug Administration (MDAs) for Onchocerciasis chemotherapy and deworming of children and reproductive age women complemented with Social and Behavioural Change Communication (SBCC) interventions to promote appropriate knowledge and practices.

**Methods:** Implementation research involving mixed methods with a pre-posttest approach was conducted in ten villages between June 2021 and September 2022. Formative qualitative assessment through focus group discussions and key informant interviews was conducted to explore enablers and barriers for co-delivery. Household survey involving 732 households was conducted to assess communities’ knowledge, attitude & practice (KAP). Informed by the formative assessments, co-delivery strategy was developed through a participatory process involving all stakeholders. Health extension workers (HEWs), supported by community volunteers, co-administered the MDAs in May 2022. KAP integrated post-campaign coverage validation survey was conducted on 776 households and qualitatively through FGDs with beneficiaries, and KIIs.

**Findings:** Co-delivery strategy achieved treatment coverage of 89.5%, 84.1%, and 83.2% for Onchocerciasis, STH, and combination (IVM plus ALB/MEB) with no adverse events. Communities’ overall satisfaction (91.6%) and intention to receive co-administration in future (96.3%) was quite high. Communities and stakeholders perceived that co-delivery was effective, acceptable, and feasible. They accepted co-delivery due to direct engagement of HEWs in co-administration; perceived fairness, and equity; transparency; and impartiality in the distribution of drugs, perceived quality, effective management of drugs; improved quality and timely documentation and reports. Major challenge was that community drug distributors (CDD) in a few villages were reluctant to engage because they perceived that their role as CDD was negated.

**Implications for D&I Research:** The co-delivery led by the HEWs and supported by community volunteers was appropriate; acceptable; feasible and produced high treatment coverage promising to contribute NTD targets. It is worthy of adoption, continuation, and scaling up. Effectively engaging health campaign actors at multiple level is vital for planning, designing and implementing co-delivery strategy including community. Co-administration of medicine through health campaign needs to be supported by well-designed SBCC interventions.

**Primary Funding Source:** Health Campaign Effectiveness, USA

### S12 Implementation of mindful awareness in body-oriented therapy (MABT) as a chronic pain treatment modality in an integrative health clinic: A mixed methods pilot study

#### Erin Abu-Rish Blakeney^1^, Kathryn Hansen^2^, Cynthia Price^3^

##### ^1^University of Washington School of Nursing, Seattle, WA, USA; ^2^Vanderbilt University Medical Center, Nashville, TN, USA; ^3^University of Washington School of Nursing, Seattle, USA

###### **Correspondence:** Erin Abu-Rish Blakeney (erin2@uw.edu)

*Implementation Science 2024*, **19(2):**S12

**Background:** Treatments that prioritize mind-body complementary and integrative health (CIH) therapies are now considered best practice for chronic pain. Yet, these treatments are prescribed less than 30% of the time and few studies have focused on implementation of these modalities into clinical care. This abstract reports on implementation outcomes assessed before, during, and after implementation of an evidence-based CIH treatment, Mindful Awareness in Body-oriented Therapy (MABT), in an integrative health clinic. MABT, delivered individually by massage therapists using an 8-week protocol, was designed to reduce symptomatic distress through teaching interoceptive awareness skills for self-care and emotion regulation. In previous studies MABT demonstrated statistically significant reductions in physical symptoms and substance use and improvements in emotion dysregulation, interoception, and depressive symptoms.

**Methods:** This mixed methods pilot implementation study examined the uptake of MABT for community-based patients with chronic pain within the CIH clinic. Key implementation outcomes among clinic staff were examined. Implementation strategies and outcomes were selected and defined using the Expert Recommendations for Implementing Change (ERIC) and Proctor Implementation Outcome Framework. The primary implementation strategy clusters were stakeholder education, iterative strategies, and infrastructure change. Targeted implementation outcomes were: acceptability, appropriateness, adoption, feasibility and penetration. Data were collected from multiple sources, including a) surveys of clinic staff at 7 time points over 20 months, b) focus groups with clinic staff stakeholders, and c) electronic health records that tracked MABT referrals and appointments.

**Findings:** Clinic staff surveys (n=190) showed high MABT acceptability and appropriateness on all surveys following initial presentation of the MABT approach. High acceptability and appropriateness were even more apparent in focus group results which highlighted that MABT addresses a gap in individualized mind-body approaches for symptom management and regulation among those with chronic pain. Adoption was high, with 70 patient referrals placed by clinicians, resulting in 42 patients attending MABT sessions; the majority of referrals were from Nurse Practitioners. 71% of referred patients completed 6-8 MABT sessions. Primary referral barriers were cost and availability of appointments.

**Implications for D&I Research:** Results demonstrate high implementation feasibility of MABT within a CIH clinic for chronic pain.

**Primary Funding Source:** University of Washington School of Nursing Research Intramural Funding Program

### S13 Informing integration of psychiatric care with other gender-affirming services

#### Teddy Goetz^1^, Courtney Wolk^2^

##### ^1^University of Pennsylvania Health System, Philadelphia, PA, USA; ^2^Perelman School of Medicine, University of Pennsylvania, Philadelphia, PA, USA

###### **Correspondence:** Teddy Goetz (teddy.goetz@pennmedicine.upenn.edu)

*Implementation Science 2024*, **19(2):**S13

**Background:** Transgender, non-binary, and/or gender expansive (TNG) individuals experience disproportionately high rates of mental illness and unique barriers to accessing psychiatric care. Delivering psychiatric care collaboratively with other gender-affirming health services may improve engagement, but little published literature describes patient and clinician stakeholders’ perspectives on such a care delivery model to inform implementation activities. Formative evaluations conducted pre-implementation can help optimize interventions and increase the likelihood of successful implementation.

**Methods:** In this qualitative pre-implementation study, we elicited TNG patient (n=11) and gender-affirming care clinician (n=10) needs and preferences for optimizing the implementation of psychiatric care and integration with other gender-affirming clinical services. Semi-structured qualitative interview guides were developed informed by the Consolidated Framework for Implementation Research (CFIR) to ensure uniform inclusion and sequencing of topics and allow for valid comparison across interviews. We utilized a rapid analysis procedure to produce a descriptive analysis for each stakeholder group, identifying challenges of and opportunities in offering gender-affirming psychiatric care in collaboration with physical healthcare to inform planning for a subsequent hybrid effectiveness/implementation trial.

**Findings:** Patient stakeholders represented multiple ages (mean=41.6, SD=8.1 range=23-38 years), genders (non-binary=7; trans man/transmasculine=4), and races (Asian/Pacific Islander=2; Black=2; white=5; multiple=2). All had multiple psychiatric diagnoses and accessed primary care in the past year. Clinicians were physicians (n=5), family nurse practitioners (n=3), midwives (n=2), and physician’s assistants (n=1). Nine provided primary care; all cared for adults and three also for youth. Stakeholders unanimously preferred embedding psychiatry within primary care instead of siloed service models. All participants preferred psychiatry appointments be face-to-face and all gender-affirming care clinicians wanted increased access to psychiatric consultations. The need for flexible, tailored care was emphasized. Facilitators identified included taking insurance, telehealth, clinician TNG-competence, and protecting time for clinicians to collaborate and obtain consultation.

**Implications for D&I Research:** This health equity pre-implementation project engaged TNG patients and gender-affirming care clinicians to inform development of an ongoing hybrid type 1 trial investigating effectiveness and implementation outcomes of gender-affirming psychiatric services embedded within primary care. If successful, this study could provide a model for increasing access to psychiatric care for the TNG community.

**Primary Funding Source:** National Institutes of Health

### S14 Factors associated with successful alcohol screening and brief intervention implementation and sustainment in adult primary care

#### Stacy Sterling^1^, Yun Lu^2^, Felicia Chi^1^, Thekla Ross^1^, Christina Grijalva^1^

##### ^1^Kaiser Permanente Northern California, Oakland, CA, USA; ^2^Kaiser Permanente Division of Research, Oakland, CA, USA

###### **Correspondence:** Stacy Sterling (stacy.a.sterling@kp.org)

*Implementation Science 2024*, **19(2):**S14

**Background:** Hazardous drinking is a significant public health problem affecting approximately 20% of U.S. adult primary care patients. Clinical trials have documented the efficacy and effectiveness of Alcohol Screening and Brief Intervention (ASBI) at reducing hazardous use. Studies have begun to study ASBI implementation, but questions remain regarding optimal strategies for implementation and sustainment. Kaiser Permanente Northern California (KPNC) implemented systematic ASBI in adult primary care in 61 facilities in 2014. Guided by the PRISM (*Practical, Robust Implementation and Sustainability Model*) framework domains (Intervention, External Environment, Implementation Infrastructure, and Recipients, we used 8 years of *electronic health record (EHR) data*, combined with *primary care provider surveys* to characterize ASBI implementation and sustainment and test whether and how various factors are associated with screening and BI rates.

**Methods:** Using EHR data, we calculated yearly screening rates of adults with a primary care visit, and brief intervention (BI) rates among those with a positive screen, (reporting alcohol consumption exceeding the age and gender specific daily and weekly low-risk National Institute on Alcohol Abuse and Alcoholism guidelines), for each of 61 medical facilities from 2014 to 2021. We collected web-based survey data from primary care providers (n=740) to assess perspectives on ASBI implementation and sustainability, and generated PRISM domain summary scores for each facility.

**Findings:** As of 12/31/2021, there were 15,364,074 screenings of 4,575,927 unique patients (overall rate of 91%), and 1,148,533 brief interventions (BIs) delivered to 457,296 unique patients (a cumulative rate of 59%). After adjusting for patient panel characteristics (size, age, sex, race/ethnicity and socio-economic status), we found that facilities with higher “Infrastructure/Capacity” scores, indicating more robust implementation capacity, had higher screening and BI rates; and facilities with higher “Recipients” scores, indicating greater perceived patient and clinician/staff needs, had higher BI rates. We found significant variations in associations between “Infrastructure/Capacity” scores and ASBI rates across years.

**Implications for D&I Research:** Organizational capacity and structures which support implementation, and the perceived needs of clinical staff and patients, were both associated with more robust implementation and/or sustainability. Results provide concrete data on factors which facilitate successful ASBI implementation and sustainability and can inform ASBI implementation efforts in other healthcare systems.

**Primary Funding Source:** National Institutes of Health

### S15 Using implementation and behavioral science to leverage large language models for scaling up evidence-based practice: A case example

#### Nadia Minian^1^, Mackenzie Earle^1^, Sowsan Hafuth^1^, Kamna Mehra^1^, Ryan TingAKee^1^, Jonathan Rose^2^, Scott Veldhuizen^1^, Laurie Zawertailo^1^, Matt Ratto^2^, Peter Selby^3^

##### ^1^Centre for Addiction and Mental Health, Toronto, ON, Canada; ^2^University of Toronto, Toronto, ON, Canada; ^3^Family and Community Medicine, University of Toronto, Toronto, ON, Canada

###### **Correspondence:** Nadia Minian (Nadia.Minian2@camh.ca)

*Implementation Science 2024*, **19(2):**S15

**Background**: There is a growing interest in leveraging the capabilities of large language models, specifically conversational agents embedded in health apps (healthbots), to accelerate and enhance the implementation of evidence-based practices. However, numerous challenges and considerations must be addressed to ensure their ethical and effective utilization. In this presentation we will outline the process we used to co-design of a theory informed rule-based healthbot, aimed at scaling up the findings of a 2019 Cochrane review that demonstrated the effectiveness of behavioral supports in enhancing adherence to smoking cessation medications, such as varenicline.

**Methods:** Using the Discover, Design and Build, and Test (DDBT) framework the study includes three phases: 1. A rapid review of the literature; interviews with 20 patients and 20 healthcare providers to understand barriers and facilitators to varenicline adherence (Discover phase); 2. Wizard of Oz test to design the conversational agent and get a sense of the questions has to be able to answer (Design phase) and 3. Building, training and beta-testing the conversational agent (Building and Testing phases) where the Non-adoption, Abandonment, Scale-up, Spread, and Sustainability framework is used to develop the healthbot using the simplest sensible solution. We used the COM-B (Capability, Opportunity, Motivation-Behaviour) model of behaviour change and it associated framework, the Theoretical Domains Framework, to collect our data and organize the findings. We made sure that all features we included in the healthbot were affordable, practical, effective, acceptable, and addressed equity as well as potential side effects (APEASE criteria).

**Findings:** We identified that the following features as important to embed in the healthbot: setting goals, action planning, behavioural contracts, feedback on medication adherence, monitoring on medication adherence and smoking cessation, answering questions in a factual and supportive manner, providing prompts and rewards and coming from a credible source.

**Implications for D&I Research:** This case study exemplifies the potential of leveraging large language models, and existing frameworks, to scale up evidence-based practices. It will be critical to study its adoption at an individual and practice level, and identify the needed to embed it in the healthcare system.

**Primary Funding Source:** CIHR

### S16 Assessing fidelity to a complex evidence-based intervention in a pragmatic implementation initiative

#### Sonya Gabrielian^1^, Camilla Cummings^2^, Gracielle Tan^2^, Lisa Edwards^2^, Daniel Herman^3^, Erin Finley^1,4^, Kristina Cordasco^1^

##### ^1^Veterans Health Administration, VA, Greater Los Angeles Healthcare System HSR&D Center for the Study of Healthcare Innovation, Implementation & Policy, Los Angeles, CA, USA; ^2^US Department of Veterans Affairs, Los Angeles, CA, USA; ^3^Hunter College, New York, NY, USA; ^4^UT Health San Antonio, San Antonio, TX, USA

###### **Correspondence:** Sonya Gabrielian (Sonya.Gabrielian@va.gov)

*Implementation Science 2024*, **19(2):**S16

**Background:**

It is challenging and time-intensive to assess fidelity to complex evidence-based behavioral health interventions, particularly in low-resourced community-based settings. We describe a mixed methods approach to assessing fidelity to Critical Time Intervention (CTI)—an evidence-based, time-limited case management practice for homeless-experienced persons with behavioral health disorders—developed for a pragmatic implementation initiative.

**Methods:**

CTI fidelity is traditionally assessed using site visits, which enable multi-method, ethnographic data collection and document review. Using literature review, expert consultation, and iterative pilot testing with 2 community-based homeless service agencies that participated in a CTI implementation pilot, we developed a pragmatic, scalable CTI fidelity assessment process. This process integrates data from: a CTI implementation self-assessment designed to enhance provider practice; 90-minute videoconferences with case managers to collaboratively review charts from at least two randomly selected “exemplar cases” for CTI’s core components; and field notes from case manager narratives during these videoconferences. At 12 months after CTI implementation began at 11 additional homeless service agencies across the nation, we employed this process to assess fidelity and conducted semi-structured interviews with agency case managers and supervisors (n=16) to contextualize our findings.

**Findings:**

All case managers self-assessed their CTI practices as either well-implemented or ideally-implemented. Videoconference data suggested that all 11 agencies had limited fidelity to at least one of CTI’s core components; three agencies had inadequate fidelity to the overall practice. Qualitative interviews highlighted explanations for disparate results between the self-assessment and exemplar case data, including staff turnover (resulting in limited understanding of CTI’s core components) and agency mandates (e.g., for case management visit frequency that were misaligned with CTI).

**Implications for D&I Research:**

Streamlined approaches to assessing fidelity to community-based behavioral health interventions are important for pragmatic, large-scale implementation efforts. Integrating self-assessment data and targeted case review is a potential strategy that can be applied to other multifaceted evidence-based practices in low-resourced settings. Supplementing fidelity assessment data with qualitative interviews may be particularly useful to contextualize disparate findings between self-assessments and case reviews, and to shape feedback to providers and agencies that enhances adherence to interventions’ core components and improves participant outcomes.

**Primary Funding Source:** Department of Veterans Affairs

### S17 Developing implementation strategies to increase uptake of text messaging interventions for unhealthy alcohol use in emergency departments

#### Megan O'Grady^1^, Sandeep Kapoor^2^, Laura Harrison^3^, Nancy Kwon^3^, Adekemi Suleiman^1^, Fred Muench^4^

##### ^1^University of Connecticut School of Medicine, Farmington, CT, USA; ^2^Zucker School of Medicine at Hofstra/Northwell, Hempstead, NY, USA; ^3^Northwell Health, New Hyde Park, NY, USA; ^4^Partnership to End Addiction, New York, NY, USA

###### **Correspondence:** Megan O'Grady (ogrady@uchc.edu)

*Implementation Science 2024*, **19(2):**S17

**Background:**

Alcohol consumption and use of alcohol-related acute care have increased. mHealth interventions are effective at improving alcohol use outcomes, but are rarely offered in clinical settings. Development of implementation strategies to adopt mHealth interventions for alcohol use in emergency departments (EDs) has been limited. The purpose of this study is to describe a data-based and stakeholder-engaged process using intervention mapping to develop implementation strategies for text messaging interventions for unhealthy alcohol use (UAU) in EDs.

**Methods:**

First, we used an explanatory, sequential mixed-methods approach to identify barriers and facilitators to implementing text messaging interventions for UAU. Electronic health record data and surveys with department chairs from 17 EDs were used to identify EDs with low vs. high implementation potential. We then interviewed staff (n = 18) and patients (n = 21) within those EDs and conducted rapid qualitative analysis. We incorporated our qualitative findings into a 4-step, stakeholder-engaged intervention mapping process to identify and select implementation strategies. Stakeholders (n = 13) met three times, providing insight on qualitative findings, change objectives, implementation strategies, and implementation materials; they also quantitatively ranked potential implementation strategies. The i-PARIHS framework guided study activities.

**Findings:**

Patients indicated interest in receiving the text intervention for different drinking goals: cut down, quit, prevent future heavy drinking. They indicated seeing it as a source of: support, information, health improvement, symptom reduction, relationship improvement. Patients stressed that empathy should be expressed by ED staff offering patients the intervention. Staff suggested a high patient need for the intervention, but challenges in how to offer it. A team-based approach was favored, to not burden any one team member. Ensuring staff understand the potential patient benefit was emphasized. Intervention mapping resulted in the following implementation strategies: champions, increasing patient demand, automated patient follow-up after 1 week, learning collaboratives, PDSA cycles, educating team members, data audit/feedback, and adjusting relevant roles.

**Implications for D&I Research:**

Using both data and stakeholder input to develop implementation strategies may increase adoption and reach of evidence-based practices in clinical settings. This data-based, mixed-method, stakeholder engaged intervention mapping process resulted in an actionable multi-component implementation strategy that can be tested in a pilot cluster-randomized trial.

**Primary Funding Source:** National Institutes of Health

### S18 Emerging technology and implementation of evidence-based collaborative physical health care for persons with serious mental illness

#### Emily Woltmann

##### University of Michigan Medical School, Ann Arbor, MI, USA

###### **Correspondence:** Emily Woltmann (ewoltman@med.umich.edu)

*Implementation Science 2024*, **19(2):**S18

**Background:** Persons with serious mental illnesses (SMI) experience disparities in health care and are more likely to die from physical health conditions including cardiovascular disease than the general population. Behavioral health homes are used in public sector mental health programs to deploy collaborative care to improve physical health for those with SMI. During the COVID-19 pandemic, these programs often responded to new barriers to receiving these services by employing technological strategies (e.g., telemedicine, secure messaging), and many sites are considering permanent adoption of these new technologies. To this end, this study describes technological barriers to implementing or sustaining behavioral health homes experienced by community mental health workers during the COVID-19 pandemic and the strategies used to address these challenges.

**Methods:** In-depth qualitative interviews were conducted among mental health providers (N=94) from 21 community mental health programs in Maryland and Michigan. Interview responses related to implementing and sustaining health homes during the pandemic were coded and themes were analyzed using a grounded theory approach.

**Findings:** 72 staff members (77%) across all 21 sites discussed COVID-related modifications to delivery of their behavioral health homes, and staff members at 13 sites noted that technological changes in the delivery of their services were likely to be enduring. Technological implementation barriers/strategies were identified across multiple levels (client, provider, mental health system). Frequently mentioned client-level barriers included access to, and ability to use, new telehealth technology, and challenges with engaging with behavioral health home staff during video visits. Mental health system barriers included reduced ability to collect biometric data, perceptions that online staff trainings were not as effective as in-person training, and difficulty translating interventions to a telehealth format. Frequently discussed strategies included increasing clients’ access to technology and coaching clients on technology use. Systems-level strategies included allocation of funding for technology needs and new processes for remote monitoring of biometric data.

**Implications for D&I Research:** Frontline behavioral health providers reported a number of potential barriers and strategies to technology use at the consumer and organizational levels. This in-depth qualitative analysis across a large provider sample may inform improved tailoring of technology-focused behavioral health interventions and implementation strategies in community mental health settings.

**Primary Funding Source:** National Institutes of Health

## Building the Future of D&I Science: Capacity Building, Infrastructure, and Emerging Research Areas

### S19 Building an implementation community in Pittsburgh

#### Shari Rogal

##### Department of Surgery, University of Pittsburgh, Pittsburgh, PA, USA

###### **Correspondence:** Shari Rogal (rogalss@upmc.edu)

*Implementation Science 2024*, **19(2):**S19

**Background:** Interdisciplinarity is essential to building D&I infrastructure in academic settings. However, despite the many barriers to interdisciplinary collaboration, there is a paucity of recommendations for addressing them. Investigators at the University of Pittsburgh and the Pittsburgh VA aimed to build D&I capacity and collaboration in the region through an inclusive D&I community. Here we describe how these leaders adapted D&I capacity-building strategies to local conditions with an emphasis on overcoming barriers to interdisciplinary work.

**Methods:** Using a snowball approach, we gathered investigators, trainees, and implementers to coalesce a grassroots community. We iteratively assessed participant needs and interests to develop a sustainable and inclusive Dissemination and Implementation Science Collaborative (DISC). Our annual needs assessment has informed changes to group activities including workgroups, seminars, and mentoring, and shaped the development of educational materials.

**Findings:** DISC has grown to over 170 investigators, trainees, and community partners from across the health sciences. Our bimonthly meetings include a seminar series featuring national experts, implemented in partnership with the CTSI, with 40-60 attendees. An overabundance of potential mentees relative to self-identified experts/mentors led us to develop mentoring “pods” of 4-6 members. Two methodologically-focused workgroups developed organically: one (now independently funded) focused on assessing evidence for implementation strategies and one focused on de-implementation. We created a website and other educational resources targeted toward disseminating foundational D&I capacity: a YouTube series, currently with over 1,800 views, and a D&I curriculum, GTI Teach, disseminated through the *Journal of Clinical and Translational Science*. To support community partnership interests, we provide a monthly forum for presentations by local non-profit organizations. The addition of a D&I-focused core to the CTSI has allowed D&I frameworks to be incorporated into research all along the translational continuum, including work beyond university walls in marketing and community engagement.

**Implications for D&I Research:** Strategies for nurturing interdisciplinarity essential to the development of DISC have included: disseminating expertise through small, mixed-discipline mentoring “pods;” forging connections with community partners; and providing an inclusive environment with cross-cutting group projects. Built by junior investigators, Pittsburgh's DISC offers a unique approach to increasing D&I capacity.

**Primary Funding Source:** National Institutes of Health

### S20 Collaborative community-building at Penn

#### Meghan Lane-Fall

##### University of Pennsylvania Perelman School of Medicine, Philadelphia, PA, USA

###### **Correspondence:** Meghan Lane-Fall (meghan.lane-fall@uphs.upenn.edu)

*Implementation Science 2024*, **19(2):**S20

**Background:** The ability to conduct paradigm-shifting dissemination and implementation (D&I) research requires capacity building across professional disciplines, fostering collaborative relationships, and equipping established investigators to incorporate implementation science approaches into their work. The Penn Implementation Science Center at the Leonard Davis Institute of Health Economics (PISCE@LDI) was founded in 2018 to support implementation science at the University of Pennsylvania.

**Methods:** Initial research community building efforts included local outreach to current and former students in health services research and related disciplines; established investigators including behavioral scientists; and health system and center leaders focused on population health. From this initial group, the community grew via word of mouth, educational program outreach, and continued outreach to institutional leaders. PISCE@LDI activities include seminars, works in progress sessions, a certificate program, a la carte coursework, and consultation and technical assistance. The program is supported financially primarily through revenues from educational programs and institutional support to center leaders. Metrics of effectiveness include the number of people engaged with the center, number and success of consultations (e.g., grant submissions, grants funded, publications), federal grants, event attendance, and training outcomes (e.g., number of trainees, training competencies achieved).

**Findings:** PISCE@LDI has grown to a community of more than 600 members. The Penn implementation science grant portfolio has similarly grown over time, now exceeding $50M. More than 500 individuals in and outside the institution have undertaken implementation science coursework led by center faculty. Ongoing challenges include maintaining sustainable funding, meeting demand for implementation science consultations, providing mentorship to early stage faculty, and recruiting mid- and late-career implementation scientists to the institution.

**Implications for D&I Research:** Ongoing, multipronged efforts are needed to build and sustain D&I communities. The experience of centers like PISCE@LDI can inform capacity building efforts at other institutions seeking to grow their D&I research.

**Primary Funding Source:** National Institutes of Health

### S21 Building capacity for dissemination and implementation research and practice at Washington University in St. Louis and beyond

#### Byron Powell

##### Washington University in St. Louis, St. Louis, MO, USA

###### **Correspondence:** Byron Powell (bjpowell@wustl.edu)

*Implementation Science 2024*, **19(2):**S21

**Background:** Universities play a central role in generating and disseminating knowledge of dissemination and implementation (D&I) research and practice to diverse audiences. Through the lens of the Center for Dissemination and Implementation at Washington University in St. Louis, this presentation will focus on efforts to build capacity for D&I at Washington University in St. Louis and beyond by focusing on opportunities for learning, research support, connection, and cross-cutting efforts to infuse methodological innovation in the field.

**Methods:** We provide an overview of past, present, and future efforts to build capacity for D&I locally and internationally. We also discuss ongoing challenges and opportunities for growth, both locally and in the field of D&I.

**Findings::** Washington University has created opportunities for learning about D&I, including an introductory workshop for investigators, a seminar series that features international experts in D&I and adjacent fields, four graduate-level courses that are central to both a certificate program and a PhD-level concentration for public health and social work, and multiple fellowship programs that train researchers nationally and internationally (e.g., Implementation Research Institute; HIV, Infectious Disease and Global Health Implementation Research Institute; Institute for Implementation Science Scholars). Research in D&I is supported through multiple mechanisms, including opportunities for consultation and collaboration through the Dissemination and Implementation Research Core, an annual Proposal Development Bootcamp, and multiple forms of pilot funding supported through the Center for Dissemination and Implementation (e.g., seed funding for pilot and small grants, conceptual and methodological review grants, rapid add-on funding, methods add-on funding, and D&I symposium funding). Recently formed initiatives focus on integrating D&I within infectious diseases and advancing methods and meta-science in dissemination. Opportunities for networking and connection include the Washington University Network for Dissemination and Implementation Research (~5 half-day meetings/year), an annual “D&I Day,” and WUNDIR Cafés, each of which serves as an opportunity for sharing cutting-edge D&I methods as well as social connection.

**Implications for D&I Research:** This presentation will convey lessons learned from ongoing D&I capacity-building efforts; discuss challenges related to avoiding stagnation, broadening transdisciplinary collaborations, and meeting the needs of diverse audiences; and discuss future directions to advance D&I research and practice internationally.

**Primary Funding Source:** National Institutes of Health

### S22 Strengthening capacity for implementation research: Learnings from the global alliance for chronic diseases implementation science school

#### Zahra Aziz^1^, Tilahun Haregu^2^, Isobel Bandurek^3^, Morven Roberts^3^, Kevin Mao^4^, Brian Oldenburg^2,5^

##### ^1^School of Public Health and Preventive Medicine, Monash University, Melbourne, VIC, Australia; ^2^Baker Heart and Diabetes Institute, Melbourne, VIC, Australia; ^3^Global Alliance for Chronic Diseases, London, United Kingdom; ^4^University of Melbourne, Melbourne, VIC, Australia; ^5^LaTrobe University, Melbourne, VIC, Australia

###### **Correspondence:** Zahra Aziz (zahra.aziz@monash.edu)

*Implementation Science 2024*, **19(2):**S22

**Background:**

Many contextual and political factors have hindered the implementation of evidence-based interventions and policies to tackle non-communicable diseases (NCDs) prevention and control, including building robust capacity at national and local levels. Implementation research capacity strengthening initiatives, although increasing in number, are still limited and are not accessible for the majority of healthcare professionals, globally. The Global Alliance for Chronic Disease (GACD) brings together 12 national health and medical research funding agencies with the common mission to reduce the burden of chronic NCDs globally. As part of its mission, GACD has also been supporting a range of implementation research capacity strengthening activities since 2014, through three main channels: Implementation Science Workshops, Implementation Science Schools, and most recently, through the development of the GACD Implementation Science e-Hub (https://implementationscience-gacd.org/), which is a free online publicly available resource to advance knowledge and practice in implementation science in relation to NCDs.

This paper describes the design, delivery, and evaluation of the three annual GACD Implementation Science Schools (ISS) that utilised the GACD Implementation Science e-Hub and delivered between 2020 and 2022.

**Methods:**

Our program evaluation focused on the feasibility, acceptability and effectiveness of the virtual ISS. We used data from program documents and evaluation surveys completed by trainees at the end of each ISS. The e-Hub data analytics were also collected over the duration of the ISS.

**Findings:**

One hundred and thirty-five participants from 28 countries in five WHO regions attended the three ISS between 2020 and 2022. The virtual delivery was well-received and found to be efficient in program delivery, networking, and for providing collaborative opportunities for trainees from many different countries. The recently developed GACD Implementation Science e-Hub has recorded 20,000+ users since its launch in late 2020 and was found to be an instrumental platform to support the program by providing a stand-alone, comprehensive online learning space for knowledge and skill development in implementation research.

**Implications for D&I Research:**

The delivery of the virtual GACD ISS has proved to be feasible, acceptable and effective and offers greater scalability and sustainability as part of a future strategy for capacity strengthening in implementation research globally.

**Primary Funding Source:** Global Alliance for Chronic Diseases

### S23 A scoping review of potential social marketing applications for implementation science

#### Heather Colquhoun^1^, Moriah Ellen^2^, Jamie Brehaut^3^, Nedra Kline Weinreich^4^, Coby Morvinski^5^, Sareh Zarshenas^6^, Tram Nguyen^6^, Justin Presseau^7^, Nicola McCleary^7^, Enola Proctor^8^

##### ^1^University of Toronto, Toronto, ON, Canada; ^2^Ben-Gurion University of the Negev, Be'er Sheva, Israel; ^3^Ottawa Hospital Research Institute, Ottawa, ON, Canada; ^4^Jerusalem, Israel; ^5^Tel Aviv, Israel; ^6^Toronto, Canada; ^7^University of Ottawa, Ottawa, ON, Canada; ^8^George Warren Brown School of Social Work, Washington University in St. Louis, St. Louis, MO, USA

###### **Correspondence:** Heather Colquhoun (heather.colquhoun@utoronto.ca)

*Implementation Science 2024*, **19(2):**S23

**Background:** While dissemination and implementation (D&I) has a history of borrowing from other fields to advance its science, understanding how approaches from marketing might enhance D&I methodology remains a yet untapped area of theoretical and methodological potential. Social marketing (i.e., applying commercial marketing to solve social or health problems) is a branch of marketing that shares many conceptual features to D&I (e.g., behaviour change) but remains an unrealized opportunity for synergy. This review aimed to 1) describe studies that have tested social marketing interventions in controlled designs; 2) describe the interventions and their context, mechanism, and outcomes; and 3) propose ‘best bet’ social marketing approaches to apply into D&I.

**Methods:** This scoping review followed Joanna Briggs Institute Methodological Guidance, and included a team of D&I and social marketing scientists. Nine databases were searched. Studies were included that 1) utilized a randomized or non-randomized controlled intervention design, and 2) tested a social marketing intervention as defined by five essential social marketing criteria. Two reviewers independently performed all screening and extraction. Variables extracted included intervention details using social marketing criteria and the intervention’s context, mechanism, and outcomes. Team consensus discussions of the review results were used to determine ‘best bet’ strategies to borrow.

**Findings:** Screening of 4,866 citations yielded 27 included studies published from 1999-2022. Topics were all in health and included booster seat use, sanitation, construction safety, cancer screening, nutrition, and sexual health. Novel theories identified included ‘Exchange Theory’ and ‘Consumer Information Processing Model’. All studies outlined clear intervention components for product, price, place, and promotion (the 4 Ps of social marketing). Proposed strategies to borrow are being finalized but include: better understanding of trade-offs, considering that a social offering is necessary, considering the behaviour to be changed as a ‘product’, and recognizing the ‘price’ or what one needs to give up to change their behaviour.

**Implications for D&I Research:** This review will be the first to examine the potential for using social marketing theories and approaches for application to D&I science, and could invigorate novel and creative thinking around a previously untapped and potentially beneficial field.

### S24 Using routinely-collected health data to understand how automaticity contributes to evidence-practice gaps: A scoping review

#### Nicola McCleary^1,2^, Niyati Mistry^1^, Mona Maier^3^, Justin Presseau^1,2^, Sebastian Potthoff^4^, Heather Colquhoun^5^, Jamie Brehaut^1,2^

##### ^1^Ottawa Hospital Research Institute, Ottawa, ON, Canada; ^2^University of Ottawa, Ottawa, ON, Canada; ^3^University of Aberdeen, Aberdeen, United Kingdom; ^4^Northumbria University, Newcastle, United Kingdom; ^5^University of Toronto, Toronto, ON, Canada

###### **Correspondence:** Nicola McCleary (nmccleary@ohri.ca)

*Implementation Science 2024*, **19(2):**S24

**Background:** Behaviour is often guided by thought processes that occur automatically (i.e. system 1/fast thinking/automaticity). Understanding how automaticity contributes to evidence-practice gaps and developing automaticity-informed D&I interventions has been identified as an area needing growth in D&I science. Given the increasing availability of routinely-collected health data, studies are emerging which use these data to investigate impacts of automaticity on health care. We conducted a scoping review to chart these studies and identify ways that routine data could enhance D&I intervention development through better understanding how automaticity impacts care.

**Methods:** We searched MEDLINE, EMBASE, CINAHL, and PsycINFO for studies using routine data to investigate automaticity in healthcare provider behaviour. Two reviewers independently screened records and extracted data on clinical settings, provider and patient groups, clinical actions studied, data sources, automatic processes investigated, and findings. Data were narratively summarized.

**Findings:** Of 17,696 identified records, we included 59 studies (published 1990-2022). Seventy-eight percent took place in the USA, 75% in hospitals, and 75% focused on physicians. Clinical actions spanned illness prevention, diagnosis, treatment provision, and general health management, across various patient populations. Data sources included patient records, insurance databases, and population-based health databases. Fifteen studies explored specific manifestations of automaticity (e.g. the availability heuristic – relying on what easily comes to mind when making likelihood judgments – whereby physicians reduced their rate of prescribing an appropriate medication after a patient had a rare adverse response). Thirteen identified conditions that predispose to automaticity (e.g. greater reliance on automaticity as cognitive resources deplete over time (decision fatigue) – identifying lower rates of ordering guideline-recommended screening as the workday progressed). Eleven developed automaticity-informed D&I interventions (e.g. using audit data to modify default selections within electronic order sets). Other approaches were less frequent (e.g. investigating the accuracy of automaticity - assessing intuition and comparing to subsequent patient outcomes).

**Implications for D&I Research:** We identified a range of ways in which our understanding of automaticity can be enhanced using routine data. This is an under-utilized approach in D&I science that can support better understanding of how automaticity impacts care in real-world environments, thereby supporting the development of automaticity-informed D&I interventions.

**Primary Funding Source:** Canadian Institutes of Health Research

### S25 Rethinking our future: Describing and enhancing the impacts of dissemination and implementation science

#### Ross Brownson^1^, Douglas Luke^2^, Karen Emmons^3^, Vianca Cuevas Soulette^4^

##### ^1^Prevention Research Center, Brown School, Washington University in St. Louis, St. Louis, MO, USA; ^2^Center for Public Health Systems Science, Washington University in St. Louis, Saint Louis, MO, USA; ^3^Harvard University, Boston, MA, USA; ^4^Center for Obesity Prevention and Policy Research, Washington University in St. Louis, St. Louis, MO, USA

###### **Correspondence:** Ross Brownson (rbrownson@wustl.edu)

*Implementation Science 2024*, **19(2):**S25

**Background:** Researchers, including those in dissemination and implementation (D&I) science, do an excellent job tracking scientific impacts of their scholarship in ways relevant for academia (e.g., publications, grants)—metrics that have limited utility in demonstrating broader, real-world impacts on population health and equity. The National Cancer Institute’s Implementation Science Centers in Cancer Control (ISC3) includes 7 P50 Centers. ISC3 advances the rapid development and testing of innovative approaches to increase the D&I of evidence-based cancer control interventions. We provide an overview of the approach underway within the ISC3 network to identify and track health and social impacts which are highly relevant to practice, policy, the economy, and society at large.

**Methods:** ISC3 adapted and applied the Translational Science Benefits Model (TSBM) to identify the impact on the discipline of D&I science, and to consider D&I impacts in the other four TSBM domains: 1) clinical and medical benefits; 2) community and public health benefits; 3) economic benefits; and 4) policy and legislative benefits. To systematically collect data from all Centers, we: 1) developed a set of detailed impact indicators with examples; 2) created a common data collection template; 3) gathered and summarized the impact data from each center; and 4) identified next steps and support needs.

**Findings:** Based on data from 48 ISC3 pilot studies, cores or network activities, we identified 84 distinct benefits. The most common impacts were shown for implementation science (43%), community/public health (28%), clinical (18%), policy (8%), and economic (2%). Frequent audiences included primary care providers, public health practitioners, community partners, and policy makers. Needs identified by ISC3 members to accelerate benefit impacts included product feedback, assistance in storytelling, case study development, and support for early career investigators.

**Implications for D&I Research:** The ISC3 network is using a participatory approach to successfully apply the TSBM—thus seeking to maximize the real-world impacts of D&I science. We will provide tools for impact data collection and creative ways of telling compelling stories. The D&I field needs to prioritize ways to more fully document and enhance impacts on clinical and public health practice, policy and systems change, and health equity.

**Primary Funding Source:** National Institutes of Health

### S26 Walk the talk – a toolkit that builds organizational capacity, shifts power and provides an alternative to top-down approaches

#### Eleni Sofouli

##### Douglas Hospital Research Centre, Montreal, Canada

###### **Correspondence:** Eleni Sofouli (eleni.sofouli@mail.mcgill.ca)

*Implementation Science 2024*, **19(2):**S26

**Background:** The prevailing way of implementing innovations is for organisations to be told what needs to be implemented and how to do it. Our project employed an alternative approach which built capacity in community-based organisations in implementation science and shifted power. At the centre of the novel implementation strategy – Walk the Talk toolkit - is an implementation team made-up of service users, family members, knowledge users, service providers and managers. Teams follow a facilitated planning process and receive coaching in order to implement a recovery-oriented innovation of their choice that aligns with the Guidelines for Recovery-Oriented Practice.

**Methods:** The implementation strategy was designed and tested in seven organisations providing housing and mental health services across Canada. 55 implementation team members (12 managers, 19 service providers, 16 service users, 3 family members and 5 knowledge users) participated in eight 60-90 minute group interviews. Questions were open-ended and focused on the experience of the implementation strategy. Subsequent funding enabled the development of the online bilingual toolkit in partnership with an expert panel of facilitators and implementation team members, and a social enterprise specializing in website and digital content development.

**Findings:**

All seven organisations completed the process and implemented one recovery-oriented innovation based on the guidelines. Findings of the qualitative study were published in *Evaluation and Program Planning* in 2022. Implementation team members, none of whom were previously trained in implementation science, learned how to apply implementation science frameworks and tools (such as the CFIR) to their planning process. They reflected on how the process stood in contrast to usual approaches to change that they were accustomed to. For instance, rather than go fast, they could take time, rather than falling flat, they could follow through, and rather than top-down, it was bottom-up. The online Walk the Talk toolkit was launched in February 2022.

**Implications for D&I Research:**

For implementation science to achieve its aims, non-scientists need to be able to apply it. This involves both translating the knowledge of implementation science into practical resources *and* building capacity in organisations. Walk the Talk toolkit addresses both and can inform future approaches to implementation in and beyond mental health recovery.

**Primary Funding Source:** Canadian Institutes of Health Research, McGill University, Healthy Brains, Healthy Lives

### S27 Pathfinding, peace-making, power, and passion: Exploring the lived experience of facilitation during implementation of Canada's mental health recovery guidelines

#### Marie-Pier Rivest

##### University of Moncton, Moncton, NB, Canada

###### **Correspondence:** Marie-Pier Rivest (marie-pier.rivest@umoncton.ca)

*Implementation Science 2024*, **19(2):**S27

**Background:** We build on a 6 year project to implement Canada's mental health recovery guidelines using the co-produced Walk the Talk Toolkit. (https://walkthetalktoolkit.ca). Facilitation is explored from multiple stakeholder perspectives' to embed lived experience within the toolkit, enhancing appeal and inclusivity.

**Methods:** This study explored facilitation as an active and on-going process propelling the work of implementation teams to deliver recovery-oriented interventions. Forty semi-structured interviews with those who use and deliver services, alongside facilitators across 7 organizations were conducted between May 2022 and May 2023. Interviews explored how facilitation can be improved from each stakeholder's perspective, during planning, implementation, and on-going coaching. Thematic analysis reveals what is important during each phase, and how this can be used to enhance the experience and outcomes of future implementation efforts.

**Findings:** Four themes emerged: people, process, pitfalls and payoff. People refers to stakeholders involved, focusing on service user needs and role of facilitator. Process refers to facilitation stages. Pitfalls alerts to potential challenges (unmet needs) whilst payoff captures the outcomes of successful facilitation eg. evidence-based equity driven services and empowered stakeholders. Implementation work requires more groundwork for both stakeholders and facilitators. Effective facilitators are passionate about recovery, providing a catalyst to open dialogue and establish a shared vision around implementation. They play various pathfinding roles when navigating each stage of facilitation. Facilitators must embrace conflict and anticipate advocating when establishing parity and flattening entrenched hierarchies. On-going coaching maintains momentum and motivation, increasing successful implementation outcomes. Capacity building relating to recovery and implementation science requires facilitators to nurture relationships at all levels. Facilitator gender is less important than cultural competence.

**Implications for D&I Research:** Co-producing implementation toolkits need meaningful engagement from all stakeholders at all stages. Facilitation is a key component to successfully engage implementation teams and generate ownership and thus ensure successful implementation outcomes.

**Primary Funding Source:** Canadian Institutes of Health Research

### S28 Adapting walk the talk toolkit for equity deserving groups: Building capacity in community organizations

#### Myra Piat

##### The Montreal West Island Integrated University Health and Social Services Center (Douglas Mental Health University Institute), Montreal, QC, Canada

###### **Correspondence:** Myra Piat (myra.piat@douglas.mcgill.ca)

*Implementation Science 2024*, **19(2):**S28

**Background:** The aim of this study is to adapt *Walk the Talk Toolkit* for equity deserving groups. *Walk the Talk Toolkit* is a pragmatic implementation science-based approach to promoting the uptake and sustainability of integrated care and a bridge between psychiatric and community-based services. This study is about adapting an implementation strategy - our Toolkit and not about adapting an intervention.

**Methods:** In this 5-year project we will: 1) identify suggested adaptations through process of co-production; 2) determine the core components of the Toolkit; 3) create an Adaptation Guide; 4) implement the Toolkit and Adaptation Guide in community organizations; 5) evaluate the impact of implementing the Adapted version of the Toolkit and the new intervention and; 6) translate lessons learned into *Walk the* Talk Toolkit V2.0. Seven community organisations in Canada and Europe will participate. Each organization serves intersectional equity-deserving populations including: at-risk, socially isolated women and trans people, the 2SLGBTQI+communities, Indigenous persons, and those from cultural and linguistic minority groups in both urban and rural sites. Building capacity in the 7 organizations is a priority. None of the organizations have ever participated in an implementation project.

**Findings:** In 2023 we will conduct 10 Adaptation Team meetings where stakeholders will make suggestions on adapting the toolkit to their context. Based on these findings we will develop an Adaptation Guide. Sites will implement the toolkit using the Adaptation Guide. We will measure the impact on those using the toolkit and Adaptation Guide.

**Implications for D&I Research:** We are among the first to propose studying the adaptation of an implementation strategy (our Toolkit) for equity deserving groups. This research will make important contributions to the field of implementation science and adaptation. Community organizations will build capacity as they learn how to co-produce an Adaptation Guide and actually implement the Toolkit. This knowledge may be used in the future to implement other interventions into their organizations. Other equity deserving groups will have free access to use the process and materials developed to adapt new health interventions, thus making this work pertinent for a wide audience internationally.

**Primary Funding Source:** Canadian Institutes of Health Research

### S29 Reconsidering the phases from discovery to population impact: A case study of hospital at home

#### Albert Siu^1^, Bruce Leff^2^

##### ^1^Icahn School of Medicine at Mount Sinai, New York, NY, USA; ^2^Division of Geriatric Medicine and Gerontology, Johns Hopkins University School of Medicine, Baltimore, MD, USA

###### **Correspondence:** Albert Siu (albert.siu@mssm.edu)

*Implementation Science 2024*, **19(2):**S29

**Background:** The phases of translational research are described as starting from initial discovery to clinical trials and then afterwards to dissemination and implementation. We propose that implementation phases should instead be overlapped with translational research phases from the start. We apply these combined phases to a case study of hospital at home (HaH) implementation.

**Methods:** We reviewed descriptions of translational research phases and combined them with implementation phases previously elaborated. We retrospectively assigned studies of HaH to corresponding phases. We then applied the Consolidated Framework for Implementation Research (CFIR) to efforts to implement HaH from 3 different phases (early effectiveness study, a CMS project to scale HaH, and sustainability during and after a pandemic regulatory waiver),

**Findings:**

20+ years of HaH-related research dating to T0 discovery phases were assigned to all phases in the Table. The sequence of studies was often not temporally sequential. Applying CFIR to 3 efforts to implement HaH during different phases of implementation, we found that the content and importance of different constructs (e.g., inner vs. outer setting) and the choice of implementation strategies differed depending on implementation context (testing of effectiveness, scaling, or sustainability). Early effectiveness studies mostly examined implementation issues in intervention, inner setting, and individuals domains. However, formal, explicit and early consideration of many CFIR constructs wasn’t done, particularly issues affecting scale and sustainment. For example, HaH program intake is primarily through hospital emergency departments (ED). Initial efforts would have benefited from incorporating strategies (e.g., incorporating ED leadership into program leadership) to address night and weekend admissions, as well as ED overcrowding. Many regulatory barriers did not surface during initial considerations.

**Implications for D&I Research:** Considering implementation issues late may contribute to delay in bringing discoveries to population impact. Our case study suggests that scale and sustainability bear earlier consideration because barriers and facilitators to implementation are likely to be different in content and importance at different phases of implementation.

**Primary Funding Source:** The John A. Hartford Foundation

**Table 1 (abstract S29).** See text for description

**Phases of Translation**				
**T0:** Discovery of approach	**T1:** Intervention development	**T2:** Efficacy/ effectiveness studies	**T3:** Effectiveness research	**T4:** Evaluation of population impact
	**D1:** Pre-implementation exploration/ preparation	**D2:** Implementation	**D3:** Scaling	**D4:** Research on factors affecting sustainability
**Phases of implementation**				

### S30 Understanding sustainment challenges and opportunities from a longitudinal analysis of evidence-informed practices implemented in the Veterans Health Administration

#### Andrea Nevedal^1^, Caitlin Reardon^1^, Laura Damschroder^1^, Marilla Opra Widerquist^2^, Maria Arasim^2^, George Jackson^3^, Brandolyn White^4^, Madison Burns^4^, Jennifer Lindquist^4^, Gemmae Fix^5^, Kathryn DeLaughter^6^, Sarah Cutrona^7^, Allen Gifford^8^, Guneet Jasuja^9^, Timothy Hogan^9^, Heather King^10^, Blake Henderson^11^

##### ^1^Veterans Health Administration, VA Ann Arbor Healthcare System, Ann Arbor, MI, USA; ^2^Veterans Health Administration, Center for Clinical Management Research, Ann Arbor, USA; ^3^Veterans Health Administration, Center of Innovation to Accelerate Discovery and Practice Transformation (ADAPT), VA Durham Healthcare System, Dallas, TX, USA; ^4^Durham VA Health Care System, Durham, NC, USA; ^5^Veterans Health Administration, Boston, MA, USA; ^6^University of Massachusetts Medical School, Worcester, MA, USA; ^7^University of Massachusetts Chan Medical School, Worcester, MA, USA; ^8^Boston University Chobanian & Avedisian School of Medicine, Boston, MA, USA; ^9^Veterans Health Administration, VA HSR&D Center for Healthcare Organization and Implementation Research, Bedford, MA, USA; ^10^Duke University, Durham, NC, USA; ^11^VHA Innovation Ecosystem, Washington, DC, USA

###### **Correspondence:** Andrea Nevedal (andrea.nevedal@va.gov)

*Implementation Science 2024*, **19(2):**S30

**Background:** The Veterans Health Administration (VHA) is the United States largest integrated healthcare system. The VHA’s Diffusion of Excellence (DoE) seeks to identify, spread, and sustain evidence-informed practices. Studying sustainment is crucial to the field of implementation science; over half of innovations fail to be sustained over time. Our objective was to characterize the longitudinal pathways of DoE practices as they transition from initial implementation to long-term sustainment.

**Methods:** From 2016-2021, we conducted a mixed-methods evaluation with post-implementation interviews and three annual sustainment surveys to 82 implementation leaders (hereafter: “leads”) of 57 DoE practices. Primary outcomes included (implementation, sustainment), and secondary outcomes (institutionalization, effectiveness, anticipated sustainment). We performed descriptive statistics and directed content analysis using Hailemariam et al.’s factors influencing sustainment.

**Findings:** Approximately five years post-implementation, we identified three sustainment pathways, each representing about one-third of leads. First, one-third of leads reported that their practice was fully sustained. Second, one-third of leads reported that their practice was not fully sustained, which included practices that were in a liminal stage (i.e., neither sustained nor discontinued) or permanently discontinued. Third, one-third of leads had missing outcomes at the final assessment in 2021. Over time, a higher percentage of leads (41%) reported inconsistent primary outcome pathways (ie., earlier outcomes differed from recent outcomes) compared to those who reported consistent primary outcomes (28%). Thirty-four percent of leads with fully sustained practices reported overcoming contextual barriers (e.g., inadequate *workforce*). Leads for fully sustained practices were more likely to report positive secondary outcomes compared to those who did not sustain their practice. Key contextual barriers to practice sustainment included inadequate *workforce*, *unable to maintain practice fidelity/integrity*, *critical incidents* related to the COVID-19 pandemic, o*rganizational leadership did not support sustainment of the practice, and no ongoing support* from constituents. Key facilitators to practice sustainment included demonstrating practice *effectiveness/benefit*, *organizational leadership support*, adequate *workforce*, and *adaptation/alignment* with local context.

**Implications for D&I Research:** We identified diverse pathways from initial implementation to longer-term sustainment; initial implementation outcomes did not always predict long-term sustainment. Findings illuminate DoE’s impact, including return on investment, achieving learning health system goals, and insights into achieving high-quality health care in VHA.

**Primary Funding Source:** Department of Veterans Affairs

### S31 Are they all just bridging factors? distinguishing between epis constructs within the context of co-located cross sectoral services for interpersonal violence

#### Tonya Van Deinse^1^, Cynthia Fraga Rizo^1^, Adrianna Carter^1^, Lauren Ericksen^1^, Julia Metz^1^, Christine Murray^2^, Alicia Bunger^3^

##### ^1^University of North Carolina at Chapel Hill, Chapel Hill, NC, USA; ^2^University of North Carolina at Greensboro, Greensboro, NC, USA; ^3^The Ohio State University, Columbus, OH, USA

###### **Correspondence:** Tonya Van Deinse (tbv@email.unc.edu)

*Implementation Science 2024*, **19(2):**S31

**Background**: Family justice centers (FJCs) are complex, cross-sectoral service models aimed to improve safety and well-being of people experiencing intimate partner and sexual violence (IPSV). FJCs co-locate multiple agencies to foster collaboration and streamline service delivery (e.g., legal, medical, mental health). However, co-located models are susceptible to multilevel implementation determinants that impact exploration, planning, implementation, and sustainment. This study addresses a critical question in the emerging area of implementation science and IPSV service delivery: What factors influence exploration and implementation of co-located IPSV service models?

**Methods**: The research team interviewed 59 representatives from 8 co-located centers. The research team used the Exploration, Preparation, Implementation, and Sustainment (EPIS) framework to inform development of the interview guide and tailor it to centers’ implementation phase. The data were inductively coded to identify implementation determinants and then deductively coded based on the EPIS constructs: outer context, inner context, bridging factors, and innovation factors.

**Findings**: During exploration/preparation, the outer context and bridging factors emerged as most prominent. Participants identified high lethality incidents, an increase in partner homicides, and new funding streams as precipitating factors to exploring the feasibility of a FJC. During the preparation phase, bridging factors, including funding and processes that enhanced partner collaboration, were most relevant. During implementation/sustainment, the distinction between constructs was less clear. Specifically, moving from preparation to implementation requires entities from the outer context to create an inner context (e.g., FJC) yet maintain organizational affiliation and influence. However, the FJC model itself is, by design, collaborative and inter-organizational, making it difficult to distinguish between bridging factors, inner context, and factors related to the innovation.

**Implications for D&I Research:** Findings from this study have implications for developing and specifying implementation strategies, particularly related to a strategy’s timing and targets. For FJCs and other cross-sectoral approaches, implementation strategies may need to target partnerships and other factors in the outer setting during the exploration and preparation phases and then focus more on the inner context (e.g., leadership, communication, adaptation) during implementation/sustainment. Lastly, there may be other factors not included in existing frameworks that may help understand the implementation context of FJCs and other co-located and complex service models.

**Primary Funding Source:** National Institute of Justice

### S32 Engagement science in D&I research at the National Institutes of Health: Opportunities to improve synergy among the fields

#### Aubrey Villalobos^1^, Dr. Manami Bhattacharya^1^, Antoinette Percy-Laurry^2^, Dara Blachman-Demner^3^

##### ^1^National Institutes of Health, National Cancer Institute, Rockville, MD, USA; ^2^National Institutes of Health, National Institute on Minority Health and Health Disparities, Bethesda, MD, USA; ^3^National Institutes of Health, Office of Behavioral and Social Science Research, Bethesda, MD, USA

###### **Correspondence:** Aubrey Villalobos (aubrey.villalobos@nih.gov)

*Implementation Science 2024*, **19(2):**S32

**Link:** https://implementationsciencecomms.biomedcentral.com/articles/10.1186/s43058-023-00462-y

### S33 Integrating intervention optimization and implementation science to optimize multicomponent implementation strategies

#### Kate Guastaferro, Jillian Strayhorn

##### New York University, New York, NY, USA

###### **Correspondence:** Kate Guastaferro (kate.guastaferro@nyu.edu)

*Implementation Science 2024*, **19(2):**S33

**Background:** When intervention scientists optimize interventions using the multiphase optimization strategy (MOST), they start by identifying promising components that are candidates for inclusion in the optimized intervention. They then use the empirical results of a rigorous optimization randomized control trial (ORCT) to determine which candidate components merit inclusion because their individual and/or combined effects justify their resource requirements (e.g., money, interventionist time, etc.). To date, applications of MOST have generally focused on optimizing intervention content. However, in some cases, the key empirical question driving optimization may not be which content components are worth including, but instead which implementation strategies (individually and in combination) are worth including to support the successful dissemination and implementation of an already-effective intervention. There is also great potential in the use of MOST in these cases—i.e., to optimize a multicomponent package (i.e., wrap-around intervention) comprised of implementation strategies.

**Methods:** Using hypothetical scenarios, this talk will outline a contrast between the optimization of intervention content and the optimization of implementation strategies. We begin by establishing a common language through the development of a conceptual model for a wrap-around intervention. Then, we will demonstrate how to design an ORCT that empirically informs the selection of an optimized package of implementation strategies.

**Findings:** Despite their different purposes, there are fundamental similarities in these two applications of MOST (i.e., for the optimization of intervention content and for the optimization of implementation strategies). We highlight considerations specific to the construction of an optimized multicomponent implementation strategy for an existing evidence-based intervention.

**Implications for D&I Research:** Like components of intervention content, implementation strategies incur costs (in money, time, etc.). Integrating intervention optimization and implementation science for the optimization of multicomponent implementation strategies that successfully support implementation outcomes, while themselves remaining affordable, scalable, and efficient, is an exciting new frontier.

### S34 Applying the multiphase optimization strategy to optimize for health equity: New methods and future directions in the incorporation of implementation science.

#### Jillian Strayhorn^1^, Linda Collins^2^, David Vanness^3^

##### ^1^New York University, New York, NY, USA; ^2^New York University School of Global Public Health, New York, NY, USA; ^3^Pennsylvania State University, University Park, PA, USA

###### **Correspondence:** Jillian Strayhorn (jcs9972@nyu.edu)

*Implementation Science 2024*, **19(2):**S34

**Background:** Intervention optimization using the multiphase optimization strategy (MOST) is an empirical process that involves using data from an optimization randomized control trial (ORCT) to strategically balance the criteria that are deemed important for the eventual implementation of the intervention, including effectiveness, affordability, scalability, and efficiency criteria. In general, the goal is to identify the intervention, composed of some combination of candidate intervention components or component levels, that is not only effective but also more likely to be readily implementable (e.g., because the intervention is affordable and efficient in the use of available resources).

Recently, we have proposed intervention equitability as a key additional criterion in intervention optimization, with equitability defined as the extent to which the health benefits provided by an intervention are distributed evenly versus concentrated among those who are already advantaged. Since it is plausible that the various alternative versions of a multicomponent intervention will differ in their equitability, choosing one optimized version of the intervention may mean that investigators have to confront challenging tradeoffs among overall effectiveness, equitability, and implementability criteria.

**Methods:** Using a hypothetical case study and simulated ORCT data, we show how MOST can be used to balance tradeoffs in a principled fashion, with transparency, in the selection of an optimized intervention.

**Findings:** Importantly, different interventions emerge as optimal when equitability is considered versus when it is not. The selection of an optimized intervention differs further depending on how strongly decision-makers (e.g., the intervention scientists optimizing the intervention) care about equitability versus overall effectiveness and implementability criteria—and depending on how resource-rich the intended context for implementation is anticipated be, because tradeoffs between equitability and other criteria tend to be more severe when fewer resources are available for implementation.

**Implications for D&I Research:** This new work opens possibilities for approaching intervention equitability as a criterion to be strategically balanced with effectiveness and implementability, ideally such that the interventions that are eventually implemented help to ameliorate (and at least, do not worsen) health disparities. When approached in the optimization of implementation strategies, there is also exciting potential to consider equitability in implementation outcomes.

**Primary Funding Source:** National Institutes of Health

## Clinical Care Settings: Patient-level Interventions

### S35 Disability claims as a pathway to pain treatment in veterans: Screening, brief intervention and referral to treatment for pain management (SBIRT-PM) analyzed using a relational coordination framework

#### Marc Rosen^1^, Steve Martino^2^

##### ^1^Yale University, West Haven, CT, USA; ^2^Veterans Health Administration, VA Connecticut Healthcare System, West Haven, CT, USA

###### **Correspondence:** Steve Martino (steve.martino@yale.edu)

*Implementation Science 2024*, **19(2):**S35

**Background:**

Veterans with chronic pain from musculoskeletal disorders (MSD) from military service often file claims which are evaluated by Compensation & Pension (C&P) examinations. A hybrid type I effectiveness implementation multi-site randomized clinical trial is testing the effectiveness of telephone-based SBIRT-PM across eight VA medical centers. Lists of veterans with recent MSD claims are used to identify and contact veterans for study participation.

**Methods:**

Participation in SBIRT-PM by veterans in the clinical trial was reviewed. Pre- and post-trial, staff from the medical centers completed a relational coordination survey that examined the communication and relationships among different workgroups (primary care, administration, pain management, and C&P teams) vested in veteran access to pain care. A subset of those staff also participated in a semi structured interview about pain treatment referral practices within their medical centers.

**Findings:**

To date, >70% of veterans assigned to SBIRT-PM participated in at least one session, and more than 50% of those participated in a second session. Pre-trial, the C&P group’s relational coordination composite scores were lower than any other workgroup’s. VA staff reported that there was little communication between the C&P team and other clinical and administrative teams. During the trial, the VA transitioned from VA-based C&P exams to mostly private contracted C&P exams, further separating the C&P and treatment workgroups. Post-trial findings and pre-post changes will be presented, including implications for future efforts to implement SBIRT-PM in VA.

**Implications for D&I Research:**

Most study participants, identified by having filed a C&P claim for an MSD, were receptive to SBIRT-PM counseling. C&P can be an important point of contact with veterans who might benefit from VA-supported pain care, but information flow between the clinical and disability adjudication systems has to be established. Facilitating successful implementation of interventions like SBIRT-PM depends on the underlying quality of relationships and communication among those involved in the VA pain care pathway.

**Primary Funding Source:** National Institutes of Health

### S36 Insights from a multi-level, process evaluation of LAMP (Learning to Apply Mindfulness to Pain), a type 1 hybrid effectiveness/implementation pragmatic clinical trial

#### Diana Burgess

##### Veterans Health Administration, Minneapolis VA Health Care System, Minneapolis, MN, USA

###### **Correspondence:** Diana Burgess (diana.burgess@va.gov)

*Implementation Science 2024*, **19(2):**S36

**Background:**

The Learning to Apply Mindfulness to Pain (LAMP), Type 1 hybrid effectiveness/implementation pragmatic clinical trial examined the effectiveness of two scalable approaches to delivering mindfulness-based interventions for improving Veterans’ chronic pain and mental health comorbidities, compared to Usual Care, and identified barriers and facilitators to implementing these interventions, within the VA system.

**Methods:**

We conducted a multi-level mixed-methods process evaluation, guided by the Reach, Effectiveness, Adoption, Implementation, and Maintenance (RE-AIM) framework. This included semi-structured interviews with VA healthcare system leaders and staff, focus groups with study interventionists and VA patients, mixed-methods surveys of VA participants in the two intervention arms, and quantitative data to assess intervention application, adherence, fidelity and inform cost estimate. We also obtained ongoing feedback from our stakeholder and patient engagement panels.

**Findings:**

Ongoing conversations with stakeholders shaped our plan for how LAMP should be modified to be delivered by VA Whole Health (WH) coaches and be better integrated with the VA WH initiative. Semi-structured interviews identified key facilitators (e.g., alignment of LAMP with organizational goals; support for telehealth delivery) and barriers, many of which were expressed by front-line staff and managers (e.g., lack of awareness about WH coaches; lack of enthusiasm and resistance to new programs, due in part to overload and burnout). Patient engagement panel feedback revealed a lack of awareness about VA WH, but enthusiasm from patients who had used WH and interest in those who had not, and additional recommendations for optimizing these programs. Mixed method surveys revealed successful implementation of the LAMP programs as illustrated by high degrees of satisfaction and engagement, and the ability to address participants’ capability, resource, and motivational needs. Specific aspects of program delivery format that should be retained or optimized for future implementation were also identified.

**Implications for D&I Research:**

Our multi-level process evaluation yielded key insights into future implementation of our interventions but also revealed the need to broaden the types of stakeholders that are included, particularly front-line staff, and managers and others who may be resistant to such programs, to develop robust implementation strategies that address a broad array of barriers.

**Primary Funding Source:** Department of Veterans Affairs

### S37 Tailored to fit: How an implementation framework can support adaptation of a pragmatic trial of a whole health approach to pain care for veterans in diverse VA clinical settings

#### Karen Seal^1^, William Becker^2^, Natalie Purcell^3^

##### ^1^San Francisco VA Health Care System, San Francisco, USA; ^2^Yale School of Medicine, New Haven, CT, USA; ^3^San Francisco, CA, USA

###### **Correspondence:** Karen Seal (Karen.Seal@va.gov)

*Implementation Science 2024*, **19(2):**S37

**Link:** https://journals.lww.com/lww-medicalcare/abstract/2020/09001/tailored_to_fit__how_an_implementation_framework.5.aspx

### S38 Implementing pharmacist-led chronic care management in rural primary care

#### Geoffrey Curran^1^, Shweta Pathak^2^, Jeremy Thomas^1^, Melanie Livet^3^

##### ^1^University of Arkansas for Medical Sciences, Little Rock, AR, USA; ^2^University of North Carolina at Chapel Hill, Chapel Hill, NC, USA; ^3^University of North Carolina-Chapel Hill, Chapel Hill, NC, USA

###### **Correspondence:** Geoffrey Curran (currangeoffreym@uams.edu)

*Implementation Science 2024*, **19(2):**S38

**Background:** Patients with uncontrolled diabetes living in rural communities experience many challenges, including lack of access to needed medication management services. Telepharmacy is a promising approach for addressing this gap. This study provides insights into the implementation of an evidence-based Comprehensive Medication Management (CMM) service in 7 rural and low resourced primary care clinics in the Southeastern US. The CMM service involved 4 pharmacists meeting telephonically with patients (2-4 times) in their homes to identify and resolve Medication Related Problems (MTPs). Implementation strategies (training, recommended clinic workflows, site-specific implementation roadmaps, local champions, external facilitation, technical assistance, audit & feedback) supporting adoption and sustainment of CMM were developed and iterated with clinic stakeholders.

**Methods:** This exploratory mixed methods hybrid type 2 study used a pre-post design. The primary effectiveness measure was *patient A1C* (pre-post). The primary implementation measure was *fidelity* (defined as appropriate resolution of a MTP). Data sources included surveys (clinic staff and patient experiences), qualitative interviews (implementation barriers/facilitators [pre-implementation], feasibility of and satisfaction with implementation strategies [post-implementation]), administrative data (eligible patients contacted and served), and medical records (MTPs, A1C change) collected before, during, and after a 15-month implementation period. The study received human subjects approval.

**Findings:** The service was delivered to 280 unique patients who received 1300+ telephonic pharmacist visits. Fidelity was strong: 85.1% of MTPs identified by pharmacists (1031 0f 1212) were successfully resolved (e.g., provider adjusted medications based upon pharmacist recommendations). Mean patient A1C levels were significantly reduced after receiving the CMM service (adjusted GEE model; 10.55 to 9.0; *p*< .0001). Clinic stakeholders reported significantly higher levels of service acceptability, appropriateness, and intent-to-sustain from baseline to post-implementation (87.5% reported intent to sustain). Service has been maintained and expanded in 6 of 7 clinics. Qualitative results highlighted the importance establishing clinic leader buy-in, adapting implementation strategies to context, establishing positive communication channels between pharmacists and providers, and engaging patients with multiple clinic staff.

**Implications for D&I Research:** Remote and under-resourced clinics can successfully implement and sustain telephonically-delivered evidence-based practices when actively engaged in developing and adapting implementation strategies. The implementation strategies developed here should be evaluated next in controlled roll-out studies.

**Primary Funding Source:** Eshelman Institute for Innovation

### S39 Development of a tailored implementation strategy for an electronic prospective surveillance model for cancer rehabilitation

#### Christian Lopez^1^, Sarah Neil-Sztramko^2^, Kylie Teggart^2^, Kristin Campbell^3^, Tony Reiman^4^, Jonathan Greenland^5^, David Langelier^6^, Mohammed Ahmed^7^, Anita Borhani^8^, Jackie Bender^1^, Gillian Strudwick^9^, Rouhi Fazelzad^10^, Jeffrey Kong^11^, Jennifer Jones^1^

##### ^1^Princess Margaret Cancer Centre, Toronto, ON, Canada; ^2^McMaster University, Hamilton, ON, Canada; ^3^University of British Columbia, Vancouver, Canada; ^4^Saint John Regional Hospital, Saint John, NB, Canada ^5^Memorial University of Newfoundland, St. John's, NF, Canada; ^6^Princess Margaret, Toronto, ON, Canada; ^7^University of Toronto, Toronto, Canada; ^8^University of Toronto, Toronto, ON, Canada; ^9^Centre for Addiction and Mental Health, Toronto, ON, Canada; ^10^University Health Network, Toronto, ON, Canada; ^11^University of British Columbia, Vancouver, BC, Canada

###### **Correspondence:** Christian Lopez (christian.lopez@uhn.ca)

*Implementation Science 2024*, **19(2):**S39

**Background**: An electronic Prospective Surveillance Model (ePSM) uses patient-reported outcomes to monitor symptoms along the cancer pathway for timely identification and treatment. Randomized controlled trials show that ePSMs can effectively manage treatment-related adverse effects. However, an understanding of optimal approaches for implementing these systems into routine care is limited.

**Methods**: Guided by the Knowledge-to-Action cycle, we used a multi-methods approach to identify feasible and important strategies to implement our ePSM, REACH, for four cancer types (breast, colorectal, head and neck, and lymphoma) in four Canadian centres. First, we conducted a scoping review to synthesize the approaches to implementing ePSMs in cancer. Next, qualitative focus groups were conducted at each centre to identify relevant implementation barriers and facilitators. Determinants across both studies were categorized using the Consolidated Framework for Implementation Research (CFIR). These determinants were mapped to the Expert Recommendations for Implementing Change (ERIC) taxonomy using the CFIR-ERIC matching tool. Implementation strategies identified from the ERIC taxonomy were subsequently selected based on their feasibility and importance at each centre.

**Findings**: The scoping review synthesized 46 studies highlighting key determinants and implementation strategies. Twenty-two focus groups were conducted with cancer survivors (n = 13), clinicians (n=44) and management (n=12). Across both studies, adaptability, complexity, compatibility with clinic workflows, and stakeholder engagement were identified has critical for successful implementation. As such, implementation strategies involving key opinion leaders, tailoring to the context, developing processes to monitor outcomes for the purpose of improvement, and technical assistance were chosen.

**Implications for D&I Research:** The implementation of evidence-based practices is highly dependent on local context and mapping barriers and facilitators to specific implementation strategies has been suggested to reduce the impact of barriers. The CFIR and CFIR-ERIC were valuable tools for tailoring the implementation of an ePSM for cancer rehabilitation. This approach may have relevance for adapting other patient-level interventions and developing tailored implementation strategies across diverse contexts to optimize uptake and use. REACH has been implemented at one site, with the launch of subsequent sites planned. An evaluation of implementation success using the implementation outcomes framework is underway.

**Primary Funding Source:** Canadian Institutes of Health Research / Canadian Cancer Society

### S40 Use of quality dashboards to inform implementation of evidence-based preventive care for women veterans

#### Catherine Chanfreau^1,2^, Bevanne Bean-Mayberry^3,4^, Erin Finley^3^, Kimberly Clair^2^, Cody Knight^2^, Rebecca Oberman^2^, Quoc Doan^2^, Rachel Lesser^2^, Tannaz Moin^3,4^, Alison Hamilton^3,4^, Melissa Farmer^3^

##### ^1^Veterans Health Administration, Veterans Affairs Informatics and Computing Infrastructure (VINCI), Salt Lake City, UT, USA; ^2^Veterans Health Administration, VA Greater Los Angeles Healthcare System, Center for the Study of Healthcare Innovation, Implementation & Policy (CSHIIP) Los Angeles, CA, USA; ^3^Veterans Health Administration, HSR&D, Greater Los Angeles Health Care System, CSHIIP, Los Angeles, USA; ^4^UCLA David Geffen School of Medicine, Los Angeles, USA

###### **Correspondence:** Catherine Chanfreau (catherine.chanfreau@va.gov)

*Implementation Science 2024*, **19(2):**S40

**Background:**

Quality dashboards are important tools in improvement and implementation, particularly as part of “audit and feedback” strategies. We developed quality dashboards for an implementation trial comparing Replicating Effective Programs (REP) versus Evidence-Based Quality Improvement (EBQI) to support delivery of three preventive care evidence-based practices (EBPs) for women at 20 Veterans Health Administration (VA) sites. We report here on ongoing dashboard utilization and adaptations requested by the sites.

**Methods:**

We developed a series of dashboards integrating data from VA electronic health records, performance metrics, and program participation. Aggregated-data reports aimed to help sites visualize population health metrics and EBP implementation progress. A patient-level Sharepoint was specifically designed to facilitate recruitment for one EBP. All reports were restricted to site-specific data to reduce potential contamination between sites randomized to EBQI versus REP. Dashboards were demonstrated during pre-implementation calls and sites were asked for a list of potential users. During implementation, dashboards were used to support monthly facilitation calls with EBQI sites.

**Findings:**

To date, dashboards have been deployed at 15 sites (7 EBQI, 8 REP) with registration of 140 site staff. Over 14 months quality dashboards were accessed by 11 staff at 5 EBQI sites (119 views), 11 staff at 4 REP sites (135 views), and 8 implementation team members (738 views). The patient-level Sharepoint was shared with 8 sites (4 REP and 4 EBQI sites; total 93 staff) and accessed at all sites. User number was higher at EBQI sites (22) versus REP (9), but usage volume was similar (9,396 views versus 8,563). Several modifications of the Sharepoint were requested by the EBQI sites to facilitate workflow and improve patient tracking, and those adaptations were deployed at all sites. The decision at one site to offer one EBP to all patients led to an additional tool providing enrollment numbers for both men and women.

**Implications for D&I Research:**

Dashboards facilitating workflow were more widely adopted than those providing an overview of population health and implementation progress. Dashboards benefited from user feedback to improve usability. Communication with sites and responsiveness to their requests may increase the likelihood of utilization sustainment of those tools over time.

**Primary Funding Source:** Department of Veterans Affairs

### S41 Assessing the implementation of two approaches for delivering a pediatric practice-based obesity intervention to support families: Participant perspectives

#### Grace Ryan^1^, Chris Frisard^1^, Sybil Crawford^2^, Susan Druker^1^, Jenny Bram^1^, Alan Geller^3^, Michelle Trivedi^1^, Lori Pbert^1^

##### ^1^University of Massachusetts Chan Medical School, Worcester, MA, USA; ^2^UMass Chan Medical School, Worcester, MA, USA; ^3^Harvard T.H. Chan School of Public Health, Boston, MA, USA

###### **Correspondence:** Grace Ryan (grace.ryan1@umassmed.edu)

*Implementation Science 2024*, **19(2):**S41

**Background:** The American Academy of Pediatrics recommends families of children with overweight and obesity make lifestyle changes to improve body mass index, but provider time and access to treatment are limited. We developed Fitline, a pediatric practice-based program involving a brief provider-delivered intervention (using the Ask-Advise-Refer model) ending with referral to either a mailed family workbook (Fitline-Workbook) or telephonic coaching along with the workbook (Fitline-Coaching) and found similar reductions in BMI z-scores and sustained changes in diet and physical activity behaviors for both interventions. Our goal in this analysis was to assess implementation outcomes of fidelity and acceptability.

**Methods:** We assessed fidelity to the intended protocol and acceptability of the intervention using parent surveys and research staff tracking data. We generated frequencies and descriptive statistics.

**Findings:** 501 families participated (Fitline-Workbook n=258; Fitline-Coaching n=243). We found high fidelity to the “Ask-Advise-Refer” protocol: 81% of parents reported their provider asked about their child’s weight, diet, or level of physical activity, 72% reported their provider advised them on health behaviors, and 99% reported referral to the intervention. The Fitline-Coaching protocol called for 8 coaching calls; 83% of families completed all 8 coaching calls, 8% completed 4-7 calls, and 9% completed 0-3 calls. Acceptability in both conditions was also relatively high; 62% of the Fitline-Workbook condition reported they liked the workbook materials “well” or “very well” compared to 91% of families in the Fitline-Coaching condition; additionally, 90% of Fitline-Coaching families liked the coaching sessions “well” or “very well.”

**Implications for D&I Research:** Overall, we observed high fidelity from providers to the intervention protocol. While acceptability was higher in the Fitline-Coaching intervention compared to the Fitline-Workbook intervention, it was high among both groups, suggesting that the lower-touch and less time-intensive approach to a pediatric weight management intervention may be sufficient for many families. Future research should explore processes for implementing a stepped care approach, wherein families begin in the lower-intensive Fitline-Workbook intervention, then move into the Coaching intervention, if indicated. Establishing effectiveness, as well as fidelity and acceptability was a critical first step, that can help streamline future research exploring a stepped-care model.

**Primary Funding Source:** National Institutes of Health

### S42 A mixed-methods feedback loop to enhance implementation science outcomes: Representative recruitment as a foundation for appropriateness, adoption and sustainability

#### Kathleen Thomas

##### University of North Carolina at Chapel Hill Eshelman School of Pharmacy, Chapel Hill, NC, USA

###### **Correspondence:** Kathleen Thomas (kathleen_thomas@unc.edu)

*Implementation Science 2024*, **19(2):**S42

**Background:**

Implementation/clinical innovation studies rarely provide information on how the distribution of participant characteristics compares to recruitment sites’. This lack of detail raises questions about study representativeness and ability to address the needs of end-users. Lack of representativeness undermines innovation appropriateness which jeopardizes implementation outcomes of adoption and sustainability.

**Methods:**

We gathered a study team with broad expertise, parent advisors and professional champions to design a hybrid comparative effectiveness-implementation study to improve advocacy/care coordination skills among parents of youth with IDD. We planned strategies for data collection to characterize clinic populations pre-recruitment as a benchmark for success. We made plans to compare benchmarks with real-time assessment via team interactions, survey data and linked medical records.

**Findings:**

We used a mixed-methods stakeholder-engaged multifaceted approach to monitor and adjust recruitment protocols to achieve a representative study sample. Stakeholders included parent and professional advisors and clinic personnel. A project website provided public updates and established legitimacy. We extracted electronic medical record data via a data warehouse to describe our target study population. Early benchmarks for success were race and ethnicity. Parent advisors added disability severity. The plan to monitor recruitment with stakeholder input was established a priori. We provided clinicians weekly lists of eligible parents. We used a web-based data collection tool to capture screening and survey data from study participants. This facilitated real-time quality-monitoring of eligible, screened, recruited and retained samples to identify gaps. The study team met weekly to examine recruited samples vis-à-vis benchmarks. The team engaged in problem-solving with parent advisors, professional champions and clinician partners to address recruitment gaps. Strategies included matching family and recruiter characteristics by race, preference for Spanish, and gender of the youth with IDD. If recruited and benchmark proportions were significantly different or persistent, recruitment stopped for all families but those in the low recruitment group.

**Implications for D&I Research:**

Weekly assessment is time-consuming but critical to make small adjustments quickly. Findings provide a set of co-created strategies to monitor, assess and address gaps in recruitment. These strategies to enhance innovation appropriateness to support the adoption and sustainability of clinical innovation studies that address the needs of parents of youth with IDD.

**Primary Funding Source:** Patient-Centered Outcomes Research Institute

### S43 User-centered design to understand the outcomes that matter for youth and families using behavioral health services

#### Genevieve Graaf

##### University of Texas at Arlington, Arlington, TX, USA

###### **Correspondence:** Genevieve Graaf (Genevieve.graaf@uta.edu)

*Implementation Science 2024*, **19(2):**S43

**Background:**

Implementation or clinical innovation study designs are often powered on outcomes pre-specified by funders rather than those most important to patients and families, compromising consumer, provider, or organizational leaders’ perceptions of the innovation’s appropriateness. Thus, evidence generated from outcomes that are not the most important to patients and families undermines uptake and sustainability of study findings. Understanding the priorities and goals of innovation end users (youth and families using behavioral health services, in this case) is a fundamental step in user-centered design.

**Methods:**

To address gaps in evidence regarding the behavioral health outcome priorities of patients and their families, a team of family-led organization leaders and researchers designed a mixed-methods study to document outcomes that matter to youth and their parents. Based on family-led organization leader relationships, the team sought applications for partnership from a diverse set of family-run community organizations that serve youth who use behavioral health services. Each organization had youth-directed and parent-directed programming. Partner organizations had to be ready to collect quantitative data, recruit participants, host and assist in facilitating parent and youth focus groups, and meet with the study team quarterly.

**Findings:**

Six partner organizations were chosen, diverse in geography, race and ethnicity of membership. Partners were fully engaged in planning the timing, process, and content of youth and family focus groups. In round 1, each group identified what they hoped to gain from using behavioral health services for themselves, their families, and their parent or child and discussed what made services a positive experience. Data were coded and summarized through software reports. In round 2, groups reflected on round 1 findings, validated and refined summaries. Groups selected the top three outcomes most important to them. Although the prompts sought behavioral health outcomes, parents and youth emphasized service process measures such as provider communication, collaboration, and showing that they cared, and families feeling respected, validated, and heard.

**Implications for D&I Research:**

Study designs that provide time for stakeholder planning and reflection have the potential to promote network weaving that will support later implementation. These steps also establish foundations from which to co-create innovations that are appropriate and support increased penetration and sustainability.

**Primary Funding Source:** Patient-Centered Outcomes Research Institute

### S44 Using implementation mapping to co-create protocols supporting the implementation of a state policy on screening children for adverse childhood experiences (ACEs) in a system of health centers in inland southern California

#### Monica Perez Jolles

##### University of Colorado, Denver Anschutz Medical Campus, Aurora, CO, Aurora, CO, USA

###### **Correspondence:** Monica Perez Jolles (MONICA.JOLLES@CUANSCHUTZ.EDU)

*Implementation Science 2024*, **19(2):**S44

**Link**: https://www.frontiersin.org/journals/public-health/articles/10.3389/fpubh.2022.876769/full

### S45 User-centered multiple methods approach to inform innovative redesign of care coordination

#### Denise Hynes

##### Oregon State University, Center to Improve Veteran Involvement in Care, VA Portland Healthcare System, Corvallis, OR, USA; Portland, OR, USA

###### **Correspondence:** Denise Hynes (denise.hynes@va.gov)

*Implementation Science 2024*, **19(2):**S45

**Link:** https://www.frontiersin.org/articles/10.3389/frhs.2023.1211577/full

### S46 The implementation of infant pain practice change resource: Facilitators and barriers for implementation in neonatal intensive care units

#### Bonnie Stevens^1,2^, Mariana Bueno^3^, Kate Pearson^3^, Megha Rao^15^, Shirine Riahi^3^, Melanie Barwick^1,2^, Anne Synnes^4^, Carole Estabrooks^5^, Charles Victor^6^, Christine Chambers^7^, Denise Harrison^8,16^, Janet Yamada^13^, Jennifer Stinson^3,6^, Marsha Campbell-Yeo^7^, Melanie Noel^9^, Rachel Flynn^10^, Sharyn Gibbins^14^, Sylvie LeMay^11^, Wanrudee Isaranuwatchai^12^

##### ^1^The Hospital for Sick Children, Toronto, ON, Canada; ^2^University of Toronto, Toronto, Canada; ^3^The Hospital for Sick Children, Toronto, Canada; ^4^ BC Children’s Hospital, University of British Columbia, Vancouver, Canada; ^5^Faculty of Nursing, University of Alberta, Edmonton, AB, Canada; ^6^ University of Toronto, Toronto, Canada; ^7^Dalhousie University, Departments of Psychology & Neuroscience and Pediatrics, IWK Health Centre, Halifax, Canada; ^8^University of Melbourne, Melbourne, Australia; ^9^University of Calgary, Department of Psychology, Calgary, Alberta, Canada; ^10^University College Cork, Cork, Ireland; ^11^Université de Montréal, Montreal, Canada; ^12^St. Michael's Hospital, Toronto, ON, Canada; ^13^Toronto Metropolitan University, Daphne Cockwell School of Nursing, Toronto, ON, Canada; ^14^Trillium Health Partners, Mississauga, ON, Canada; ^15^Mount Sinai Hospital, Toronto, ON, Canada; ^16^University of Ottawa, Ottawa, Canada

###### **Correspondence:** Bonnie Stevens (Bonnie.stevens@sickkids.ca)

*Implementation Science 2024*, **19(2):**S46

**Background:** The *Implementation of Infant Pain Practice Change* (*ImPaC) Resource* is a 7-step, multifaceted, online implementation strategy to improve pain assessment and management in Neonatal Intensive Care Units (NICUs). We aimed to identify implementation facilitators and barriers of the ImPaC Resource across NICUs.

**Methods:** We conducted a hybrid type 1 implementation science study that included an RCT (reported elsewhere) and descriptive component. Canadian NICUs with >15 beds were invited to participate and were randomized to intervention (INT=12) or standard practice (SP=11). INT NICUs recruited a Change Team (CT) who were trained and given access to the Resource for 6 months; SP NICUs were waitlisted for 6 months and then offered access to the Resource. Focus groups (FG) were conducted virtually with all CTs by a trained interviewer following completion of the Resource use. FG questions and data analyses were guided by the Consolidated Framework for Implementation Research (CFIR 1.0). Professionally transcribed qualitative data were analysed using directed content analysis. Valence ratings based on direction (+/-) and strength (–2, –1, 0, +1, +2) were assigned to each CFIR construct/subconstruct. Inductive codes were identified. The most salient construct/subconstruct facilitators (rated as +1 or +2) and barriers (rated as -1 or -2) were described in relation to the frequency of transcripts where they were identified.

**Findings:** We conducted 23 focus groups with 83 CT participants from 23 Canadian NICUs. 1105 discrete codes were identified relating to 31 CFIR constructs/subconstructs. *Innovation characteristics,* i.e., design and quality packaging (21/22), and evidence strength (15/16), were the most salient implementation facilitators. *Inner Setting* facilitators were compatibility with local practices (18/19) and *Process* facilitators related to enabling users to: engage key stakeholders (i.e. clinicians) (13/14) and reflect and evaluate their implementation (12/13). *Inner Setting* barriers included available resources (e.g., lack of time) (15/19), and relative priority (14/14). The COVID-19 pandemic hindered implementation (15/15); this inductive code fits the critical incidents construct introduced in CFIR 2.0.

**Implications for D&I Research:** *Innovation Characteristics* were the most salient implementation facilitators across NICUs. *Inner and Outer Setting* factors hindered the implementation process. Site specific actions may mitigate barriers and their influence on implementation success.

**Primary Funding Source:** Canadian Institutes of Health Research (CIHR)

### S47 Reach and adoption of a family-based treatment for child obesity into primary care practices using the RE-AIM framework

#### Alyssa Button^1^, Amanda Staiano^1^, Alison Baker^2^, Robbie Beyl^1^, Anne-Marie Conn^3^, Angela Lima^4^, Jeanne Lindros^2^, Robert Newton^1^, Rick Stein^4^, R. Robinson Welch^4^, Stephen Cook^3^, Denise Wilfley^4^

##### ^1^Pennington Biomedical Research Center, Baton Rouge, LA, USA; ^2^American Academy of Pediatrics, Itasca, IL, USA; ^3^University of Rochester Medical Center, Rochester, NY, USA; ^4^Washington University in St. Louis, St. Louis, MO, USA

###### **Correspondence:** Alyssa Button (alyssa.button@pbrc.edu)

*Implementation Science 2024*, **19(2):**S47

**Background:** Few children with obesity receive treatment that meets U.S. Preventive Services Taskforce guidelines, and children from disadvantaged and underrepresented backgrounds have lower treatment engagement. The TEAM UP pragmatic trial combines family-based behavioral treatment (FBT) with enhanced standard of care (FBT+eSOC) and compares this approach to eSOC alone. This sub-study describes reach and adoption of both treatment arms, delivered by coaches and primary care providers to underserved populations.

**Methods:** *N*=47 clinics, *n*=43 FBT coaches, *n=*234 eSOC providers, and *n*=730 participating families were included in the evaluation, which uses dimensions of the RE-AIM framework, assessed using a mixed-methods approach. Cross-sectional data were collected using surveys and interviews. Correlations were used to examine associations among both provider attitude (*Evidence-Based Practice Attitude Scale*) and knowledge (*Provider Competence*) with duration of providers’ delivery of FBT or eSOC (days).

**Findings:** The *reach* (i.e., individuals willing to participate) of FBT+eSOC and eSOC spanned to a sample of 48% racial minority (34% Black, 13% Other) and 52% White; 11% Hispanic; and 32% insured by Medicaid.

FBT+eSOC and eSOC were *adopted* (i.e., agents willing to deliver treatment) by providers with positive attitudes toward evidence-based treatments (M=3.8±0.5 on a scale of 0-5) and who had moderate knowledge about pediatric obesity treatment (M=7.2±1.6 on a scale of 0-12). Of FBT coaches, *n*=1 was a Doctor of Osteopathic Medicine (DO), *n*=1 nurse practitioner (NP), *n*=1 nurse, *n*=19 registered dietitians, *n*=8 social workers, *n*=6 working towards their degree, and *n*=7 other. Of eSOC providers, 78% were medical doctors, 7% DOs, 12% NPs, 2% physician assistants, and 1% other. Providers thus far have delivered FBT or eSOC for an average of 2.6 years within TEAM UP. Providers’ attitudes and knowledge were not significantly associated with the number of days that they participated in delivering the intervention.

**Implications for D&I Research:** FBT+eSOC and eSOC reached diverse samples of children from disadvantaged backgrounds. Providers remained engaged in delivering FBT+eSOC and eSOC regardless of baseline attitude or knowledge. As the trial concludes, further work will examine effectiveness, adoption (i.e., number of intervention sessions delivered), implementation, and maintenance. Evaluation of reach and adoption of FBT has potential to expand access to child obesity treatment.

**Primary Funding Source:** Patient-Centered Outcomes Research Institute

### S48 Implementation of the support for HIV integrated education, linkages to care, and destigmatization (SHIELD) program to strengthen the delivery of youth-friendly HIV services for adolescent girls and young women: An ingredients-based costing analysis

#### Natalie Blackburn^1^, Jenny Beizer^2,3^, Suzannah Scanlon^4^, Nachela Chelwa^5^, Lyson Phiri^5^, Drosin Mulenga^5^, Sophie Inambwae^5^, Sarah Roberts^6^, Laura Nyblade^4^, Yevgeniya Kaganova^2^, Michael Mbizvo^5^, Sujha Subramanian^2^

##### ^1^RTI International, Research Triangle, NC, USA; ^2^RTI International, Waltham, MA, USA; ^3^Harvard T.H. Chan School of Public Health, Cambridge, MA, USA; ^4^ RTI International, Washington, DC, USA; ^5^Population Council, Lusaka, Zambia; ^6^RTI International, Berkeley, CA, USA

###### **Correspondence:** Natalie Blackburn (nblackburn@rti.org)

*Implementation Science 2024*, **19(2):**S48

**Background:** Youth-friendly patient-level health care services can address HIV-related health outcomes among adolescent girls and young women (AGYW) but effective strategies for strengthening health care service engagement among AGYW are required. Community-centered interventions, including peer navigation, are effective strategies but have not been well defined in terms of cost. The objective of this study was to describe the implementation and costs of the Support for HIV Integrated Education, Linkages to care, and Destigmatization program (SHIELD) and integration into the delivery of youth-friendly services - Integrated Wellness Care (IWC) - in Lusaka, Zambia.

**Methods:** Using an ingredients-based costing approach, the following data sources were utilized: salary and budgetary analyses (staff interviews), peer navigator (PN) self-reported daily tasks, meeting and youth club attendance, and IWC time and motion studies conducted by clinic managers. We classified the costs of SHIELD and costs to integrate with IWC for the 375 AGYW receiving SHIELD only and 375 AGYW receiving coordinated SHIELD + IWC. Cost and intervention-related data were collected from July 2021 to December 2022 in Zambian Kwacha (ZMW) and United States Dollars (USD).

**Findings:** The IWC provides vaccinations, reproductive health services, and health-related needs in a youth-friendly clinical setting. The SHIELD program is delivered by a peer navigator (PN) monthly in a group session; the sessions cover social support, stigma, and self-efficacy in healthcare seeking behaviors. Average number of monthly sessions attended out of the 12 offered ranged from 6.6 to 7.4. The most common reason for PN visits with participants at the IWC was described as “improve overall wellbeing” (92%) in coordination with discussions of pregnancy (11%) or transportation issues (5%).

The total economic cost of SHIELD alone was $36,724 USD and IWC was $9,342 USD. For SHIELD, costs were greatest for participant follow-up (scheduling, session reminders, missed sessions). For IWC, costs were greatest for the visits (in-clinic and in-home), followed by participant follow-up (scheduling, appointment reminders).

**Implications for D&I Research:** Understanding the costs of community-centered implementation strategies for strengthening existing health care models are needed to inform scale-up and adaptation, particularly for policymakers who require understanding of economic costs to make such decisions.

**Primary Funding Source:** National Institutes of Health

### S49 Enhancing systems of care for children with medical complexity: Participatory design of a multi-site evaluation

#### Elizabeth Cope^1^, Sarah Hoyt^1^, Maria Mutka^1^, LaToshia Rouse^2^, Richard Antonelli^3^, Jeff Schiff^4^

##### ^1^AcademyHealth, Washington, DC, USA; ^2^Family Voices, Greenville, NC, USA; ^3^Boston Children's Hospital, Boston, MA, USA; ^4^Minnesota Department of Human Services, St. Paul, MN, USA

###### **Correspondence:** Elizabeth Cope (elizabeth.cope@academyhealth.org)

*Implementation Science 2024*, **19(2):**S49

**Background:** Five HRSA-funded sites in four states are implementing evidence-informed care models designed to optimize care experience and outcomes for children with medical complexity and their families. Care models should meet health home criteria and incorporate expanding access to services, optimizing use of innovative technologies, and applying equity-informed approaches to equip families with needed resources. Herein, we present results from a participatory design process for the initiative’s cross-site evaluation (to be conducted 2023-2027).

**Methods:** Multistakeholder partnerships and advisory groups were established to guide all activities and support participatory approaches--collectively engaging 53 patients/family members, clinical and social service delivery leaders, content experts, Title V, Medicaid, HRSA, and others. Evaluation design inputs included implementation science frameworks; literature reviews; review of site-specific data collection and evaluation plans; structured and unstructured engagement with advisory groups; and consultation with experts from adjacent initiatives. Top-down and bottom-up strategies were jointly applied to balance internal/external validity. Rank-order, rating scale, and open questions were used to solicit feedback on proposed data collection items. Town hall and deliberative methods were used for synchronous engagement. Sequencing and comingling techniques were used to balance ensuring all voices were heard with the benefits that come from collective empathy-building.

**Findings:** Evaluation design sought to balance: importance to patients/families; actionability and salience for clinical leaders; burden across parties; information needs for sustainability (policy); and generalizable lessons to inform scalability. Stakeholder contributions resulted in refinement of three overarching research questions, adjustment from outcome to process evaluation (including framework), revisions to more than 50% of metrics and survey questions (domains: access, coordination experience, engagement, cultural sensitivity, and compassion), and refinements to qualitative data protocols (family interviews; implementation tracking). The process highlighted the importance of relationship-building in longitudinal engagement, as well as the need for both targeted and mixed engagement opportunities to ensure all voices can be heard while promoting an “all teach, all learn” culture.

**Implications for D&I Research:** Participatory implementation science has been shown to enhance uptake and sustainability of evidence-informed practices. This work contributes to the field by documenting real-world examples of how this can be operationalized to meet competing needs across service providers, families served, policymakers, and research goals.

**Primary Funding Source:** Health Resources and Services Administration

### S50 A theory-informed, mixed-methods approach to assess the real-world trial feasibility and implementation potential of a telehealth-enhanced hybrid vs. traditional cardiac rehabilitation program: A pilot randomized controlled trial

#### Andrea T. Duran^1^, Adrianna Keener-DeNoia^2^, Emma K. Whittman^1^, Maria A. Serafini^1^, Kimberly Stavrolakes^3^, Emily Fleisch^3^, Nicole Pieszchata^3^, Donald Edmondson^1^, Rachel C. Shelton^4^, Nathalie Moise^1^

##### ^1^Columbia University Irving Medical Center, New York, NY, USA; ^2^Teachers College, Columbia University, New York, NY, USA; ^3^NewYork-Presbyterian Hospital, New York, NY, USA; ^4^United States, Columbia Mailman School of Public Health, New York, NY, USA

###### **Correspondence:** Andrea T. Duran (atd2127@cumc.columbia.edu)

*Implementation Science 2024*, **19(2):**S50

**Background:** We evaluated the feasibility of conducting a pilot randomized controlled trial (RCT) comparing telehealth-enhanced hybrid cardiac rehabilitation (THCR) with traditional cardiac rehabilitation (CR; Aim 1) and the implementation potential of each intervention (Aim 2) in a real-world hospital setting.

**Methods:** Acute coronary syndrome (ACS) patients from a university hospital in Manhattan, New York were enrolled into a single center, two-arm, 1:1 parallel group pilot RCT comparing THCR (5 clinic-based + 19 home-based sessions) with traditional CR (24 clinic-based sessions). For Aim 1, we assessed recruitment (% patients consented/randomized), program participation (initiation [≥1 CR session], fidelity [# of total CR sessions], completion [24 CR sessions]), and outcome assessment (functional capacity; health-related quality of life) for both arms using electronic health record data. For Aim 2, we assessed Proctor’s implementation outcomes (feasibility, acceptability, appropriateness) and implementation determinants using validated 4-item measure surveys (1-5 Likert scale) and semi-structured exit interviews, respectively. Implementation determinants were categorized into relevant Consolidated Framework for Implementation Research (CFIR) 2.0 domains and constructs.

**Findings:** From March-December 2022, we approached 27 ACS patients, of which 37% (n=10) were enrolled and randomized to THCR (n=5; 60.9±10.7 years, 100% male, 80% non-Hispanic White, 40% Medicare/Medicaid) or traditional CR (n=5; 61.4±13.1 years, 60% male, 40% non-Hispanic White, 20% Medicare/Medicaid). All participants initiated the program and completed outcome assessments, with greater program adherence (21.0±6.7 vs. 16.8±10.1 sessions) and completion rates (80% vs. 60%) detected among THCR participants. Participants in each arm (THCR vs. traditional) reported similar program feasibility (mean±SD: 4.8±0.4 vs. 4.7±0.5), acceptability (4.9±0.3 vs. 4.5±0.5), and appropriateness (4.7±0.5 vs. 4.3±0.7). Key patient-perceived implementation determinants were related to CFIR constructs in the innovation (relative advantage, design, cost), inner setting (compatibility, available resources), and individuals’ domains (motivation, capability), with differences detected between arms.

**Implications for D&I Research:** We successfully applied a theory-informed, mixed-methods approach to assess RCT feasibility and implementation potential of a nontraditional CR program with standard of care within a real-world hospital setting. Complementing the translational research pipeline with implementation science methods at earlier stages of behavioral intervention development may provide foundational data that can be used to support enhanced real-world efficacy/effectiveness and implementation of future nontraditional CR models.

**Primary Funding Source:** National Institutes of Health

### S51 Guess what? you have prediabetes, also. Examining prediabetes knowledge, barriers/facilitators to lifestyle change, and preference for prediabetes education among patients with screen-detected prediabetes in an emergency department

#### Angela Kong^1^, Mary Smart^1^, Deci Limpoco^1^, Yuka Asada^2^

##### ^1^University of Illinois Chicago, Chicago, IL, USA; ^2^School of Public Health, UIC, Chicago, IL, USA

###### **Correspondence:** Angela Kong (akong@uic.edu)

*Implementation Science 2024*, **19(2):**S51

**Background:**

Timely detection of prediabetes through screening can provide patients with an earlier opportunity to intervene. However, delays in diagnosis disproportionately affect racially/ethnically minoritized and lower socioeconomic status (SES) groups. In response to this inequity, an emergency department (ED) in Chicago, IL began routinely screening patients for prediabetes and diabetes using hemoglobin A1C tests. Given this unique setting, little is known about ED patients with screen-detected prediabetes and how to connect them to prediabetes education. This study aimed to 1) examine patients’ prediabetes awareness and knowledge, 2) identify barriers/facilitators to lifestyle change, and 3) assess interest in and delivery preferences for prediabetes education.

**Methods:**

A purposive sample of ED patients (n=35) with screen-detected prediabetes (Hba1c: 5.7 to 6.4%) were interviewed. The interview guide and coding were informed by the Theoretical Domains Framework(TDF). Patients also rated their motivation (1:not motivated; 5:very motivated) and self-efficacy(1: not confident; 5: completely confident) for lifestyle change and responded to prediabetes education options. Qualitative data was merged with quantitative responses to contextualize ratings.

**Findings:**

In this racially/ethnically diverse sample (65.7% African American, 25.7% Latina/o/x, 5.7% Asian, 2.9% Native American), 57% were unaware they had prediabetes and knowledge about the condition was limited. Motivation to eat healthfully (mean: 4.5, SD: 0.76) was high and mostly explained by patients’ intention to avoid diabetes after observing family members struggle with the condition (TDF construct: belief about consequences). Patients were somewhat less motivated to exercise (mean: 3.9; SD: 1.0) due to busyness and medical conditions that interfered with exercise. High self-efficacy for eating healthfully (mean:4.29; SD:1.0) and exercising (mean:4.1; SD:1.3) were reported by patients who already engaged in changes(TDF construct: skills). Barriers to lifestyle change included food insecurity, time constraints, limited access to healthy foods and safe spaces. Most patients (85%) were interested in prediabetes education and preferred interactive (phone calls or text messaging with a diabetes educator) over passive options (videos, automated text messages).

**Implications for D&I Research:**

These findings can be used to inform the development and delivery of prediabetes education that considers the perceptions, context, and preferences of patients with screen-detected prediabetes in an urban ED.

**Primary Funding Source:** The Chicago Center for Diabetes Translation Research (CDTR) Pilot & Feasibility Program

### S52 Applying the EPIS framework to guide the development of a pharmacy-based colorectal cancer screening program: A series of PharmFIT™ studies

#### Alison Brenner^1,2,3^, Mary Wangen^3^, Austin Waters^3^, Catherine Rohweder^3^, Morgan Glascock^4^, Macary Marciniak^5^, Olufeyisayo Odebunmi^3^, Renée Ferrari^3^, Stephanie Wheeler^3^, Parth Shah^4^

##### ^1^Division of General Medicine and Clinical Epidemiology, University of North Carolina School of Medicine, Chapel Hill, NC, USA; ^2^Lineberger Comprehensive Cancer Center, University of North Carolina at Chapel Hill, Chapel Hill, NC, USA; ^3^University of North Carolina at Chapel Hill, Chapel Hill, NC, USA ^4^Fred Hutchinson Cancer Center, Seattle, WA, USA; ^5^UNC Eshelman School of Pharmacy, Chapel Hill, NC, USA

###### **Correspondence:** Alison Brenner (alison.brenner@unc.edu)

*Implementation Science 2024*, **19(2):**S52

**Background:** Increasing access to colorectal cancer (CRC) screening among medically underserved populations requires the development of additional equitable. Pharmacies provide a viable option as they are conveniently located, frequently visited by individuals, and pharmacists regularly offer counseling and preventive care services. Fecal immunochemical testing (FIT) for CRC is well-suited for pharmacy-based distribution, given its home-based administration and community lab processing.

**Methods:** The Exploration, Preparation, Implementation, and Sustainability (EPIS) Framework was used to guide the development of PharmFIT™. In the Exploration phase, qualitative interviews were conducted with patients eligible for CRC screening (*N*=32), community pharmacists (*N*=23), and primary care providers (PCPs) (*N*=30) to gauge perceptions of PharmFIT™. National surveys were administered to patients (*N*=1,045) and pharmacists (*N*=578) to assess willingness to use PharmFIT™ and preferences for design. In the Preparation phase, we pilot tested PharmFIT™ in two states, using collaborative process flow diagramming (PFD) for intervention planning. A larger randomized controlled trial and cost-effectiveness analysis are planned for the Implementation and Sustainability phases.

**Findings:** Exploration phase activities indicated general acceptability of PharmFIT™ across all participant groups. Patients expressed the advantages of easy access to CRC screening. PCPs and pharmacists displayed enthusiasm, emphasizing the need for coordinated care during implementation. Patient surveys revealed high willingness to utilize PharmFIT™ (72%), particularly among those perceiving its advantage over usual care. Pharmacist surveys similarly revealed high willingness to provide PharmFIT™ (81%), especially when accounting specific design preferences (reporting results to PCP, training, and workflow integration). In the Preparation phase, PharmFIT™ models, based on design preferences revealed in the Exploration phase, were informed by collaborative PFD with our pilot sites. In the three distinct designs that emerged, patients were recruited from an insurance group, a PCP’s e-prescriptions, and from a panel of patients due for CRC screening. Pilot results showed high rates of response (FIT pick-up) (40-93%) and completion (FIT return) (88-100%) across the models.

**Implications for D&I Research:** By employing the EPIS framework, the PharmFIT™ study developed and pilot-tested a highly successful CRC screening program, aiming to enhance screening accessibility for medically underserved populations. The next steps involve a larger randomized controlled trial and cost-effectiveness analysis to facilitate implementation and sustainability.

**Primary Funding Source:** Centers for Disease Control and Prevention

## Clinical Care Settings: System-level Interventions

### S53 Effect of patient centered care strategy to change provider behaviors and improve patient experience during HIV treatment in Zambia, a stepped-wedge trial

#### Kombatende Sikombe^1,2^, Aaloke Mody^3^, Charles Goss^4^, Sandra Sinzala Simbeza^5^, Laura Beres^6^, Jake Pry^7^, Ingrid Eshun-Wilsonova^8^, Anjali Sharma^7^, Njekwa Mukamba^1^, Brian Rice^9^, Jacob Mutale^7^, James Hargreaves^2^, Carolyn Bolton^10^, Charles Holmes^11^, Izukanji Sikazwe^1^, Elvin Geng^4^

##### ^1^Centre for Infectious Disease Research in Zambia (CIDRZ), Lusaka, Zambia; ^2^London School of Hygiene and Tropical Medicine, London, United Kingdom; ^3^University of Washington in St. Louis, St. Louis, MO, USA; ^4^Washington University in St. Louis, St. Louis, MO, USA; ^5^Plot No 34620 Corner of Lukusu and Danny Pule Roads, Lusaka, Zambia; ^6^Department of International Health, Johns Hopkins Bloomberg School of Public Health, Baltimore, MD, USA, Baltimore, USA; ^7^Centre for Infectious Disease Research in Zambia, Lusaka, Zambia; ^8^Johnson and Johnson, Capetown, South Africa; ^9^University of Sheffield, Sheffield, United Kingdom; ^10^P.O Box 24681, Centre for Infectious Disease Research in Zambia, Lusaka, Zambia; ^11^Johns Hopkins University, Baltimore, MD, USA

###### **Correspondence:** Kombatende Sikombe (Kombatende.Sikombe@cidrz.org)

*Implementation Science 2024*, **19(2):**S53

**Background:**

Poor client-provider interactions can drive poor retention in HIV care, but how to change health care worker (HCW) attitudes, communication, and practice is poorly understood. We co-designed with HCW and evaluated a multicomponent Person-Centred Care (PCC) strategy targeting HCW behavior to improve patient experience, and, ultimately, retention and viral suppression under real world service delivery conditions in Zambia.

**Methods:**

We implemented a three-part PCC curriculum strategy in 24 clinics in Lusaka, Zambia in a stepped wedge trial (August 2019-November 2021), over approximately four six-month periods. The PCC strategy had three components: 1) HCW PCC training and mentoring, 2) systematic measurement and feedback of patient experience metrics (satisfaction, HCW attitude/communication) and, 3) small facility-level incentives for improved performance. We assessed the impact on improved patient experience, which we hypothesised would have downstream effects on retention in care after 15 months, reduction in missed appointments and treatment failure at 15 months (HIV RNA > 400 copies/ml or disengagement). We used mixed-effects logistic regression, accounting for clustering at facility-level using random effects, to assess the effect of PCC on patient experience and clinical outcomes adjusting for sex, age, and care status.

**Findings:**

Among 1,111 client visits, patient experience improved after at least six months of intervention (Sum score mean, 0.85 of a total score of 7; 95% CI: 0.37 - 1.32). Among all individuals represented in the electronic health record at the clinic (N=84,926), retention at 15-months increased by 4.7% [95% CI: -0.3 - 9.7] and missed visits declined by -3.3% ([95% CI: -3.7 - 2.9] N=1,087,809 visits). We found no difference in treatment failure between control (N=453) and intervention (N=480) (RD=0.9%, 95% CI: -5.4 - 7.2) overall. Across facilities, we trained 60-100% of HCWs and conducted mentorship visits at least once a week at the beginning of each period and conducted data feedback review meetings every three months.

**Implications for D&I Research:**

The PCC strategy targeting knowledge, skills, and motivation of providers improved patient experience as well as retention in care. Systematic strategies targeting provider behavior and organizational culture have potential to optimize care delivery and improve overall outcomes.

**Primary Funding Source:** Bill and Melinda Gates Foundation

### S54 Low implementation fidelity to client-centred HIV post-test counselling in primary healthcare clinics in South Africa: A case of unintended negative consequences of performance targets

#### Tonderai Mabuto^1^, Cebile Hlophe^1^, Salome Charalambous^1^, Rohit Ramaswamy^2^

##### ^1^The Aurum Institute, Johannesburg, South Africa; ^2^Cincinnati Children’s Hospital Medical Center, Cincinnati, USA

###### **Correspondence:** Tonderai Mabuto (tmabuto@auruminstitute.org)

*Implementation Science 2024*, **19(2):**S54

**Background:** HIV testing service guidelines in South Africa require counsellors to provide client-centred post-test counselling that tailors patient-provider communication to the values, preferences, and needs of people receiving an HIV-positive diagnosis. Low fidelity to client-centred counselling undermines the anticipated benefits of antiretroviral therapy (ART) to reduce HIV transmission and may negatively affect ART uptake. We sought to qualitatively explore the determinants for implementation fidelity to client-centred post-test counselling from the perspective of actors in the implementation support system providing technical assistance (TA) to counsellors in 43 primary healthcare clinics (PHCs) in the North West Province of South Africa.

**Methods:** From November 2022-January 2023, we conducted virtual in-depth interviews with nine Counsellor Supervisors and six Training Officers who provided TA to counsellors working in PHCs in the study setting. We used the organisational readiness heuristic to develop an interview guide to explore the role played by the general capacity of PHCs, counsellor capacity, and counsellor motivation on implementation fidelity to client-centred counselling. All interviews were audio-recorded and transcribed for thematic analysis.

**Findings:** Participants perceived that most PHCs lacked the general capacity, and most counsellors lacked the intervention-specific capacity and motivation to implement client-centred counselling. All participants explained that giving counsellors daily HIV testing targets (i.e., number to be tested and new HIV-positive cases identified) negatively affected all three organisational readiness components and emerged as a key root cause for low implementation fidelity. Participants perceived that an overemphasis by managers on daily targets, created an implementation climate where counsellors subverted client-centred counselling to meet daily targets by resorting to didactic and paternalistic counselling approaches. Further, participants were concerned that without openly discussing these unintended effects of performance targets on client-centred counselling, commonly used implementation strategies such as refresher training, and mentorship would continue to have limited success in improving the quality of counselling.

**Implications for D&I Research:** Our findings highlight the value of engaging actors in the implementation support system to characterise determinants that may not be easily identified from the direct delivery system. We also highlight the importance of exploring unintended consequences of implementation strategies, particularly the use of performance targets in routine HIV programmes.

**Primary Funding Source:** National Institutes of Health

### S55 Cost as key to sustainability: Revenue optimization as an implementation strategy for integrated behavioral health

#### Charlotte Vieira^1^, Elijah Boliver^1^, Anita Morris^1^, Radley Sheldrick^2^

##### ^1^Boston Medical Center, Boston, MA, USA; ^2^UMass Chan Medical School, Worcester, MA, USA

###### **Correspondence:** Charlotte Vieira (charlotte.vieira@bmc.org)

*Implementation Science 2024*, **19(2):**S55

**Background:** Cost is a key determinant for successful implementation. Yet financial sustainability, while ranked highest in determining stakeholder buy-in, is frequently under-reported in implementation science.

TEAM UP for Children is an integrated behavioral health (BH) initiative designed to transform pediatric care by increasing access to BH services within community health centers (CHCs). Revenue optimization programming was conducted to ensure sufficient and reliable revenue for implementing and sustaining new behavioral health clinician (BHC) roles at CHCs. This process involved collection of billing and reimbursement information and establishing and convening billing champions across CHCs. Further, we tracked BHC salary coverage to ensure optimal, clinically-relevant coding and reimbursement practices through time.

**Objective:** To demonstrate the utility of revenue optimization as an implementation strategy, including use of tools to facilitate the process and evaluate outcomes.

**Methods:**

**Population:** Four CHCs in Massachusetts participating in TEAM UP.

**Design:** Reports were completed biannually and contained the number and type of billable encounters per week, average reimbursement rates based on available fee schedules, average denial rates, and BHC salary expenses. Overall BHC salary coverage was calculated for each submission, accounting for fluctuations in patient engagement and reimbursement over time.

**Analyses:** Reports were compiled and analyzed using Stata-17 to calculate estimated salary expense coverage via direct BHC-accrued revenue through time and determinants of changes in revenue.

**Findings:** By the end of the implementation period, participating CHCs were covering, on average, 104% of their BHC salary expenses. Multiple revenue drivers were identified. These included anticipated elements such as staffing models, clinical productivity, and training, and unanticipated shifts in state-level healthcare financing policy, Electronic Medical Record functionality, and nuances in CHC licensing and payer contracts.

CHCs described reports as feasible to complete and useful for program sustainment and identification of opportunities for revenue optimization.

**Conclusions:** Our case example demonstrates the importance of routinely capturing and tracking BHC-related revenue and expenses as it can predict and facilitate the financial sustainability of integrated care.

**Implications for D&I Research:** Simple and feasible methods of cost modeling can improve success of implementation efforts and address an often-overlooked component of sustainability.

**Primary Funding Source:** Philanthropic Foundations

### S56 Using implementation mapping to guide the implementation of an intensive blood pressure control intervention in federally qualified health centers

#### Samantha O'Connell^1^, Erin Peacock^2^, Farah Allouch^1^, Maria E. Fernandez^3^, Amy K. Carreras^1^, Jing Chen^2^, Marie Krousel-Wood^2^, Jiang He^4^, Katherine T. Mills^1^

##### ^1^Tulane University School of Public Heath and Tropical Medicine, New Orleans, LA, USA; ^2^Tulane University School of Medicine, New Orleans, LA, USA; ^3^The University of Texas Health Science Center at Houston School of Public Health, Houston, TX, USA; ^4^Tulane University, New Orleans, LA, USA

###### **Correspondence:** Samantha O'Connell (soconne1@tulane.edu)

*Implementation Science 2024*, **19(2):**S56

**Background:** The Implementation of Multifaceted Patient-Centered Treatment Strategies for Intensive Blood Pressure Control (IMPACTS) study is a cluster-randomized hybrid type II trial conducted in 36 Federally Qualified Health Center (FQHC) clinics in Louisiana and Mississippi. This study describes the application of Implementation Mapping (IM) to identify determinants of implementation of an intensive blood pressure (BP) control intervention, create matrices of change objectives, and map change objectives to appropriate implementation strategies.

**Methods:** Using IM, we identified program implementers and determined who would deliver different components of the multifaceted implementation strategy (Task 1). We then stated performance objectives, identified determinants, and created matrices of change objectives (Task 2). We used matrices to choose mechanisms of change (theory-based change methods) and selected implementation strategies that operationalized those methods that focused on addressing determinants of implementation for each type of program implementer (Task 3). The Theoretical Domains Framework was used to specify key determinants of implementation behaviors.

**Findings:** IM Task 1 determined providers, health coaches, and patients were required to implement different components of the multifaceted implementation strategy including dissemination of SPRINT findings, team-based collaborative care, BP audit and feedback, home BP monitoring, and health coaching. In Task 2, the behavioral determinants key to implementation included knowledge, beliefs, self-efficacy, skills, outcome expectations, and social/professional role and identity. Behavioral determinants varied by implementer. Key change objectives for providers included modifying existing beliefs of the benefits and harms of intensive BP treatment. Among health coaches, change objectives included increasing self-efficacy to collaborate with patients, clinic staff, and providers. Patient change objectives involved achieving confidence in BP management. Task 3 identified methods and practical applications which addressed the change objectives and facilitate implementation. Practical applications included development of presentation slides, implementer (healthcare providers and health coaches) trainings, handouts, manuals, and protocols.

**Implications for D&I Research:** This study advances implementation science by furthering our understanding of the use of IM for hypertension interventions in low-resource primary care settings. IM in this setting indicates behavioral determinants for implementation vary by implementer. Providers, health coaches, and patients in FQHCs require a variety of methods to aid behavioral change.

**Primary Funding Source:** National Institutes of Health

### S57 Mentored de-implementation initiatives to enhance medication deprescribing at nursing homes during the COVID-19 pandemic

#### Sunil Kripalani

##### Vanderbilt University Medical Center, Nashville, TN, USA

###### **Correspondence:** Sunil Kripalani (sunil.kripalani@vanderbilt.edu)

*Implementation Science 2024*, **19(2):**S57

**Background:** Optimizing medication management is a major challenge in nursing homes, where elderly patients have a high risk of harmful adverse drug events due to frequent polypharmacy (≥10 medications) and prescription of sedating medications. During the COVID-19 pandemic, facilities sought to streamline medication regimens to reduce medication burden and risk of adverse events, as well as to limit repeated contact and virus transmission. We worked with nursing homes participating in a mentored quality improvement (QI) initiative to deprescribe or simplify medication regimens, a form of de-implementation.

**Methods:** A study nurse practitioner (NP) and pharmacist led 3 facilities through a structured assessment of medication-related opportunities, root cause analysis, setting a specific goal, Plan-Do-Study-Act (PDSA) cycles, and evaluation. Local QI efforts were led by nursing home staff including the director of nursing, providers, and consultant pharmacists.

**Findings:** Two facilities focused on deprescribing unnecessary medications and decreasing the total number of medication passes, while the third facility focused specifically on deprescribing antipsychotics. Over 9-20 months, facilities completed 2-3 PDSA cycles; NPs supported facilities with an average of 6 meetings and 18 other communications. Facility 1 successfully achieved 85% of residents on <10 total medications, and reduced medication passes from 3.5 to 2 per day. Facility 2 reduced total medications by approximately 15%, or 2 medications per resident. Facility 3 encountered barriers in deprescribing antipsychotics but ultimately reduced or stopped low-dose antipsychotics in 63% of targeted patients. Strategies used in de-implementation included a learning collaborative, external facilitation, champions, involving patients and family members, and providing ongoing consultation. Facilitators to deprescribing included 1) facility administration support, 2) collaboration with facility providers and/or consultant pharmacist, and 3) family education related to medication changes. Barriers included staffing shortages, staff turnover, and competing priorities related to COVID-19 outbreaks.

**Implications for D&I Research:** Mentored de-implementation using QI tools can lead to successful deprescribing in high-risk environments. It is imperative to engage support of key stakeholders including facility leadership and consultant pharmacists.

**Primary Funding Source:** Centers for Medicare and Medicaid Services

### S58 Barriers and enablers of patients and outpatient providers to deprescribing recommendations in a clinical trial (Shed-MEDS)

#### Amanda Mixon

##### Vanderbilt University Medical Center, Nashville, TN, USA

###### **Correspondence:** Amanda Mixon (amanda.s.mixon@vumc.org)

*Implementation Science 2024*, **19(2):**S58

**Background:** Few studies have examined how patients and outpatient providers respond to recommendations initiated in the hospital about stopping or reducing medications (deprescribing). In a clinical trial to reduce polypharmacy (Shed-MEDS), we assessed patient participants’ and outpatient providers’ willingness to deprescribe medications and the associated barriers and enablers to inform future adaptations for de-implementing harmful medications.

**Methods:** The clinical trial included hospitalized older patients requiring additional care in a post-acute care setting and taking 5 or more medications. A study clinician reviewed all medications and reviewed deprescribing recommendations with the patient. Patients’ responses were categorized into barriers and enablers from a published framework: appropriateness of cessation, pragmatic considerations, fear (return of condition), dislike of a medication, influences (impactful events/people), and process of cessation. If the patient agreed, the study clinician attempted to contact outpatient provider(s) to discuss the recommendations. Providers’ responses were categorized into barriers and enablers from another published framework: awareness (insight), inertia (failure to act), self-efficacy (belief and confidence), feasibility (external factors), and/or tacit (no clear reason given).

**Findings:** Participants (N=177) agreed with 63% (883 total medications) of the study clinician’s deprescribing recommendations. Thematic analysis revealed that appropriateness of a medication was the most common barrier (88%) and enabler (67%) to deprescribing. Other deprescribing enablers were: influences (23%), process (22%), pragmatic considerations (19%), and dislike (5%). Provider conversations were completed for 98 (76%) of the 129 intervention patients with deprescribing recommendations. Among provider conversations, 349 medications (mean = 4.5+2.9 per patient) were discussed. Outpatient providers agreed to deprescribe 291 (83%) medications. The most common enablers to deprescribing were categorized as tacit (28%), self-efficacy (25%), and inertia (24%). However, inertia (39%) and self-efficacy (35%) also were common barriers.

**Implications for D&I Research:** Participants and outpatient providers agreed with the majority of deprescribing recommendations. Appropriateness being the most reported barrier indicates the deprescribing recommendation did not suffice to change the patient’s belief in the need for cessation. Inertia was the most frequent barrier for outpatient providers. These results inform future efforts to better engage patients in deprescribing conversations and coordinate efforts between the hospital and outpatient settings for successful de-implementation of harmful medications.

**Primary Funding Source:** National Institutes of Health

### S59 Decreasing pre-procedural fasting times in hospitalized children

#### Alison Carroll

##### Vanderbilt University Medical Center, Nashville, TN, USA

###### **Correspondence:** Alison Carroll (alison.carroll@vumc.org)

*Implementation Science 2024*, **19(2):**S59

**Link:** https://shmpublications.onlinelibrary.wiley.com/doi/10.1002/jhm.12782

### S60 The role of interprofessional education for implementation of complex therapies in the intensive care unit: The METEOR trial

#### Timothy Girard

##### University of Pittsburgh, Pittsburgh, PA, USA

###### **Correspondence:** Timothy Girard (timothy.girard@pitt.edu)

*Implementation Science 2024*, **19(2):**S60

**Background:**

Continuing education is a cornerstone of efforts to implement evidence-based practice. However, traditional education only occurs within professional silos. Interprofessional education (IPE) can overcome this problem by creating shared-mental models about evidence-based practices. We sought to better understand the role of IPE in the context the intensive care unit (ICU), a dynamic care environment where interprofessional collaboration is key to adoption of best practice.

**Methods:**

We interviewed and surveyed ICU providers in a large mid-Atlantic health system about the barriers and facilitators to two preventive post-extubation respiratory support strategies with demonstrated efficacy: non-invasive ventilation (NIV) and oxygen by high-flow nasal cannula (HFNC). We then assessed current usage rates and piloted a multi-component implementation intervention centered around IPE. We used these results to design the Maximizing Extubation outcomes Through Educational and Organizational Research (METEOR) trial, a cluster-randomized, stepped-wedge, hybrid effectiveness-implementation trial of IPE for preventive post-extubation respiratory support.

**Findings:**

In qualitative interviews (n=32) providers endorsed the lack of effective communication as a barrier to effective care delivery in the ICU. A web-based survey (n=482) confirmed a lack of consensus among team members about the value of preventive post-extubation therapy (Table). Rates of use were less than 5% and were increased in a single-center pilot of an IPE-based intervention (RR: 4.9, 95% CI: 1.4–17.3). Based on this work we launched the METEOR trial in March 2023, which will randomize 28 ICUs, comparing IPE to traditional on-line education. ICUs will also be randomized to receive education focused on either NIV or HFNC. The endpoints are implementation of best-practice and in-hospital mortality at 60 days.

**Implications for D&I Research:**

METEOR will be among the first systematic evaluations of IPE as an implementation strategy in inpatient care, substantially expanding the evidenced-base around the role of IPE in the adoption of evidence-based therapies that rely on interprofessional collaboration.

**Primary Funding Source:** National Institutes of Health

**Table 1 (abstract S60).** Percent agreement with statements about preventive post-extubation respiratory support in the ICU

	RN	RT	MD	*P*-value
Post-extubation NIV is beneficial for high-risk patients	59%	56%	79%	*p*<0.001
Post-extubation NIV is often contraindicated	63%	70%	43%	*p*<0.001
Other team members are opposed to post-extubation NIV	45%	27%	23%	*p*<0.001

### S61 Implementation of coordinated spontaneous awakening and breathing trials using telehealth-enabled, real-time audit and feedback for clinician adherence (TEACH): A type II hybrid effectiveness-implementation

#### Andrew J Knighton

##### Intermountain Healthcare, Murray, UT, USA

###### **Correspondence:** Andrew J Knighton (andrew.knighton@imail.org)

*Implementation Science 2024*, **19(2):**S61

**Background:**

Although invasive mechanical ventilation (IMV) is a lifesaving treatment for about 300,000 U.S. patients with acute respiratory failure each year, it is associated with significant risks. Spontaneous awakening and breathing trials during IMV improve patient outcomes. Coordination of spontaneous awakening and breathing trials (C-SAT/SBT) is complex and significant barriers to implementation exist and adherence with C-SAT/SBT across institutions highly variable. Telehealth-enabled remote care is positioned to improve C-SAT/SBT use. At Intermountain Health, we have system-wide tele-critical care services. This approach could help identify candidates for C-SAT/SBT protocols, prompt bedside providers to perform C-SAT/SBT and guide execution, taking responsibility for certain activities usually held by busy front-line supervisors.

**Methods:**

We determined baseline adherence to C-SAT/SBT best practices across in 16 intensive care units spread across 12 Intermountain Health hospitals and conducted 55 field interviews with physicians, nurses and RTs across the system. We adapted baseline implementation strategies to target C-SAT/SBT using the Consolidated Framework for Implementation Research (CFIR) and initiated a type II cluster- randomized hybrid effectiveness-implementation trial beginning to compare a usual supervisor-driven audit and feedback implementation approach to a usual audit and feedback implementation approach augmented with a Telehealth-Enabled, real-time Audit and feedback for Clinician adHerence (“TEACH”) to promote C-SAT/SBT.

**Findings:**

Primary implementation strategies included deploying the usually audit and feedback approach augmented with TEACH at randomized sites, along with management emphasis, deploying local training materials and developing of a monitoring system. For the measurement period through May 2023, we have seen increased adherence to SAT, SBT and Combined SAT/SBT across all study sites (Table).

**Implications for D&I Research:**

Pairing of clinical effectiveness researchers with implementation science researchers will advance knowledge regarding the effective and sustainable strategies for C-SAT/SBT implementation specifically and the effectiveness generally of telehealth remote monitoring and prompting strategies to aid best practice implementation in ICUs. The final study aim will evaluate difference in long-term sustained use of c-SAT/SBT between TEACH and non-TEACH sites.

**Primary Funding Source:** National Institutes of Health

**Table 1 (abstract S61).** Adherence Results to Date

Measure	Baseline Period (Jan – Jun 2022)	Measurement Period (Dec 2022 – May 2023)
**SAT**	60.3%	88.3%
**SBT**	58.1%	90.3%
**Combined SAT/SBT**	42.9%	81.4%

### S62 Sustainment of continuous pulse oximetry deimplementation: The eliminating monitor overuse (EMO) cluster-randomized hybrid type III effectiveness-deimplementation trial

#### Christopher Bonafide

##### Perelman School of Medicine, University of Pennsylvania, Philadelphia, PA, USA; Children's Hospital of Philadelphia, Philadelphia, PA, USA

###### **Correspondence:** Christopher Bonafide (bonafide@chop.edu)

*Implementation Science 2024*, **19(2):**S62

**Background:**

Viral bronchiolitis is a common cause of infant hospitalization. Using continuous pulse oximetry (cSpO2) to monitor children with bronchiolitis who are not receiving supplemental oxygen is a form of medical overuse discouraged by national guidelines. The Eliminating Monitor Overuse (EMO) Trial, currently underway, seeks to identify the optimal strategies to promote sustained deimplementation of cSpO2 in bronchiolitis.

**Methods:**

This cluster-randomized trial includes 3 phases: Baseline (P1), Active Deimplementation (P2), and Sustainment (P3). In the Baseline phase, we measured cSpO2 overuse in 50 hospitals. We then randomized 38 hospitals to 2 arms (standard versus enhanced approach). In both arms, during Active Deimplementation, we conducted educational outreach and 2 forms of audit and feedback (A&F): (1) group-level feedback and (2) individual-level real-time inquiry. In hospitals randomized to the enhanced approach, we also provided pediatric EHR coaches to each site to support integration of appropriate clinical decision support. After P2, all sites ceased educational outreach and A&F; EHR decision support remained active in the enhanced approach arm. In the Sustainment phase (not yet started), we will re-measure cSpO2 overuse in all sites. There will be no additional education or A&F, but EHR decision support will remain active in enhanced approach arm sites. The primary outcome is sustainment of deimplementation.

**Findings:**

In P1, sites conducted 2323 cSpO2 patient observations; 54% were cSpO2-monitored contrary to guidelines. In P2, 36 sites convened a total of 713 educational outreach and group-level A&F sessions and conducted 2051 cSpO2 patient observations; 30% were continuously monitored, for an adjusted reduction of 24 percentage points (95% CI 20-28). There were no significant differences in the adjusted reduction between the (deidentified) trial arms, consistent with our hypothesis that the differences will arise in the Sustainment phase (P3) due to superior routinization and institutionalization produced by EHR decision support.

**Implications for D&I Research:**

Within a diverse pediatric hospital research network, sites were highly engaged and successfully reduced the overuse of an entrenched monitoring practice. The next phase will provide new insights into the role of EHR decision support in promoting sustainment of deimplementation after withdrawal of personnel-intensive educational outreach and A&F.

**Primary Funding Source:** National Institutes of Health

### S63 Patient- and clinician-directed implementation strategies to improve serious illness communication for high-risk patients with cancer: A cluster-randomized pragmatic trial

#### Samuel Takvorian^1^, Alicia Clifton^1^, Peter Gabriel^2^, E. Paul Wileyto^1^, David Asch^1^, Alison Buttenheim^2^, Katharine Rendle^1^, Rachel Shelton^3^, Krisda Chaiyachati^1,4^, Oluwadamilola Fayanju^1^, Susan Ware^1^, Lynn Schuchter^2^, Pallavi Kumar^1^, Lawrence Shulman^1^, Adina Lieberman^5^, Callie Scott^1^, Corey Chivers^6^, Nina O'Connor^1,7^, Tasnim Salam^1^, Daniel Ragusano^1^, Daniel Blumenthal^1^, Sharon Tejada^1^, Justin Bekelman^1^, Rinad Beidas^8^, Robert Schnoll^1^, Ravi Parikh^1^

##### ^1^Perelman School of Medicine, University of Pennsylvania, Philadelphia, PA, USA; ^2^University of Pennsylvania, Philadelphia, PA, USA; ^3^Columbia University Mailman School of Public Health, New York, NY, USA; ^4^Verily Life Sciences, San Francisco, CA, USA; ^5^Palliative and Advanced Illness Research Center, University of Pennsylvania, Philadelphia, USA; ^6^Penn Predictive HealthCare Team, University of Pennsylvania Health System, Philadelphia, PA, USA; ^7^Temple Center for Population Health, Temple University Health System, Philadelphia, PA, USA; ^8^Northwestern University, Chicago, IL, USA

###### **Correspondence:** Samuel Takvorian (samuel.takvorian@pennmedicine.upenn.edu)

*Implementation Science 2024*, **19(2):**S63

**Background:**

Serious illness conversations (SICs) allow patients to share their goals and care preferences and are an evidence-based practice that improves quality of life and reduces healthcare utilization. Despite national guidelines recommending early SICs, most patients with cancer die without a documented SIC. This study (NCT04867850) tested the effects of patient- and clinician-directed implementation strategies informed by behavioral economics (“nudges”) on SIC completion.

**Methods:**

In this 2x2 factorial cluster-randomized trial, clinicians and their high-risk patients were independently randomized to receive nudges or not, resulting in four arms: active control, clinician nudge, patient nudge, or both nudges. In the active control, clinicians received messages identifying patients with high 180-day mortality risk, as predicted by a validated machine learning algorithm. Clinician nudges added performance reports comparing clinicians’ SIC rates to those of their peers. Patient nudges included a brief electronic questionnaire priming patients for SICs before appointments. The primary outcome was SIC documentation within six months of enrollment. Secondary outcomes included palliative care referral and a composite measure of aggressive end-of-life care. Time-to-event analyses were performed using a Cox proportional hazards model with cluster robust standard errors.

**Findings:**

Between September 2021 and March 2022, 4,450 patients (median age 67 years, 52.9% female, 17.3% Black) seen by 166 clinicians were randomized. Patient-level SIC completion rates were highest in the both nudges arm (14.1%), followed by the clinician nudge (11.5%), patient nudge (11.5%), and active control (11.2%). In adjusted analyses, patients in the combined nudges arm were significantly more likely to have SICs than those in the active control (ratio of hazard ratios: 1.55, 95% confidence interval: 1.00-2.40; p=0.049). Interventions did not impact palliative care referral rates or receipt of aggressive end-of-life care. Ongoing patient and clinician interviews will uncover implementation strategy mechanisms and contextual factors influencing SIC completion.

**Implications for D&I Research:**

Clinician- and patient-directed nudges synergistically promoted SICs. However, given low overall SIC rates, there may be a ceiling effect of priming alone. Multilevel implementation strategies supplementing patient- and clinician-level prompts, like those in the Expert Recommendations for Implementing Change (ERIC) taxonomy, or utilizing trained non-physician professionals to initiate conversations, may be necessary to achieve sustained behavior change.

**Primary Funding Source:** National Institutes of Health

### S64 Implementation evaluation of a digital adverse drug event reporting platform in British Columbia, Canada

#### Yuen Yan^1^, Serena Small^2^, Amber Cragg^2^, Kate Butcher^3^, Corinne Hohl^2^

##### ^1^University of British Columbia, Vancouver, BC, Canada; ^2^University of British Columbia, Vancouver, Canada; ^3^Vancouver General Hospital, Vancouver, Canada

###### **Correspondence:** Yuen Yan (erica.lau@ubc.ca)

*Implementation Science 2024*, **19(2):**S64

**Background:** Over 2 million Canadians visit emergency departments annually because of an adverse drug event (ADE). These unintended, harmful medication-related incidents often recur because of communication gaps between healthcare providers. ActionADE is a web-based application designed to bridge this gap. ActionADE provides a user-friendly data entry interface for hospital providers to document standardized ADE information and communicates the information to community pharmacy systems via the provincial medication dispensing system (PharmaNet). The objectives of this study were to describe the implementation strategies employed during the first two years of ActionADE's deployment and explore their relationships with providers' use of ActionADE.

**Methods:** We used a prospective observational design and a mixed methods approach. We analyzed implementation data from six BC hospitals, collected between August 2020 and December 2022. Two members of the research team logged implementation activities during the study period and classified them into strategies as per the framework proposed by Powell et al. We retrieved data on ADEs documented by providers from the ActionADE server on a monthly basis during study period. We explored patterns of relationships by triangulating implementation strategies with the observed trends in ADE reporting.

**Findings:** Throughout the two-year implementation period, we identified 817 implementation activities, which were further classified into 11 implementation strategies: technical assistance, audit and feedback, clinician reminder, training, implementation team meeting, educational meeting, advisory board and workshop, incentives, mass media, educational material, and patient involvement. Hospital clinicians documented 3,458 ADEs over the study period. Data triangulation demonstrated a distinct pattern that the use of a bundle of strategies, including incentives, patient involvement, clinician reminders, and implementation team meetings substantially increased providers' use of ActionADE.

**Implications for D&I Research:** The findings of our study identified a suite of strategies that played a crucial role in promoting providers’ use of ActionADE. Future research should test and explore how these strategies can be adapted to different healthcare environments and other technologies to provide insights for scaling digital health interventions.

**Primary Funding Source:** Canadian Institute of Health Research

### S65 Conducting pragmatic-clinical trials in nursing homes: Lessons learned and recommendations for future implementation research

#### Natalie Leland^1^, Carin Wong^1^, Victoria Shier^2,3^, Dominique Como^1^, Cara Lekovitch^1^, Catherine Piersol^4^, Felicia Chew^4^

##### ^1^University of Pittsburgh, Pittsburgh, PA, USA; ^2^University of Southern California, Los Angeles, CA, USA; ^3^USC Schaeffer Center for Health Policy & Economics, Los Angeles, CA, USA; ^4^Thomas Jefferson University, Philadelphia, PA, USA

###### **Correspondence:** Natalie Leland (nel24@pitt.edu)

*Implementation Science 2024*, **19(2):**S65

**Background:** In 2022 the National Academies of Science, Engineering, and Medicine released a report detailing a need to improve nursing home (NH) quality, which included staff training and implementation of high quality, equitable care. In response, this presentation will highlight lessons learned from a large-scale pragmatic trial conducted in NHs across the US and provide recommendations for proactively planning for multi-level factors that impact the success of NH implementation research.

**Methods:** This cluster randomized comparative effectiveness study compared two non-pharmacological approaches to dementia care (i.e., team-based approach and problem-based approach) using a convergent mixed methods design. The study was guided by Donabedian’s Model of Healthcare Quality, the Health Equity Implementation Framework, and an actively engaged Advisory Committee.

Eighty NHs were randomized to one-of-the-two treatment approaches in which staff were trained on their respective dementia care approaches. Data collection included the NH Minimum Data Set, electronic medical records, and interviews with NH staff and families of residents living with dementia. Leveraging our mixed methods approach, we have synthesized lessons learned, which can guide future NH implementation.

**Findings:** Two overarching themes emerged with respect to identifying and accounting for the multi-level factors that impact US NH implementation research. First, a multi-level implementation framework was pivotal to our study design. During study development, we accounted for factors directly impacting the two comparators, including societal and contextual influences (e.g., federal- and state-level NH training regulations, inequities in NH dementia care access) as well as the clinical encounter (e.g., leveraging staff workflow, mandated NH documentation). Yet, our planning did not account for societal and contextual influences that were peripheral to our approaches (e.g., new federal and state policies, NH leadership and ownership changes, and a global pandemic). Second, purposeful, transparent, and meaningful community-engagement was essential. Our Advisory Committee provided key insights into the multi-level factors that had to be integrated into the study design. They also helped us navigate unanticipated barriers that emerged throughout the study.

**Implications for D&I Research:** The design of NH implementation research should consider factors directly related to the intervention as well as peripheral factors at the societal, contextual, and encounter level.

**Primary Funding Source:** Patient-Centered Outcomes Research Institute

### S66 Mentored quality improvement initiative reduces nursing home hospitalizations during COVID-19 pandemic

#### Sunil Kripalani, Yaping Shi, Isaac Schlotterbeck, Jonathan Schildcrout, Mattie Brady, Carole Bartoo, Anna Gallion, April Hanlotxomphou, Elizabeth Coughlin, Kristina Niehoff, Tara Horr, Victor Legner, Sandra Simmons

##### Vanderbilt University Medical Center, Nashville, TN, USA

###### **Correspondence:** Sunil Kripalani (sunil.kripalani@vanderbilt.edu)

*Implementation Science 2024*, **19(2):**S66

**Background:** Nursing home residents are particularly vulnerable to COVID-19 infection and complications, due to high transmission in congregated living facilities and greater susceptibility secondary to advanced age and underlying medical conditions. The Quality Improvement Collaborative for COVID-19 Prevention and Control in Middle Tennessee Nursing Homes was designed to provide quality improvement training and mentored implementation support to prevent infections, hospitalizations, morbidity, and mortality due to COVID-19 and associated conditions.

**Methods:** From August 2021 to March 2023, our multidisciplinary team partnered with 51 participating nursing homes to 1) conduct a structured needs assessment, 2) identify quality improvement priority areas, 3) provide mentored support to implement Plan-Do-Study-Act (PDSA) cycles to improve quality of care in targeted areas, and 4) evaluate impact with a primary outcome of hospitalization rates. We utilized the Centers for Medicare and Medicaid Services’ (CMS) COVID-19 Nursing Home Dataset to compare weekly COVID-19 hospital admissions per 1,000 residents between participating and non-participating sites from the same counties, adjusting for facility characteristics. We also evaluated barriers and facilitators to implementation and engagement using the Consolidated Framework for Implementation Research (CFIR) through staff interviews with participating nursing homes.

**Findings:** Nursing homes that participated in the collaborative had significantly lower cumulative hospitalization rates during the intervention period compared to non-participating homes (N=111 vs. 246 admissions per 1,000 residents; cumulative difference=135, 95% confidence interval 37 to 239). Additionally, participating nursing homes maintained consistently higher occupancy rates compared to non-participating homes. The CFIR constructs that most strongly distinguished between high and low engagement sites include external policy and incentives, culture, and champions. For example, nursing homes in the high engagement group tended to view mandated quality metrics and data reporting more positively compared to low engagement sites. Similarly, nursing homes in the high engagement group typically had a more collaborative culture, internal motivation, and strong champions.

**Implications for D&I Research:** This large, mentored quality improvement collaborative significantly reduced hospitalization rates of nursing home residents during the pandemic. Our evaluation of barriers and facilitators provides insight for future nursing home quality initiatives.

**Primary Funding Source:** Centers for Medicare and Medicaid Services

### S67 Diffusion of excellence: Evaluating a system to identify, replicate, and spread promising innovative practices across the veterans health administration

#### George Jackson^1,2^, Gemmae Fix^3,4^, Brandolyn White^5^, Sarah Cutrona^3,6^, Caitlin Reardon^7^, Laura Damschroder^7^, Madison Burns^5^, Kathryn DeLaughter^3^, Marilla Opra Widerquist^7^, Maria Arasim^7^, Allen Gifford^4,8^, Heather King^5,9^, Jenesse Kaitz^3^, Guneet Jasuja^3,4^, Timothy Hogan^3,10^, Jaifred Christian Lopez^5,9^, Blake Henderson^11^, Blaine Fitzgerald^11^, Amber Goetschius^11^, Danielle Hagan^11^, Carl McCoy^11^, Alex Seelig^12^, Andrea Nevedal^7^

##### ^1^Durham Veterans Affairs (VA) Health Care System, Durham, NC, USA; ^2^University of Texas Southwestern Medical Center, Dallas, TX, USA; ^3^VA Bedford Healthcare System, Bedford, MA, USA; ^4^Boston University, Boston, MA, USA; ^5^Durham VA Health Care System, Durham, NC, USA; ^6^University of Massachusetts Chan Medical School, Worcester, MA, USA; ^7^VA Ann Arbor Healthcare System, Ann Arbor, MI, USA; ^8^VA Boston Healthcare System, Center for Healthcare Organization and Implementation Research (CHOIR), Boston, MA, USA; ^9^Duke University, Durham, NC, USA; ^10^Peter O'Donnell Jr. School of Public Health, UT Southwestern Medical Center, Dallas, TX, USA; ^11^VHA Innovation Ecosystem, Washington, DC, USA; ^12^Agile Six Applications, Inc., San Diego, CA, USA

###### **Correspondence:** George Jackson (george.jackson@utsouthwestern.edu)

*Implementation Science 2024*, **19(2):**S67

**Link:** https://www.frontiersin.org/articles/10.3389/frhs.2023.1223277/full

### S68 Implementation facilitation efforts on intimate partner violence screening and subsequent psychosocial service utilization

#### Kelly Stolzmann^1^, Christopher Miller^2^, Julianne Brady^1^, Omonyele Adjognon^1^, Melissa Dichter^3^, Katherine Iverson^4^

##### ^1^Veterans Health Administration, VA Boston Healthcare System, Boston, MA, USA; ^2^Harvard Medical School, Boston, MA, USA; ^3^Veterans Health Administration, Department of Veterans Affairs, Center for Health Equity Research and Promotion, Philadelphia, Pennsylvania & Department of Family Medicine and Community Health, University of Pennsylvania Perelman School of Medicine, Philadelphia, PA, USA; ^4^Women's Health Sciences Division, National Center for PTSD, Veterans Health Administration, Boston, MA, USA

###### **Correspondence:** Kelly Stolzmann (kelly.stolzmann@va.gov)

*Implementation Science 2024*, **19(2):**S68

**Background:** Intimate partner violence (IPV) against women is a population health issue. Screening increases IPV detection and, when paired with appropriate interventions, can mitigate IPV’s health effects. The Veterans Health Administration (VHA) initiated implementation facilitation (IF) to roll out IPV screening programs in primary care. IF consists of personalized, interactive support that can include trainings, education, and technical assistance. VHA recommends that women primary care patients are screened annually. This study examines IPV screening rates and post-screening psychosocial service use at sites receiving IF.

**Methods:** A cluster randomized, stepped wedge, Hybrid Type II trial was used in this study. IF occurred in two waves across nine sites. We examined rates of IPV screening and post-screening psychosocial service use among women during the implementation phase (9 months) and the maintenance phase (12 months post-IF). We used medical records to identify IPV screening and psychosocial service utilization (e.g., social work and psychology) in the 60 days post-screening. Generalized linear mixed models with site as a random effect were used to predict IPV screening and psychosocial service use. An Institutional Review Board approved this study.

**Findings:** Women in the IF period were 2.57 times more likely to be screened (95% CI: 2.33-2.85) and women in the sustainment period 2.38 times more likely to be screened (95% CI: 2.12-2.67) compared to the pre-IF period. Women screened in the IF period were 1.29 times more likely to receive psychosocial services (95% CI: 1.07-1.56) and women screened in the maintenance period were 1.31 times more likely to receive psychosocial services (95% CI: 1.06-1.62) within 60-days post screen compared to the pre-IF period, after adjusting for pre-screening psychosocial service use.

**Implications for D&I Research:** IF was associated with an increase in IPV screening, which was sustained up to one year after IF. Importantly, IF was associated with an increase in psychosocial service use among those screened at post-IF and the maintenance period. Increased psychosocial service use among women screened for IPV provides initial evidence that IF results in IPV screening programs being implemented with fidelity (i.e., women who may have experienced IPV are being effectively connected with potentially life-saving follow-up interventions).

**Primary Funding Source:** Department of Veterans Affairs

### S69 Using the matrixed multiple case study approach to identify factors impacting IPV screening implementation success in primary care clinics

#### Julianne Brady^1^, Omonyele Adjognon^1^, Kelly Stolzmann^1^, Megan Gerber^1^, Katherine Iverson^2^, Robert Lew^3,4^, Melissa Dichter^5^, Galina Portnoy^6^, Samina Iqbal^7^, Sally Haskell^8^, LeAnn Bruce^9^, Christopher Miller^10^

##### ^1^Veterans Health Administration, VA Boston Healthcare System, Boston, MA, USA; ^2^Women's Health Sciences Division, National Center for PTSD, Veterans Health Administration, Boston, MA, USA; ^3^Boston University School of Medicine, Boston, MA, USA; ^4^Maveric Research, Veterans Health Administration, Jamaica Plain, MA, USA; ^5^Temple University, Philadelphia, PA, USA; ^6^Yale School of Medicine, New Haven, CT, USA; ^7^VA Palo Alto Healthcare System Palo Alto, CA, USA; ^8^VA Connecticut Healthcare System, West Haven, CT, USA; ^9^Veterans Health Administration, Washington, DC, USA; ^10^Harvard Medical School, Boston, MA, USA

###### **Correspondence:** Julianne Brady (julianne.brady@va.gov)

*Implementation Science 2024*, **19(2):**S69

**Link**: https://implementationsciencecomms.biomedcentral.com/articles/10.1186/s43058-023-00528-x

### S70 Conducting a hybrid-type I randomized, stepped-wedge trial while navigating the syndemic of COVID-19 and a global positive airway pressure (PAP) recall – interim results from addressing sleep apnea post-stroke/TIA (ASAP)

#### Jason Sico^1^, Laura Burrone^2^, Ali Sexson^3^, Kristin Story^3^, Nick Rattray^3^, Dawn Bravata^3^, Tony Perkins^3^, Laura Myers^3^, Edward Miech^4^, Stanley Taylor^5^, Joanne Daggy^6^, Brian Koo^7^

##### ^1^Veterans Health Administration, Headache Centers of Excellence (HCoE), Orange, CT, USA; ^2^VHA Headache Centers of Excellence Research and Evaluation Center, VA Connecticut Healthcare System, Orange, CT, USA; ^3^Veterans Health Administration, Indianapolis, IN, USA; ^4^VHA HSR&D Center for Health Information and Communication (CHIC) and Quality Enhancement Research Initiative Expanding expertise Through E-health Network Development (EXTEND) QUERI Centers, Roudebush VA Medical Center, Indianapolis, IN, USA; ^5^Richard L. Roudebush VAMC, Indianapolis, IN, USA; ^6^Indiana University School of Medicine & Fairbanks School of Public Health, Indianapolis, IN, USA ^7^Veterans Health Administration, VA Connecticut Healthcare System, West Haven, CT, USA

###### **Correspondence:** Jason Sico (jason.sico@yale.edu)

*Implementation Science 2024*, **19(2):**S70

**Background:** Current cerebrovascular guidelines recommend evaluating ischemic stroke/transient ischemic attack (TIA) patients for obstructive sleep apnea (OSA). Despite evidence favoring treatment soon after a cerebrovascular event, OSA testing rates remain low.

**Methods:** Addressing Sleep Apnea Post-Stroke/TIA (ASAP; NCT04322162) is a randomized, stepped-wedge trial of six diverse VA Medical Centers (VAMCs) designed to increase the rate of timely, guideline-concordant diagnosis and treatment of OSA among Veterans with ischemic stroke/TIA. Active implementation consists of three, 7-month data periods, during which VAMCs create an acute OSA testing and positive-airway pressure (PAP) treatment service (i.e., ASAP Program) by conducting quality improvement initiatives and incorporating a systems redesign Virtual Collaborative and data monitoring. Implementation strategies include external facilitation, local adaptation, and audit and feedback. ASAP has as its primary implementation outcome the Group Organization (GO) Score for providing care, grouped as follows: No Facility-Wide Approach (1-3), Cross-Service Approach (4-5), Facility-Wide Approach Emerging (6-7), Facility-Wide Approach Established (8-10). The GO Scores and select Consolidated Framework for Implementation Research (CFIR) construct valence and magnitude were determined at baseline and at the end of each data period by the investigative team through blinded, individual scoring based on interviews and prospective evaluation updates/reports. ASAP primary effectiveness outcome is 30-day facility-level OSA testing rate. ASAP was conducted entirely during the COVID-19 pandemic, and later, a global recall of PAP devices.

**Findings:** At baseline, no ASAP Program existed at any VAMC (GO Score of 1). End of active implementation GO Scores for 4 sites were 4, 7, 8, and 9. VAMCs obtaining higher GO Scores also had CFIR scores reflecting engaged champions which developed networks and communications and reflected and evaluated on the performance data. External policies and incentives (e.g., limitations in testing patients if OSA treatment not available) hindered ASAP development at all VAMCs, requiring tailored external facilitation to address challenges from the COVID-19 pandemic and the global PAP recall.

**Implications for D&I Research:** Comprehensive, facility-wide ASAP Programs can effectively be developed despite pronounced, negative external factors, when strong champions emerge who create and strengthen networks while reflecting and evaluating on data and embracing a culture of continuous quality improvement.

**Primary Funding Source:** Department of Veterans Affairs

### S71 Measuring sustainability in patient navigation programs: Adaptation of the program sustainability assessment tool (PSAT)

#### Elsa Staples^1^, Andrea Dwyer^2^, Sara Malone^3^, Doug Luke^4^, Kim Prewitt^4^, Caren Bacon^5^, Betsy Risendal^1^

##### ^1^Colorado School of Public Health, Aurora, CO, USA; ^2^University of Colorado Cancer Center, Aurora, CO, USA; ^3^Department of Surgery (Division of Public Health Sciences) and Alvin J. Siteman Cancer Center, Washington University School of Medicine, St. Louis, MO, USA; ^4^Washington University in St. Louis, St. Louis, MO, USA; ^5^Washington University in St Lois, St Lois, MO, USA

###### **Correspondence:** Elsa Staples (elsa.staples@cuanschutz.edu)

*Implementation Science 2024*, **19(2):**S71

**Background:** Patient navigation is an evidence-based approach for reducing health disparities in multiple health conditions. Sustaining patient navigation (PN) programs beyond grant funding and building capacity for institutional support is a critical issue facing clinic systems, especially those reaching persons with limited access to care. Strategies to improve sustainability must be targeted to be effective.

**Methods:** The Program Sustainability Assessment Tool (PSAT) and Clinical Sustainability Assessment Tool (CSAT), well-established self-assessments developed at Washington University for use by public health and clinical programs, were adapted by the Colorado Cancer Screening Program (CCSP) to select priority domains and activities to sustain PN. Version 1 of the modified Patient Navigation Sustainability Assessment Tool (PNSAT) contained up to 40 questions in 8 domains from the PSAT relating to engagement/capacity of the organization, staff, and community, funding stability, communication and planning, workflow integration, and evaluation and outcomes. Based on the responses of the PNSAT Version 1 (n=111), the tool was modified and shortened in 2023 using Cronbach’s alpha and feedback from respondents in consultation with the PSAT/CSAT tool developers. The resulting PNSAT Version 2 included all 8 original domains and was piloted by CCSP with 10 clinic systems statewide reaching rural and medically underserved populations.

**Findings:** The shortened tool was completed by 1-4 respondents per site and required 15-20 minutes to complete. It contains 4 items in each domain (vs. 6-8 in Version 1). Internal consistency of the long and shortened versions of the PNSAT indicated that the shortened version was similarly reliable, with PNSAT Version 1 Cronbach alpha scores ranging from 0.88-0.94 and PNSAT version 2 ranging from 0.85-0.93. Average scores of PNSAT v. 2 ranged from 3.9-6.6 (on a 1-7 scale, with 7 being highest capacity). The domains most frequently targeted for improvement by clinics were Workflow Integration and Communication, Planning, and Implementation, based on both domain scores and actionability.

**Implications for D&I Research:** PN is a unique intervention that addresses both clinic and community factors, requiring adapted approaches reflecting both settings to address sustainability. Modification of an existing tool resulted in a robust method useful in improving key areas for future programmatic activities.

**Primary Funding Source:** Colorado Cancer, Cardiovascular and Pulmonary Disease Fund

### S72 Strategies to de-implement low-value pre-operative testing: The use of a research-practice partnership as a model to design & deliver high-value care

#### Anthony Cuttitta^1^, Valerie Gavrila^2^, Caroline Richburg^2^, Cecilia Pesavento^2^, Alexis Antunez^3^, Shawna Smith^4^, Lesly Dossett^2^

##### ^1^University of Michigan-Health, Ann Arbor, MI, USA; ^2^University of Michigan, Ann Arbor, MI, USA; ^3^Brigham and Women's Hospital, Boston, MA, USA; ^4^University of Michigan School of Public Health, Ann Arbor, MI, USA

###### **Correspondence:** Anthony Cuttitta (cuttitta@med.umich.edu)

*Implementation Science 2024*, **19(2):**S72

**Background:** De-implementation of low-value care represents an opportunity to reduce patient burden, re-purpose limited clinician time, and increase healthcare system efficiency. This is particularly relevant to pre-operative care, where testing utilization is variable and overuse is common despite a strong evidence base to avoid it.

Research-practice partnerships that integrate clinical care interventions with rigorous research methods & implementation science frameworks present an opportunity to advance dissemination & implementation through using these methods to *design* strategic, multi- quality improvement interventions.

**Methods:** Multiple methods informed intervention *design.* Common pre-operative tests (CBCs, BMPs/CMPs, EKGs) and low-risk surgeries (lumpectomy, inguinal hernia repair, laparoscopic cholecystectomy) were identified as intervention candidates based on clinician input. Claims data was reviewed to determine testing variability & potential overuse. An ethnographic study engaged 30 clinicians to learn about testing practices & perceptions. A pre-intervention chart review of testing appropriateness prior to target low-risk surgeries was conducted. Lastly, the intervention was designed based on these learnings, identification of Tailored Implementation in Chronic Diseases (TICD) factors, and implementation mapping to align strategies with barriers. Intervention *evaluation & impact* will be assessed through post-intervention chart review and claims analysis which will be complete by November 2023.

**Findings:** Analyses of claims data & clinician input confirmed overuse of low-value pre-operative testing before the three target procedures. The ethnographic study identified three themes: 1) Shared Values of patient safety and evidence-based care (TICD Social, Political, and Legal Factors), 2) Gaps in Knowledge (TICD Individual Health Professional Factors, Guideline Factors), and 3) Communication Breakdown (TICD Professional Interactions, Incentives and Resources, Capacity for Organizational Change). The chart review found a large proportion of pre-operative tests to be low-value: 69% of CBCs, 59% of BMP/CMPs, and 43% of EKGs were unnecessary. The comprehensive intervention design based on learnings & data led to a multi-component intervention, including: detailed testing guidelines, structured provider engagement & education, and coordination across clinicians/stakeholders.

**Implications for D&I Research:** This approach is relevant to the science of dissemination & implementation by demonstrating how research-practice partnerships can utilize research methods to inform a local quality improvement effort *design* as well as developing learnings which can inform broader dissemination and spread opportunities.

### S73 Implementing special initiative funding to improve women’s health services: The crucial role of facility leaders and office of women’s health resources

#### Kimberly Clair^1^, Danielle Dunn^1^, Tanya Olmos-Ochoa^1^, Kristina Cordasco^1^, Shay Cannedy^1^, Agatha Palma^1^, Alicia Gable^1^, Melissa Farmer^2^, Erin Finley^3^, Chelsea Cosby^4^

##### ^1^Veterans Health Administration, Department of Veterans Affairs, Los Angeles, CA, USA; ^2^Veterans Health Administration, VA Greater Los Angeles Healthcare System, Center for the Study of Healthcare Innovation, Implementation & Policy (CSHIIP) Los Angeles, USA; ^3^University of Texas Health San Antonio, San Antonio, USA; ^4^District of Columbia, MD, USA

###### **Correspondence:** Kimberly Clair (kimberly.clair@va.gov)

*Implementation Science 2024*, **19(2):**S73

**Background:** The Women’s Health Innovations and Staffing Enhancements (WHISE) Initiative, launched in 2021, provides funding to improve Women’s Health programs at Veterans Administration (VA) facilities through staffing, equipment, and program development. To inform VA’s Office of Women’s Health’s (OWH) implementation of WHISE, we characterized challenges encountered by the frontline personnel—Women Veteran Program Managers (WVPMs)—responsible for implementing WHISE at their local facilities. We also examined facilitators to overcoming these challenges in relation to WVPMs’ tenure in VA employment.

**Methods:** We conducted semi-structured interviews (November 2022 – February 2023) with WVPMs across 37 VA facilities. Facilities were randomly selected within strata based on VA regions, size of women Veteran enrollment, and facility model (arrangements) for WH care, with an oversample of rural facilities. Interviews were summarized using a template; team-based content analysis using matrices was performed.

**Findings:** The primary challenges WVPMs faced were developing and managing new relationships with facility leaders and staff (e.g., communicating across service lines) and having knowledge gaps around hiring and purchasing processes. These challenges were encountered by WVPMs regardless of tenure in employment. To overcome these challenges, WVPMs relied on supportive relationships, particularly from facility leaders and other WVPMs, and OWH resources (e.g., position descriptions) and weekly information sessions. While WVPMs new to VA employment (< 3 years) generally relied on facility leadership for hands-on training, more tenured WVPMs were more likely to leverage pre-existing facility-based relationships to address knowledge deficits. Both new and more tenured WVPMs relied on facility leaders to develop interdisciplinary relationships and intervene with communication delays across service lines.

**Implications for D&I Research:** Facility leaders and OWH resources provided crucial support for both new and more tenured WVPMs in overcoming WHISE implementation challenges. However, our findings indicate that, when tasked with implementing system-level improvement initiatives, WVPMs and other frontline personnel may require varying levels of implementation support based on length of tenure in their employment. Investing in mechanisms to strengthen facility leaders’ capacities for mentorship, and augmenting implementation resources (e.g., guidebooks, tools), will enhance the implementation effectiveness of both new and more tenured frontline personnel. Organizations should identify variations in workforce support needs to optimize implementation of system-level interventions.

**Primary Funding Source:** Department of Veterans Affairs

### S74 Implementation modifications to support injectable HIV pre-exposure prophylaxis into standard of care in the United States: FRAME-IS results from the PrEP implementation study for cabotegravir long acting for men in the real world (PILLAR)

#### Nanlesta Pilgrim^1^, Sydney Perlotto^2^, Peter Shalit^3^, Mitchell Witehead^4^, Taimur Khan^5^, Olga Boiko^6^, Nicola Barnes^6^, Deanna Merrill^1^, Dina Odrobina^1^, Todd McKeon^1^, Heidi Swygard^1^, Maggie Czarnogorski^1^

##### ^1^ViiV Healthcare, Durham, NC, USA; ^2^Yale School of Public Health, New Haven, CT, USA; ^3^Tribalmed, Seattle, WA, USA; ^4^AHF Healthcare Center, Pensacola, FL, USA; ^5^Fenway Health, Boston, MA, USA; ^6^Evidera, London, United Kingdom

###### **Correspondence:** Nanlesta Pilgrim (nanlesta.a.pilgrim@viivhealthcare.com)

*Implementation Science 2024*, **19(2):**S74

**Background:** Black and Latino men who have sex with men (MSM) and transgender men (TGM) experience a higher lifetime risk for HIV infection than the general male population. Yet they are significantly less likely to access and use pre-exposure prophylaxis (PrEP) for HIV. With the availability of long-acting injectable PrEP, APRETUDE, consideration should be given to implementation that reduces inequitable access. The PILLAR study assesses implementation strategies for integrating APRETUDE into standard of care for MSM and TGM. Here, we report implementation modifications over 1 year.

**Methods:** From June 2022-June 2023, PILLAR employed the FRAME-IS to systematically document monthly modifications to APRETUDE implementation from up to 86 staff study participants (SSPs) across 17 clinics. Clinic activation was staggered. SSPs completed the FRAME-IS starting in month 2 following clinic activation; the average completion rate was 79%. Questions aligned with core FRAME-IS modules (1-4) and two optional modules (5-6). Modifications and their rationales were coded, themes identified, and results mapped onto the CFIR and Proctor frameworks.

**Findings:** Fifty-eight modifications were reported; 70.6% of clinics reported at least one modification. Most modifications related to clinic operations or education, specifically staffing changes (20.7%, n=12), provider/patient education (15.5%, n=9), clinic system changes (13.8%, n=8), and screening/enrollment procedures (13.8%, n=8) (Modules 1-3). Modification reasons sought to improve patient flow management, make staffing changes, and provide continuity; overarching modification goals focused on improving fit with the clinical/practice setting and speeding up the injection/implementation process (Module 4, Part 1). Examples included managing staff workload, developing APRETUDE-specific protocols, updating office procedures, and improving appointment scheduling. Most modifications were aimed at the patient and clinic/organizational levels (Module 4, Part 2). Modifications occurred at all implementation phases and in response to an encountered barrier (Module 5, Parts 1-2). Clinic leaders/administrators, PrEP providers, and nurses were the most identified decision-makers (Module 6). Mapped onto the CFIR and Proctor frameworks, modifications focused on the inner setting structural characteristics and resources and impacted implementation feasibility, respectively.

**Implications for D&I Research:** Using the FRAME-IS, we identified important clinic modifications needed for the effective implementation of a novel prevention product into care, which is essential for sustainability and equitable access for key populations.

**Primary Funding Source:** ViiV Healthcare

### S75 Partnerships to empower: Using the implementation and sustainability infrastructure domain of PRISM to assess the role of engagement and relationships

#### Sarah Koopman Gonzalez

##### Case Western Reserve University, Cleveland, OH, USA

###### **Correspondence:** Sarah Koopman Gonzalez (sjk98@case.edu)

*Implementation Science 2024*, **19(2):**S75

**Background:**

EMPOWER partners with a network of over 100 primary care practices of an integrated health care system to improve diagnosis and care for urinary incontinence (UI) among women. Partnership with stakeholders is essential for adoption and sustainability. We used the Implementation and Sustainability Infrastructure domain of the Practical Robust Implementation and Sustainability Model (PRISM) to understand the role of partnership on Reach, Effectiveness, Adoption, Implementation, and Maintenance (RE-AIM) outcomes.

**Methods:**

A multi-level, multi-methods approach was used to assess provider education on UI care management, implementation of UI screening in the primary care setting, and empowering women through education and interactions with a nurse navigator and a digital health chat bot. The RE-AIM model and PRISM were used to guide the evaluation using the assessment of an engagement log maintained by the implementation team, tracking screenings, dissemination tracking, field notes, and monthly surveys and interviews with clinic stakeholders.

**Findings:**

Engagement with the system as a whole included: (1) meetings, presentations, and emails with leadership, regional and practice medical directors, office managers, and frontline staff, (2) identifying a system liaison, and (3) in-person visits with all staff at clinics for training, support, and rapport building. Direct patient engagement strategies included UI screening tools and educational materials in clinic, encouragement to address symptoms, additional interventions, a newsletter, and news media. Qualitative methods revealed positive comments from stakeholders about these methods. Partnership efforts resulted in 26 enrolled clinics and over 3500 completed screeners in the first six months. Only two clinics completed no screeners. Practices were offered a menu of sustainability options such as a screening template, EMR integration planning, continence advisor training, educational resources for patients, and tools to aid providers with UI diagnosis and management. Engaging clinics around sustainability is ongoing. Although there was regular communication, barriers were noted for both implementation and sustainability, such as misunderstandings about screening administration. Lessons learned are being applied to the project as we move forward.

**Implications for D&I Research:**

This study demonstrates use of the PRISM framework for understanding the approaches to multi-level partnership and how assessment of this throughout the project can lead to sustainment of implementation outcomes.

**Primary Funding Source:** Agency for Healthcare Research and Quality

### S76 Streamlining practice facilitation and local partnerships: Early results from EvidenceNOW MUI in Wisconsin primary care

#### Edmond Ramly

##### Indiana University, Bloomington, IN, USA

###### **Correspondence:** Edmond Ramly (eramly@iu.edu)

*Implementation Science 2024*, **19(2):**S76

**Background:** Wisconsin’s EvidenceNOW project for Managing Urinary Incontinence (WI-INTUIT) is an ongoing hybrid trial for bridging community-based continence promotion and primary care practices. By streamlining implementation strategies, we aim to integrate sustainable interventions into local contexts with minimal practice burden. We sought to specify the core elements of streamlined practice facilitation and partnership building based on early implementation results.

**Methods:** We enrolled 32 Wisconsin practices, randomized to two implementation strategy arms, one receiving streamlined practice facilitation and the other additionally receiving local partnership building. These multi-level strategies aim to address the needs of overburdened primary care practices building on previous implementation research.

Following the Consensus on Relevant Elements (CORE) process guided by the Expert Recommendations for Implementing Change (ERIC), we specified the details of streamlined practice facilitation and local partnership building as implementation strategies. We iteratively refined and operationalized the strategies through practice team meetings, multidisciplinary debriefs, and guided reflections. The configurations of core functions that comprise the strategies were determined through rapid qualitative analyses of these interactions.

**Findings:** We specified 16 and 5 core functions of streamlined practice facilitation and local partnership building respectively. Streamlined practice facilitation moved practice change teams through graduated autonomy in stages: 31 practices completed facilitated assessments of local context, 14 practices collaborated with facilitators to plan interventions tailored to local needs, and 5 practices launched implementation with only targeted facilitator support. Each stage involved a 45-60 minute meeting. Practices gradually took ownership of implementation planning, with 9 site champions constructing and executing detailed launch plans for their local implementations. Each of the 14 practices that completed planning received information on at least three local resources in their community, and practices assigned to local partnership building are currently selecting the resources to form local partnerships with.

**Implications for D&I Research:** Streamlining facilitation and local partnership building appears to be a feasible and low burden way to adapt implementations to local needs across non-integrated geographically diverse primary care practices. Processes that limit the effort required from overburdened sites and support them with local partnerships may enhance sustainability and scalability of facilitated implementation.

**Primary Funding Source:** Agency for Healthcare Research and Quality

### S77 Improving primary care understanding of resources and screening for urinary incontinence to enhance treatment (PURSUIT) for women veterans in the Southeast within the VA health care system

#### Alayne Markland

##### Veterans Health Administration, Birmingham VA Health Care System and University of Alabama at Birmingham, Birmingham, AL, USA

###### **Correspondence:** Alayne Markland (markland@uab.edu)

*Implementation Science 2024*, **19(2):**S77

**Background:**

Through the AHRQ’s EvidenceNow: Managing Urinary Incontinence (UI) Initiative, the PURSUIT team aims to improve access to evidence-based nonsurgical UI treatment for women Veterans in the Department of Veterans Affairs Integrated Service Network (VISN 7). Guided by the Reach, Effectiveness, Adoption, Implementation, and Maintenance (RE-AIM) framework, we will compare two models at the practice level: (1) using a practice facilitation toolkit with our Mobile health or mHealth UI treatment modality (MyHealtheBladder) alone and (2) the practice facilitation toolkit with MyHealtheBladder combined with clinical care pathways.

**Methods:**

PURSUIT will target 62 practices with over 50,000 women Veterans across the three states included in VISN 7 (Alabama, Georgia, and South Carolina). All primary care practices will receive practice facilitation including (1) a virtual or on-site visit, along with 1-3 practice facilitator visits engaging practices and site champions; (2) MyHealtheBladder training; and 3) Online Toolkit, including a clinical dashboard that provides the site champion information about clinic-assigned women Veterans at high risk of UI. Those practices randomized to MyHealtheBladder combined with clinical care pathways will also receive education on telehealth referrals for evaluation with a remote continence specialist. Additionally, once a practice is enrolled, assigned women are eligible for direct-to-patient outreach from the PURSUIT team either through a mailed flyer or a secure email through MyHealtheVet, the VA’s patient messaging portal.

**Findings:**

The PURSUIT team has strong relationships with local and regional leadership in women’s health, experts in informatics, industry, and VA information technology infrastructure. To date, we have approached 24 clinics and completed 17 visits across Alabama and Georgia using our practice facilitation approach. From the practices, we sent 3350 emails through MyHealtheVet and 2,800 mailed flyers. From this recruitment strategy, we screened 266 women and enrolled 204 into MyHealtheBladder and received 190 consults through clinical care pathways.

**Implications for D&I Research:**

Leveraging leadership support and technology within the VHA healthcare system, we anticipate reaching our goal and improving access to nonsurgical UI treatments. Our online toolkit, remote access to MyHealtheBladder, and consult pathways are scalable throughout the VHA, if successful at the regional level.

**Primary Funding Source:** Agency for Healthcare Research and Quality

## Global Dissemination and Implementation Science

### S78 Implementing universal suicide screening in a Nepali emergency department: A mixed methods pilot implementation study

#### Ashley Hagaman^1^, Anmol Shrestha^2^, Roshana Shrestha^2^, Ajay Risal^2^, Renu Shakya^2^, Kripa Sigdel^3^, Riya Bajracharya^2^, Lakshmi Vijayakumar^4^, Divya Gumudavelly^1^, Pratiksha Paudel^2^

##### ^1^Yale School of Public Health, New Haven, CT, USA; ^2^Kathmandu University School of Medical Sciences, Dhulikhel, Nepal; ^3^Possible Health, Kathmandu, Nepal; ^4^Volunteer Health Services Hospital, Chennai, India

###### **Correspondence:** Ashley Hagaman (ashley.hagaman@yale.edu)

*Implementation Science 2024*, **19(2):**S78

**Background:** Suicide detection is an integral component of system-integrated strategies to prevent suicide. However, little research in non-western and low-income contexts has informed if suicide screening is appropriate, feasible, and effective. Nepal has high suicide burden and increased mental health infrastructure. This study investigated the implementation readiness, adaptation, and preliminary implementation of a universal suicide screening and referral approach in a busy Emergency Department of a large peri-rural hospital in Nepal.

**Methods:** We conducted a pilot hybrid type one implementation trial testing a package to support universal screening and referral using the culturally adapted Ask Suicide Questionnaire. We used mixed methods and the EPIS framework to design, adapt, and assess the feasibility and acceptability of universal screening for suicide risk among implementing staff and recipient patients. We conducted embedded ethnography and iterative qualitative interviews to examine implementation climate, barriers, and facilitators. We tracked implementation adaptations with the FRAME.

**Findings:** Between December 2022 and June of 2023, 1258 patients were screened. More than 2.5% of patients screened as high risk, 12% as moderate risk, and 83% as low risk. Clinician knowledge, confidence, and attitudes surrounding implementation significantly increased following training and implementation at 3 and 6 months. All clinicians (n=34) were trained in screening implementation while only 5 nurses/paramedics conducted 75% of all screens. Doctors did not implement any screens but were integral for patient consent and referral procedures. The dynamic adaptation process illuminated several key implementation strategies to support staff implementation including supportive supervision on the floor, staff psychosocial programs, improved patient confidentiality infrastructure, and innovative platforms for data review. Ethnographic and qualitative data highlight deeply important climate and context concerns that demand attention including staff hierarchies, staff mental health, and protocols for department patient overload and patient-family negotiation.

**Implications for D&I Research:** Universal screening indeed requires robust infrastructure, but particularly in resource strained health systems where clinical staff are overburdened and under resourced, additional supports are necessary. While suicide screening is preliminarily feasible, important implementation strategies are necessary to support the staff and climate. We discuss the complex ethicalities, strategies, and future steps for suicide screening in health systems in Nepal.

**Primary Funding Source:** American Foundation for Suicide Prevention

### S79 Identifying implementation strategies supporting World Health Organization’s cervical cancer elimination agenda from ongoing screening programs

#### Prajakta Adsul^1^, Anisha Loeb^2^, Joseph Rodman^3^, Allison Frank^4^, Douglas Puricelli^4,5^, , Leshia Hansen^4^, Margarita Correa-Mendez^4^, Anita Das^4^, Anita Das^4^, Hyo Sook Bae^4^, Kalina Duncan^6^, Patti Gravitt^6^, Linda Eckert^7^, Maribel Almonte^8^, Nathalie Broutet^9^

##### ^1^University of New Mexico, Albuquerque, NM, USA; ^2^University of Washington, Seattle, USA; ^3^University of New Mexico, Albuquerque, USA; ^4^National Institutes of Health, National Cancer Institute, Bethesda, USA; ^5^Clinical Monitoring Research Program Directorate, Fredrick, USA; ^6^National Institutes of Health, Rockville, National Cancer Institute, MD, USA; ^7^University of Washington, Bethesda, USA; ^8^International Agency for Research on Cancer, Lyon, France; ^9^World Health Organization, Geneva, Switzerland

###### **Correspondence:** Prajakta Adsul (padsul@salud.unm.edu)

*Implementation Science 2024*, **19(2):**S79

**Background:** To achieve the global targets for cervical cancer elimination, there is an immediate need to shift our research focus to adoption and implementation of national screening programs aligned with the World Health Organization’s (WHO) guidelines. A key step is to develop practice-based evidence around implementation that can inform the implementation, scale-up, and sustainability of screening programs in an effort to reduce global inequities in cervical cancer.

**Methods:** Guided by the Implementation Working Group for WHO guidelines, we conducted 32 interviews with practitioners involved in cervical cancer screening programs from six WHO Regions. Interviews were informed by the Exploration, Preparation, Implementation, and Sustainment Model, with visual probing techniques tracing the screening, diagnostic, and treatment continuum. Interviews were conducted and recorded on Zoom with simultaneous translation as needed, and notes and transcripts were analyzed to identify implementation strategies.

**Findings:** Overall, 48 unique strategies were found to influence the key outcomes of: (1) improving individual uptake of screening (e.g., using self-collected samples or using a pre-existing community workforce was critical in building community trust for participation), (2) ensuring retention in the screening continuum (e.g., having a well-planned, systematic process for delivering positive results to women), (3) improving provider acceptability (e.g., leveraging relationships with professional organizations, provider reminders), (4) improving national healthcare system adoption and implementation (e.g., engaging partnerships, external facilitation, and funding), and (5) improving global support towards implementation (e.g., building capacity, training, and technical assistance). Compared to the Expert Recommendations for Implementing Change (ERIC) compilation of strategies, the 48 strategies overlapped with 52 of the 73 strategies and health services, financial, and engagement focused strategies. Interestingly, strategies focused on patient navigation, improving care coordination, and conducting cost-effectiveness analysis were mentioned by participants but missing from ERIC.

**Implications for D&I Research:** This work directly provides methodological guidance on tracking and reporting implementation strategies informed by practice-based evidence. Strategies identified in this study build on the ERIC compilation by contextualizing the form and functions as they relate to cervical cancer screening programs. Highlighting commonly used strategies through an in-depth practice-based taxonomy can guide future global and national implementation efforts in varied contexts.

**Primary Funding Source:** World Health Organization

### S80 The Rwanda PLAY collaborative: A hybrid type 2 implementation-effectiveness cluster randomized trial evaluating the scale out of a home-visiting program to promote early child development and prevent violence

#### Theresa Betancourt^1^, Candace Black^2^, Matias Placencio-Castro^2^, Gabriela Phend^2^, Jean Marie Vianney Havugimana^3^, Grace Umulisa^4^, Laura Bond^1^, Kayla Hernandez^2^, Shauna Murray^5^, Vincent Sezibera^6^

##### ^1^Boston College School of Social Work, Chestnut Hill, MA, USA; ^2^Boston College, Chestnut Hill, MA, USA; ^3^FXB Rwanda, Kigali, Rwanda; ^4^FXB Rwanda, Kigali, Rwanda; ^5^Boston College, Chestnut Hill, USA; ^6^University of Rwanda, Kigali, Rwanda

###### **Correspondence:** Theresa Betancourt (theresa.betancourt@bc.edu)

*Implementation Science 2024*, **19(2):**S80

**Background:** In low resource settings, especially those with a history of communal violence, children face elevated risk of underdevelopment, poor health, and exposure to family violence, all of which can lead to intergenerational cycles of poverty and lost human potential. In Rwanda, the national government has established strong policy to promote Early Child Development (ECD) and is actively involved in implementing interventions that support ECD. The Boston College School of Social Work, in partnership with the Government of Rwanda and the University of Rwanda Center for Mental Health, has studied the scale out of an evidence-based home-visiting intervention, Sugira Muryango (“Strengthening Families”, SM) designed to increase father engagement, reduce family violence, and promote ECD.

**Methods:** Using a Collaborative Team Approach called the PLAY Collaborative, SM has been scaled to reach 10,000 of the most impoverished families across three districts in Rwanda while strengthening multi-level stakeholder engagement. Using a Hybrid Type 2 implementation-effectiveness design, we evaluated key implementation outcomes and embedded a cluster randomized trial to evaluate whether SM effectiveness is maintained with expanded eligibility for families with children 0-36 months (previously 6-36 months) and delivery by a new, government child protection workforce, the Inshuti Z'Umuryango (“Friends of the Family”, IZU).

**Findings:** The PLAY Collaborative trained 2,608 IZUs; delivered SM to 19,548 caregivers and 9,483 children; and engaged 15,351 ECD stakeholders and Rwandan government officials. PLAY Collaborative activities involved cross-site learning and shared problem-solving using PDSA cycles, thereby alleviating high workload burden, reducing redundancies, and supporting effective monitoring and case management. Caregivers, IZUs, and PLAY Collaborative members rated SM highly on key dissemination and implementation outcomes. SM families improved home environments; increased parent responsiveness, nurturing care, and play; and reduced some harsh parenting practices. SM fathers engaged in more caretaking and both male and female caregivers experienced reduced depression and anxiety symptoms.

**Implications for D&I Research:** The PLAY Collaborative supported successful scale out while maintaining program quality and impact across several ECD and violence outcomes. In addition to promoting nurturing care in hard-to-reach populations, the PLAY Collaborative is a comprehensive, community-centered implementation strategy which engages key stakeholders and strengthens local structural and institutional support to sustain an EBI.

**Primary Funding Source:** National Institutes of Health

### S81 A stakeholder-selected implementation strategy package improves PrEP implementation for women seeking maternal and child health services in western Kenya

#### Joseph Sila^1^, Anjuli Wagner^2^, Felix Abuna^1^, Julia Dettinger^3^, Ben Odhiambo^1^, Nancy Mwongeli^1^, George Oketch^1^, Enock Sifuna^1^, Lauren Gomez^3^, Sarah Hicks^3^, Grace John-Stewart^3^, John Kinuthia^4^, Bryan Weiner^3^

##### ^1^Kenyatta National Hospital, Nairobi, Kenya; ^2^University of Washington, Seattle, USA; ^3^University of Washington, Seattle, WA, USA; ^4^Department of Research & Programs, Kenyatta National Hospital, Nairobi, Kenya

###### **Correspondence:** Anjuli Wagner (anjuliw@uw.edu)

*Implementation Science 2024*, **19(2):**S81

**Background:** Pre-exposure prophylaxis (PrEP) is recommended by the WHO and Kenyan Ministry of Health as a safe and effective HIV prevention method during pregnancy and postpartum. PrEP integration in maternal and child health (MCH) clinics is feasible; however, evidence on implementation strategies to support integration is scarce.

**Methods**: From August 2022 to April 2023, the PrEPARE team conducted a difference-in-differences study in Kisumu, Homa Bay, and Siaya counties of Western Kenya. The study included 3 months pre- and 3 months post-intervention periods with 4 intervention & 4 comparison facilities. We tested a 3-component stakeholder-selected implementation strategy bundle to improve PrEP delivery: 1) training different cadres of health care workers, 2) task shifting PrEP counselling from nurses/clinicians to HIV testing providers, and 3) dispensing PrEP in MCH clinics. Absolute changes were compared in percentage of women screened for PrEP (PrEP penetration), proportion receiving all PrEP steps (HIV testing, risk screening, PrEP counseling; PrEP fidelity), client PrEP knowledge, client satisfaction, and timeliness. We measured provider perceptions of acceptability and appropriateness of the strategy bundle.

**Findings**: Overall, 1,916 clients completed exit surveys (959 in intervention; 957 in comparison periods) and 768 completed service timings surveys (384 for each period). The median age was 25 years and 15.6% were seeking their first antenatal services.

We observed significant increases in two implementation outcomes in intervention facilities relative to comparison facilities: PrEP penetration (+7.0 percentage points; p=0.004) and PrEP fidelity (+16.7 percentage points; p<0.001). There were non-significant increases in PrEP offer (+7.4 percentage points; p=0.071) and client PrEP knowledge (+0.22/7 total points; p=0.059). There were no changes in satisfaction (-0.098/24 points; p=0.539), service time (-0.13 minutes; p=0.249) or waiting time (+0.20 minutes; p=0.131). HCW perceptions of the implementation strategy bundle were favorable (median acceptability: 18/20 points; median appropriateness: 19/20 points).

**Implications for D&I Research:** A stakeholder-selected-implementation strategy bundle with training different cadres on PrEP, task shifting PrEP counselling, and PrEP dispensing in MCH improved PrEP penetration and fidelity without adversely affecting satisfaction, service time, or waiting time. These strategies may be useful in scaling up PrEP in low- and middle-income countries with health care worker shortages.

**Primary Funding Source:** National Institutes of Health

### S82 Implementation of a virtual intervention for adolescents and young people on ART: Barriers and recommendations

#### Cathrine Chinyandura^1^, Anele Jiyane^1^, Fezile Buthelezi^1^, Kate Rees^1^, Roshit Ramaswamy^2^, Sophie Marie Bartels^2^, Audrey Pettifor^2^

##### ^1^Anova Health Institute, Johannesburg, South Africa; ^2^University of North Carolina, Chapel Hill, Chapel Hill, NC, USA

###### **Correspondence:** Cathrine Chinyandura (chinyandura@anovahealth.co.za)

*Implementation Science 2024*, **19(2):**S82

**Implementation of a virtual Intervention for adolescents and young people on ART: barriers and recommendations**

**Background:** Youth Care Clubs (YCC) are youth-friendly adherence groups that provide integrated psychosocial support and clinical care to adolescents and young people living with HIV (AYPLHIV) aged 12-24 years. Anova Health Institute supports the Department of Health to implement YCCs in Johannesburg District. During COVID-19, we transitioned YCCs to virtual platforms. Virtual interventions enable continuous support without physical visits to clinics, thus supporting differentiated services. We assessed the acceptability, barriers and facilitators to implementation of virtual Youth Care Clubs (vYCCs) in Johannesburg, South Africa.

**Methods:** We used a mixed-methods design guided by the Consolidated Framework for Implementation Research (CFIR). Data was collected from July 2022 to April 2023 from 22 clinics. A total of 175 AYPLHIV completed a questionnaire online or during in-person YCCs. We conducted 6 in-person focus group discussions with a subset of survey participants. In-depth interviews were conducted with 30 service providers, 22 facilitators and 8 managers. We used descriptive statistics to analyze survey data and thematically analyzed qualitative data through an iterative, team-based coding process, blending deductive and inductive approaches.

**Findings:** vYCCs were highly acceptable for AYPLHIV and providers, although many preferred in-person or hybrid models. We identified *structural characteristics, compatibility, availability of resources*, *relative priority*, *critical incidents* and *innovation cost* as key barriers. *Critical incidents* due to loadshedding (scheduled power cuts) and the high cost of data incurred by AYPLHIV hindered implementation. Barriers varied by age, older AYPLHIV were more likely to have access to data and smart phones. The major facilitators were adaptability, relative advantage, complexity and design of the program. The flexibility of the program to be customized to participants’ needs was convenient to users. *Complexity* and *design* were facilitators as both providers and AYPLHIV considered the program appropriate, understandable and easy to implement.

**Implications for D&I Research:** vYCCs are feasible, youth friendly, acceptable, convenient and have the potential to reach an extended population of AYPLHIV. To improve implementation, barriers must be addressed, particularly availability of resources and disruptive events such as loadshedding. The adaptability of the program should be leveraged.

**Primary Funding Source:** National Institutes of Health

### S83 Key influences on the sustainability of an adolescent transition package for youth living with HIV in Kenya

#### Sarah Shaw^1^, Kristin Beima-Sofie^2^, Caren Mburu^1^, Cyrus Mugo^1,3^, Alvin Onyango^3^, Florence Nyapara^3^, Calvince Aballa^3^, Dalton Wamalwa^4^, Grace John-Stewart^2^, Irene Njuguna^1,3^

##### ^1^University of Washington, Seattle, USA; ^2^University of Washington, Seattle, WA, USA; ^3^Kenyatta National Hospital, Nairobi, Kenya; ^4^University of Nairobi, Nairobi, Kenya

###### **Correspondence:** Sarah Shaw (sarahs29@uw.edu)

*Implementation Science 2024*, **19(2):**S83

**Background:** Improving HIV treatment outcomes among youth living with HIV (YLH) requires sustaining evidence-based interventions (EBIs) into routine care. The ATTACH study developed and evaluated an Adolescent Transition Package (ATP) which improved youth’s readiness to transition from pediatric to adult care. We aimed to identify factors influencing implementation of the ATP post-trial.

**Methods:** One year post-trial, we surveyed 147 healthcare workers (HCWs) supporting YLH at the 20 ATTACH clinics and interviewed a purposive subset of HCWs from each site (2-3 per site). We conducted descriptive analyses of survey data to understand implementation outcomes influencing sustainability. Informed by the Consolidated Framework for Implementation Research (CFIR) and Structural Readiness for Change, we conducted thematic analyses of debrief reports and a subset of transcripts to explore themes on sustainability.

**Findings:** HCWs had implemented the ATP for a mean of 1.7 years and most (90%) were trained by the ATTACH team. Almost all (94%) HCWs agreed they would like to continue using the ATP. Long-term implementation was influenced by strong clinic support, ease of integrating the ATP into clinic operations, and supportive HIV clinic leadership. Barriers to sustained use included lack of ownership among new staff who did not participate in the ATTACH study and high workload. HCWs felt strongly that the ATP was an appropriate tool to improve adherence, retention, and viral suppression among YLH. When evaluating long-term fidelity to intervention tools, HCWs used the disclosure and transition booklets most frequently. However, some clinics adapted booklet use to focus only on YLH experiencing care challenges. When considering future scale-up, HCWs highlighted the need for adolescent champions to support intervention delivery, integration into electronic medical records, strategies to increase HCW motivation, and Ministry of Health engagement.

**Implications for D&I Research:** We observed high rates of continued ATP use across sites one year post-trial, likely attributed to the high acceptability, appropriateness, and feasibility of the ATP. Given variations in implementation fidelity, strategies promoting inclusive training and reduced HCW workload such as refresher courses and simplified tools, may support sustainability. Understanding the factors which influence sustainability outside of research settings can inform future scale-up of the ATP and other EBIs.

**Primary Funding Source:** National Institutes of Health

### S84 Impact of an implementation facilitation strategy to improve task-shared CBT across education and health sectors in Kenya: A mixed methods study

#### Prerna Martin^1^, Caroline Soi^2^, Sharon Kiche^2^, Christine Gray^3^, Julie Nguyen^4^, Rashed AlRasheed^2^, Noah Triplett^2^, Clara Johnson^2^, Cyrilla Amanya^5^, Bryan Weiner^2^, Kathryn Whetten^3^, Shannon Dorsey^2^

##### ^1^University of California, Los Angeles, Los Angeles, CA, USA; ^2^University of Washington, Seattle, WA, USA; ^3^Duke University, Durham, NC, USA; ^4^University of South Carolina, Columbia, SC, USA; ^5^ACE Africa Kenya, Bungoma, Kenya

###### **Correspondence:** Prerna Martin (prmartin@ucla.edu)

*Implementation Science 2024*, **19(2):**S84

**Background:**

Mental health interventions can be effectively delivered via task-sharing in LMIC; however, methods to scale and sustain interventions are lacking. We examine the impact of facilitation or “coaching” as an implementation strategy to improve task-shared delivery of trauma-focused CBT (TF-CBT) for orphans in Kenya. The effects of facilitation on mental health programs in LMIC are largely unexplored, making this a significant contribution to the limited implementation research in LMIC.

**Methods:**

Within a NIMH-funded R01 trial, we collaboratively developed a novel, minimally intensive implementation coaching strategy delivered by Kenyan lay counselors. We used mixed methods (QUAN + QUAL) to assess the impact and perceived value of coaching to improve TF-CBT implementation in the education and health sectors. A total of 150 lay counselors (75 teachers, 75 community health volunteers [CHVs]) completed quantitative surveys. We used generalized estimating equations to analyze the relationship between coaching condition and provider- and organizational-level implementation outcomes. We applied rapid qualitative methods and conducted semi-structured interviews with 32 lay counselors (16 teachers, 16 CHVs) to explore perceptions of coaching.

**Findings:**

Coaching condition predicted higher acceptability and feasibility of TF-CBT among teachers, and lower feasibility and appropriateness of TF-CBT among CHVs. Coaching did not affect implementation climate or leadership. Qualitatively, teachers and CHVs reported high acceptability, feasibility, and utility of coaching as it increased readiness for implementation and helped address multilevel implementation barriers.

**Implications for D&I Research:**

This is the first global study to assess lay counselor perspectives on the impact of implementation coaching to support child mental health interventions in LMIC. Study findings inform strategies to provide tailored implementation support for task-shared interventions in U.S. and global low-resource settings.

**Primary Funding Source:** National Institutes of Health

### S85 Applying social network analysis and processes mapping to inform the implementation of a cysticercosis control program in rural Peru

#### Lisset Dumet^1^, Angela Spencer^1^, Ruth Atto^2^, Vanessa Cruz^2^, Percy Vilchez^2^, Javier Bustos^3^, Hector Garcia^4^, Sarah Gimbel^5^, Seth O'Neal^6^

##### ^1^Oregon Health & Science University - Portland State University School of Public Health, Portland, OR, USA; ^2^Centro de Salud Global, Universidad Peruana Cayetano Heredia, Tumbes, Peru; ^3^Centro de Salud Global, Universidad Peruana Cayetano Heredia, Lima, Peru; ^4^Centro de Salud Global, Universidad Peruana Cayetano Heredia; Cysticercosis Unit, National Institute of Neurosciences; Department of Microbiology, Universidad Peruana Cayetano Heredia, Lima, Peru; ^5^University of Washington, Seattle, WA, USA; ^6^OHSU - PSU School of Public Health, Portland, OR, USA

###### **Correspondence:** Lisset Dumet (dumet@ohsu.edu)

*Implementation Science 2024*, **19(2):**S85

**Background:**

Cysticercosis is an infectious disease responsible for 30-40% of acquired epilepsy in endemic areas such as rural Peru. Ring Treatment (RT) is a community-engaged cysticercosis control strategy, efficacious when delivered by research teams. We applied the Consolidated Framework for Implementation Research and used social network analysis (SNA) and process mapping to understand the inner setting (e.g., networks and communications) and outer setting (e.g., cosmopolitanism) to develop a protocol for implementing RT.

**Methods:**

We included small, medium, and large municipal districts (populations served <5000; <6000; <36000, respectively) from northern Peru representing different rural primary healthcare delivery settings. We conducted 169 semi-structured interviews with municipal health workers, with questions from the CFIR interview guide tool about networks and opinion leaders. We interviewed the external collaborators participants nominated and applied SNA to identify key players, roles, and collaborations to inform the RT implementation. We developed process maps to identify the steps and critical people to deliver RT. In preparation for a subsequent pilot study, our team delivered workshops about cysticercosis transmission and the RT intervention to the small district's healthcare team and community agents. Workshop participants proposed and presented process maps for implementing RT, followed by a group discussion.

**Findings:**

Participants named 172 external collaborators. Districts' network sizes differed among small, medium, and large districts. However, some key players for successful implementation were similar among districts, such as the head of the health care center and the community health care worker (CHW). CHWs had more nominations in the small district than in larger districts. Proposed process maps created in the workshops identified the importance of integrating other community players in the intervention, such as the mayor, and brought new ideas for community case reporting, incentives, and forms for evaluation and reporting.

**Implications for D&I Research:**

This presentation describes implementation progress to translate an effective approach to control infectious disease transmission in a low-resource rural setting. Implementation tools allowed us to integrate health professionals' feedback for better optimization of the protocol which is currently being piloted in preparation for scaling-up to multiple health districts.

**Primary Funding Source:** National Institutes of Health

### S86 Documenting adaptations to an evidence-based intervention in 58 resource-variable pediatric oncology hospitals across implementation phases

#### Asya Agulnik^1^, Alejandra Quesada-Stoner^2^, Sayeda Islam^3^, Amela Sijecic^3^, Sara Malone^4^, Maria Puerto-Torres^5^, Adolfo Cardenas^6^, Kim Prewitt^3^, Bobbi Carothers^7^, Douglas Luke^8^, Virginia Mckay^7^

##### ^1^St. Jude Children’s Research Hospital, Memphis, TN, USA; ^2^St. Jude Children's Research Hospital, Memphis, TN, USA; ^3^Washington University in St. Louis, St. Louis, MO, USA; ^4^Department of Surgery (Division of Public Health Sciences) and Alvin J. Siteman Cancer Center, Washington University School of Medicine, St. Louis, MO, USA; ^5^St. Jude Children's Research Hospital, Memphis, USA; ^6^St. Jude Children’s Research Hospital, Memphis, USA ^7^Center for Public Health Systems Science, Brown School, Washington University in Saint Louis, St. Louis, MO, USA; ^8^Center for Public Health Systems Science, Washington University in St. Louis, Saint Louis, MO, USA

###### **Correspondence:** Asya Agulnik (Asya.Agulnik@STJUDE.ORG)

*Implementation Science 2024*, **19(2):**S86

**Background:** Adapting evidence-based interventions (EBIs) is necessary to fit a variety of local contexts and promote implementation in resource-limited settings. Pediatric Early Warning Systems (PEWS) are EBIs that aid early identification of deterioration in hospitalized children with cancer. It is unclear, however, what components of PEWS are adapted and how these changes impact PEWS fidelity. This study evaluates PEWS adaptations among 58 resource-variable pediatric oncology hospitals implementing and sustaining PEWS.

**Methods:** Clinical staff using PEWS at 58 diverse Spanish- and Portuguese-speaking pediatric cancer centers from 19 countries in Latin America and Europe participating in a collaborative to implement PEWS were included. Adaptations to PEWS were assessed via 3 multiple choice and 1 free-text questions administered anonymously during PEWS planning, pilot, implementation, and sustainability. Descriptive statistics were used to quantitatively describe what, when, and why adaptations were made. Qualitative analysis of free-text responses applied the Framework for Reporting Adaptations and Modifications made to Evidence-based interventions, expanded (FRAME) to describe details of staff perspectives on PEWS adaptations.

**Findings:** We analyzed 1984 responses from 58 pediatric oncology centers. Participants were predominantly female (n=1629, 82.1%). Most were nurses (n=1094, 55.1%) and physicians (n=727, 36.6%) who were either PEWS implementation leaders (n=434, 21.9%) or clinical staff (n=1373, 69.2%). Respondents described a range of adaptations made to PEWS across all implementation phases, including to the PEWS tool, when and how often PEWS is used in patient care, and the patient population to which PEWS was applied. Reasons for adaptations included to better fit with local context and available resources, make PEWS easier to use and monitor, better integrated with clinical workflow, and make PEWS more effective. Qualitative analysis of participants free-text descriptions of adaptations suggests these were primarily fidelity-congruent and further identified staff perspectives on impact of adaptations on PEWS implementation and effectiveness.

**Implications for D&I Research:** This mixed-method evaluation of adaptations made to PEWS across implementation phases in resource-variable pediatric oncology centers highlight the variety of ways EBIs are adapted to fit real-world clinical settings. This work pushes implementation science to examine how to guide adaptations of EBIs to promote global scale up in hospitals of all resource-levels.

**Primary Funding Source:** National Institutes of Health

### S87 The team approach to malnutrition services (TeAMS) toolkit: Co-design of a tailored implementation strategy for improved adherence to Kenyan pediatric guidelines for children with acute malnutrition

#### Megan Coe^1^, Geoffrey Okoth Olieng'o^2^, Megan Lewis^1^, Phlona Amam^2^, Chrisantus Oduol^2^, Catherine Achieng^2^, Sarah Gimbel^1^, Beth Kolko^1^, Benson Singa^1,3^, Arianna Means^1^

##### ^1^University of Washington, Seattle, WA, USA; ^2^KEMRI-UW, Migori, Kenya; ^3^Kenya Medical Research Institute (KEMRI), Nairobi, Kenya

###### **Correspondence:** Megan Coe (mmcoe@uw.edu)

*Implementation Science 2024*, **19(2):**S87

**Background:**

Malnutrition plays a part in 45% of global child deaths and is an important risk factor for death among hospitalized children in Kenya. Although treatment guidelines for malnutrition have been available for decades, adherence to them remains suboptimal. Feasible implementation strategies that support health workers in providing guideline-adherent care are urgently needed.

**Methods:**

We used a participatory co-design process to select a challenge to focus on, identify an implementation strategy to address the challenge, and tailor the strategy to meet the needs of health workers at one hospital in Kenya. First, a barrier-strategy mapping workshop utilizing the nominal group technique was conducted to match previously identified barriers to potential interventions. The selected strategy was then tailored for use in the facility through both an iterative design process informed by human-centered design methods and questionnaire responses focused on perceived competence and role perceptions.

**Findings:**

Seven health workers participated in the barrier-strategy mapping workshop and selected the challenge that reliance on nutritionists to handle critical tasks sometimes delays guideline adherent care. They then brainstormed ideas to overcome this challenge, ranked them based on acceptability, feasibility, and potential for impact, and came to consensus on a strategy that clarifies team member roles in providing care to children with malnutrition and summarizes information from guidelines to support performance of these roles. To develop the Team Approach to Malnutrition Services (TeAMS) Toolkit, eleven design sessions have been conducted to date, with additional sessions planned in July 2023. Activities in design sessions included the use of scenarios and cognitive walkthrough and results identified participants’ perceptions of team member roles, priorities for content to include, and ease of using a Toolkit prototype. This feedback informed refinement of the Toolkit, which will be piloted in the coming months.

**Implications for D&I Research:**

We have gained valuable insights into the use of design methods to develop a tailored implementation strategy in a Kenyan hospital. Participants actively engaged and offered feedback that was instrumental in strategy development. We also learned from challenges in balancing design activity scope with available time and harmonizing the speed and flexibility of design methods with the expectations of a research environment.

**Primary Funding Source:** University of Washington

### S88 Impact of contextual factors on bubble continuous positive airway pressure (bCPAP) implementation for pediatric respiratory distress in low- and middle-income countries: A realist review

#### Nadir Ijaz^1^, Maria Nader^1^, Matthew Ponticiello^1^, Ashlee Vance^2^, Brittney van de Water^3^, Melissa Funaro^4^, Sebastián González^5,6^, Patrick Wilson^7^, Brenda Morrow^8^, J. Lucian Davis^1,9^

##### ^1^Yale University School of Medicine, New Haven, CT, USA; ^2^Henry Ford Health, Detroit, MI, USA; ^3^Boston College, Chestnut Hill, MA, USA; ^4^Yale University, New Haven, CT, USA; ^5^Red Colaborativa Pediátrica de Latinoamérica (LARed Network), Montevideo, Uruguay; ^6^Universidad de la República, Montevideo, Uruguay; ^7^University of Colorado, Aurora, CO, USA; ^8^University of Cape Town, Cape Town, South Africa; ^9^Yale School of Public Health, New Haven, CT, USA

###### **Correspondence:** Nadir Ijaz (nadir.ijaz@yale.edu)

*Implementation Science 2024*, **19(2):**S88

**Background:** Bubble continuous positive airway pressure (bCPAP) is a low-cost therapy that has been associated with large mortality reductions in the treatment of pediatric pneumonia in some settings but increased mortality in other contexts. The objectives of our realist review are to (1) identify contextual factors impacting safe and effective bCPAP implementation in children under 5 years in low- and middle-income countries (LMICs) and (2) develop a pertinent implementation theory.

**Methods:** We searched peer-reviewed and grey literature for publications describing bCPAP use for respiratory distress in our target population. Following a realist approach, we coded implementation-related themes using abductive content analysis to develop an initial program theory in the form of context-mechanism-outcome (CMO) configurations. We assembled a multi-sectoral, international advisory panel of bCPAP experts to refine the theory through iterative written individual critiques, focus groups, and, when necessary, key informant interviews. We organized resulting themes using the socio-ecological model.

**Findings:** We screened 821 abstracts and 83 full text documents. We included 36 peer-reviewed references in our review. We identified relevant contextual factors, including *prior bCPAP experience*, *intensive care capacity*, *financial resources*, *oxygen and electricity availability, availability of single-use consumables*, *bCPAP introduction in research studies*, *adequate staffing*, *the role of nurses*, *local clinical practice guidelines*, and *program priority*. Both implementation outcomes (i.e., feasibility, acceptance, sustainability) and service outcomes (i.e., quality, safety, complications) were identified. Mechanisms linking contexts to outcomes included *bCPAP integration into pre-existing frameworks, efficient resource allocation*, *emergence of local champions*, *staff skill specialization and retention*, *staff time for bCPAP adjustment, reduced care disruptions, a culture reinforcing standards of care, and community trust-building*. We generated an initial program theory consisting of 16 CMO configurations; half occurred at the institutional level. Next steps include: (1) grey literature review, (2) program theory refinement with expert input, and (3) generation of a final implementation theory by October 2023.

**Implications for D&I Research:** In this realist review conducted in collaboration with a representative group of global, multi-sectoral experts, we found many contextual factors impacting safe and effective bCPAP implementation in LMICs. Local teams should consider the applicability of these factors to their contexts and identify tailored strategies to facilitate implementation.

**Primary Funding Source:** National Institutes of Health

### S89 Introducing a scalable model for embedded implementation science in resource-constrained healthcare systems through participatory co-design

#### Fatou Jallow^1^, Aubrey Villalobos^1^, Elise Garton^1^, Ann Chao^1^, Julia Gage^1^, Patti Gravitt^1^, Andrea Matos^2^, Maria del Carmen Caruhapoma^3^, Víctor Palacios^3^, Carlos Santos^3^, Milagros Montes^4^, Margarita Correa-Mendez^5^, Hawa Camara^5^, Hyo Sook Bae^5^, Peter Hovmand^6^, Camilo Olaya^7^, Laura Guzman^7^, Laura Nervi^8^, Joanna Brown^9^, Karina Roman^9^, Valerie Paz Soldan^10^

##### ^1^National Institutes of Health, National Cancer Institute, Rockville, USA; ^2^Peruvian Ministry of Health, Lima, Peru; ^3^Ministerio de Salud, Peru, Lima, Peru; ^4^DIRIS Lima Norte, Lima, Peru; ^5^National Institutes of Health, National Cancer Institute, Bethesda, USA; ^6^Case Western Reserve University, Cleveland, OH, USA; ^7^Universidad de los Andes, Bogata, Colombia; ^8^University of New Mexico, Albuquerque, USA; ^9^AB PRISMA, San Miguel, Peru; ^10^Tulane University School of Public Health and Tropical Medicine, New Orleans, LA, USA

###### **Correspondence:** Fatou Jallow (fatou.jallow@nih.gov)

*Implementation Science 2024*, **19(2):**S89

**Background:** The successful Integrative Systems Praxis for Implementation Research (INSPIRE) methodology was developed based on systems thinking principles to actively engage implementers and researchers in shared decision-making and program adaptation. We conducted a 5-day series of participatory ‘co-design workshops’ for the implementation of a new HPV cervical screening program in the Northern Lima region of Peru to determine whether short cycles of facilitated reflection on implementation could lead to improved outcomes, with ongoing embedded facilitation with the Peruvian Ministry of Health (MOH) as the program was scaled to 12 additional regions.

**Methods:** For the workshop, we assembled a team of local and international experts in various relevant fields. Workshop agendas were co-designed by the MOH, Northern Lima, and the US National Cancer Institute (NCI) using principles of participatory action research, implementation science, and systems thinking. To clarify the goals and processes of the HPV cervical screening program and reflect on the initial implementation experience, the design was attentive to a) building trust among the health system planners and actors, b) surfacing misconceptions and process bottlenecks, c) equitable representation of perspectives on the process from all levels of the screening system, and d) shared decision-making in actions to improve the system. Sustainment of the facilitation was enabled by a local NGO team with content and facilitation expertise and close ties to the MOH and the communities it serves.

**Findings:** Context-specific challenges were rapidly identified through real-time participatory reflection and process mapping of the cervical cancer screening program. Participants gained a shared understanding of the complexity of the screening system and identified how one part of the system created barriers for proximal and distal parts of the system. Previously detached from the distal barriers, after the workshops, the participants now envisioned their role in decision-making and problem-solving. They demonstrated a willingness to actively participate in implementation improvement, ensuring women received appropriate follow-up care.

**Implications for D&I Research:** Rapid embedded implementation research using facilitated workshops was an acceptable quality improvement activity. The MOH continues to work with a local NGO on facilitating workshops in new regions to enable broader adaptation and scale-up of the HPV cervical screening program.

**Primary Funding Source:** National Institutes of Health

### S90 Inductive approaches to bi-directional learning and implementation adaptation in embedded implementation research

#### Margarita Correa-Mendez^1^, Aubrey Villalobos^2^, Julia Gage^2^, Patti Gravitt^2^, Fatou Jallow^2^, Laura Nervi^3^, Andrea Matos^4^, Maria del Carmen Caruhapoma^5^, Víctor Palacios^5^, Carlos Santos^5^, Milagros Montes^6^, Joanna Brown^7^, Valerie Paz Soldan^8^

##### ^1^National Institutes of Health, National Cancer Institute, Bethesda, USA; ^2^National Institutes of Health, National Cancer Institute, Rockville, MD, USA; ^3^University of New Mexico, Albuquerque, USA; ^4^Peruvian Ministry of Health, Lima, Peru; ^5^Ministerio de Salud, Peru, Lima, Peru; ^6^DIRIS Lima Norte, Lima, Peru; ^7^AB PRISMA, San Miguel, Peru; ^8^Tulane University School of Public Health and Tropical Medicine, New Orleans, LA, USA

###### **Correspondence:** Margarita Correa-Mendez (margarita.mendez@nih.gov)

*Implementation Science 2024*, **19(2):**S90

**Background:** Improving the adoption and integration of cervical cancer screening programs in dynamic, resource-constrained global settings is critical, requiring engaged implementation science approaches. We hypothesized that participatory, facilitated co-design workshops are a replicable model leading to rapid ascertainment of implementation barriers and context-adapted implementation strategies through bi-directional learning.

**Methods:** In November 2022, we held five 1-day-long facilitated workshops with the Peruvian Ministry of Health (MOH) and health system actors from the Northern District of Lima. As research partners, we analyzed notes from the facilitated dialogue and system mapping activities using the Consolidated Framework for Implementation Research and the Expert Recommendations for Implementing Change strategies, to identify priority multilevel contextual factors that influence program implementation and aligned strategies for improvement.

**Findings:** We identified three key context-specific challenges: 1) power hierarchies between specialists and midwives obstructing the efficiency of screening services; 2) complexities around communicating screening results, appointments, and referrals are burdensome for patients, causing loss to follow up; and 3) implementation plans are not adequately matched to resources and workforce capacity. We identified matched strategies to address these challenges: 1) develop relationships between midwives and specialists through additional facilitated discussions to surface assumptions and align screening goals; 2) co-create counseling tools to help patients understand results and follow-up steps for early detection and treatment of precancerous lesions that also reduce patients’ burden; and 3) apply design workshops with key actors to develop tailored implementation plans that align goals to prevention objectives and resources. In 8 months since the workshop, the MOH team expanded screening to 12 new regions and held meetings incorporating planning tools and facilitation strategies to tailor implementation plans to local resources. They have initiated engagement between champion gynecologists and local hospital specialists.

**Implications for D&I Research:** Engaging health systems actors through rapid, participatory, system design workshops enable reciprocal learning for scaling up implementation, identifying context-specific priorities, and tailoring strategies to accelerate integration of sustainable cervical cancer screening programs. The embedded IR increased capacity of the MOH team to facilitate their own workshops as a continuous quality improvement program requiring only modest ongoing support from embedded implementation scientists.

**Primary Funding Source:** National Institutes of Health

### S91 The Peruvian experience of scaling-up a national cervical cancer screening program incorporating tailored, embedded and participatory co-design processes

#### Andrea Matos^1^, Maria del Carmen Caruhapoma^2^, Víctor Palacios^2^, Milagros Montes^3^, Carlos Santos^2^, Valerie Paz Soldan^4^, Joanna Brown^5^, Karina Roman^5^, Julia Gage^6^, Fatou Jallow^6^, Patti Gravitt^6^ , Laura Nervi^7^

##### ^1^Peruvian Ministry of Health, Lima, Peru; ^2^Ministerio de Salud, Peru, Lima, Peru; ^3^DIRIS Lima Norte, Lima, Peru; ^4^Tulane University School of Public Health and Tropical Medicine, New Orleans, LA, USA; ^5^AB PRISMA, San Miguel, Peru; ^6^National Institutes of Health, National Cancer Institute, Rockville, USA; ^7^University of New Mexico, Albuquerque, USA

###### **Correspondence:** Julia Gage (julia.gage@nih.gov)

*Implementation Science 2024*, **19(2):**S91

**Background:** The Peruvian Ministry of Health Cancer Prevention and Control Program (MOH-CPCP) provides health care to the country’s most socio-economically disadvantaged population not covered by other insurance. In 2021, MOH-CPCP aligned with the World Health Organization’s call to eliminate cervical cancer by initiating a human papillomavirus (HPV)-based screening program utilizing self-sampling. With large-scale procurement, trainings, information systems, and laboratories, the initiative launched in 3 metropolitan regions of Lima and Junín and Loreto provinces. In 2023, scale-up was expanded to 12 additional provinces. The MOH-CPCP technical team partnered with Proyecto Precancer (PPC) research team (comprised of international content experts and an NGO with long ties to the MOH-CPCP and the communities it serves) and the US National Cancer Institute (NCI) to facilitate system-level planning.

**Methods:** Regular small meetings between the three parties established a strong foundation for ongoing engagement at all levels of MOH leadership. Conversations often focused on the importance of trust among health system planners and actors, shared understanding of the system and decision-making, and consideration of resource constraints in intermediate and long-term planning. In November 2022, MOH-CPCP and NCI led a series of co-design workshops to reflect on implementation experiences and clarify goals and processes. During the 2^nd^ wave of scale-up, PPC helped facilitate regional planning conversations, where MOH-CPCP shared local experiences for implementation science dissemination.

**Findings:** The shared understanding of the system’s complexity gained from workshops promoted novel adaptations, allowing MOH-CPCP to successfully defend the new program and advocate for resources. During scale-up, MOH-CPCP adapted and incorporated planning tools ensuring a shared understanding of the importance of resource-stratified planning and staged implementation, leading to realistic, achievable goals. In secondary care settings, acceptability barriers remain, requiring continued adaptations. The context-specific priorities and implementation strategy interventions identified by PPC and NCI scientists are being communicated internationally.

**Implications for D&I Research:** This model of embedded engagement successfully allowed MOH-CPCP to create sustainable changes in cervical cancer screening planning and implementation. The MOH-CPCP has experienced a changed orientation to planning, away from aspirational goals like 70% screening coverage, towards realistic short-term goals like incremental scale-up within a region

**Primary Funding Source:** National Institutes of Health

### S92 System dynamics modelling & reverse engineering as implementation strategy

#### Peter Hovmand^1^, Camilo Olaya^2^, Laura Guzman^2^, Andrea Matos^3^, Maria del Carmen Caruhapoma^4^, Víctor Palacios^4^, Carlos Santos^4^, Milagros Montes^5^, Fatou Jallow^6^, Julia Gage^6^, Patti Gravitt^6^ Joanna Brown^7^, Karina Roman^7^, Valerie Paz Soldan^8^

##### ^1^Case Western Reserve University, Cleveland, OH, USA; ^2^Universidad de los Andes, Bogata, Colombia; ^3^Peruvian Ministry of Health, Lima, Peru; ^4^Ministerio de Salud, Peru, Lima, Peru; ^5^DIRIS Lima Norte, Lima, Peru; ^6^National Institutes of Health, National Cancer Institute, Rockville, USA; ^7^AB PRISMA, San Miguel, Peru; ^8^Tulane University School of Public Health and Tropical Medicine, New Orleans, LA, USA

###### **Correspondence:** Peter Hovmand (psh39@case.edu)

*Implementation Science 2024*, **19(2):**S92

**Background:** System Dynamics (SD) is a system science method for understanding and managing complex social systems. Group Model Building (GMB) is an approach to SD that engages stakeholders in the process of conceptualizing a system along with the selection and tailoring of implementation strategies. Central to GMB is the use of models as boundary objects that allow stakeholders to develop shared understanding of a system and identify leverage points for change. However, use of SD/GMB in implementation science has been limited by the formality of the SD modeling conventions. More efficient and scalable methods are needed for eliciting stakeholders’ mental models for SD modeling in implementation science.

**Methods:** National and local government decision makers along with nurses, midwives, physicians and pathologists participated in 4-days of GMB workshops on cervical cancer prevention using flowcharts as the main boundary objects. Facilitators and experts helped build consensus and shared understanding expressed as aggregated flowcharts, which were then “reversed engineered” to create locally-informed SD models that included barriers, good practices, and opportunities for scaling cervical cancer prevention.

**Findings:** Reverse engineering using flowcharts led to SD models identifying future drivers of implementation dynamics of cervical cancer prevention scale-up. Three major short-term feedback control loops were identified including (1) the efforts of the Ministry of Health, the DIRIS Lima Norte and the primary level of attention for closing the gap between the screening goal and the actual number of screened women*,* (2) gynecologists and screening providers controlling the number of women drop-out through counseling, and (3) gynecologists controlling the number of confirmed women with pre-cancer through treatment*.* These feedback control loops compete with hidden reinforcing loops of long-term re-screening of HPV negatives, triage negatives and treated women which in turn are reinforced by the inertia of accumulations.

**Implications for D&I Research:** SD-based reverse engineering amplifies deep immersion research by building explicit models of complex adaptive systems underlying implementation dynamics, identifying selection and tailoring of implementation strategies, that can subsequently be formalized into SD computer simulation models for identifying endogenous sources of implementation outcome dynamics, explore what-if scenarios and design high-impact contextually relevant implementation strategies.

**Primary Funding Source:** National Institutes of Health

## Health Policy Dissemination and Implementation Science

### S93 Developing implementation strategies for SNAP enrollment among a diverse Latinx community

#### Elena Byhoff^1^, Rebecca Rudel^2^, Tuhin Roy^3^, Keith Nokes^4^, Mari-Lynn Drainoni^5^

##### ^1^University of Massachusetts Chan Medical School, Worcester, MA, USA; ^2^Boston Medical Center, Boston, MA, USA; ^3^Greater Lawrence Family Health Center, Lawrence, MA, USA; ^4^Tufts University School of Medicine, Boston, MA, USA; ^5^Boston University School of Medicine, Boston, MA, USA

###### **Correspondence:** Elena Byhoff (elena.byhoff@umassmed.edu)

*Implementation Science 2024*, **19(2):**S93

**Background:** SNAP (the Supplemental Nutrition Assistance Program) is the largest federal nutrition assistance program aimed at improving food security and nutrition among low-income individuals and families. We designed and conducted a SNAP outreach and enrollment implementation pilot in a community where 71% of individuals face food insecurity but only 32% of eligible persons receive SNAP benefits. Causes of under-enrollment are multifactorial and include federal policies that penalize immigrants who use public benefit programs, state policies that limit enrollment for recent immigrant populations, stigma, and administrative enrollment barriers.

**Methods:** Anchored in the PRISM framework, we convened a stakeholder panel to design and pilot SNAP enrollment strategies incorporating external context (state/federal policy, community characteristics) and internal context (program user and staff perspectives). Panel members included community organization SNAP specialists; state SNAP program staff; community health workers and providers from a Community Health Center; and an academic partner. The stakeholder panel met weekly from 2020-2023 to review existing data on SNAP enrollment barriers and identify feasible strategies to increase program reach. Strategies were piloted from February – May 2023 and data on SNAP enrollment was collected by the community partner, as available.

**Findings:** The stakeholder panel identified two feasible implementation strategies: 1) including SNAP referral as part of the workflow of a food bank sponsored program providing fresh fruits and vegetables to community members; 2) including SNAP enrollment as part of Medicaid re-enrollment at the end of the COVID-19 public health emergency. Strategy 1 reached 50 participants over the 4 month implementation period, with 7 successfully enrolled in SNAP. The most common reason for non-enrollment was ineligibility due to immigration status. Strategy 2 had an unmeasurable outcome, as Medicaid enrollment staff did not track SNAP referrals that were directed to the state SNAP specialists.

**Implications for D&I Research:** While identifying opportunities to engage likely eligible individuals for SNAP enrollment produced feasible strategies, success was limited to due difficulties with state agency data and eligibility screening. Future implementation and dissemination strategies must engage both state entities and individuals with lived experience to ensure data and eligibility issues are included in any SNAP outreach implementation design.

**Primary Funding Source:** National Institutes of Health

### S94 Identifying and engaging policy networks to co-create sustainment strategies

#### Erika Crable^1^, Ryan Kenneally^2^, Gregory Aarons^3^

##### ^1^University of California, San Diego, La Jolla, CA, USA; ^2^UC San Diego, Child and Adolescent Services Research Center, San Diego, CA, USA; ^3^ImplementatioN Science and Team Effectiveness in Practice (IN STEP) Children's Mental Health Research Center, San Diego, CA, USA

###### **Correspondence:** Erika Crable (ecrable@health.ucsd.edu)

*Implementation Science 2024*, **19(2):**S94

**Background:** The Leadership and Organization Change for Implementation-Systems Level (LOCI-SL) implementation strategy aims to foster collaborations across clinic-organization-payer-policymaker levels to align initiatives and build cross-level implementation climates to support sustainment of motivational interviewing (MI) and an artificial intelligence quality assurance platform (Lyssn.io) across substance use treatment clinics in Oregon. Sustainment planning requires understanding how multilevel outer context policy networks (linkages between actors in the state government, advisory boards, payers, professional associations) and inner context provider organizations consider real-time implementation data, evidence-based practice goals, competing initiatives, and resource constraints.

**Methods:** The Exploration, Preparation, Implementation, Sustainment (EPIS) framework guided our consideration of implementation and engagement processes across levels of outer/inner contexts and “bridging factors” that align strategies that span policy to practice. We conducted a multi-step and multi-level process for engaging actors: (1) Exploration phase landscape policy analysis (document review, informational and qualitative interviews) to identify cross-context policy networks that influence MI+Lyssn sustainment, (2) top-down outreach and sustainment planning with state government and payers, (3) bottom-up engagement with provider organizations. During Preparation and Implementation, researchers explored (in)formal partnership approaches to promote ongoing communication, data sharing, and sustainment planning. Researchers also facilitated brainstorming meetings with payers and provider organization leaders to co-create strategies to achieve Sustainment phase goals.

**Findings:** Qualitative analyses highlighted turnover and state leadership barriers to engagement in sustainment efforts. For one partner, a memorandum of understanding promoted ongoing formal partnership during leadership turnover. State leadership barriers resulted in another partner preferring informal partnerships and information sharing between researchers and street-level bureaucrats during sustainment planning. Policy analysis revealed differential spheres of influence for each policy entity. Policy actors and researcher relationships were bi-directional. Researchers provided partners qualitative reports of policy network relationships and influence, and MI+Lyssn progress. Policy entities provided multi-level buy-in, insights on opportunities to align MI+Lyssn sustainment and state quality goals.

**Implications for D&I Research:** This presentation highlights approaches for understanding and engaging with policy-level factors and actors that are underreported on but critical to advancing policy-focused D&I efforts. We describe approaches for identifying and engaging with individual policy entities and policy networks to achieve sustainment goals.

**Primary Funding Source:** National Institutes of Health

### S95 Engaging policy actors to co-design a policy implementation study after policy implementation: Case study of a state-wide substance use service expansion policy roll-out

#### Gracelyn Cruden^1^, Erika Crable^2^

##### ^1^Chestnut Health Systems, Eugene, OR, USA; ^2^University of California, San Diego, La Jolla, CA, USA

###### **Correspondence:** Gracelyn Cruden (gcruden@chestnut.org)

*Implementation Science 2024*, **19(2):**S95

**Background:** New state-level policies (e.g., laws) are passed at least annually. Given the volume of new policies, implementation scientists cannot always be at the table prior to policy design (*Exploration* in the EPIS framework) or roll-out (*Preparation, Implementation)*. Implementation scientists can strategically engage relevant policy actors throughout policy implementation phases. This presentation describes policy actor engagement strategies used to co-design a state-wide policy implementation study when the policy was early in *Implementation*. The policy decriminalized possession of personal amounts of substances and allocated over $300 million to expand substance use services.

**Methods:** Policy actors from the state organization responsible for implementing the new policy were first engaged to understand the policy context and upcoming implementation steps to identify which steps could most appropriately and acceptably be supported through research. Next, policy actors at other state agencies (e.g., justice department), local actors (e.g., substance use service providers, law enforcement), and state-wide actors (e.g., payers, advocates) were engaged to understand their policy attitudes, implementation activities to-date, and planned activities. Engagement activities included monthly phone calls, networking at the state public health conference, unprompted and regularly-scheduled phone calls, grant document sharing (for both receiving direct edits and for transparency), and local law enforcement meet n’ greets and ride-alongs.

A novel implementation strategy will be co-developed and tested. The strategy’s goal is to support daily policy implementation decisions by providing primary and secondary data to implementers through data dissemination products that are prioritized and co-designed with policy actors.

The study will overlap with the *Preparation* phase for modifications to the original policy and within the original policy’s *Implementation* and *Sustainment* phases.

**Findings:** Targeted implementation outcomes include *penetration* of the policy across state counties (e.g., use of a new class of misdemeanor citations created through the original policy, use of data products to inform substance use service delivery decisions and policy development). Service outcomes include collaboration between policy actors — a hypothesized mechanism of impact on client outcomes (e.g., opioid-related overdoses, service satisfaction).

**Implications for D&I Research:** The iterative nature of policy development and roll-out, combined with policy actors’ concurrent involvement in multiple policies, begets opportunities for policy implementation research.

### S96 Establishing sustainable policies to improve HIV care for the community health workforce: Lessons from Shelby County, TN

#### Serena Rajabiun^1^, Brandon Williams^2^, Melissa HIrschi^3^, Abigail Smack^2^, Chandni Shahdev^1^, Robin Lennon-Dearing^2^

##### ^1^University of Massachusetts, Lowell, Lowell, MA, USA; ^2^University of Memphis, Memphis, TN, USA; ^3^Utah Valley University, Drem, UT, USA

###### **Correspondence:** Brandon Williams (bbwillia@memphis.edu)

*Implementation Science 2024*, **19(2):**S96

**Background:** In 2020, Shelby County Ryan White Program community leaders launched *End 901 HIV Plan* to reduce new infections and improve access to care and treatment for people with HIV according to the *National Strategy to Ending the HIV Epidemic by 2030.* One objective was to expand the use of Community Health Workers (CHWs) to improve viral suppression. Guided by the EPIS-RE-AIM frameworks, organizational and county leaders, people with HIV and CHWs designed and implemented an organizational capacity building intervention to create new and enhance existing policies to standardize procedures and integrate the CHW workforce in HIV primary care.

**Methods:** We used the Program Sustainability and Assessment Tool to survey organizational staff, program and policy leaders on sustainment of changes in CHW infrastructure and policy. Survey elements included: the implementation of skills and knowledge, agency and county support for the CHW workforce, funding, and organization capacity to integrate CHWs. Open ended questions were added to describe examples of sustainment benefits and challenges of the CHW workforce, and policy changes. Findings were disseminated and shared at a community wide meeting in June 2023 and policy successes and opportunities for workforce enhancement were identified and documented.

**Findings:** 25 organizational staff and county health leaders participated in the survey and dissemination meeting. Majority of participants reported their agency’s continued use of CHWs to connect people to HIV and gender affirming care, educate on medication adherence and HIV disclosure and building healthy relationships. At the organizational level, policies were established for supervision, having a champion and access to client data. Benefits were establishing a network of referral services and creating partner networks for addressing client social and medical needs. Sustainment challenges included staff and leadership turnover, lack of decisions on standardized CHW roles and implementation delays with a county-wide referral system. Health department leadership in coordinating partnerships and implementing policies changes were identified as critical to the sustainment process.

**Implications for D&I Research:** Policy sustainment for the CHW workforce in HIV primary care requires organizational champions and community leaders who have the historical knowledge, decision-making power and ability to advocate for and coordinate networks.

**Primary Funding Source:** Providence/Boston Center for AIDS Research (CFAR), NIH grant P30AI042853.

### S97 Implementing policies that earmark taxes for mental health services: Determinants of positive impacts and increasing reach of evidence-based practices

#### Jonathan Purtle^1^, Nicole Stadnick^2^, Jonathan Purtle^3^, Sarah Walker^4^, Eric Bruns^5^, Gregory Aarons^6^

##### ^1^New York University, New York, USA; ^2^University of California San Diego, La Jolla, CA, USA; ^3^New York University School of Global Public Health, New York, NY, USA; ^4^University of Washington, Seattle, WA, USA; ^5^UW SMART Center, UW SMART Center, Seattle, WA, USA; ^6^UC San Diego Child and Adolescent Services Research Center, San Diego, CA, USA

###### **Correspondence:** Jonathan Purtle (jonathan.purtle@NYU.edu)

*Implementation Science 2024*, **19(2):**S97

**Background:** States and counties are increasingly adopting polices which earmark tax revenue for mental health services. No prior research, however, has examined which determinants may be the most promising targets for implementation strategies that seek to ensure that these policies generate positive impacts and increase the reach of evidence-based practices (EBPs). This study sought to identify such determinants using survey data from professionals involved with earmarked tax policy implementation.

**Methods:** Web-based surveys of public agency and community organization professionals involved with earmarked tax implementation were conducted in 2022-2023 (N=274, response rate=28.7%). The primary dependent variable was a composite score (α=.70), derived from nine items, which quantified the extent to which the tax was perceived as generating positive impacts. The secondary dependent variable was a single item which quantified the extent to which the tax was perceived as increasing the reach of EBPs. Independent variables were composite scores that mapped to determinant constructs in the Exploration, Preparation, Implementation, and Sustainment framework. The determinants were: perceptions of tax policy attributes (innovation determinant), implementation climate for the tax supporting EBPs, (inner-setting), frequency of inter-agency collaboration in tax implementation (outer-setting, cosmopolitanism), and level of support for the tax across constituent groups (outer-setting, peer-pressure). Separate multiple linear regression models were created that adjusted demographic covariates.

**Findings:** The mean positive tax impact score was 46.75 (range=9-63, SD=8.64) and the mean increase reach of EBP score was 5.69 (range=1-7, SD=1.51). Favorable perceptions of tax policy attributes (β=0.43, p<.0001) and positive implementation climate for the tax supporting EBPs (β=0.38, p<.0001) were significantly associated with more positive perceptions of tax impact. When perceptions of the extent to which the tax increased the reach of EBPs served as the department variable, perceptions of tax attributes (β=0.26, p=.01) and implementation climate (β=0.24, p=.01) remained significantly associated but the magnitude of these associations were smaller.

**Implications for D&I Research:** Strategies which target implementation climate for earmarked taxes supporting EBPs have potential to positively affect the impacts of these taxes and improve the reach of EBPs. Strategies which alter tax policy attributes could produce similar effects, but may theoretically be more challenging to deploy.

**Primary Funding Source:** National Institutes of Health

### S98 Using implementation science (IS) frameworks and strategies to support implementation of health and wellness-related policies in Chicago public schools: The healthy CPS network specialist strategy

#### Jamie Chriqui^1^, Elizabeth Jarpe-Ratner^1^, Julien Leider^1^, Yu Chen Lin^1^, Madison Offstein^1^, Cassidy Malner^2^, Lauren Pett^2^, Jeremiah Simon^2^, Sean Zolfo^2^, Jamie Tully^2^, Tarrah DeClemente^2^

##### ^1^University of Illinois Chicago School of Public Health, Chicago, IL, USA; ^2^Chicago Public Schools, Chicago, IL, USA

###### **Correspondence:** Jamie Chriqui (jchriqui@uic.edu)

*Implementation Science 2024*, **19(2):**S98

**Background:** Chicago Public Schools (CPS; 4th largest US school district) are subject to >50 federal, state, and local health and wellness-related policies, many involving unfunded mandates. Implementing these policies is a challenge, particularly for under-resourced schools. CPS’ Office of Student Health and Wellness, with the University of Illinois Chicago PRC, conducted an implementation efficacy trial to test a Healthy CPS Network Specialist implementation strategy on implementation- and school-level outcomes.

**Methods:** The Specialist implementation strategy was fully launched in school year (SY) 2020-21 and leverages the ERIC^1^ and SISTER^2^ implementation strategy frameworks. Key Proctor^3^ implementation outcomes included: reach and cost (compiled from administrative data and an activity-based time tracking log^4^, respectively); while acceptability, appropriateness, feasibility, fidelity, and adoption were assessed through stakeholder discussion groups/interviews and the annual Healthy CPS Survey, which assesses school practices/compliance with the 50+ policies. A difference-in-difference (DID) analysis examined the relative change in overall Healthy CPS scores (from 2018-19 to 2022-23) for the Specialist Network vs. a matched comparison Network. Cost-effectiveness was calculated by: Specialist’s school-specific costs/ estimated DID impact.

**Findings:** On average, the Specialist served 26 schools with ~7500 students (reach) at a cost of ~$12.20/student between SY 20-21 and 22-23. Qualitative data elucidated stakeholders' perceptions of the acceptability (“a win-win” for schools); appropriateness (“definitely needed,” “more attuned to needs than we are”); feasibility (“shared resources”); and fidelity and adoption (i.e., Specialist provided more intense implementation strategies based on school needs). Schools in the Specialist’s Network maintained their Healthy CPS scores over time, including during the pandemic (adj. mean 72% pre vs. 76% post), whereas the comparable Network experienced a significant decline over time (74% pre vs. 68% post; p<.05 for change over time and for DID impact). On average, the Specialist strategy costs $372/school to increase their Healthy CPS score by 1 percentage point.

**Implications for D&I Research:** Using IS frameworks and strategies to support schools is a cost-effective approach for supporting school health and wellness-related policy implementation.

**Primary Funding Source:** Centers for Disease Control and Prevention

**References:**

1. Powell et al. *Impl Sci.*, 2015
2. Cook et al., *Prev. Sci.* 2019
3. Proctor et al., Adm Policy Ment Health, 2011
4. Cidav et al., *Impl Sci*, 2020

### S99 Effective financing strategies to support evidence-based treatment implementation: Case comparison analyses of federal grant funding across 18 states

#### Sarah Hunter^1^, Alex Dopp^1^, Matthew Lee^2^

##### ^1^RAND Corporation, Santa Monica, CA, USA; ^2^NYU Grossman School of Medicine, New York, NY, USA

###### **Correspondence:** Sarah Hunter (shunter@rand.org)

*Implementation Science 2024*, **19(2):**S99

**Background:** Financing robust support to implement high quality adolescent substance use treatment is a vexing issue. Treatment organizations primarily rely on state funding that is often derived from federal grants. Starting in 2012, the U.S. Substance Abuse and Mental Health Services Administration (SAMHSA) initiated a series of grant opportunities to improve the state-level infrastructure to support the delivery of evidence-based adolescent substance use disorder treatments, with the Adolescent Community Reinforcement Approach (A-CRA) as one of the most frequently supported treatments. Our project examined the extent to which these grants, as one type of financing strategy, led to staff-level reach of A-CRA, that is, the proportion of providers certified to deliver and/or supervise A-CRA across treatment organizations within the 18 states who obtained SAMHSA funding.

**Methods:** Using a longitudinal mixed-methods comparative case study design, we examined infrastructure and treatment capacity-building elements among states with high versus low staff-level reach rates. Our data sources included documentation of clinician certification records to assess reach, documents collected about each state’s adolescent treatment delivery efforts, and interviews with state substance use service agency administrators following the end of their grants.

**Findings:** We found few differences between states with high versus low rates of A-CRA staff-level reach in terms of states’ financial mapping documentation, inter-agency coordination, and state administrative staff perspectives about the treatment’s effectiveness. States with higher reach appeared to have greater intent to invest in implementation, as demonstrated by support for clinical assessment monitoring and state agency staff persistence to continue delivery despite funding challenges. States with lower reach expressed challenges with treatment staff turnover and “fit” between the treatment model and how care is delivered in their state (e.g., perceived treatment was too long or emphasized community-delivered care too much).

**Implications for D&I Research:** This research used a novel mixed-methods approach to conduct policy-focused implementation research on financing strategies across 18 states. The findings help delineate differences in policies, capacities, and infrastructure between high and low performing states. These findings have implications for how future grant initiatives and policies can help support the financing and delivery of evidence-based substance use treatments.

**Primary Funding Source:** National Institutes of Health

## Models, Measures, and Methods

### S100 Developing the business case of implementing evidence-based interventions to improve colorectal cancer screening uptake: Overview of methodology used in CDC’s colorectal cancer control program

#### Sujha Subramanian^1^, Florence Tangka^2^, Sonja Hoover^1^

##### ^1^Implenomics, Dover, DE, USA; ^2^Centers for Disease Control and Prevention, Atlanta, GA, USA

###### **Correspondence:** Sujha Subramanian (sujha.subramanian@implenomics.com)

*Implementation Science 2024*, **19(2):**S100

**Background:**

The Colorectal Cancer Control Program (CRCCP) is a nation-wide program to increase colorectal cancer (CRC) screening uptake. The cost of the interventions is a key concern raised by many health systems in sustaining EBIs shown to be effective. In this presentation we describe an approach to develop the business case for implementing screening promotion activities by considering the budget impact and assessing affordability.

**Methods:**

For this assessment, we worked with a subset of 6 CRCCP recipients and 8 of their partner federally qualified health centers (FQHCs). First, we collected detailed activity-based cost of the EBI implementation by tailoring a validated cost data collection tool. We collected data on staff, salaries, and time spent on activities specific to the interventions and nonlabor costs (e.g., software, travel). We also collected data on CRC screening uptake and process measures. Second, we estimated the per person cost of implementing the EBIs, generated the short-term incremental intervention cost per person successfully screened and the long-term cost-effectiveness using a microsimulation model. Third, we estimated the budget required to implement and sustain the interventions at scale and compared this cost with activities already implemented for other chronic diseases. Affordability is this context is defined as the economic capacity of health systems to implement and sustain strategies and interventions.

**Findings:**

The short-term incremental intervention cost per person successfully screened ranged from $18.76 to $144.65. Most of the EBIs resulted in cost savings while all combination of EBIs implemented were cost-effective (less than $29,326 per life year saved). Furthermore, the EBIs were considered affordable by majority of the health systems and were less costly to implement that some other ongoing interventions (for example, diabetes prevention intervention could cost an average of $450 per patient).

**Implications for D&I Research:**

The comprehensive approach described for the CRCCP could be used to estimate cost, generate cost-effectiveness and assess affordability to develop the business case. A one-size-fits all approach is not possible and tailored assessments at the health systems levels

**Primary Funding Source:** Centers for Disease Control and Prevention

### S101 Pragmatic user-centered methods and tools for health equity-focused implementation science evaluated with the translational sciences benefits model

#### Kelly Aschbrenner^1^, Douglas Levy^2,3^, Dr. Stephen Bartels^4^, Karen Emmons^5^

##### ^1^Geisel School of Medicine at Dartmouth College, Lebanon, NH, USA; ^2^Massachusetts General Hospital, Boston, MA, USA; ^3^Harvard Medical School, Boston, MA, USA; ^4^Massachusetts General Hospital, Harvard Medical School, Boston, MA, USA; ^5^Harvard University, Boston, MA, USA

###### **Correspondence:** Kelly Aschbrenner (kelly.aschbrenner@dartmouth.edu)

*Implementation Science 2024*, **19(2):**S101

**Background:** The Implementation Research Methods Incubator (IRMI) at the Harvard Implementation Science Center in Cancer Control Equity (Harvard ISCCCE) addresses methodological challenges in implementation centered on health equity. Aligned with this year’s D&I Conference theme, this presentation provides an overview of IRMI-led methods and practical tools for health equity-focused implementation science applying a systematic evaluation of the real world impact of this work.

**Methods:** Our IRMI team at ISCCCE partnered with investigators and community health center implementation teams to develop low-burden and user-centered methods and tools to advance health equity-focused implementation research. These methods and tools include: (1) co-design of an implementation strategy to improve equity in cancer screening through rapid cycle testing of data-driven adaptations; (2) measuring effort devoted to implementation and intervention activities in resource-constrained settings; and (3) cross-center development of an online toolkit to prepare implementation scientists to engage in health equity-focused implementation research. We evaluated this effort focused on developing and disseminating research methods using the Translational Sciences Benefits Model (TSBM), a framework that facilitates assessment of research impact beyond traditional metrics to emphasize public health and social benefits.

**Findings:** IRMI-led methods and tools were estimated to have high potential for real world impact in three TSBM domains: Clinical and Medical Benefits, Community and Public Health Benefits, and Economic Benefits. Rapid cycle testing of data-driven adaptations designed to close disparity gaps in cancer screening can have downstream impact on reducing cancer inequities. Low burden methods for collecting time use data in resource-constrained settings can be used to evaluate Economic Benefits of research. Finally, a publicly available toolkit with guidance for implementation researchers on how to get started and engage in health equity-focused implementation research can impact Clinical and Medical Benefits and Community and Public Health Benefits by informing procedures and guidelines and health activities and products in the context of D&I Science.

**Implications for D&I Research:** Pragmatic, equity-focused implementation research methods and tools can result in public health and social benefits by ensuring research can be performed with partners in resource-constrained settings and can help decision makers to weigh the potential benefits and costs of research.

**Primary Funding Source:** National Institutes of Health

### S102 Development of a pragmatic measure for the practical, robust implementation and sustainability model

#### James Pittman^1,2^, Erin Almklov^1^, Borsika Rabin^1,3^, John Gault^1^, Brian Blanco^1^, Laurie Lindamer^1^

##### ^1^Veterans Health Administration, VA Center of Excellence for Stress and Mental Health, San Diego, CA, USA; ^2^University of California San Diego, San Diego, CA, USA; ^3^UC San Diego Altman Clinical and Translational Research Center, San Diego, CA, USA

###### **Correspondence:** James Pittman (james.pittman@va.gov)

*Implementation Science 2024*, **19(2):**S102

**Background:** There is a need for pragmatic quantitative measures of contextual factors to promote comparison across sites or time. We developed and evaluated a quantitative survey based on the Practical Robust Implementation and Sustainability Model (PRISM) as part of a mixed-method, hybrid type-II trial to evaluate the implementation and effectiveness of a web-based screening tool in VA settings. The purpose of this study is: 1) To describe the PRISM survey instrument development and preliminary psychometric properties, and 2) To demonstrate its usefulness rapidly quantifying contextual domains to inform implementation and sustainment.

**Methods:** The 29 item survey was developed based on an existing tool, expert feedback, and iterative pilot testing. Three to six items for each of the six PRISM domains (i.e., Organizational Perspective, Patient Perspectives, Infrastructure, Organizational Characteristics, Patient Characteristics, and External Environment) were included, each rated on a five-point Likert scale. Eight VA Healthcare Systems stratified by rurality, staffing level, and patient volume, with an interest in implementing eScreening at their facility participated. During the pre-implementation phase, 30 staff completed the PRISM survey and another quantitative measure of implementation outcomes to establish concurrent validity.

**Findings:** The final survey took 14 minutes to complete. The mean overall score for the 29-item survey across participants and sites was 3.95 (.42) and mean PRISM domain scores across participants and sites ranged from 3.5 (.40) for Patient Characteristics to 4.1 (.22) for Organizational Characteristics. Properties: Internal consistency for the subscales ranged from .51 to .82; and concurrent validity with the other implementation outcomes - Weiner scales - varied from r = .696, p < .001 for feasibility to r = .796, p < .001 for appropriateness. We will provide examples of how pre-implementation survey results we used to identify areas to target for improvement and discuss other uses.

**Implications for D&I Research:** PRISM, a widely used implementation science framework, was converted to a survey that has exhibited good psychometric and pragmatic properties and demonstrated variability over several clinic sites involved in the implementation project. This tool could be useful for implementation projects to plan, develop implementation strategies, and evaluate/predict implementation success.

**Primary Funding Source:** Department of Veterans Affairs

### S103 Raising expectations for rapid qualitative implementation efforts: Guidelines to ensure rigor in rapid qualitative study design, conduct, and reporting

#### Christine Kowalski^1^, Andrea Nevedal^1^, Erin Finley^2,3^, Jessica Young^4^, Allison Lewinski^5,6^, Amanda Midboe^7,8^, Alison Hamilton^3,9^

##### ^1^Veterans Health Administration, VA Ann Arbor Healthcare System, Ann Arbor, MI, USA; ^2^University of Texas Health San Antonio, San Antonio, USA; ^3^Veterans Health Administration, VA Greater Los Angeles Healthcare System, Center for the Study of Healthcare Innovation, Implementation & Policy (CSHIIP),Los Angeles, USA; ^4^VA Puget Sound Health Care System, Seattle, WA, USA; ^5^Duke University School of Nursing, Durham, NC, USA; ^6^Veterans Health Administration, Durham VA Health Care System, Durham, NC, USA; ^7^Stanford University School of Medicine, Stanford, CA, USA; ^8^Veterans Health Administration, Center for Innovation to Implementation (Ci2i), VA Palo Alto Health Care System, Menlo Park, CA, USA; ^9^UCLA David Geffen School of Medicine, Los Angeles, USA

###### **Correspondence:** Christine Kowalski (christine.kowalski@va.gov)

*Implementation Science 2024*, **19(2):**S103

**Background:**

Rapid qualitative methods have taken shape over the past decade and are now being utilized across numerous implementation efforts and reported in well over a hundred publications. This brisk increase begs the questions: what does rigor entail in rapid qualitative methodology? How do we define pragmatic standards to help research teams design and conduct rigorous and valid rapid studies? How can authors articulate rigor in their methods descriptions? Lastly, how can manuscript reviewers evaluate the rigor of qualitative work that involved rapid methods?

**Methods:**

As part of the Veterans Health Administration’s Rapid Qualitative Quality Enhancement Research Initiative (QUERI) Learning Hub, seven experts with over 100 years of collective expertise developed consensus guidelines for rigor in rapid qualitative methods. We reviewed literature, standard procedures across multiple qualitative teams, and a repository of rapid qualitative training materials to create a table of guidelines, which was iteratively refined through a series of group working meetings. We then circulated the draft amongst authors to invite feedback on items and ensure completeness, clarity, and comprehensibility of guidelines.

**Findings:**

The resulting consensus guidelines support rigor and validity in rapid qualitative work by supporting rapid qualitative study design, conduct, write-up, and review. The consensus guidelines offer a structured checklist covering rigorous design, data collection, summary creation, rapid qualitative analysis, and rapid qualitative data synthesis, as well as offering examples and important considerations to support continuous improvement in the rigor of rapid qualitative methods.

**Implications for D&I Research:**

A key criticism of traditional qualitative methods is the time-intensive nature of the process. By contrast, rapid qualitative methodology is pragmatic and can be utilized even in time-and resource-constrained studies. These methods have gained traction in implementation research and practice, wherein real-time adjustments are often made to optimize processes and outcomes. Rapid qualitative methods play a central role in implementation evaluations, yielding critical in-depth information and insights about context, process, and relationships.

These consensus guidelines fill a gap in the literature by offering standardized evaluation criteria to facilitate planning and conduct of rapid qualitative studies. In so doing, these guidelines provide an expert-driven resource to support high-level methodological rigor in real-world implementation science.

**Primary Funding Source:** Department of Veterans Affairs

### S104 Developing concurrent validity for the clinical sustainability assessment tool (CSAT) in global settings

#### Virginia Mckay^1^, Yichen Chen^2^, Kim Prewitt^3^, Sara Malone^4^, Maria Puerto Torres^5^, Adolfo Cardenas-Aguirre^5^, Meenakshi Devidas^2^, Douglas Luke^6^, Asya Agulnik^7^

##### ^1^School of Social Work, Washington University in St. Louis, St. Louis, MO, USA; ^2^St. Jude Children’s Research Hospital, Memphis, USA; ^3^Washington University in St. Louis, St. Louis, MO, USA; ^4^Department of Surgery (Division of Public Health Sciences) and Alvin J. Siteman Cancer Center, Washington University School of Medicine, St. Louis, MO, USA; ^5^St. Jude Children's Research Hospital, Memphis, TN, USA; ^6^Center for Public Health Systems Science, Washington University in St. Louis, Saint Louis, MO, USA; ^7^St. Jude Children’s Research Hospital, Memphis, TN, USA

###### **Correspondence:** Virginia Mckay (virginia.mckay@wustl.edu)

*Implementation Science 2024*, **19(2):**S104

**Link:** https://link.springer.com/article/10.1007/s43477-023-00106-2

### S105 Simulation modeling as a community level decision support tool

#### Kimberly Johnson^1^, C. Hendricks Brown^2^, Holly Hills^3^, Lia Chin-Purcell^4^, Wouter Vermeer^2^

##### ^1^University South Florida, Tampa, FL, USA; ^2^Northwestern University Feinberg School of Medicine, Chicago, IL, USA; ^3^University of South Florida, Tampa, FL, USA; ^4^Stanford University School of Medicine, Palo Alto, CA, USA

###### **Correspondence:** Wouter Vermeer (wouter.vermeer@northwestern.edu)

*Implementation Science 2024*, **19(2):**S105

**Background:**

Implementation of strategies to improve population health happens at the county level in most of the country. Many counties have used federal resources to develop a data driven response to reducing morbidity and mortality but find themselves tracking an increasing rates in exquisite detail without knowing how to translate epidemiological data to community action. Using C-DIAS common frameworks, measures, and methods, this project evaluates decision support tools using simulation modeling to help communities rationalize their process of selecting interventions. Simulation modeling may support improved decision making at the community level by allowing community leaders to ask “what if” questions using their own local data.

**Methods:**

The progress of three counties is examined through the first three stages of implementation completion at three levels (community development, data coordination, model development). Key informant interviews provide an understanding of the perceived barriers and facilitators of implementing modeling tools and making decisions at the community level.

**Findings:**

In this first year of C-DIAS, community leadership in three counties has successfully been engaged. Cycles of simulation experiments for Pinellas County have been conducted using agent-based modeling to help make decisions about Narcan distribution and treatment expansion. Initial modeling with Vermillion County has identified potential locations for mobile services. Santa Clara County has been engaged in discussions about the type of decisions modeling may help them make. Baseline qualitative data has been collected and common implementation issues across the sites have been identified. Output from the modeling experiments and baseline qualitative interviews will be shared during this symposium. Baseline county data and preliminary data on perceived barriers and facilitators collected via qualitative interviews and team based organizational measures will also be presented.

**Implications for D&I Research:**

Johnson will discuss how the use of C-DIAS common measures and frameworks will produce translatable lessons about simulation modeling to support communities in making resource allocation decisions in a timely and rational manner. As an innovation itself, implementing simulation modeling requires a multilevel implementation strategy focused on community engagement.

**Primary Funding Source:** National Institutes of Health

### S106 Evaluating a multi-level implementation strategy to install contingency management in opioid treatment programs

#### Sara Becker^1^, Kira DiClemente-Bosco^1^, Kelli Scott^1^, Kimberly Yap^1^, Fariha Hasan^1^, Sarah Salino^1^, Bryan Garner^2^

##### ^1^Northwestern University Feinberg School of Medicine, Chicago, IL, USA; ^2^The Ohio State University, Columbus, OH, USA

###### **Correspondence:** Sara Becker (sara.becker@northwestern.edu)

*Implementation Science 2024*, **19(2):**S106

**Background:**

Contingency management (CM) is one of the most effective interventions for stimulant use and is a highly effective adjunct to medication for opioid use disorder but is rarely offered in opioid treatment programs (OTPs). The gap between the overwhelming evidence for CM and its minimal uptake in OTPs represents one of the greatest research-to-practice gaps in health services. Addressing this gap requires the specification and evaluation of implementation strategies that can address barriers to CM uptake at provider- and organization-levels. Using C-DIAS common frameworks, measures, and methods, a type 3 hybrid effectiveness-implementation trial was designed to evaluate a multi-level implementation strategy, the Science to Service Laboratory, to implement CM targeting stimulant use in OTPs.

**Methods:**

We initially proposed a fully powered stepped wedge trial with 10 OTPs randomized to receive the Science of Service Laboratory over five time points spanning 50 months. At six intervals, each of the OTPs will provide de-identified electronic medical record data on common C-DIAS implementation outcomes. OTP staff will also complete a series of surveys and in-depth interviews documenting common C-DIAS contextual determinants influencing implementation.

**Findings:**

After notice of grant award, adaptations to the protocol were necessary following California’s announcement that it would fund CM as a reimbursable Medicaid service. This shift in the outer setting resulted in multiple Departments of Health planning state-wide CM rollouts and OTPs being unwilling to be randomized to delayed CM implementation support. We therefore consolidated our stepped-wedge trial to 30 months and, in parallel, launched a naturalistic evaluation of a state-wide rollout of CM, using the same C-DIAS frameworks, measures, and methods. To date, we have launched Science to Service Laboratory support with six OTPs and collected baseline data on provider- (CM attitudes, CM knowledge, provider demographics) and organization-level (implementation climate, leadership engagement) determinants from 78 OTP staff.

**Implications for D&I Research:**

Harmonization across the stepped wedge trial and natural evaluation via the C-DIAS frameworks, measures, and methods will produce translatable lessons to promote the implementation of CM in OTPs at scale.

**Primary Funding Source:** National Institutes of Health

### S107 An effectiveness-implementation trial of a care navigation intervention to address barriers to care and increase initiation of substance use disorder treatment

#### Joseph Glass, Theresa Matson, Chayana Davis, Deborah King, Jennifer Bobb

##### Kaiser Permanente Washington Health Research Institute, Seattle, WA, USA

###### **Correspondence:** Joseph Glass (Joseph.E.Glass@kp.org)

*Implementation Science 2024*, **19(2):**S107

**Background:**

More information is needed on how to implement innovations in health systems to help patients successfully initiate care when referred to specialty treatment. Using C-DIAS common frameworks, measures, and methods, this cluster-randomized effectiveness-implementation trial evaluates the integration of a care navigation intervention within an integrated health system’s behavioral health triage center that processes and/or authorizes referrals for substance use disorder (SUD) care in the community or within the healthcare system. This study also seeks to improve equity in treatment initiation.

**Methods:**

Care navigation seeks to enhance patient motivation to follow-up on their treatment plan, reduce barriers to accessing treatment, and advocate for patients to reduce disparities. SUD treatment initiation will be compared across triage center staff who are randomized to offer care navigation versus not. Multilevel implementation strategies will be used. To unpack the “black box” of implementation, this study will examine mechanisms via which the intervention and implementation strategy exert their impact on implementation and effectiveness outcomes.

This study was initially designed as an implementation trial in primary care to improve the implementation and sustainment of a smartphone-based prescription digital therapeutic for SUD. During the first year of funding, two major changes made it infeasible to conduct the proposed study: 1) the health system accumulated staffing vacancies in primary care, and 2) the digital therapeutic vendor filed for bankruptcy. The re-designed study builds on an active collaboration with health care system partners and C-DIAS and will enhance implementation science while testing the effectiveness of interventions to improve treatment initiation among patients with SUD.

**Findings:** Qualitative data, collected from patients and providers using the C-DIAS framework and analyzed with a rapid group analysis process, will describe the determinants faced by patients with different lived experiences and resources after they are referred to SUD treatment, the type and level of support necessary to improve treatment initiation, and the mechanisms by which care navigation could achieve this goal. User-centered design methods will identify strategies to support implementation of care navigation in routine care.

**Implications for D&I Research:** Glass will discuss how the C-DIAS frameworks, measures, and methods will produce translatable lessons, across disciplines, from this trial.

**Primary Funding Source:** National Institutes of Health

### S108 A comparative implementation trial: Stagewise implementation-to-target medications for addiction treatment (SITT-MAT)

#### Jay Ford II^1^, Hannah Cheng^2^, Michele Gassman^3^, Kimberley Mount^4^, Ryan Keith^4^, Tony Walton^4^, Harrison Fontaine^5^, Hélène Chokron Garneau^6^, Mark McGovern^6^

##### ^1^University of Wisconsin-Madison, Madison, WI, USA; ^2^Stanford University School of Medicine, Palo Alto, USA; ^3^University of Wisconsin - Madison, Madison, WI, USA; ^4^Washington State Healthcare Authority, Olympia, WA, USA; ^5^Washington State Health Care Authority, Olympia, WA, USA; ^6^Stanford University School of Medicine, Palo Alto, CA, USA

###### **Correspondence:** Jay Ford II (jhfordii@wisc.edu)

*Implementation Science 2024*, **19(2):**S108

**Background:**

Expert recommendations including tailored strategies, strategy engagement and fidelity, and economic analysis, have not been evaluated in primary care and specialty addiction treatment programs to address challenges of implementing and sustaining medications for opioid use disorder (MOUD). Using C-DIAS common frameworks, measures, and methods, the SITT-MAT project addresses this gap by employing a measurement-based stepped implementation-to-target approach within an adaptive trial design to improve access to MOUD.

**Methods:**

Participating programs are exposed to a sequence of implementation strategies of increasing intensity and cost: 1) enhanced monitoring and feedback; 2) two-day workshop; and then, if outcome targets are not achieved, randomization to either Internal Facilitation or External Facilitation. Outcomes are organized by the RE-AIM taxonomy. Study aims are to: (1) evaluate the relative impact of implementation strategies on target outcomes; (2) examine contextual moderators and mediators of performance; and (3) document and model costs per implementation strategy using the Stages of Implementation Completion (SIC).

**Findings:**

Fifty-five clinics, 32 specialty addiction (SAC) and 23 primary care (PCC), agreed to participate. Baseline characteristics differ by clinic type. A goal of all PCCs is to expand MOUD versus 60% of SACs. While SACs have more OUD clients, only 44% were prescribed MOUD versus 61% of PCC clients. At baseline, PCCs report higher IMAT scores (3.62 versus 3.17) than SAC as well as reach and access outcomes. Data trends associated with IMAT scores, patients prescribed MOUD (overall and within 72 hours), and patient linkages will be shared. We will examine these trends overall, by clinic type, and by MOUD focus (start-up or expansion).

**Implications for D&I Research:**

Real world implementation does not align with proposed plans as actual implementation requires adaptation. In SITT-MAT, SAC recruitment was impacted by research oversaturation, workforce depletion, and staff turnover which necessitated open recruitment of PCCs and delayed program engagement. Enhanced monitoring and feedback was not a low intensive strategy requiring changes to simplify collection of reach and effectiveness outcome data. SIC development required mapping across and sequencing between all four implementation strategies. To be successful, implementation researchers must recognize and adapt their approaches to address real-world challenges.

**Primary Funding Source:** National Institutes of Health

### S109 Application of innovation tournament methods to inform patient, provider and system-level de-implementation strategies for reducing mammography overscreening among older women

#### Rachel Shelton^1^, Nathalie Moise^2^, Savannah Alexander^1^, Emmy Burroughs^1^, Anita Karr^1^, Parisa Tehranifar^1^

##### ^1^Columbia University Mailman School of Public Health, New York, NY, USA; ^2^Columbia University Irving Medical Center, New York, NY, USA

###### **Correspondence:** Rachel Shelton (rs3108@cumc.columbia.edu)

*Implementation Science 2024*, **19(2):**S109

**Background:** There is a need to advance the science of de-implementation. Mammography screening in women 75 and older presents a robust area for inquiry related to de-implementation, as routine screening can lead to potential harms (e.g., overdiagnosis, overtreatment) while health benefits remain uncertain. Most guideline organizations do not support routine mammography in older women with average breast cancer risk and suggest consideration of preferences, health, and life expectancy. However, intersecting patient, provider, and system factors promote and reinforce over-screening among older women.

**Methods:** Guided by de-implementation frameworks and methodological approaches from implementation studies, we conducted an online innovation tournament to identify de-implementation strategies at the patient, provider, and system levels for reducing mammography overuse in older women. The Rethink Mammography Innovation Tournament (November 2022 to May 2023) brought together medical providers and administrators from two large primary care systems serving racially/ethnically diverse women in New York City to explore and exchange ideas about mammography among women 75 and older. Participants completed surveys and engaged in an online discussion forum through posts (e.g., of stories, de-implementation ideas), comments, and/or up/down votes on posts/comments.

**Findings:** 47 participants completed surveys and accessed the discussion forum, with 36 actively engaging through posts, comments and/or votes. Participants were predominantly primary care providers (PCP; n=25) and included radiologists, breast surgeons, oncologists, OB/GYN, geriatricians, and administrators. Participants posted 13 ideas/strategies and submitted 71 comments and 256 votes (up/down votes on posts/comments). While 58% of participants strongly agreed with guidelines, agreement varied by provider type (67% among PCPs vs. 38% among radiologists). Using a content analysis of forum data, we identified 5 overarching categories of potential de-implementation strategies: 1) patient-facing community education; 2) shared decision-making tools; 3) integrated EHR prompts; 4) provider training/education; and 5) system-level alignment of guidelines, exemplified by changes to appointment reminder letters for patients.

**Implications for D&I Research:** Findings advance understanding of methodological approaches to enhance understanding of the perceived acceptability, feasibility, and effectiveness of strategies for de-implementation of low-value care in clinical settings. Findings also inform understanding of synergies and distinctions between methods and strategies that are appropriate for de-implementation vs. implementation efforts.

**Primary Funding Source:** National Institutes of Health

### S110 A structured approach to adapting an implementation package while spreading a complex evidence-based practice

#### Kristina Cordasco^1,2^, Jenny Barnard^1^, Taylor Harris^1^, Tanya Olmos-Ochoa^1^, Lauren Hoffman^1^, Erica Fletcher^1^, Eleni Skaperdas^1^, Agatha Palma^1^, Sonya Gabrielian^1,3^, Matthew McCoy^4^, Erin Finley^1,5^

##### ^1^Veterans Health Administration, VA HSR&D Center for the Study of Healthcare Innovation, Implementation & Policy (CSHIIP), Los Angeles, CA, USA; ^2^David Geffen School of Medicine at UCLA, Los Angeles, CA, USA; ^3^Department of Psychiatry and Biobehavioral Sciences, UCLA David Geffen School of Medicine, Los Angeles, CA, USA; ^4^Veterans Health Administration, VA Greater Los Angeles Healthcare System, Center for the Study of Healthcare Innovation, Implementation & Policy (CSHIIP), Los Angeles, USA; ^5^UT Health San Antonio, San Antonio, TX, USA

###### **Correspondence:** Kristina Cordasco (kristina.cordasco@va.gov)

*Implementation Science 2024*, **19(2):**S110

**Background:** The Housing Transitions Quality Enhancement Research Initiative is implementing Critical Time Intervention (CTI)--a multifaceted evidence-based, time-limited case management practice for homeless-experienced persons--across 36 community-based (“Grant and Per Diem (GPD)”) homeless service agencies that are partnering with the Veterans Health Administration (VA). These agencies, spanning the nation, vary widely across multiple inner and outer contextual elements. In a rollout design with four sequential cohorts, we are using, and comparing the impacts of, two implementation strategies (Replicating Effective Programs (REP) versus REP augmented with facilitation [“enhanced REP”]) on implementation and effectiveness outcomes. We describe a structured approach to formatively evaluating and adapting an implementation package employing a rollout design.

**Methods:** We developed the initial CTI implementation package in the first cohort. With each cohort, to evaluate and inform adaptations to the implementation package, we used rapid qualitative methods, analyzing and integrating information from interviews with GPD case managers (n=49) and supervisors (n=33); periodic reflections with project implementation leaders (n=15), staff (n=21), and VA clinicians who liaison with the GPD providers (n=65); and project logs. Adaptations were selected and formulated via implementation leaders and practitioners, in collaboration with evaluators. We used the Framework for Reporting Adaptations and Modifications to Evidence-based Implementation Strategies (FRAME-IS) to describe and track adaptations.

**Findings:** After each implementation cohort, in response to variations in patient, clinician, organizational, and sociopolitical factors, we made serial adaptations to the content, training, context, and evaluation of REP and enhanced REP to increase CTI adoption and fidelity. Adaptations included adding and reordering elements, changing packaging, refining materials, and integrating further strategies; strategy core elements were preserved. Some adaptations were planned and proactive, others were reactive and unplanned.

**Implications for D&I Research:** Multiple rounds of adaptation are often needed to optimize practice translation when spreading complex evidence-based practices across heterogenic, resource-constrained settings. We offer a structured, pragmatic strategy for making data-driven adaptations, and discuss the opportunities, tensions, and tradeoffs of projects having simultaneous formative and summative evaluation aims.

**Primary Funding Source:** Department of Veterans Affairs

### S111 Using rapid cycle approaches to develop implementation strategies to improve the use of genetic testing in a diverse health system

#### Robert Schnoll^1^, Anna-Marika Bauer^1^, Anna Raper^2^, Benita Weathers^2^, Wenting Zhou^2^, Paul Wileyto^2^, Colin Wollack^2^, Rachel Shelton^3^, Marylyn Ritchie^2^, Katherine Nathanson^2^

##### ^1^Perelman School of Medicine, University of Pennsylvania, Philadelphia, PA, USA; ^2^University of Pennsylvania, Philadelphia, PA, USA; ^3^Columbia University Mailman School of Public Health, New York, NY, USA

###### **Correspondence:** Robert Schnoll (schnoll@pennmedicine.upenn.edu)

*Implementation Science 2024*, **19(2):**S111

**Background:** The number of medical conditions for which genetic testing is indicated to manage patient treatment is increasing. Yet, a minority of eligible patients receive genetic testing to help manage their care. System- (methods to identify eligible patients and return results), clinician- (e.g., knowledge), and patient- (e.g., concerns about costs and adverse effects) level barriers to using genetic testing to manage treatment fosters uncertainty, which undermines willingness to integrate testing into clinical practice. Prior to launching a clinical trial to test implementation strategies to promote clinician referral or ordering of genetic testing across 10 medical conditions, we are using rapid cycle approaches (RCAs) to refine methods to identify eligible patients for the trial and develop the content of clinician messages that can be integrated into the electronic health record (EHR) as an implementation strategy to encourage genetic testing.

**Methods:** We tested electronic phenotyping methods using random manual chart review to ensure correct identification of patients eligible for the clinical trial. We used stakeholder meetings and a discrete choice experiment (DCE) with clinicians who provide genetic testing across our health system to develop and test four messages, each addressing a bias, informed by behavioral economics (status quo, focusing effect, impact, and omission), that may be associated with willingness to order, or refer for, genetic testing. Clinicians were interviewed and selected their preference between each pairing of messages and in both orders.

**Findings:** We are showing acceptable levels of positive predictive value using our electronic phenotyping methods. Following stakeholder meetings to design the messages, 43/79 (54%) clinicians completed our DCE. Preference was significantly greater for the message that addresses status quo bias (44%), vs. focusing effect (22%), impact (18%), or omission (17%) bias (chi-square[3]=46.17, p<0.001). Preference did not vary by clinician sex or academic rank (p’s >0.05).

**Implications for D&I Research:** These findings show we can identify patients for this trial and support the use of an EHR message targeting status quo bias in our trial as an implementation strategy to increase the use of genetic testing across our health system. This study also highlights the use of RCAs as formative research prior to conducting clinical trials.

**Primary Funding Source:** National Institutes of Health

### S112 From SIPREP to playbook: Adapting implementation navigation and monitoring into a clinical partner playbook

#### Sean Baird^1^, Alaina Preddie^1^, Jessica Kirchgassner^1^, Brandon Edwards^1^, Nicholas Rattray^1^, Teresa Damush^1^, Edward Miech^2^, Linda Williams^3^

##### ^1^Veterans Health Administration, VA HSR&D Center for Health Information and Communication (CHIC) and VHA HSR&D PRIS-M QUERI; Richard L. Roudebush VA Medical Center, Indianapolis, IN, USA; ^2^VHA HSR&D Center for Health Information and Communication (CHIC) and Quality Enhancement Research Initiative Expanding expertise Through E-health Network Development (EXTEND) QUERI Centers, Roudebush VA Medical Center, Indianapolis, IN, USA; ^3^ Richard L. Roudebush VA Medical Center, Indianapolis, IN, USA

###### **Correspondence:** Sean Baird (sean.baird@va.gov)

*Implementation Science 2024*, **19(2):**S112

**Background:** Creating an implementation playbook can provide operational partners detailed and practical guides for implementing Evidence Based Practices (EBP) (Huynh et al. 2018). The “State of Implementation” Progress Report (SIPREP) uses a grid system with cells organized by implementation activities, stages, personnel roles, and other implementation process categories to detail and monitor the implementation process and to generate operational partner playbooks (Miech et al. 2020). Our aim was to demonstrate how the SIPREP can be adapted to offer a tailored implementation playbook for clinical partners.

**Methods:** The VA Expanding Expertise Through E-health Network Development (EXTEND) QUERI Program aims to expand VA patient care to improve Veteran outcomes by delivering high-quality evidence-based telehealth services. EXTEND is implementing three projects to promote evidence-based practices that address high-priority Veteran conditions delivered via telehealth: TeleNeurology, TeleGRACE, and Cognitive-Behavioral Therapy for Headache. To outline and monitor implementation processes across stages, each project utilized a project specific SIPREP. Project SIPREPs provided the template for making clinical partner playbooks that detail key components, tasks, necessary personnel and respective roles, sequence of activities to implement a respective project. We conducted an inductive mixed-methods analyses of the VA EXTEND QUERI’s three SIPREPs which monitored implementation processes and the development of a clinical partner playbook.

**Findings:** During pre and early implementation phases for each of the respective EXTEND programs, SIPREPs were created and tailored for the needs of each project. For each of the projects, updates to the SIPREP tracked important changes and adaptations to implementation, as well as determined priority or relevance for tasks and roles for implementation of respective programs. The TeleNeurology (TN) SIPREP was updated throughout the implementation project and served as the basis for a TeleNeurology e-consult implementation playbook for clinical partners that features the most central implementation activities and personnel required for implementation.

**Implications for D&I Research:** The integrated, temporal approach of the TN EXTEND SIPREP laid the groundwork for adapting a SIPREP to be a clinical partner playbook. This approach demonstrates the usefulness of the SIPREP for not only implementing and monitoring implementation, but providing the blue print for future partners to continue the implementation of targeted EBPs.

**Primary Funding Source:** Department of Veterans Affairs

### S113 Primary care pharmacist modeling tool to alleviate provider workload burden

#### Marie Smith^1^, Mary Mulrooney^2^

##### ^1^69 North Eagleville Road, Unit 3092, The University of Connecticut School of Pharmacy, Storrs, CT, USA; ^2^University of Connecticut School of Pharmacy, Storrs, CT, USA

###### **Correspondence:** Marie Smith (marie.smith@uconn.edu)

*Implementation Science 2024*, **19(2):**S113

**Background:**

A challenge exists to identify the optimal use of pharmacists in primary care (PC) teams to offset PCP workload. Objectives: (1) describe the development of a PC modeling tool, PCImpact, and (2) measure the impact of integrated pharmacist services on PCP workload burden and patient/PCP appointment access.

**Methods:**

PCImpact has 2 types of pharmacist practice models: population health (PH) and direct patient care (DPC). In the PH model, a pharmacist performs one-time, comprehensive medication reviews using EHRs with no patient interaction. PCPs review/implement any pharmacist recommendations. In a DPC model, an embedded PC pharmacist has written collaborative practice agreements (CPAs) with PCPs. CPAs allow the pharmacist to schedule patient appointments, assess medication regimens, implement medication changes, and order follow-up lab tests without requiring PCP implementation -- thus, lowering PCP workload for medication management.

PCImpact was developed/tested with 6 provider organization leaders by calculating: (1) pharmacist workload capacity annually, (2) PCP time saved or required for implementation of the pharmacist recommendations and any additional PCP appointment opened, and (3) number of patients who benefit from pharmacist medication optimization.

**Findings:**

More patients can receive a medication review in the PH vs DPC model (2,304 vs. 640) because PH medication reviews require less pharmacist time than DPC initial and follow-up visits. The PH practice model adds 384 hours to the PCP workload per year whereas, the DPC model shows a 640-hour PCP workload reduction leading to opening up 1,920 PCP appointments per year for more acute patients. The DPC model has more patients who benefit from pharmacist medication optimization/management recommendations (3,040 vs. 922) with the higher implementation rate of pharmacist recommendations (95% vs. 40%) with CPAs.

**Implications for D&I Research:**

PCImpact is a novel method to forecast the impact of different pharmacist models/services on PCP clinical workload and patient access to PCP visits. PC organizational leaders can justify initiating/expanding pharmacist services by forecasting the annual capacity of the pharmacist to perform medication reviews/patient visits and the resulting impact on PCP workload and patient access to PCP appointments.

**Primary Funding Source:** Centers for Medicare and Medicaid Services

### S114 Use of concept mapping to derive a taxonomy of practice-level structures and processes to improve primary care outcomes

#### Margaret Paul^1^, Stephanie Albert^2^, Donna Shelley^3^, Saul Blecker^4^, Lorraine Kwok^4^, Carolyn Berry^4^

##### ^1^Mayo Clinic, Phoenix, AZ, USA; ^2^New York University Grossman School of Medicine, New York, NY, USA; ^3^NYU School of Global Public Health, New York, NY, USA; ^4^NYU Grossman School of Medicine, New York, NY, USA

###### **Correspondence:** Margaret Paul (paul.margaret@mayo.edu)

*Implementation Science 2024*, **19(2):**S114

**Background:** Primary care providers are under increasing pressure to achieve high performance standards but lack guidance on what specific structures and processes, such as care coordination and panel management, are associated with improvements in care quality and clinical outcomes.

**Methods:** We used a multifaceted approach including a scoping literature review, a Delphi study, and qualitative interviews with quality improvement specialists and lead clinicians at high-performing primary care practices to generate a list of 258 individual structures and processes associated with high performance. The list was collapsed to 121 items for inclusion in the concept mapping process. We recruited a multidisciplinary pool of experts which included health system leaders, primary care providers, and health services researchers. Participants used the GroupWisdom concept mapping platform to sort items into groups meaningful to them and the empirical data generated from sorting was used to establish consensus around a taxonomy.

**Findings:** A total of 31 experts completed the sorting exercise and the resulting taxonomy includes 8 conceptually distinct domains related to primary care practice: 1) address social factors and encourage patient engagement; 2) reduce clinical risk factors; 3) provide enhanced care; 4) expand access to care; 5) provide ancillary services; 6) establish care team processes and workflows; 7) use clinical information systems; and 8) use data and evidence. We moved a limited number (n=7) of items from one domain to another to improve fit, but only if the item was relatively close on the map and had high bridging values with items in the new domain. Our team, in consultation with our expert panel, developed a subdomain structure within 7 of the 8 domains to further organize taxonomy content. Domain 4 on expanding access to care did not require subdomain structure as it was relatively small, and all items were closely related.

**Implications for D&I Research:** We developed a novel and empirically derived taxonomy of evidence-based structures and processes for enhancing primary care at the practice level. The taxonomy provides organization to the large number of structures and processes that can improve primary care performance.

**Primary Funding Source:** National Institutes of Health

### S115 Unique CFI Attributes that Drive EDI Uptake in Criminal-Legal (CL) Settings

#### Rosemarie Martin

##### Brown Univ., Providence, RI, USA

###### **Correspondence:** Rosemarie Martin (rosemarie_martin@brown.edu)

*Implementation Science 2024*, **19(2):**S115

**Background:** CL systems are hierarchical, have a low propensity for risk, and are sometimes politically driven, but generally excel at sustaining innovations once implemented. The implementation process can be exceptionally challenging, but the potential for sustained success is high.

**Methods:** This project is implementing medication for opioid use disorders (MOUD) in seven adult probation offices across three states. Using the CFIR framework to organize the mixed methods organizational and staff data, we identify and describe key determinants in implementing Evidence-Based Innovations (EBIs) for overdose prevention in these systems and the implementation strategies to address them.

**Findings:** (a) Characteristics of the innovation: We will describe implementation strategies used to make the “relative advantage” case for the EBI to garner acceptance of CL staff within a culture that considers change a safety risk. (b) Inner setting attributes: We will describe how change teams were built to foster an adoption decision by leadership and to align the targeted EBI with existing workflows. (c) Outer Setting characteristics: We will outline the complex relationship between lawsuit risk and CL practice and how the implementation team leveraged this tension to motivate change. Specifically, we demonstrate the use of a stakeholder inventory process to identify and engage those who need to be involved in planning and conducting the EBI implementation. (d) Lastly, examples of audit and feedback methods using visualization of local jurisdiction data and statewide benchmarks will be presented along with strategies to guide and motivate the EBI implementation process.

**Implications for D&I Research:** CL systems are highly resistant to change. This study highlights multi-level strategies successfully used to engage staff and support implementation of EBIs, providing a model for other settings.

**Primary Funding Source:** National Institutes of Health

### S116 Using the implementation strategy integrity framework (ISIF) to interpret, replicate, and improve D&I efforts in criminal-legal (CL) settings

#### Todd Molfenter

##### University of Wisconsin, Madison, WI, USA

###### **Correspondence:** Todd Molfenter (todd.molfenter@chess.wisc.edu)

*Implementation Science 2024*, **19(2):**S116

**Background:** The unique context of CL settings means that even well-planned D&I strategies can often go off course, leading to unanticipated adaptations or results. Successful results often spark efforts to replicate the approach in other CL systems. Both circumstances require systematic documentation of D&I strategies and their intended and unintended variations and results.

**Methods:** Researchers from the Justice Community Opioid Innovation Network (JCOIN) developed the Implementation Strategy Integrity Framework (ISIF) as a structured approach to assessing the fidelity with which planned implementation strategies were executed, the contextual factors that contributed to planned and unplanned adaptations, and their impacts on study results.

**Findings:** The ISIF relies on and combines the conceptual underpinnings of implementation strategy fidelity and mechanisms of change literature. The ISIF addresses and documents (1) implementation strategy rigor (i.e., adherence, dose, and quality) as applied by the actors/ implementers; (2) the extent and quality of targeted users’ participation in implementation strategy activities; and (3) the degree to which those actions activate mechanisms of change that lead to EBI use. These areas are combined with select Inner and Outer Setting variables to explain the moderating and mediating effect of implementation strategy integrity on EBI outcomes. The presentation will include an example of how the ISIF was applied to a JCOIN study implementing opioid treatment services in jail settings across 13 states.

**Implications for D&I Research:** If D&I research results are to be replicated in other studies and settings, it is essential to document the fidelity with which the planned implementation strategies were actually applied. Because the ISIF was developed to capture the array of strategies deployed across various EBIs and CL systems, it has potentially broad applicability to other D&I research studies.

**Primary Funding Source:** National Institutes of Health

### S117 Strategies for enhancing collaborative practice in criminal-legal (CL) settings

#### Jennifer Becan^1^, Amanda Wiese^2^, Chelsea Wood^2^, Noah Painter Davis^3^, David Olson^4^, Danica Knight^1,5^, Kevin Knight^6^

##### ^1^Institute for Behavioral Research, Texas Christian University, Fort Worth, TX, USA; ^2^Fort Worth, TX, USA; ^3^Albuquerque, NM, USA; ^4^Chicago, IL, USA; ^5^fort worth, TX, USA; ^6^Texas Christian University, Ft Worth, TX, USA

###### **Correspondence:** Jennifer Becan (j.becan@tcu.edu)

*Implementation Science 2024*, **19(2):**S117

**Background:** Change teams” and similar collaborative implementation strategies are often used to support an organization’s adoption of a novel practice. When adoption requires changes that span the mission or boundaries of multiple systems – and when those missions conflict or are philosophically opposed to innovation – change teams must also incorporate activities to build trust, communication, and collaboration across systems.

**Methods:** This presentation draws from a study of parole offices across three states and the strategies required to facilitate the implementation of evidence-based substance use and opioid treatment services for their clients. A bundled implementation strategy called the Opioid-Treatment Linkage Model (O-TLM), as overlaid on the EPIS Framework, was developed to support collaboration and coordination across public and private sectors within 18 communities, including CL, contracted healthcare providers, and community-based addiction treatment settings. A structured approach was used to assess the extent of collaboration (Brewer et al., 2019; Palinkas et al., 2017) and its impacts on proximal and distal implementation outcomes. These measures are collected at four-time points along the 2.5 years of the bunded implementation strategy (during exploration, end of preparation, end of implementation, and end of sustainment phase) and have been completed by members of the 18 community change teams, representing approximately 130 leaders and line staff from partnering CL and health agencies.

**Findings:** The approach uses proximal indicators to monitor the degree of collaboration and how well implementation strategies enhance collaboration performance. Results indicate that agencies represented on the change teams participate in more collaboration opportunities at the end of the preparation phase compared to the exploration phase. Additionally, change teams are working toward desired implementation outcomes by prioritizing interagency collaboration in their proposed implementation plans, e.g., information sharing, allocation of resources, and network weaving.

**Implications for D&I Research:** CL and addiction treatment systems are under-resourced and present numerous structural barriers to implementation. This presentation will provide examples of a practical approach to assessing and enhancing collaboration between multiple community and organizational stakeholders.

**Primary Funding Source:** National Institutes of Health

## Prevention and Public Health

### S118 Adapting the ROSE postpartum depression prevention program for Chinese American mothers in Brooklyn

#### Yi-Ling Tan^1^, Bonnie Kerker^1^, Erica Willheim^1^, Grace Tian^1^, Angel Mui^2^, Simona Kwon^1^

##### ^1^NYU Grossman School of Medicine, New York, USA; ^2^NYU Grossman School of Medicine, New York, NY, USA

###### **Correspondence:** Yi-Ling Tan (yi-ling.tan@nyulangone.org)

*Implementation Science 2024*, **19(2):**S118

**Background:** Postpartum depression (PPD) is linked to negative outcomes for women and children, and disproportionately affects low-income women of color. Asian Americans have the lowest rate of mental health service uptake and face complex barriers to mental health care-seeking. Reach Out and Stay Strong Essentials for new mothers (ROSE) is an evidence-based intervention (EBI) that aims to increase the use of social support and coping skills to decrease PPD. ROSE was developed for and tested among Latinx and Black women. Using a rigorous, community-engaged approach, we culturally and linguistically adapted ROSE for low-income, immigrant Chinese American mothers who seek prenatal care in an immigrant community in Brooklyn.

**Methods:** We used an iterative 2-phase approach to adapt and pilot ROSE among Chinese-speaking mothers guided by a systematic process which integrates community-engagement, implementation science, and surface and deep cultural domains. Firstly, we culturally and linguistically adapted ROSE through an iterative process which included a literature review, key informant interviews, and intensive study team discussion guided by community stakeholder feedback. During the second phase, we piloted the adapted ROSE program and collected data through participant surveys, facilitator consultation calls, post-intervention participant debrief sessions, and focus groups.

**Findings:** We will present on the framework applied to guide the adaptation. Additionally, we will share the major adaptations of the ROSE curriculum, which include modifications to a) a Western-centric linear communication spectrum emphasizing “assertive” communication to a focus on direct and indirect communication styles; b) encouraged mothers to engage in “stress-reduction activities” rather than recharging activities; c) highlighted the impact of mind-body balance on family harmony; and d) introduced more external characters and scenarios. We will also share data collected from three waves of the pilot intervention.

**Implications for D&I Research:** In order for EBIs to be successful in diverse communities, cultural adaptation is key. We anticipate that this adaptation will increase the acceptability and usability of ROSE among Chinese American women, addressing a gap in the practice-base for a culturally tailored PPD prevention intervention among high-risk Chinese American mothers in an immigrant community.

**Primary Funding Source:** Robin Hood Foundation

### S119 Science-based habit formation app as an adjunct to the national diabetes prevention program lifestyle change program (LCP) for populations in locations with a high community vulnerability index

#### Michael Cannon^1^, Josh Leichter^2^, Zena Belay^3^, Yvonne Mensa-Wilmot^1^, Casey Hughes^2^, Mallory Rowell^2^, Anyssa Garza^2^, Keenan Walch^4^, Marieta Pehlivanova^5^, Abby Schmalz^2^, Kyra Bobinet^2^

##### ^1^Centers for Disease Control and Prevention, Atlanta, GA, USA; ^2^Fresh Tri, Inc, Felton, CA, USA; ^3^Independent Consultant, New York, NY, USA; ^4^Kins Health, Inc., San Diego, CA, USA; ^5^University of Virginia Health System, Charlottesville, VA, USA

###### **Correspondence:** Michael Cannon (mcannon@cdc.gov)

*Implementation Science 2024*, **19(2):**S119

**Background:** Individuals with prediabetes who participate in lifestyle change programs (LCP) can reduce their risk of developing type 2 diabetes, but enrollment and retention remain significant challenges. These challenges may be addressed through program adaptations to improve implementation. We evaluated the use of a habit formation app intended to increase program engagement and improve behavioral outcomes, focused on locations with a high community vulnerability index.

**Methods:** We recruited study sites nationally from a registry of CDC-recognized National Diabetes Prevention Program sites, targeting sites with a high community vulnerability index. We compared outcomes for participants in LCP cohorts randomized to the control arm (standard LCP curriculum) and intervention arm (standard LCP curriculum plus the Fresh Tri habit formation app). The Fresh Tri app employs the proprietary Iterative Mindset MethodTM, based on a dynamic, non-linear cycle of behavioral practice, assessment, and iteration. The analysis included 212 participants in the intervention arm (25 cohorts) and 152 in the control arm (26 cohorts), with a primary endpoint of six months. Participants in the intervention arm came from study sites in locations that scored significantly higher on a community vulnerability index than control (0.69 vs. 0.64, p = 0.046; national average = 0.5).

**Findings:** Number of sessions attended through six months was significantly higher in the intervention arm than the control arm (14.4 vs. 13.2, P=0.014). Similarly, the percentage of participants retained through six months was significantly higher in the intervention arm than the control arm (79.7% vs. 67.1%, P=0.0065). At our 12-month secondary endpoint, retention was also significantly higher in the intervention arm than the control arm (78.3% vs. 58.1%, P=0.0099). The percentage of participants losing > 5% body weight at six months was similar for the intervention and control arms (26% vs. 27%, P=0.83) and non-significantly different at 12 months (43.5% vs. 28.6%, P=0.13).

**Implications for D&I Research:** An implementation improvement strategy of supplementing the standard LCP curriculum with a habit formation app led to more sessions attended and greater time-in-program among individuals from locations with a high community vulnerability index. Next, we plan to analyze qualitative study data to identify factors that predicted higher engagement.

**Primary Funding Source:** Centers for Disease Control and Prevention

### S120 Applying the frame for systematic adaptation of a state health curriculum to address outreach and content gaps for school-based physical activity

#### Anna Schwartz^1^, Andria Eisman^2^, Lisa Jo Gagliardi^3^, Rebecca Hasson^1^

##### ^1^University of Michigan School of Kinesiology, Ann Arbor, MI, USA; ^2^Wayne State University College of Education, Detroit, MI, USA; ^3^LJ Gagliardi, LLC and Michigan School Health Coordinators' Association, Hessel, MI, USA

###### **Correspondence:** Anna Schwartz (anschwa@umich.edu)

*Implementation Science 2024*, **19(2):**S120

**Background:** The purpose of this study was to develop a planned, fidelity-consistent adaptation for an evidence-based state health curriculum (Michigan Model for Health, MMH) to address outreach and content gaps and ultimately improve the reach, engagement, and effectiveness of a comprehensive Tier 1 (universal prevention) intervention.

**Methods:** The Framework for Reporting Adaptations and Modifications-Enhanced (FRAME) guided the development of the adaptation, which included eight aspects: (1) timing; (2) planning; (3) stakeholder involvement; (4) scope of adaptation; (5) level of delivery; (6) type of contextual or content adaptations; (7) relationship with fidelity; and (8) reasons for adaptation. Regional school health coordinators, who provide training and technical assistance for MMH, were introduced to the adapted curriculum, with their initial perceptions (acceptability, appropriateness, feasibility) of the curriculum assessed via survey.

**Findings:** The timing of the adaptation occurred during pre-implementation (1) and was planned (2). The Michigan Department of Health and Human Services, individual practitioners, and researchers made the decision to develop an adaptation (3) where MMH curriculum content and training would be modified (4) at the network/system-level (5). The content modification consisted of integrating elements of an evidence-informed physical activity program (Interrupting Prolonged sitting with ACTivity) into the MMH curriculum and creating changes in curriculum packaging (6). This was a fidelity-consistent adaptation, as core elements of MMH were left unchanged (7). The goal of the adaptation was to address content (physical activity) and outreach (family engagement) gaps of the MMH curriculum identified by program developers to ultimately improve curriculum reach, engagement, and effectiveness (8). Initial perceptions from 17 out of 21 regional school health coordinators indicated that on average, 34% “agreed” and 66% “completely agreed” the adapted curriculum was acceptable; 38% “agreed” and 62% “completely agreed” it was appropriate; and 1% “neither agreed nor disagreed,” 53% “agreed,” and 46% “completely agreed” it was feasible.

**Implications for D&I Research:** The systematically adapted MMH curriculum demonstrated high acceptability, appropriateness, and feasibility among key facilitators. This process allowed for adaptable components of MMH to be updated and offer more options to support structured tailoring of MMH to ultimately enhance reach, engagement, and curriculum effectiveness.

**Primary Funding Source:** Michigan Health Endowment Fund

### S121 Adaptation of an evidence-based occupational sun safety program for underserved outdoor workers in southwest Georgia

#### Alexandra Morshed^1^, Mary Buller^2^, Barbara Walkosz^2^, Kayla Anderson^1^, Cam Escoffery^1^, Radhika Agarwal^1^, Irene Adjei^2^, Brandon Herbeck^2^, David Buller^2^

##### ^1^Rollins School of Public Health, Emory University, Atlanta, GA, USA; ^2^Klein Buendel, Inc., Golden, CO, USA

###### **Correspondence:** Alexandra Morshed (alexandra.morshed@emory.edu)

*Implementation Science 2024*, **19(2):**S121

**Background**: African American and Hispanic workers face unique skin cancer risks from UV exposure and are underrepresented in occupational sun safety research. We report on adaptation of an existing evidence-based sun safety program—Go Sun Smart at Work—to predominantly African American and Hispanic outdoor workers in local government employers in Southwest Georgia.

**Methods**: Our systematic adaptation process was informed by Escoffery’s Key Adaptation Steps,^1^ Implementation Research Logic Model (IRLM),^2^ and the Framework for Reporting Adaptations and Modifications.^3^ The original intervention promotes workplace policy and education to improve sun protection and was effective in a randomized trial in Colorado.^4-5^ We mapped the program and delivery using IRLM and developed an adaptation tracking tool based on Rabin et al. ^6^ to catalogue and make decisions about potential adaptations. We are integrating multiple sources of information to identify compatible and feasible adaptations: consultation with program experts and community partners, qualitative data collection with outdoor workers and managers at two local government employers (Jul-Sep 2023), and synthesis of literature.

**Findings**: The intervention includes training of peer coaches; written audit of employer sun safety policies, practices; employee training; and resource website (e.g., training, sample policies, print materials, videos). In a pilot study with two local government employers, 11 employees identified by senior managers completed virtual 30-minute instruction to be peer coaches. Peer coaches delivered the sun safety training at staff meetings (21-39 employees per session). Several promising adaptations have been identified, including modification of educational materials to better represent worker population, addressing misconceptions, and use of external peer coaches at the workplace. Interviews with managers and focus groups with employees are scheduled for late summer. The adaptation process will be summarized and next steps on the project discussed.

**Implications for D&I Research:** Adaptation of existing, effective interventions to better address population characteristics and needs can increase feasibility and scalability with employers throughout Georgia, which furthers dissemination and reach of existing evidence base for improving occupational sun safety and preventing cancer.

**Primary Funding Source:** Centers for Disease Control and Prevention

**References**

1. Escoffery et al. 2019. 10.1093/tbm/ibx067
2. Smith et al. 2020. 10.1186/s13012-020-01041-8
3. Wiltsey Stirman et al. 2019. 10.1186/s13012-019-0898-y
4. Buller et al. 2017. 10.1177/0890117117704531
5. Walkosz et al. 2018. 10.1097/JOM.0000000000001427
6. Rabin et al. 2018. https://doi.org/10.3389%2Ffpubh.2018.00102

### S122 Identification of strategies to enhance the provision of holistic services across three community-based organizations in King County, Washington

#### Sarah Shaw^1^, Anjuli Wagner^1^, Alejandra Grillo-Roa^2^, Matt Jarrell^3^, Steven Sawyer^4^, Martha Zuniga^2^, Kai Horton^3^, Taylor Rapson^1^, David Katz^5^

##### ^1^University of Washington, Seattle, USA; ^2^Entre Hermanos, Seattle, USA; ^3^Seattle's LGBTQ+ Center, Seattle, USA; ^4^POCAAN, Renton, USA; ^5^Department of Global Health, University of Washington, Seattle, WA, USA

###### **Correspondence:** Sarah Shaw (sarahs29@uw.edu)

*Implementation Science 2024*, **19(2):**S122

**Background:** Addressing social determinants of health within HIV services may improve HIV outcomes and health equity. Community-based organizations (CBOs) are indispensable in providing holistic support to historically underserved communities, yet strategies supporting integration remain a key knowledge gap. In a community-academic partnership, we applied a systematic consensus-building approach to identify, prioritize, and characterize strategies supporting three CBOs’ goals to enhance holistic services and address health inequities.

**Methods:** We applied nominal group technique (NGT) methods with each CBO to generate the list of strategies which aligned with conditions CBOs deemed necessary for successful integration (community engagement, positive work environment, data, inclusive and accessible environment, and staff learning). Staff evaluated each strategy’s perceived feasibility and effectiveness using 5-item Likert Scales (Feasibility: 1=Very hard to do with current resources to 5=Very easy; Effectiveness: 1=Large negative impact to 5=Large positive impact). To inform implementation prioritization, we generated Go-Zone plots, i.e., scatterplots comparing strategies’ feasibility and effectiveness scores divided into quadrants using the mean for each dimension. Afterwards, we mapped strategies to thematic clusters from Expert Recommendations for Implementing Change (ERIC) and constructs from the Consolidated Framework for Implementation Research (CFIR).

**Findings:** The process generated 118 strategies with mean perceived feasibility and effectiveness scores of 3.35 (range=3.23-4.57) and 4.67 (range=4.64-5.0), respectively. Higher feasibility-higher effectiveness strategies (n=33) most commonly aligned with the following ERIC clusters and CBO-defined conditions: engaging consumers (n=9) (community engagement, inclusive/accessible environment), training and educating stakeholders (n=9) (positive work environment, community engagement, staff learning, data), developing stakeholder interrelationships (n=6) (community engagement, work environment, inclusive/accessible environment), and supporting clinicians/staff (n=5) (work environment, inclusive/accessible environment). These strategies most frequently mapped to the structural characteristics, organizational culture, staff capability, and assessment of recipient needs constructs from CFIR.

**Implications for D&I Research:** This community-driven implementation science approach generated strategies perceived as highly feasible and effective for enhancing holistic services and advancing health equity related to engaging communities, building positive work environments and organizational cultures (through training, developing stakeholder interrelationships, and supporting staff), and optimizing organizational structures to use data effectively and create accessible services. Strategy mapping highlights the need to test strategies that support systems-strengthening for CBOs and community engagement.

**Primary Funding Source:** National Institutes of Health

### S123 An ecological comparison of a push-pull community-based dissemination intervention with online and conventional venue dissemination of oral HIV self-testing

#### M Margaret Dolcini^1^, Joseph Catania^1^, Ashley Schuyler^1^, Tim Menza^2^, Jonathan Garcia^1^, Edgar Mendez^3^, Tara Casey^3^, Tony Diep^3^, Christina Sun^4^, E Roberto Orellana^5^, Mia Tognoli^1^, Nell Carpenter^3^, Lance Pollack^6^, Jesse Canchola^7^, Jeffrey Klausner^8^, Christopher Hamel^9^

##### ^1^Oregon State University, Corvallis, OR, USA; ^2^Oregon Health Authority, Portland, OR, USA; ^3^Cascade AIDS Project, Portland, OR, USA; ^4^University of Colorado, Denver, Denver, CO, USA; ^5^University of Washington, Seattle, WA, USA; ^6^University of California San Francisco, San Francisco, CA, USA; ^7^StatCon Consulting, Hayward, CA, USA; ^8^University of Southern California, Los Angeles, CA, USA; ^9^Multnomah County Health Department, Portland, OR, USA

###### **Correspondence:** M Margaret Dolcini (Peggy.Dolcini@oregonstate.edu)

*Implementation Science 2024*, **19(2):**S123

**Background:** Oral HIV self-testing (OHST) has been slow to gain acceptance within public sectors. Using a Push-Pull Model, we developed a free community-based network intervention (CBNI) to increase dissemination to men-who-have-sex-with-men in Multnomah County (Portland), Oregon. We compared the CBNI to other HIV testing programs in Multnomah County to address two questions: a) Will home-testing programs reduce the need for clinic-based testing?, and b) Is free online at-home-testing more efficient than CBNI in disseminating OHSTs?

**Methods:** We developed a dissemination network of commercial businesses (n = 6; e.g., bars, bathhouses) serving the LGBTQ+ community that provided continuous dissemination of free OHSTs over 3 months. We compared the CBNI to the Oregon Health Authority’s free OHST online dissemination program, and the Multnomah County Department of Public Health’s clinic-based HIV testing program for the 3-month period prior to the CBNI intervention and for the CBNI intervention window.

**Findings:** The CBNI disseminated 1,684 OHSTs. The online program disseminated 55 test kits in Multnomah County during the intervention period (M = 13.7) and 99 kits pre-intervention (M = 33.0; *t* = 15.9, p < .0001). The decline in online testing was reflected in a general downward trend statewide (State Ms = 98.0 pre-intervention, 46.7 intervention-period; *t* = 26.3, p < .0001), but the percent decline was significantly greater at the county vs. state level (X^2^ = 5.6, p = .01). Clinic testing increased from pre-intervention (n = 1,242 tests, M = 414.0) to the intervention-period (n = 1,685 tests, M = 421.2; *t* = -9.1, p < .0001).

**Implications for D&I Research:** The CBNI disseminated substantially more OHSTs than the online program. Despite a downturn in online dissemination both statewide and at the county level, the county decline was significantly larger, suggesting that the CBNI had a negative effect on online dissemination. Contrary to predictions, clinic-based testing significantly increased during the CBNI. This may reflect efforts by the CBNI to encourage clinic-based confirmatory testing for persons testing HIV-positive. Overall, implementation of novel dissemination strategies may be combined with conventional strategies to maximize community dissemination, and potentially reach.

**Primary Funding Source:** National Institutes of Health

### S124 Better together: A multi-level implementation approach to improve health promotion in early care and education

#### Falon Smith^1^, David Yates^1^, Caliste Chong^2^, Aviva Starr^1^, Erik Willis^1^, Derek Hales^1^, Emily Alexander^1^, Dianne Ward^1^

##### ^1^University of North Carolina at Chapel Hill, Chapel Hill, NC, USA; ^2^Nemours Children's Health, Wilmington, DE, USA

###### **Correspondence:** Falon Smith (ftilley@unc.edu)

*Implementation Science 2024*, **19(2):**S124

**Background:** Systemic barriers contribute to challenges faced in implementing sustainable improvements in early care and education (ECE) health promotion practices, including fragmented state ECE systems, limited organizational capacity, and a lack of accessible, high-quality professional development opportunities. Guided by the Reach, Effectiveness, Adoption, Implementation, and Maintenance (RE-AIM) framework, the objective of this study is to evaluate the Better Together approach—a multi-level quasi-experimental implementation initiative aimed at enhancing health promotion practices in ECE programs.

**Methods:** Better Together, developed through collaboration between Nemours Children's Health and Go NAPSACC (UNC-Chapel Hill), used organizational- and systems-level approaches to improve ECE health promotion practices. Learning collaboratives (organizational level) addressing ECE health practices (content areas: breast/infant feeding, child nutrition, physical activity, and screen time) were delivered by trained consultants. State partners coordinated efforts with key stakeholders to create state-level systems change using the Centers for Disease Control and Prevention’s Spectrum of Opportunities Framework. Surveys and program data were collected to assess reach, effectiveness, adoption, and implementation. Descriptive statistics were used to evaluate RE-AIM outcomes.

**Findings:** From 2020 to 2022, four states implemented Better Together. Overall uptake included 1,046 ECE staff from 569 programs (reach). On average ECEs experienced a 15.1% increase in health promoting practices (range 11.8-17.5%; effectiveness). Consultants implemented all planned sessions (n=26; implementation). Across states, ECE program completion of the Better Together initiative components ranged from 58.0-94.7% (adoption). At the state level, stakeholder groups generated system-level 10 action plans to initiate changes across six of the nine Spectrum of Opportunities that remains ongoing.

**Implications for D&I Research:** The RE-AIM evaluation of the Better Together program showed positive results. It generated meaningful improvements in state systems and reached a significant number of ECE staff, leading to an average 15.1% increase in health-promoting practices. However, completion rates varied, indicating potential barriers to adoption. Identifying and addressing these barriers is crucial for program improvement. The structure and funding for the current project did not allow for a maintenance evaluation, which will be needed to assess long-term sustainability and impacts of the Better Together approach. By considering these implications, future D&I research can enhance program implementation and promote lasting changes in early childhood settings.

**Primary Funding Source:** Private Foundation

### S125 D&I training in evidence-based prevention: A developmental focus

#### Cady Berkel^1,2^, Nancy Gonzales^3^, Jd Smith^4^, Krista Leonard^2^, Matt Buman^2^, Paul Estabrooks^5^, Dana Epstein^6^, Felipe Castro^2^, Sabrina Oesterle^2^, Sarah Lindstrom Johnson^1^, Abi Gewirtz^3^, Jessica Austin^7^, Prajakta Adsul^8^, Niko Verdecias^2^, Chris Kemp^9^

##### ^1^Arizona State University, Tempe, AZ, USA; ^2^Arizona State University, Phoenix, AZ, USA; ^3^Arizona State University, Tempe, AZ, USA; ^4^Room 1N410, Spencer Fox Eccles School of Medicine at the University of Utah, Salt Lake City, UT, USA; ^5^University of Utah, Salt Lake City, UT, USA; ^6^Arizona State University, Phoenix, AZ, USA; ^7^Mayo Clinic Cancer Center Arizona, Scottsdale, AZ, USA; ^8^University of New Mexico, Albuquerque, NM, USA; ^9^Johns Hopkins, Seattle, WA, USA

###### **Correspondence:** Cady Berkel (cady.berkel@asu.edu)

*Implementation Science 2024*, **19(2):**S125

**Background:** Despite the fact that most morbidity and mortality is driven by preventable health behaviors and the decades of evidence to suggest that investment in prevention can reduce cost and suffering at the individual and societal levels, the United States healthcare system is oriented towards treatment, and has had limited focus on prevention. This focus on treatment has resulted in a dearth of formal training opportunities focused on the implementation of evidence-based preventive interventions. Furthermore, to achieve health equity, there is a need for greater focus and specific training on methods and practices for effective community engagement in D&I. Without engagement of partners from under-resourced communities, there is no chance of rectifying the health and implementation disparities which co-occur in the communities most affected by inequitable social structures.

**Methods:** We describe efforts to enhance skills in community-engaged dissemination and implementation (CEDI) science among prevention scientists from the graduate to early/mid career stages via two training programs. Graduate and postdoctoral training is supported by a T32 training program (MPIs: Berkel & Gonzales) funded by NIMH for over two decades, and by NIDA for the past 7 years. During this time, the program shifted from basic and efficacy trials to effectiveness and implementation studies. In 2019, ASU launched a D&I training program geared towards early career faculty, which has evolved this year to encompass the Mountain States Partnership for Community Engaged Dissemination & Implementation (MS-CEDI Directors: Berkel, Smith, Buman & Estabrooks).

**Findings:** We will describe the similarities and differences of the two training programs, highlighting the developmental needs of graduate students, postdocs, and early career faculty. In particular, we will focus on adaptations in both programs in the past year to provide opportunities for community-engaged research. We will also provide quantitative data on trainee improvement in CEDI skills and assessing each component of the respective program. Posttest data collection is nearly complete.

**Implications for D&I Research:** Results will inform future training efforts to enhance the CEDI skills of implementation scientists. We will also comment on the appropriateness and sufficiency of metrics of success for D&I training programs.

**Primary Funding Source:** National Institutes of Health

### S126 Identifying jurisdiction-level determinants and implementation strategies for rapid ART, same-day PrEP, and HIV status-neutral implementation: Key informant interview results from the rapid START multisite preparatory study

#### Alithia Zamantakis^1^, Sheree Schwartz^2^, Anna-Sophia Katomski^3^, Ana Michaela Pachicano^1^, Nanette Benbow^4^, Cathleen Willging^5^, Alison Hamilton^6^, Ashley Hagaman^7^, Joseph G. Rosen^8^, Robin G. Lanzi^9^, Laura Beres^2^

##### ^1^Northwestern University, Chicago, IL, USA; ^2^Johns Hopkins Bloomberg School of Public Health, Baltimore, MD, USA; ^3^Johns Hopkins University, Baltimore, MD, USA; ^4^Northwestern University Feinberg School of Medicine, Chicago, IL, USA; ^5^Pacific Institute for Research and Evaluation, Albuquerque, NM, USA; ^6^Veterans Health Administration, VA Greater Los Angeles Healthcare System, Los Angeles, CA, USA; ^7^Yale School of Public Health, New Haven, CT, USA; ^8^Johns Hopkins University, Baltimore, USA; ^9^University of Alabama at Birmingham, Birmingham, AL, USA

###### **Correspondence:** Alithia Zamantakis (alithia.zamantakis@northwestern.edu)

*Implementation Science 2024*, **19(2):**S126

**Background:** Ending the HIV Epidemic (EHE) in the U.S. requires optimized HIV **s**ervice delivery. Rapid initiation of antiretroviral therapy following HIV diagnosis (rapid ART) and same-day pre-exposure prophylaxis (PrEP) are evidence-based interventions to treat and prevent HIV. A status-neutral approach in which those who test positive for HIV receive ART and those who test negative receive PrEP services may mitigate stigma and barriers to care.

**Methods:** We sought to understand definitions, determinants, and implementation strategies for rapid ART, PrEP, and status-neutral implementation within HRSA-funded Ryan White HIV/AIDS Program clinics in five EHE-priority jurisdictions: Alabama, Baltimore, Chicago, Dallas, Los Angeles, and San Diego. We conducted key informant interviews (KIIs) with 2-3 health department (HD) experts across jurisdictions (n=11). KIIs were guided by the Consolidated Framework for Implementation Research. We used rapid thematic analysis consisting of completion of debriefs, memos, and matrices to identify salient constructs and emergent themes, with findings compared across jurisdictions.

**Findings:** KIIs highlighted the importance of Outer Setting context. Policy and insurance were facilitators in states like California but functioned as barriers in states like Texas. In states lacking prevention funding, the ability for HDs to cover initial costs of rapid ART prior to reimbursement enabled same-day PrEP implementation. HDs’ contracts with clinics emerged as a notable bridging factor, but gaps in what is covered, and a lack of performance-metrics reporting reduced its potential to scale up implementation. Quality improvement initiatives and learning collaboratives were utilized in most jurisdictions to develop definitions of rapid start, collect data, and initiate scale up. Prioritizing a status-neutral approach can facilitate same-day PrEP scale up, whereas implementing rapid ART and same-day PrEP as separate interventions reinforces siloes, due to complications in the separation of prevention and testing funds.

**Implications for D&I Research:** Research must attend to heterogeneities in multilevel determinants of rapid ART, same-day PrEP, and status-neutral implementation. Understanding Outer Setting contexts across jurisdictions is important for developing implementation strategies appropriate to different settings. Status-neutral implementation is conceptually ahead of the implementation infrastructure necessary to enable implementation. Standardized procedures may support wider adoption of status-neutral approaches.

**Primary Funding Source:** National Institutes of Health

### S127 A push-pull intervention to improve community-based dissemination of oral HIV self-testing

#### Joseph Catania^1^, M Margaret Dolcini^1^, Ashley Schuyler^1^, Jonathan Garcia^1^, Edgar Mendez^2^, Tara Casey^2^, Tony Diep^2^, Christina Sun^3^, E Roberto Orellana^4^, Mia Tognoli^1^, Nell Carpenter^2^, Lance Pollack^5^, Jesse Canchola^6^, Jeffrey Klausner^7^, Christopher Hamel^8^

##### ^1^Oregon State University, Corvallis, OR, USA; ^2^Cascade AIDS Project, Portland, OR, USA; ^3^University of Colorado, Denver, Denver, CO, USA; ^4^University of Washington, Seattle, WA, USA; ^5^University of California San Francisco, San Francisco, CA, USA; ^6^StatCon Consulting, Hayward, CA, USA; ^7^University of Southern California, Los Angeles, CA, USA; ^8^Multnomah County Health Department, Portland, OR, USA

###### **Correspondence:** Joseph Catania (catania1951@comcast.net)

*Implementation Science 2024*, **19(2):**S127

**Background:** Oral HIV self-testing (OHST) was first approved in 2002, and has been slow to gain acceptance within public health sectors and among the general population. Using a Push-Pull Model (PPM), we developed a community-based intervention to increase the rate of OHST dissemination to men-who-have-sex-with-men (MSM) in Portland, Oregon.

**Methods:** We developed a dissemination network of commercial businesses (n = 6) serving the LGBTQ+ community, a network communication and OHST supply system, and a community-driven multi-channel program to promote OHST dissemination. Businesses were continuously resupplied, and data were collected weekly on the number of kits distributed and business personnel’s observations on program acceptability. Street intercepts were conducted with customers at multiple points during the intervention to assess awareness of the dissemination program. Post-intervention interviews were conducted with customers and business personnel to obtain reactions to the dissemination process.

**Findings:** Over a 12-week intervention period, the dissemination effort distributed 1,684 OHSTs. We examined changes in dissemination rates corresponding to intensive promotional efforts (first 6 weeks) of the intervention, and a low-intensity promotional effort (final 6 weeks). There was a significant drop in dissemination rates (% total dissemination) between the two 6-week periods (56% vs. 44%; Z = 7.08, p < .001). We examined changes in customer awareness of the program over time and found awareness had increased from 22% within the first two weeks of the promotional campaign to 60% during weeks 7-9 (Z = 6.5, p < .001). Post-intervention interviews revealed a positive response by customers and business personnel to the dissemination of OHSTs, and a desire to continue the program.

**Implications for D&I Research:** A PPM intervention facilitated dissemination of OHSTs at the community level. Community-driven multi-channel promotional efforts were successful in diffusing awareness of the dissemination effort among MSM customers. Despite increasing program awareness over time, OHST dissemination fell, suggesting that customers may have reached a saturation point in kit demand. Alternatively, decreased demand is also associated with a reduction in the intensity of the promotional program. The results underscore the value of the PPM for dissemination intervention development.

**Primary Funding Source:** National Institutes of Health

### S128 Identifying factors associated with innovation implementation and de-implementation in emergency departments during the COVID-19 pandemic

#### Shreya Huilgol^1^, Nabeel Qureshi^2^, Catherine Cohen^2^, Carl Berdahl^3^, Shira Fischer^1^

##### ^1^RAND Corporation, Boston, MA, USA; ^2^RAND Corporation, Santa Monica, CA, USA; ^3^Cedars-Sinai Medical Center, Los Angeles, CA, USA

###### **Correspondence:** Shreya Huilgol (shuilgol@rand.org)

*Implementation Science 2024*, **19(2):**S128

**Background:** During a public health emergency like the COVID-19 pandemic, ED clinicians may be able to save lives if they rapidly identify and implement innovations that are safe and effective. However, evidence is limited regarding factors that impact clinician decision-making regarding implementation and de-implementation of care innovations when evidence-based information is limited. The goal of this study was to understand how ED clinicians decided to implement and de-implement COVID-19 care innovations (e.g., proning), describe the barriers and facilitators they faced, and identify factors that led them to continue (or discontinue) using these innovations.

**Methods:** We conducted 12 virtual semi-structured focus groups representing 6 hospitals across the US, which were purposefully selected for diversity of urbanicity, geography and academic settings, and degree that communities were impacted by COVID-19. Nineteen physicians and advanced practice providers, 18 nurses, and 5 respiratory therapists consented to participate and be recorded for verbatim analysis. We utilized both inductive and deductive techniques to do content and thematic analysis.

**Findings:** Clinicians cited limited knowledge and institutional guidance and time constraints on staff training as barriers to implementing new innovations, while positive patient outcomes and perceptions that the innovations were protective to staff were considered implementation facilitators. The facilitators for innovation de-implementation were poor or no change in patient outcomes, cumbersome processes, negative experiences from sites that experienced surges earlier in time, and lack of equipment/resources. Barriers to de-implementation included a fear of discontinuing treatment that could be effective and the changing nature of the pandemic (e.g., more virulent strains, larger surges, vaccine availability).

**Implications for D&I Research:** Frontline ED clinicians are critical to the response to disease outbreaks like the COVID-19 pandemic. During those uncertain times, clinicians must rapidly implement new protocols and practices and assess these changes in real-time under uncertain and potentially dangerous conditions. Future work is needed to create mechanisms for rapidly identify promising innovations during evolving public health emergencies, and funders should support studies to monitor innovation effectiveness and safety after implementation so that evidence-based decisions can be made regarding how long to continue the implementation and when to de-implement.

**Primary Funding Source:** National Institutes of Health

### S129 Acceptability and feasibility of a train-the-trainer model for implementation of a parenting intervention

#### Amy M LeClair^1^, Briana Jurkowski^2^, Sophia Colon^3^, Amanda Lowell^4^, Amanda Zayde^5,6^, Rebecca Blanchard^7^, Amy Sommer^8^, Jessica Borelli^9^, Elizabeth Peacock-Chambers^10^

##### ^1^Tufts Medical Center, Boston, MA, USA; ^2^ UMass Chan Baystate Springfield, MA, USA; ^3^Bay Path University, Longmeadow, MA, USA; ^4^Yale Child Study Center, New Haven, CT, USA; ^5^Montefiore Medical Center, Bronx, NY, USA; ^6^Albert Einstein College of Medicine, Bronx, NY, USA; ^7^OnlineMedEd, Springfield, USA; ^8^Jewish Family and Children's Services, Waltham, MA, USA; ^9^University of California, Irvine, Irvine, CA, USA; ^10^UMass Chan Baystate, Springfield, MA, USA

###### **Correspondence:** Elizabeth Peacock-Chambers (elizabeth.peacock-chambers@baystatehealth.org)

*Implementation Science 2024*, **19(2):**S129

**Background**: Mothering from the Inside Out (MIO) is one of the few evidence-based parenting interventions specifically designed to meet the needs of parents in recovery from substance use disorders (SUDs). Training in MIO was previously conducted by two expert trainers making scalability a major limitation to wide-spread implementation of this program. This study assessed the acceptability and feasibility of a train-the-trainer model for scaling dissemination.

**Methods**: Train-the-Trainer involved virtual classroom preparation for new trainers and co-delivery of the MIO training with expert trainers. MIO training for new clinicians (mental health and SUD counselors) includes virtual didactic learning followed by delivery of 12 sessions to a parent with SUDs with weekly clinical supervision. Seven trainers completed the classroom preparation and 4 participated in training new clinicians. Of the 16 new clinicians, 11 completed the full MIO training with 10 clinicians and 8 parents participating in research assessments. Data were collected via multiple methods across different phases of the study (development, planning, implementation). Field notes, surveys, and semi-structured interviews were used to collect data from host organizations, expert trainers, new trainers, clinicians, and parents. We used rapid analysis and content analysis to assess acceptability and to map findings onto the Consolidated Framework for Implementation Research (CFIR). We then categorized CFIR domains according to their relevance to three phases of the train-the-trainer model.

**Findings**: Trainers reported the creation of a safe space to slow down, reflect, and grow into their trainer roles as the major strength. Some requested more time for preparation and reflection with the expert trainers, while recognizing scheduling limitations. New clinicians similarly identified slowing down to reflect as the primary strength of the training process and recommended more interactive activities during the didactic training. Multiple participants suggested explicitly discussing systemic racism and cultural differences within future trainings. Mothers articulated becoming more comfortable in their parenting roles after participation. Different CFIR implementation constructs aligned across the three different phases of the train-the-trainer model.

**Implications for D&I Research:** Train-the-trainer was a generally acceptable implementation strategy. This study highlights some of the strengths and limitations of this strategy for behavioral health interventions with parents in recovery.

**Primary Funding Source:** Tufts CTSI Pilot Award; NIDA K23

### S130 A multilevel implementation strategy to support scale-up of an innovative practice for parental opioid and methamphetamine use disorders

#### Lisa Saldana, Ryan Singh, Gracelyn Cruden, Mark Campbell

##### Chestnut Health Systems, Eugene, OR, USA

###### **Correspondence:** Lisa Saldana (lsaldana@chestnut.org)

*Implementation Science 2024*, **19(2):**S130

**Background**: The Families Actively Improving Relationships (FAIR) program is an evidence-based intervention to treat and prevent parental opioid and methamphetamine use, targeting families involved in the child welfare system (CWS). FAIR addresses SDOH needs and was developed, tested, and sustained for 8-years in a single county, adjacent to the local CWS, with Medicaid reimbursement. The NIH HEAL initiative provided the opportunity to expand FAIR into three rural counties in Oregon. Due to demand and demonstrated implementation success in the original counties, two additional counties adopted FAIR and another two are exploring. This unintentional scale-up is supported by a rigorous multilevel implementation process, to be described.

**Methods:** A well-defined multilevel strategy was operationalized using the Stages of Implementation Completion (SIC). The SIC is an 8-staged measure of full implementation process (pre-implementation, implementation, sustainment) fidelity. SIC scores include the Proportion of activities completed and the Duration taken to complete them. Previous SIC outcomes show that strong pre-implementation fidelity predicts program start-up and sustainment; thresholds have been modeled for high, acceptable, and poor SIC fidelity scores.

Forty-four distinct implementation activities were defined prior to launching a FAIR program. Implementation strategies included engagement with state, county, community, agency, and provider level actors: some activities were unique and others spanned across levels. Eleven activities were repeated across levels in SIC Stages 1 (Engagement) and 2 (Consideration of Feasibility), whereas Stage 3 (Readiness Planning) activities focused exclusively on agency preparation and facilitation of agency engagement across system levels. This multilevel implementation process provided the roadmap for implementation in each county.

**Findings:** Across all five counties, high pre-implementation fidelity was demonstrated using the SIC, with 100% activity completion. Engagement activities (will be describe) showed significant value. Four out of five counties demonstrated long, but acceptable pre-implementation Duration (219-366 days). One county showed slow Duration resulting from an unsuccessful implementation attempt prior to the ultimately successful agency.

**Implications for D&I Research:** There is an urgent need for effective treatment and prevention interventions to hinder the opioid and methamphetamine epidemics, particularly for CWS-involved populations. The multilevel SIC roadmap facilitates development of the necessary infrastructure to support and expand system adjacent interventions toward a public health impact.

**Primary Funding Source:** National Institutes of Health

### S131 Characterizing implementation of state and hospital policies on plans of safe care and the reporting of “substance exposed newborns” to child welfare agencies

#### Sachini Bandara, Valerie Ganetsky, Kenneth Feder, Brendan Saloner

##### Johns Hopkins Bloomberg School of Public Health, Baltimore, MD, USA

###### **Correspondence:** Sachini Bandara (sbandar2@jhu.edu)

*Implementation Science 2024*, **19(2):**S131

**Background:** Under federal law, “substance-exposed infants” must have a plan of safe care (POSC) with a child welfare agency that is often developed during the birth hospitalization. States have new federal POSC reporting requirements and significant leeway in how POSCs are implemented, but little guidance and research on best practices.

**Methods:** We conducted 20 semi-structured interviews of national experts and leadership from child welfare agencies and large hospitals in 6 states with different approaches to POSC implementation. Interview domains included: 1) criteria for identifying infants, 2) POSC implementation strategies, and 3) implementation facilitators and barriers. A hybrid inductive/deductive coding approach was used guided by the Exploration, Preparation, Implementation and Sustainment framework. Final interviews will be completed by September with ample time for further analysis of these data by December.

**Findings:** Even in states that provide criteria for identifying “substance-exposed infants” for POSC, there is considerable variation at the hospital level on the implementation of this criteria, particularly around drug testing practices. States also vary in the development of alternative notification systems for POSC and whether the initiation of a plan constitutes the initiation of a child welfare case. There is also variation in implementation of POSC for infants testing positive for cannabis exposure and for guardians engaged in substance use disorder treatment as well as the responsible entity for drafting the POSC and the required follow up from child welfare agencies.

**Implications for D&I Research:** This study applies D&I frameworks to the analysis of state policy implementation, a growing area of D&I research. POSC are a key mechanism through which infants enter the child welfare system. Because the implementation of POSC is not well understood, characterizing state policies and current practices of reporting can help identify opportunities to clarify guidance for providers and uncover potential impacts on patients.

**Primary Funding Source:** Bloomberg Philanthropies (PI: Joshua Sharfstein, no grant number)

### S132 Using CFIR to determine initial and ongoing support required to scale-up and sustain school-based COVID-19 testing: Qualitative findings from a non-inferiority trial in three predominately latinx-serving middle schools in San Diego County, California

#### Susan M. Kiene^1^, Amanda Miller^1^, Diego Ceballos^1^, Cynthia Sanchez^1^, Jamie Moody^1^, Eyal Oren^1^, Lynnette Famania-Martinez^2^, Rebecca Bravo^2^, Vernon Moore^2^, Corinne McDaniels-Davidson^1^

##### ^1^San Diego State University, San Diego, CA, USA; ^2^Sweetwater Union High School District, Chula Vista, CA, USA

###### **Correspondence:** Susan M. Kiene (skiene@sdsu.edu)

*Implementation Science 2024*, **19(2):**S132

**Background:** Equitable access to simple, convenient, regular at-home COVID-19 testing for middle school students, staff, and their families in underserved communities can have substantial impact on reducing household, school, and community transmission. We qualitatively explored the experiences of key stakeholders participating in school-based COVID-19 testing programs to assess acceptability, appropriateness and feasibility and identify modifiable aspects that could improve intervention efficacy.

**Methods:** *Communities Fighting COVID!: Returning our Kids Back to School Safely* (“RTS”) was a non-inferiority trial comparing school-based distribution of at-home COVID-19 tests (intervention) to onsite school-based testing (control) in three predominately Latinx-serving middle schools in San Diego County. Following the 21-week trial, 14 in-depth feedback and debriefing sessions (n=19; with school and district staff, and study staff) and 13 parent listening sessions (n=30) were conducted in Spring 2022 to assess implementation processes and outcomes prior to scale-up. All sessions were recorded and transcribed. The Consolidated Framework for Implementation Research (CFIR) and implementation outcomes defined by Proctor et al. informed interview guide development, codebook development, analysis, and interpretation.

**Findings:** CFIR offered a comprehensive framework for organizing determinants of RTS implementation success and refining our approach for district-wide scale-up. The inner setting domain construct of leadership engagement and the intervention characteristics domain constructs of adaptability and relative advantage were the most salient, providing concrete guidance for scale-up, such as the need to (1) adopt strategies to promote awareness of RTS among school staff and leadership at roll out, (2) have full time program staff on campus and (3) extend hours to accommodate parents. Findings from the outer setting domain provided explanation of differing community needs and ideologies and their influence on acceptability and appropriateness of RTS at a given school.

**Implications for D&I Research:** The COVID-19 pandemic required timely roll out of preventative services to reduce community spread. Use of CFIR to guide data collection and analysis ensured consideration of implementation constructs across CFIR domains and supported identification of modifiable barriers to implementation success early in the research pipeline. The CFIR will be used to assess implementation during scale-up to continue refinement of implementation processes.

**Primary Funding Source:** National Institutes of Health

### S133 Applying the RE-AIM framework to guide the evaluation of a mobile market-based fruit and vegetable incentive program pilot for lower-income seniors

#### Angelica Tutasi-Lozada^1^, Leah Vermont^2^, Lucia Leone^3^

##### ^1^University at Buffalo, Buffalo, USA; ^2^University at Buffalo School of Public Health, Buffalo, NY, USA; ^3^University at Buffalo, Buffalo, NY, USA

###### **Correspondence:** Angelica Tutasi-Lozada (adtutasi@buffalo.edu)

*Implementation Science 2024*, **19(2):**S133

**Background:** Produce incentive programs historically aimed to increase fruit and vegetable (FV) purchases by lower-income consumers at farmers' markets or other unaffordable locations. Mobile produce markets, targeting lower-income consumers, face challenges implementing traditional incentives. To address this, the Veggie Van Training Center developed the Senior Mobile Market Loyalty Program (SMMLP), overcoming barriers for seniors in utilizing produce incentives based on partner feedback.

**Methods:** The implementation evaluation of SMMLP pilot was guided by the RE-AIM framework. Data for the evaluation were collected from 11 organizations operating mobile markets in communities serving older adults through multiple sources, including surveys administered to participants, interviews with mobile market operators, and sales data. Using point-of-sales software, mobile markets automatically applied a $5 discount to customers' purchases whenever they visited a participating weekly mobile market. Participants were eligible if they were 50+ years old and 200% or less of the federal poverty level.

**Findings:** *Reach -* 889 customers enrolled. Data from 142 individuals with complete follow-up and purchasing data showed that 50 % visited the market at least once per month. *Effectiveness* - 89% of customers reported a positive impact in improving FV consumption. Customers visiting the market once or more monthly consumed an average of 3.2 servings of vegetables per day compared to 2.5 servings for less frequent shoppers. *Adoption* - Eleven out of 15 interested organizations implemented SMMLP. Non-implementation was due to survey data collection burdening mobile market operations. *Implementation* - Market operators highlighted SMMLP's benefits for seniors, including a simpler registration process and extended operating period. 85% of participants expressed a desire to continue shopping at the market even if SMMLP ended, and all partners wanted to continue the program if funding was available, with 30% willing to continue even without funding.

**Implications for D&I Research:** This study demonstrates a successful pilot adaptation of produce incentive programs to mobile markets targeting senior and lower-income consumers. It highlights the importance of simplified registration and data collection processes, and extended operating periods. The findings show strong support for SMMLP continuation and contribute valuable insights for policy regarding federal/state produce incentive programs.

**Primary Funding Source:** AARP Foundation

### S134 Are federal nutrition support program outreach requirements feasible at the local level? Exploring innovative opportunities for cross program connections

#### Yuka Asada^1^, Anna DiStefano^1^, Erin Raczynski^2^

##### ^1^University of Illinois Chicago, Chicago, IL, USA; ^2^Arizona Department of Health Services, Phoenix, AZ, USA

###### **Correspondence:** Erin Raczynski (Erin.Raczynski@azdhs.gov)

*Implementation Science 2024*, **19(2):**S34

**Background:** Federally-funded nutrition support programs—such as the Special Supplemental Nutrition Program for Women, Infants, and Children (WIC) and Child and Adult Care Food Program (CACFP)— promote food security for low-income families and children but suffer from chronic underutilization. Federal policies require cross program outreach but are ineffectively implemented. The Arizona State Nutrition Action Committee formed a trans-agency collaborative to build local level connections between WIC and CACFP-participating early childcare and education (ECE) facilities. This qualitative pilot study engaged a practitioner advisory board of WIC and ECE facility directors with an objective to understand implementation feasibility and equity considerations for cross-program connections and outreach activities.

**Methods:** The study was guided by the EquIR (Equity in Implementation Research) framework to intentionally examine equity-centered policy implementation determinants and strategies. We conducted virtual interviews with WIC and ECE directors across urban and rural counties in Arizona (n=21) and two state officials (n=2) between Jan-Jul 2023. Interviews were transcribed and uploaded into MAXQDA for organization and team coding. Two analysts iteratively created a codebook and met frequently to gauge intercoder agreement; MAXQDA tools, such as summary grids and visual tools, were utilized to confirm and disconfirm trends in the data. Themes were generated using a hybrid analysis approach.

**Findings:** In the wake of two public health crises—the COVID-19 pandemic and national infant formula shortage—directors from both WIC and ECE facilities reported ongoing challenges with staff shortages and recruitment that hinder local capacities for conducting outreach. Directors in both programs also noted a key strategy to promote feasibility is cross-program trainings to improve knowledge while dispelling myths of each program (e.g., eligibility requirements); in addition, data sharing policies between agencies and addressing staff shortage would also improve cross-program referrals. Participants noted equity considerations that include a) rural and small WIC agencies; b) WIC agencies that serve refugee populations; and c) continuing to offer COVID-era virtual certification.

**Implications for D&I Research:** This study provides actionable strategies for state departments to implement in support of local cross-program referrals to improve outreach and accessibility of critical safety net programs.

**Primary Funding Source:** UIC SPH Seed Grant

### S135 Implementation evaluation and sustainment of an opioid overdose education and naloxone distribution program in VA homelessness and housing programs

#### Sarah Javier^1,2^, Angela Kyrish^3^, Hannah Cheng^1^, Madeleine Golding^2^, Taryn Perez^2^, Kenneth Bruemmer^4^, Amanda Midboe^1,2,5^

##### ^1^Stanford University School of Medicine, Stanford, CA, USA; ^2^Veterans Health Administration, Center for Innovation to Implementation (Ci2i), VA Palo Alto Health Care System, Menlo Park, CA, USA; ^3^Veterans Health Administration, Department of Veterans Affairs, Bedford, MA, USA; ^4^Veterans Health Administration, VA Homeless Programs Office, Washington, DC, USA; ^5^University of California Davis School of Medicine, Davis, CA, USA

###### **Correspondence:** Sarah Javier (Sarah.Javier@va.gov)

*Implementation Science 2024*, **19(2):**S135

**Background:** The Homeless Overdose Prevention Expansion (HOPE) is an implementation trial that utilizes education, audit/feedback, and targeted outreach as strategies for implementing opioid overdose education and naloxone distribution (OEND), an evidence-based intervention for decreasing opioid overdose, in VA homelessness and housing programs. We conducted a formative evaluation informed by the Dynamic Sustainability Framework (DSF) across two phases to support HOPE implementation and sustainment in four Department of Housing and Urban Development-VA Supportive Housing (HUD-VASH) settings in the western U.S.

**Methods:** Using a longitudinal qualitative research design, we conducted semi-structured interviews informed by DSF with HUD-VASH leaders, social workers, and prescribers at HOPE implementation sites (N=4) over two phases (i.e., pre-implementation and during implementation). We analyzed interview data using rapid directed content analysis guided by DSF constructs.

**Findings:** We interviewed 51 HUD-VASH leaders, social workers, and site prescribers during pre-implementation. For Practitioners (Domain: Intervention), interviewees disagreed about who should deliver OEND (e.g., social workers vs. prescribers) to at-risk veterans. For Staffing (Domain: Practice Setting), interviewees perceived that implementation would be difficult due to understaffing and workload demands, as three out of four implementation sites had caseloads above the nationally recommended average of 20-25 veterans per social worker. For Population Characteristics (Domain: Ecological Systems), interviewees across sites reported that HUD-VASH veterans are hard-to-reach and often lack a mailing address in their medical record, rendering implementation difficult.

During implementation, we re-interviewed 13 HUD-VASH leaders, HUD-VASH social workers, and site prescribers and conducted eight additional interviews to gauge implementation progress. For Information Systems (Domain: Practice Context), a lack of standardized clinical documentation across sites impacted implementation. For Supervision (Domain: Practice Context), sites with leaders who developed tailored, internal processes had higher implementation success than sites whose leaders did not. For Policy (Domain: Ecological Systems), federal initiatives such as the White House’s 2022 National Drug Control Strategy and local OEND initiatives influenced some sites’ implementation progress.

**Implications for D&I Research:** Our findings highlight dynamic factors that influence implementation of OEND early-on and how these factors encourage adaptations that optimize sustainment down the road. Given the public health impact of opioid overdoses in this population, our evaluation is timely and critical.

**Primary Funding Source:** Department of Veterans Affairs

## Promoting Health Equity and Eliminating Disparities

### S136 Adapting and implementing a culturally-centered, evidence-based mental health intervention for tribal communities in the southwest: Family listening program

#### Lorenda Belone^1^, Prajakta Adsul^1,2^, Ardena Orosco^3^, Kevin Shendo^4^, David J Tsosie^5^, Mario Atencio^6^, Margaret Garcia^7^, Belkis Jacquez^8^, Beverly Gorman^8^, Vince Werito^8^, Rebecca Rae^8^, Nina Wallerstein^1^

##### ^1^University of New Mexico, Albuquerque, NM, USA; ^2^Ramah Navajo School Board Inc, Albuquerque, NM, USA; ^3^Mescalero Apache Community, Albuquerque, USA; ^4^Jemez Pueblo Community, Albuquerque, USA; ^5^Nahata Dziil Community, Albuquerque, USA; ^6^Torreon/Star Lake Community, Albuquerque, USA; ^7^Santa Ana Pueblo Community, Albuquerque, USA; ^8^University of New Mexico, Albuquerque, USA

###### **Correspondence:** Lorenda Belone (lorenda@unm.edu)

*Implementation Science 2024*, **19(2):**S136

**Background:** Given the health inequities experienced by American Indian (AI) communities, a community and culture-centered approach can improve health outcomes and processes to address structural barriers and facilitators by the active participation of the community in research. Rooted in indigenous history and developed using Community-based Participatory Research (CBPR) approaches, the Family Listening Program (FLP) is an evidence-based, culture-centered intervention that has shown to improve the mental health for children among AI communities. With this novel evidence in place and the potential for such a program to benefit tribal communities, there is an urgent need to identify strategies that can facilitate FLP implementation across Indian country.

**Methods:** Supported by the National Institutes of Health and guided by the Exploration, Preparation, Implementation, and Sustainment Model, we systematically studied the adaptation and implementation processes among three new tribal communities in the Southwest that adopted FLP for their communities. We present formative data surrounding the adoption process, periodic reflections to outline the adaptations to the program, and provide a step-by-step implementation guide informed by real-world implementation challenges. All data were recorded in meeting notes and analyzed for highlighting the adaptation and implementation process in tribal communities.

**Findings:** Implementation involved establishing community champions through the creation of advisory boards, building capacity for research in the communities through training and coaching, and engaging in a systematic process to plan and implement the newly adapted FLP program in the communities. Most adaptations focused on the 12-week FLP curriculum that was culturally-centered by each of the three new communities using peer-to-peer guidance from three long-standing tribal research teams who created the original Navajo, Pueblo, and Apache FLP curricula through previous NIH funding. Each new community had slightly different approaches to adaptations that created capacity and ownership to implement the program in their own community with a focus on sustainability.

**Implications for D&I Research:**

Study findings contribute to a deeper understanding of implementation processes, including community-driven adaptations, that are critical to tribal communities through the use of CBPR and culture-centered approaches. Such a process can enable native communities to integrate evidence-based programs through culturally-centered implementation.

**Primary Funding Source:** National Institutes of Health

### S137 Accelerating the translation of a community-based intervention into practice to promote physical activity in rural cancer survivors

#### Scherezade Mama^1^, Trevor Davis^2^, Jennifer Selman^2^, Lily Ju^1^, Brandy Friendly^1^, Ian Leavitt^1^, Laura Rogers^3^, Kathryn Schmitz^4^, Lorna McNeill^1^

##### ^1^The University of Texas MD Anderson Cancer Center, Houston, TX, USA; ^2^Kourage Health, Tyler, TX, USA; ^3^University of Alabama at Birmingham, Birmingham, AL, USA; ^4^University of Pittsburg Medical Center, Pittsburg, PA, USA

###### **Correspondence:** Scherezade Mama (skmama@mdanderson.org)

*Implementation Science 2024*, **19(2):**S137

**Background:** Rural cancer survivors are less likely to meet physical activity recommendations than urban cancer survivors, contributing to physical inactivity-related cancer health disparities. Existing evidence-based physical activity interventions were developed in urban or clinical settings and often fail when implemented in rural community settings. We used a community-engaged staged approach to adapt an evidence-based intervention to reduce psychosocial stress and increase physical activity in rural breast cancer survivors and implement it in a rural community setting.

**Methods:** First, survey and interview data were used to adapt the intervention to rural cancer survivors. Next, community organizations that serve rural cancer survivors were identified and completed a survey to inform setting-level adaptations, guided by the Consolidated Framework for Implementation Research (CFIR). To accelerate the translation of the adapted intervention into practice, we worked with community partners to understand the implementation context and further adapt and implement the intervention in Northeast Texas.

**Findings:** Rural cancer survivors (n=219) completed surveys and 38 rural breast cancer survivors completed interviews to identify multilevel determinants of leisure-time physical activity adoption and maintenance. Next, 93 community organizations provided input on key attributes that may influence successful implementation of a physical activity intervention. Findings from these stages were used to adapt an evidence-based intervention for rural cancer survivors and for implementation in a rural community setting. Community partners provided additional context and aided implementation through additional connections with churches and trusted community leaders to reach potential implementation sites and participants. This resulted in identification of a local organization interested in implementing the adapted intervention, thereby accelerating translation of the intervention into practice. The resulting Mind Your BEAT intervention combines aerobic exercise, group education, and mind-body strategies to increase physical activity and reduce psychosocial distress in rural breast cancer survivors.

**Implications for D&I Research:** This study underscores the importance of community input in the adaptation of evidence-based interventions to improve reach and implementation. Identifying community partners who are ready, willing, and able to implement programs can accelerate the translation of evidence-based programs and reduce the research-practice gap.

**Primary Funding Source:** National Institutes of Health

### S138 Cooperative extension as a century-old implementation laboratory: An overview of extension training and infrastructure to improve health equity in rural population

#### Samantha Harden^1^, Nithya Ramalingam^2^, Thomas Strayer^3^, Meghan Wilson^4^, Laura Balis^5^, April Payne^6^, Rebecca Gartner^7^

##### ^1^Virginia Tech, Blacksburg, VA, USA; ^2^Oregon Health and Science University, Portland, OR, USA; ^3^Vanderbilt University Medical Center, Nashville, TN, USA; ^4^Bluefield University, Bluefield, VA, USA; ^5^Gretchen Swanson Center for Nutrition, Omaha, NE, USA; ^6^Virginia Cooperative Extension, Spotsylvania, VA, USA; ^7^Virginia Cooperative Extension, Culpeper, VA, USA

###### **Correspondence:** Samantha Harden (harden.samantha@vt.edu)

*Implementation Science 2024*, **19(2):**S138

**Background:** The Cooperative Extension System (CES) has been an exemplar dissemination and implementation laboratory for over 100 years with a bidirectional pipeline from university-based specialists to county-based agents. However, the system status quo (like many other public health entities) related to capacity building for the integration of evidence-based interventions (EBIs) often hinges on a one-time agent training delivered by specialists. Iterative processes that equally value specialist and agent needs may improve the uptake and scalability of EBIs in CES.

**Methods:** A research-practice partnership (RPP) among agents and specialists engaged in annual assess, plan, do, evaluate report (APDER) iterative processes to collate data and determine next steps for the integration of EBIs. Overall, data collection practices included pre- and post- training surveys (demographic characteristics, program perceptions, predictors of implementation, intent to deliver, and training satisfaction); one-on-one interviews with county-based agents who implemented the program within one year of the training (n = 5) and randomly selected non-implementers (n = 4; 40% of non-implementers); a training implementation checklist. These data were presented at RPP meetings > one time per year to determine future directions.

**Findings:** Quantitatively, non-implementers were significantly more likely to report low capacity and self-efficacy for delivery of physical activity interventions compared to implementers. Qualitatively, both implementers and non-implementers reported high training satisfaction, the need for structured peer support, and a desire for ongoing training. The training checklist tracked real-time adaptations but was cumbersome to complete. Based on these data, the RPP proposed the following changes: (1) district-wide trainings (instead of state-wide); (2) co-facilitated training with agents who previously delivered the EBI; and (3) the ability for trained agents to train volunteers in their district (e.g., train-the-trainer).

**Implications for D&I Research:** Agents of CES provide real-world, feasible solutions to advance training and technical assistance. Specialists can provide tools, checklists, and measurement to data to inform ongoing program adaptations. Together, the RPP can build state and national capacity for EBI integration.

### S139 Assessing organizational determinants for implementation of the national diabetes prevention program in rural populations through the cooperative extension system

#### Anna Gorczyca^1^, Mackenzie Koester^2^, Patricia Smith^3^, Gerit Wagner^1^, Kameron Suire^1^

##### ^1^University of Kansas Medical Center, Kansas City, KS, USA; ^2^Weitzman Institute, Middletown, CT, USA; ^3^University of Colorado Anschutz Medical Campus, Denver, CO, USA

###### **Correspondence:** Anna Gorczyca (agorczyca@kumc.edu)

*Implementation Science 2024*, **19(2):**S139

**Background:** The National Diabetes Prevention Program (National DPP) has been effective at preventing type 2 diabetes across the U.S but has not had the population health impact intended, especially in rural populations. The Cooperative Extension System (CES), the outreach and community engagement arm of land grant universities, could improve the reach and accessibility of the National DPP to rural populations through Family and Consumer Science (FCS) professionals tasked with delivering health promoting programs within their local community. Currently, 23 CES organizations are CDC-recognized National DPP providers; however, there is limited evidence on organizational determinants to support implementation of health promoting programs. Guided by the Consolidated Framework for Implementation Research (CFIR), we assessed factors influencing health promotion program success in CES to identify implementation strategies for future research.

**Methods:** Guided by CFIR, individual structured interviews were conducted with CES FCS agents (n = 11) to acquire information on previous experience with health promoting program implementation. Data were analyzed thematically using a deductive approach.

**Findings:** Three themes emerged from the interviews: Barriers to recruitment included program interest, lack of relationships with participants, timing/location of programs, and competing family demands. Recruitment facilitation involved providing participant incentives, collaborating with community partners, and leveraging social media for marketing purposes. Community connections played a pivotal role in facilitating successful programming. Healthcare and faith-based organizations, local press, resource centers (food banks), government entities, senior and childcare centers were utilized to various degrees. Program implementation is a key responsibility for FCS agents with reported challenges of location accessibility, program recruitment, retention, and language barriers (i.e., an inability to communicate with community members or access to Spanish speaking resources). Successes largely focused on program registration, incentives for participation and practical location of program delivery. Common solutions for program implementation included building community trust, marketing considerations for recruiting rural populations and program topics of interest.

**Implications for D&I Research:** CES appears to be a viable system for delivery of the National DPP to rural populations where healthcare resources are substantially limited. Future research should assess recruitment methodology and collaboration with rural healthcare organizations to refer patients to the National DPP to improve rural-urban health disparities.

**Primary Funding Source:** National Institutes of Health

### S140 Engaging patients and clinicians to co-create feasible and sustainable approaches to implement evidence-based cancer control.

#### Monica Perez Jolles

##### University of Colorado, Denver Anschutz Medical Campus, Aurora, CO, Aurora, CO, USA

###### **Correspondence:** Monica Perez Jolles (MONICA.JOLLES@CUANSCHUTZ.EDU)

*Implementation Science 2024*, **19(2):**S140

**Background:** To guide cancer treatment decisions for older adults (>65 age), geriatric and patient-centered risk assessments are recommended, including social determinants of health (SDoH) and behavioral risk factors that influence patient care. This pilot study uses co-creation engagement with a multi-perspective steering committee, clinic-based workshops, and a diverse group of patients. The goal is to integrate screenings in areas relevant to older adult cancer patients in a way that is feasible, actionable, equitable, and sustainable. This co-creation engagement approach will improve the alignment of cancer treatment decisions for older cancer patients - the resulting package is termed the "Integrated Aging Assessment for Action in Cancer Patients" (IA3-CP).

**Methods:** To develop the IA3-CP tool, we used a co-creation engagement approach with partner clinics and patients, and form-function methods to develop workflow processes to feasibly integrate the tool into the oncology teams' cancer treatment planning processes. We conducted 45-60-minute workshops with clinic personnel and patients to specify the core functions and forms of the IA3-CP and clinical workflows needed, with a focus on equity.

**Findings:** We engaged professional partners with diverse roles across three oncology clinics (n=15) in the University of Colorado Cancer Center who participated in the co-creation IA3-CP design and planning process. Older cancer patients (n=5-10) ages >65 years participated in the workshops and user test sessions. Partners informed IA3-CP's core goals or functions (e.g., function priority for patients such as service linkage after screenings), acceptability, potential challenges to its equitable implementation, and suggestions for workflow. The Function of ‘completion of the IA3-CP’ will take a different form in the solid tumor clinics than in the blood clinic based on clinic characteristics (e.g., preference for completion prior to a visit or in the waiting room).

**Implications for D&I Research:** The IA3-CP implementation package has great potential to allow busy oncology practices conduct evidence-based screenings tailored to clinic workflows and provide equitable patient care with attention to SDoH. Our co-creation method used to develop the IA3-CP is an emerging and generalizable D&I science method with great potential to engage diverse partners and enhance attention to equitable partner perspectives in research.

**Primary Funding Source:** UCD internal funding

### S141 The oasis trial as a model for equitable co-creation and adaptation of an implementation blueprint: A partnership with the national community oncology research program (NCORP) community sites (WF-20817CD)

#### Kristie Foley

##### Wake Forest University School of Medicine, Winston Salem, NC, USA

###### **Correspondence:** Kristie Foley (kfoley@wakehealth.edu)

*Implementation Science 2024*, **19(2):**S141

**Background:** An implementation blueprint is a comprehensive guide of strategies, timelines, and personnel necessary for implementing evidence-based practices. They may also be used to document adaptations during the implementation process. This case study describes co-creation of an implementation blueprint to promote equity in decision-making and responsibility for implementing tobacco use treatment (TUT) in diverse imaging facilities.

**Methods:** The OaSiS Trial was a hybrid-effectiveness implementation trial designed to integrate TUT into imaging facilities for patients undergoing lung cancer screening (LCS) (NCT03291587). With support from the research team, 12 facilities developed implementation blueprints during an in-person strategic planning session derived from observation of the LCS workflow, operations, and staff engagement. After co-creation, blueprints guided coaching calls that fostered implementation. We describe the process of co-creation and the value of a co-created blueprint to foster equitable engagement of clinic staff.

**Findings:** A total of 104 health system staff (x=8.5/facility) participated in the co-design process. Staff routinely included the director of imaging, CT coordinators and technicians, nurse navigators, and smoking cessation personnel. OaSiS team members participated by offering information on the efficacy of different TUT services, but did not expect teams to adopt TUT strategies and timelines that were infeasible for the imaging setting. Facility implementation champions (n=19) attended all 70 calls (x=6/facility) after the blueprints were created. Coaching calls lasted an average of 30 minutes and included 3 members of the blueprint implementation team. ***All 12 radiology facilities adapted the blueprint during the trial.*** Common adaptations included adding active referral to health system TUT services, adding CT tech training, changing the imaging report to include access to cessation services, abandoning e-referral for smoking cessation, and moving signage regarding quitting for greater visibility.

**Implications for D&I Research:** Co-creation of the implementation blueprint engaged diverse members (e.g., CT techs, manager) of the imaging team and fostered new collaborations with smoking cessation personnel outside the radiology clinic. While a large number of people participated in co-design, a smaller implementation team led adaptations when strategies, timelines, or personnel changed. This process ensured that the blueprint was a living and locally-driven document roadmap and ensured TUT strategies that were adopted were also sustainable.

**Primary Funding Source:** National Institutes of Health

### S142 Considering co-creation among research practices in support of cancer prevention and control research network’s racial and health equity principles

#### Prajakta Adsul

##### University of New Mexico, Albuquerque, NM, USA

###### **Correspondence:** Prajakta Adsul (padsul@salud.unm.edu)

*Implementation Science 2024*, **19(2):**S142

**Background:** There is growing recognition and consideration for health equity-oriented research in implementation science. Researchers from the Health Equity Workgroup of the Cancer Prevention and Control Research Network (CPCRN), have defined and operationalized nine racial and health equity principles for cancer prevention and control, of which one is geared towards co-creation with community members and implementation partners.

**Methods:** The Health Equity Workgroup utilized a multi-phase, participatory consensus-building approach to: identify recurrent themes in health and racial equity frameworks, capture perspectives on and experiences with health equity research through an online survey; and engage in recurrent subgroup meetings and consensus-building discussion to refine the guiding principles. Overall, 28 survey respondents and 79 meeting participants provided data, feedback, and endorsements of the health equity principles and identified research practices following the principles.

**Findings:** Survey data, literature reviews, and investigator engagement together informed the development of “Equitable Research Collaborations Toolkit”. The Toolkit has nine sections organized according to each principle with four sub-sections that operationalize the principle for research collaboration; current or historical practices undertaken in CPCRN projects; reflection questions to guide researchers; and resources for methods of assessment. This talk will focus on principle two, which suggests that cancer prevention and control research should address community priorities through engagement and co-creation. We operationalized this principle as conducting and analyzing needs collaboratively, establishing shared goals and objectives, and integrating community input into recruitment and retention strategies to ensure equitable representation. CPCRN investigators reported including community members as co-investigators, establishing formal community partnerships, and co-creating linguistically and culturally centered intervention materials. Within the Toolkit, we provided researchers with a set of reflection questions and resources for assessing co-creation methods, processes, and outcomes as they contribute to equity-oriented cancer prevention and control research.

**Implications for D&I Research:** Co-creation with community members and implementing partners is recognized as a critical aspect of promoting cancer prevention and control research and practice, especially as it focuses on implementation science. Future directions include developing an evaluation framework for CPCRN projects to assess progress made towards advancing the co-creation methods for implementation science focused cancer prevention and control research.

**Primary Funding Source:** UCD internal funding

### S143 Promises and challenges of bidirectional collaboration for use of research evidence in child welfare systems

#### Cathleen Willging^1^, Elise Jaramillo^1^, Tiffany Lagare^2^, Gregory Aarons^3^, Danielle Fettes^4^

##### ^1^Pacific Institute for Research and Evaluation, Albuquerque, NM, USA; ^2^University of California, San Diego, San Diego, CA, USA; ^3^UC San Diego Child and Adolescent Services Research Center, San Diego, CA, USA; ^4^University of California, San Diego, La Jolla, CA, USA

###### **Correspondence:** Cathleen Willging (cwillging@pire.org)

*Implementation Science 2024*, **19(2):**S143

**Background:**

Children in child welfare systems face a myriad of poorer mental health, developmental, and social outcomes than their peers. These systems are hierarchically-organized bureaucracies governed by federal, state, and local regulations. The use of research evidence (URE) in such settings may enhance service provision and reduce health-related inequities for system-involved children by positively impacting outcomes. Per Cultural Exchange Theory, community-based partnerships among academic researchers, policymakers, and frontline practitioners may facilitate evidence-informed policy and service provision in child welfare systems. The bidirectional sharing of knowledge and power is a central tenant of successful research partnerships, celebrated for its potential to build trust, integrate URE into complex systems, and align research with system-partner and service-user needs. This analysis draws from a richly descriptive qualitative dataset concerning one such partnership—the Community-Academic Partnership for Translational Use of Research Evidence (CAPTURE).

**Methods:**

We collected and analyzed three waves of qualitative data—in 2018, 2019, and 2020-21—to assess the dynamics of bidirectional collaboration from the vantage points of steering committee members and others involved in CAPTURE. Data included observations of steering committee proceedings, semi-structured interviews, and member checking. We analyzed data iteratively using open- and focused-coding techniques and sensitizing concepts from Cultural Exchange Theory.

**Findings:**

Bidirectional collaboration in CAPTURE reveals the (a) difficulties of establishing capacity and a shared language for URE in a public-sector system lacking a strong research culture; (b) the promise yet subsequent neglect of URE as “proof” to inform system decisions related to policies and programs; (c) slow-moving bureaucratic processes shaped by competing priorities that work against URE; and (d) the nuances of sharing power that necessitate concessions that can undermine trust and bidirectional URE.

**Implications for D&I Research:**

CAPTURE aims to respond to growing pressures on child welfare systems to support evidence-based policies and programs. However, creating a bidirectional cultural exchange for URE takes time and perseverance to nurture in systems not traditionally oriented toward generating and applying research evidence. This exchange also requires substantial commitment and resources from academic and system-level partners to overcome entrenched power dynamics and bureaucratic processes that can hinder development of collaborative cultures and sustainable URE.

**Primary Funding Source:** William T. Grant Foundation

### S144 A community-engaged process for adapting a cardiovascular health intervention for persons with serious mental illness

#### Christina Yuan^1^, Gail Daumit^2^, Lisa Cooper^2^, Courtney Cook^2^, Casey Corches^3^, Arlene Dalcin^2^, Benjamin Eidman^2^, Tyler Fink^2^, Joseph Gennusa^2^, Stacy Goldsholl^2^, Celeste Liebrecht^3^, Eva Minahan^2^, Brianna Osorio^3^, Shawna Smith^4^, Nae-Yuh Wang^2^, Emily Woltmann^3^, Amy Kilbourne^5^

##### ^1^Johns Hopkins Bloomberg School of Public Health, Baltimore, MD, USA; ^2^Johns Hopkins University School of Medicine, Baltimore, MD, USA; ^3^University of Michigan Medical School, Ann Arbor, MI, USA; ^4^University of Michigan School of Public Health, Ann Arbor, MI, USA; ^5^University of Michigan Medical School, Department of Learning Health Sciences, Ann Arbor, MI, USA

###### **Correspondence:** Christina Yuan (cyuan16@jhmi.edu)

*Implementation Science 2024*, **19(2):**S144

**Background:** Persons with serious mental illness experience grave disparities in cardiovascular disease risk factors and behaviors, which drives mortality rates that are two to three times higher than the overall population. To scale-up effective cardiovascular disease risk reduction interventions from clinical trials to community-based settings, the literature points to the value of engaging community partners in adapting interventions but offers little guidance on *how* to do so. We describe a novel, theory-informed process of garnering community input to adapt an evidence-based intervention for improving cardiovascular disease risk factors in persons with serious mental illness.

**Methods:** Our process is based on the Enhanced Replicating Effective Programs (E-REP) framework, a systematic approach for implementing interventions into community settings that maximizes fidelity to the interventions’ core elements while incorporating end-users’ input to enhance fit. We use the Stakeholder Engagement Reporting Questionnaire to describe two community engagement activities leveraged during our study’s pre-implementation phase: (1) a “needs assessment,” including site-level surveys (N=26) and individual interviews (N=94), to identify anticipated implementation barriers and facilitators; and (2) a series of “community working groups” with clinicians and staff (mean: 24 per meeting) and persons with serious mental illness (mean: 8 per meeting), which used a focus group format to collaboratively engage end-users in the adaptation process.

**Findings:** Rapid analysis of the needs assessment data identified several key barriers (e.g., high staff turnover) that informed the intervention experts’ initial adaptations of the intervention (e.g., plans to develop online training modules to rapidly onboard new staff). The needs assessment also informed the composition and content of community working groups, which actively involved implementers and recipients in tailoring the intervention and strategies further. Suggested adaptations have been used to enhance the acceptability of the intervention’s packaging into more user-friendly components, increase flexibility in the modes of intervention delivery, and refine how training, coaching, and facilitation are operationalized in an upcoming hybrid type 3 trial.

**Implications for D&I Research:** Theory-based implementation strategies such as E-REP, which uses mixed methods and user-centered design processes, offer a promising approach to adapting interventions that can advance health equity through a systematic process that promotes shared decision-making and ownership by community partners.

**Primary Funding Source:** National Institutes of Health

### S145 Community-level implementation to promote health equity: Adaptation of cancer screening communication strategies to low-resource settings

#### Brooks Harmon^1^, Ajay Myneni^2^, Hilary Kirk^1^, Melissa Wanzer^3^, Michele Hubert-Fiscus^3^, Michelle Wysocki^4^, Armin St. George^5^, Jolean Sheffield^5^, Alidad Ghiassi^5^, Heather Orom^1^, Laurene Tumiel-Berhalter^2^, Detric Johnson^6^, Stuti Tambar^2^, Katia Noyes^1,2^

##### ^1^School of Public Health and Health Professions, University at Buffalo, Buffalo, NY, USA; ^2^Jacobs School of Medicine and Biomedical Sciences, University at Buffalo, Buffalo, NY, USA; ^3^Great Lakes Cancer Care Collaborative, Buffalo, NY, USA; ^4^Erie County Department of Health, Buffalo, NY, USA; ^5^HIA Technologies, Inc, Pasadena, CA, USA; ^6^The National Witness Project, Buffalo, NY, USA

###### **Correspondence:** Katia Noyes (enoyes@buffalo.edu)

*Implementation Science 2024*, **19(2):**S145

**Background:**

Misinformation about procedure discomfort, insurance co-pay and referral process, along with inadequate patient-provider communication can significantly reduce access to cancer screening, especially among people of color and those with low health literacy. Developing culturally tailored and interactive educational videos (Aivio™) for specific populations is a potentially effective and scalable approach to improve patient knowledge and change attitude towards screening. Evidence on effectiveness of mobile patient education interventions to improve cancer screening among minority groups is limited and conflicting. Using a diverse, multidisciplinary team of stakeholders, this study aimed to 1) identify community information needs and message receptivity to guide preparation of culturally tailored videos; and 2) identify patient-preferred dissemination strategies for cancer screening educational videos.

**Methods:**

The research team employed a storytelling approach to develop a case study scenario using a culturally representative avatar character based on current evidence about barriers to cancer screening among people of color in Western New York. Using iterative feedback-adaptation cycle the initial interactive video and avatar was revised based on focus groups (n=2, 11 and 8 people) and individual semi-structured interviews (n=8) with patients, patient advocacy groups, area health communicators, cancer and social services care coordination, and breast cancer treatment experts.

**Findings:**

Key reported knowledge gaps included clarification on screening referral pathway and insurance pre-authorization process, information on eligibility for county’s free screening services, and understanding of individual cancer risk. Patients preferred dissemination strategies that use plain language without fear inducing messaging, do not require a visit to a healthcare facility, allow for pause-repeat and share, and include comprehensive information about out-of-pocket costs, waiting times, and what to expect during and after screening.

**Implications for D&I Research:**

Tailoring health interventions for specific dissemination and implementation settings helps align the needs of end users and the intended context for use. Stakeholder involvement is critical for timely and effective translation of research findings into community practice, especially given the importance of addressing structural barriers to cancer screening, including insurance pre-authorization, transportation, eligibility for county cancer services programs to cover treatment, fear of unexpected medical bills, and false-positive results.

**Primary Funding Source:** University at Buffalo CTSI; M&T Bank

### S146 Factors affecting implementation of youth mental health first aid in schools in or near the Cherokee Nation Reservation: A qualitative evaluation

#### Caroline Barry^1^, Elizabeth Walker^2^, Sierra Talavera-Brown^1^, Juli R. Skinner^3^, Terrence Kominsky^3^, Kelli Komro^4^

##### ^1^Emory University, Atlanta, GA, USA; ^2^Emory University, Rollins School of Public Health, Atlanta, GA, USA; ^3^Cherokee Nation Behavioral Health, Tahlequah, OK, USA; ^4^Emory University Rollins School of Public Health, Atlanta, GA, USA

###### **Correspondence:** Caroline Barry (caroline.barry@emory.edu)

*Implementation Science 2024*, **19(2):**S146

**Background:** Efforts to implement culturally relevant interventions to promote mental health and prevent substance misuse among American Indian adolescents have increased; however, Tribal communities face distinct barriers and facilitators. Youth Mental Health First Aid (YMHFA), an evidence-based intervention with potential for positive impact in rural American Indian communities, is a gatekeeper mental health literacy training for adults to identify, assist, and refer youth experiencing mental health or substance use concerns to treatment. The objective of this study was to explore factors affecting implementation of YMHFA in schools in or near the Cherokee Nation Reservation.

**Methods:** YMHFA-certified instructors with experience in local schools were recruited using a community-centered sampling frame from the National Council for Mental Wellbeing’s instructor database and snowball sampling through Cherokee Nation Behavioral Health. Qualitative data were collected via semi-structured in-depth interviews. Interviews were audio recorded, transcribed, and coded using deductive methods based on the Consolidated Framework for Implementation Research and Relational World View model and inductive methods to explore emergent themes. Data were coded in MAXQDA by two coders until consensus was reached, and transcripts were thematically analyzed.

**Findings:** Interviews with 21 participants revealed several key themes. YMHFA was characterized as a useful and appropriate intervention for schools to address the mental health and substance misuse crisis among youth. All interviewees identified social support from teachers as a meaningful intervention opportunity. Significant implementation barriers were time and cost. Time has been a barrier to administrative buy-in, but once implemented, teachers have been satisfied with YMHFA content and report improvements in self-efficacy, knowledge, and skills. Barriers of time, cost, and equipment access are especially prominent for independent instructors. Grant-supported instructors or contractors benefit from established community partnerships and funding mechanisms.

**Implications for D&I Research:** Strong community partnerships can improve intervention implementation. Alongside strengthening community partnerships, the National Council may consider advocating for grants/project-related funding mechanisms and policy change to require trainings in schools among teachers and school staff. Findings provide important insight for interventionists on how to strengthen implementation and sustainability of mental health promotion and substance use prevention in rural American Indian communities.

**Primary Funding Source:** National Institutes of Health

### S147 The role of community partners in implementation of Traditions and Connections for Urban Native Americans (TACUNA), an opioid use disorder prevention intervention for urban American Indian/Alaska Native emerging adults

#### Daniel Dickerson^1^, David Kennedy^2^, Keisha McDonald^2^

##### ^1^University of California, Los Angeles, Los Angeles, CA, USA; ^2^RAND Corporation, Santa Monica, CA, USA

###### **Correspondence:** Daniel Dickerson (daniel.dickerson@ucla.edu)

*Implementation Science 2024*, **19(2):**S147

**Background:** American Indian/Alaska Native (AI/AN) people residing off-reservations are disproportionately affected by opioid, alcohol, and other drug use. More than 87% of AI/AN people reside outside of reservations and tribal lands, yet few promising culturally relevant substance use prevention interventions exist for AI/AN, particularly emerging adults living in urban areas. This study aimed to identify strategies to help disseminate and implement a promising opioid use prevention intervention for urban AI/AN emerging adults.

**Methods:** Three rounds of semi-structured interviews were conducted with Traditions and Connections for Urban Native Americans (TACUNA) research team by the National Institutes of Health (NIH) HEAL Prevention Coordinating Center (HPCC). The purpose of these interviews was to gather information to assist in the eventual implementation of TACUNA more broadly. A subset of the interviews focused on the key components of TACUNA and the role of interested parties and the TACUNA elder advisory board in developing and delivering the intervention. To supplement interviews, research project materials and communications between the HPCC and the TACUNA research and intervention delivery team were reviewed.

**Findings:** Developing a program that integrates AI/AN traditional practices with motivational interviewing and social networks resonates well with the target population. A key facilitator of TACUNA implementation has been collaboration with AI/AN community partners and hiring facilitators within urban communities or who have good rapport within the urban community as well as possess cultural knowledge; such factors helped to garnish trust among participants. Delivering TACUNA virtually and providing 1-hour sessions may help to increase the deliverability of TACUNA, thus having more potential to decreasing the impact of the opioid epidemic among AI/AN people nationally.

**Implications for D&I Research:** We describe approaches to facilitating collaboration with implementation partners and potential adopters of TACUNA for urban AI/AN emerging adults. Community engagement and partnership is pivotal to implementation of culturally relevant interventions for AI/AN. Findings from this study offer helpful guidance on building those relationships that will aid implementation, delivery, and sustainability of TACUNA for urban AI/AN programs.

**Primary Funding Source:** National Institutes of Health

### S148 Feasibility of implementing suicide prevention within a housing first intervention for youth experiencing homelessness

#### Laura Chavez^1^, Brittany Brakenhoff^2^, Alicia Bunger^3^, Ruri Famelia^3^, Kelly Kelleher^1^, Natasha Slesnick^3^

##### ^1^The Abigail Wexner Research Institute at Nationwide Children's Hospital, Columbus, OH, USA; ^2^University of Nebraska- Lincoln, Columbus, OH, USA; ^3^The Ohio State University, Columbus, OH, USA

###### **Correspondence:** Laura Chavez (laura.chavez@nationwidechildrens.org)

*Implementation Science 2024*, **19(2):**S148

**Background:** Suicide and drug overdose are the leading causes of death among youth experiencing homelessness (YEH). Interventions targeting suicide are desperately needed for YEH, many of whom misuse substances, and must be flexible enough to be delivered in nontraditional settings. The present multi-method study examines feasibility and barriers of implementing comprehensive suicide prevention, which included suicide risk screening and cognitive therapy for suicide prevention (CTSP) as indicated, delivered through youth advocates to YEH enrolled in a randomized controlled trial of Housing First (HF).

**Methods:** Youth were randomized to receive either 6 months of risk prevention services combined with HF (n=120), or risk prevention services alone (n=120), and both arms were offered suicide prevention. We examined feasibility of screening for suicide risk and delivering CTSP to those at highest risk. Individual semi-structured interviews, guided by the Consolidated Framework for Implementation Research, were conducted with YEH and youth advocates to further explore implementation barriers and facilitators (n=12). Interviews were transcribed and coded using thematic analysis by two independent coders.

**Findings:** Among 240 youth included in the parent study, all youth were successfully assessed for suicide risk and 64 (26.7%) were identified as high risk, of which 47 (73%) consented to receive CTSP. Both youth and advocate interviews identified prioritizing other needs first as barriers to delivering CTSP. However, building rapport and allowing time to increase trust with advocates, as well as ensuring that other basic needs were met first (e.g., food, safety, shelter), were possible facilitators for youth engagement with CTSP content.

**Implications for D&I Research:** The feasibility of suicide risk prevention interventions among YEH can be difficult to assess but is pivotal for successful implementation. Our findings provide insight on ways to evaluate and strengthen implementation of suicide risk assessments and CTSP among a vulnerable population of youth who are often excluded from traditional treatment settings. The relationships with youth advocates can both improve engagement with YEH and identify key barriers to delivery of suicide prevention interventions in this services delivery context.

**Primary Funding Source:** National Institutes of Health

### S149 Implementation of adverse childhood experiences (ACEs) screenings in clinical and school settings while promoting equity

#### Monica Perez Jolles^1^, Jini Puma^2^, Gabrielle Jacobs^3^, Jenn Leiferman^4^

##### ^1^University of Colorado, Denver Anschutz Medical Campus, Aurora, CO, Aurora, CO, USA; ^2^Colorado School of Public Health, Aurora, CO, USA; ^3^Mind Body Therapy & Coaching Center, Portland, OR, USA; ^4^University of Colorado Anschutz Medical Campus, Aurora, CO, USA

**Correspondence:** Monica Perez Jolles (MONICA.JOLLES@CUANSCHUTZ.EDU)

*Implementation Science 2024*, **19(2):**S149

**Background:** The implementation of risk assessments by professionals to assess the social and behavioral healthcare needs of children and their caregivers is rising. These efforts stem from increased awareness of the role of psychosocial risk factors as key determinants of physical and mental health outcomes among children. One such effort is the implementation of Adverse Childhood Experiences (ACEs) screenings to promote early detection of toxic stress and trauma. ACEs include exposure to abuse, racism, and community violence. States have enacted formal healthcare policies or practice guidelines to support ACEs screenings. Yet, we lack information on tested strategies to adapt these screening practices to be culturally appropriate while assessing fidelity.

**Methods:** We present tested strategies using two community-engaged studies as examples of the implementation of ACEs screenings in under-resourced childhood settings. The 2022-2024 California example is a NIMH-funded Hybrid 2 randomized study using implementation mapping to refine and test a strategy designed to increase the reach, equity-focused adaptations, and service referral of the state’s fee-for-service ACEs Aware policy. The 2019-2024 Colorado example implements a CDC-funded prevention intervention to support ACEs screenings and service referrals in early childhood education centers in a rural, Hispanic community. Multiple data sources inform each study (electronic records data, surveys, interviews, tracking adaptations matrix).

**Findings**: Despite contextual differences, both studies found challenges to ACEs implementation, including caregiver mistrust and workforce burnout. Reach of screenings and service referrals were supported by strategies focusing on increasing ACEs knowledge among professionals, focusing on caregiver-reported need(s) and preferences rather than on ACEs scores, and using peer coaches and family-centered scripts to introduce screenings and guide referrals. Strategies tailored to each context were also identified. Unintended consequences of implementing risk-focused childhood assessments in under-resourced settings, such as increased stigma and caregiver under-reporting of ACEs, were also identified.

**Implications for D&I Research:** The D&I field can play a key role in the implementation of healthcare and community-level implementation of ACEs screenings. The results of these two-state studies on tested family-centered and culturally relevant strategies can help practitioners, policymakers, and researchers better address social determinants of health through a family-centered implementation of pediatric screenings.

**Primary Funding Source:** National Institutes of Health

### S150 Social care integration in primary care practices and cancer care centers in an implementation science center in cancer control implementation laboratory

#### Peggy Hannon^1^, Ashley Johnson^2^, Elizabeth Bojkov^2^, Allison Cole^3^

##### ^1^Health Promotion Research Center, University of Washington, Seattle, WA, USA; ^2^University of Washington, Seattle, WA, USA; ^3^Family Medicine, University of Washington, Seattle, WA, USA

###### **Correspondence:** Peggy Hannon (peggyh@uw.edu)

*Implementation Science 2024*, **19(2):**S150

**Background:** Social determinants of health (SDOH) are nonmedical factors that influence health outcomes. Social care integration includes assessing patients’ SDOH and developing programs to address identified social needs to mitigate their negative health impacts. We explored healthcare systems’ efforts to integrate social care into clinical care and the facilitators and barriers they encounter.

**Methods:** We conducted qualitative interviews with representatives from healthcare systems in the Optimizing Implementation in Cancer Control Implementation Laboratory. Healthcare systems first completed a brief questionnaire to assess the degree to which healthcare systems were currently doing social care integration. The semi-structured qualitative interview guide was based on the Five As - awareness, adjustment, assistance, alignment, and advocacy - for Better Social Care Integration into Health framework. We coded facilitators and barriers to social care integration in line with constructs in the Consolidated Framework for Implementation Research 2.0.

**Findings:** We conducted 12 interviews with 17 representatives from 10 healthcare systems (7 primary care systems and 3 cancer centers). Seven reported having a current social needs screening program, most commonly assessing housing and food security. All health systems, including those saying they did not have a program, reported some level of activity in addressing each of the five As. Examples of commonly reported activities included standardized screening tools (awareness), modification to clinical care (adjustment), identifying and referring patients to community resources for social needs (assistance), and collaboration with government agencies and non-profit organizations (alignment and advocacy). Health systems reported significant barriers to social care integration. Barriers spanned the outer setting (availability of community resources to address SDOH), the inner setting (integrating screening into clinical workflows, EHR challenges), and the implementation process. Health systems also reported facilitators in these areas, but not as frequently as barriers.

**Implications for D&I Research:** A diverse group of primary and cancer care health systems self-reported a range of 5 As activities, including health systems that did not consider themselves as having a social care integration program. D&I science can offer health systems making any attempt at social care integration activities tools and guidance to prioritize the unique barriers they encounter and identify strategies to address the most important barriers.

**Primary Funding Source:** National Institutes of Health

### S151 Pre-implementation refinement of a multimodal intervention to increase use of diabetes technology by black youth

#### Marie Masotya, Sarah McAleer, Mollie Evans, Sarah Ronis, Sarah Macleish

##### UH Rainbow Babies & Children's Hospital, Cleveland, OH, USA

###### **Correspondence:** Marie Masotya (Marie.Masotya@UHhospitals.org)

*Implementation Science 2024*, **19(2):**S151

**Background:**

Diabetes technology, including continuous glucose monitors (CGM), is known to improve clinical outcomes for youth with type 1 diabetes (T1DM). However, non-Hispanic Black are 2.7x less likely to start a CGM and 4.1x less likely to continue use within a year of diagnosis. To improve the uptake and sustained diabetes device use by Black youth with T1DM, this IRB-approved study (UH Cleveland Medical Center IRB #STUDY20221175) aimed to refine a proposed intervention involving an interactive clinician dashboard and patient-facing mobile app supported by community health worker (CHW) visits.

**Methods:** Applying PRISM and the Health Information Technology (HIT) Acceptance Framework for Chronic Diseases’ Management, from January to June 2023 we conducted pre-implementation telephone interviews followed by 3 rounds of focus groups via Zoom (n = 8 groups) with Black young adults with T1DM, parents of Black children with T1DM, and various healthcare partners who work with youth with T1DM . Sessions were audio-recorded, transcribed, and analyzed using content analysis to identify themes related to framework domains. Findings were shared with the intervention team after each round of data collection to iteratively refine app and dashboard features and the CHW intervention.

**Findings:** Patient and parent interviewees (n=17) identified common barriers to use of diabetes technologies including: appearance and size of device, non-adhesion to skin, perceived inaccuracy of glucose readings obtained via CGM, and device maintenance. Healthcare partners (n=10) additionally identified issues with insurance coverage, phone and internet access, and patient knowledge. These barriers were used to define (1) query and response algorithms in the app and dashboard and (2) a CHW checklist-based protocol to identify and address concerns emerging between clinic encounters. Focus group participants affirmed that CHW visits should offer multiple modalities (in-home, neutral location, virtual) and patient outreach incorporating family preference (text, phone, email). Based on focus group feedback, language and visual imagery in the patient-facing app were refined to increase cultural resonance for Black youth.

**Implications for D&I Research:** Implementation frameworks facilitated efficient identification of potential barriers, facilitators, and refinements for proposed intervention to increase device use by Black children and youth with T1DM. The intervention will be tested in a RCT beginning December 2023.

**Primary Funding Source:** National Institutes of Health

### S152 Situating implementation science (IS) in res(IS)tance: Integrating black radical thought in implementation scholarship toward advancing health justice

#### Cory Bradley^1^, Whitney Irie^2^, Elvin Geng^3^

##### ^1^Northwestern Feinberg School of Medicine, Chicago, IL, USA; ^2^Boston College, Boston, MA, USA; ^3^Washington University in St. Louis, St. Louis, MO, USA

###### **Correspondence:** Cory Bradley (bcory@wustl.edu)

*Implementation Science 2024*, **19(2):**S152

**Background:** This conceptual analysis undertakes, through a tenet of Public Health Critical Race Praxis, disciplinary self-critique of the field of implementation science, which takes as it aim to bridge the gap between evidence-based interventions and their practical application. Herein, we highlight a critical oversight in the field's current discourse: the lack of attention given to racialized disparities in dissemination and implementation (D&I) failures. The prevalence of differential gaps in the adoption and utilization of evidence-based interventions among racialized groups in the United States necessitates a comprehensive understanding of the systems perpetuating these disparities.

**Methods:** By exploring and applying insights derived through the scholarship of Black radical tradition of resistance, theories such as the Public Health Critical Race Praxis, Critical Race Theory (CRT), and inclusive of post and anti-colonial studies, and Black feminist studies, dissemination and implementation research can align itself with frameworks that have long centered discourses on power dynamics and racialized oppression.

**Findings:** This alignment presents an opportunity for the field to critically examine and dismantle systems that perpetuate racial inequalities in access to and utility of goods and services, including health interventions. The authors further explore the importance of denormalization in resisting the normalization of racialized inequities, emphasizing the need to challenge assumptions and ideologies that have perpetuated systemic racism. Drawing from the concept of resistance, rooted in the Black radical tradition, implementation science can make stronger contributions to the dismantling of racialized systems and actively work towards health justice.

**Implications for D&I Research:** This is a call for integrating critical perspectives and theories of resistance rooted in Black studies into D&I research to address racialized health disparities and design research translation and implementation strategies and approaches that improve health services and health outcomes for disparity populations.

### S153 Advancing equitable implementation of evidence-based practices in cancer care through behavioral economics

#### Katharine Rendle^1^, Rinad Beidas^2^

##### ^1^Perelman School of Medicine, University of Pennsylvania, Philadelphia, PA, USA; ^2^Northwestern University, Chicago, IL, USA

###### **Correspondence:** Katharine Rendle (Katharine.Rendle@pennmedicine.upenn.edu)

*Implementation Science 2024*, **19(2):**S153

**Background:** Behavioral economics (BE) suggests that people’s emotions, habits, and cognitive biases can lead them to behave in predictably “irrational” ways. BE models and frameworks account for the fact that people lead busy lives, straining their attentions and leading them to sub-optimal decisions. However, they also recognize that these decisions are modifiable via changes in the choice architecture that can promote evidence-based decision-making. Several studies have demonstrated the benefits of nudges (implementation strategies informed by BE) on promoting evidence-based cancer care and doing so in a low-cost, scalable fashion. Despite BE’s benefits, it is not often integrated within implementation science (IS) research or frameworks.

**Methods:** The Penn Implementation Science Center in Cancer Control (Penn ISC3) integrates insights from BE and IS to rapidly accelerate the pace at which evidence-based practices for cancer care are deployed and the extent to which they are delivered equitably. Projects have included increasing screening for tobacco use and promoting referral to tobacco use treatment services (TUT), encouraging serious illness conversations, and more. All projects utilize the electronic medical record (EMR), which allows investigators to guide clinical decision-making without disrupting workflows or adding to clinician burden.

**Findings:** Penn ISC3’s research trials combining BE and IS have shown promising results, exemplified by a study in which TUT penetration nearly tripled after the implementation of a BE-informed clinician nudge in the EMR. Additionally, they have not exacerbated existing health disparities, aligning with our focus on health equity within IS. The integration of low-cost outreach systems, such as the EMR-based patient portal or text messages, can extend interventions’ reach and promote scalability.

**Implications for D&I Research:** Most research in IS and cancer care has not leveraged BE insights, presenting an opportunity to iterate on potential strategies to equitably and efficiently implement evidence-based cancer care. Utilizing theories and implementation strategies informed by BE can promote lower-cost, scalable interventions, while IS can strengthen the sustainability of BE efforts. IS and cancer research are at a critical time for innovation, and integrating BE can accelerate it.

**Primary Funding Source:** National Cancer Institute

### S154 Recommendations for addressing structural racism in implementation science: A call to the field

#### Rachel Shelton^1^, Prajakta Adsul^2^, April Oh^3^

##### ^1^Columbia University Mailman School of Public Health, New York, NY, USA; ^2^University of New Mexico, Albuquerque, NM, USA; ^3^National Institutes of Health, National Cancer Institute, Rockville, MD, USA

###### **Correspondence:** Rachel Shelton (rs3108@cumc.columbia.edu)

*Implementation Science 2024*, **19(2):**S154

**Link:** https://pubmed.ncbi.nlm.nih.gov/34045837/

